# A network-based pathway-extending approach using DNA methylation and gene expression data to identify altered pathways

**DOI:** 10.1038/s41598-019-48372-1

**Published:** 2019-08-14

**Authors:** Jie Li, Qiaosheng Zhang, Zhuo Chen, Dechen Xu, Yadong Wang

**Affiliations:** 1 Harbin Institute of Technology, School of Computer Science and Technology, Harbin, 150001 P.R. China; 2 Heilongjiang Bayi Agricultural University, College of Science, Daqing, 163319 P.R. China

**Keywords:** Cancer, Genetics

## Abstract

Pathway analysis allows us to gain insights into a comprehensive understanding of the molecular mechanisms underlying cancers. Currently, high-throughput multi-omics data and various types of large-scale biological networks enable us to identify cancer-related pathways by comprehensively analyzing these data. Combining information from multidimensional data, pathway databases and interaction networks is a promising strategy to identify cancer-related pathways. Here we present a novel network-based approach for integrative analysis of DNA methylation and gene expression data to extend original pathways. The results show that the extension of original pathways can provide a basis for discovering new components of the original pathway and understanding the crosstalk between pathways in a large-scale biological network. By inputting the gene lists of the extended pathways into the classical gene set analysis (ORA and FCS), we effectively identified the altered pathways which are correlated well with the corresponding cancer. The method is evaluated on three datasets retrieved from TCGA (BRCA, LUAD and COAD). The results show that the integration of DNA methylation and gene expression data through a network of known gene interactions is effective in identifying altered pathways.

## Introduction

Cancer etiology and progression is currently understood to be driven primarily by molecular and genetic mechanisms^1,2^. Cancer is caused by the interactions of multiple genes and pathways. Pathway analysis may help to understand the status of cancer and suggest customized anticancer therapies. Wang *et al*.^3^ classify pathway analysis methods into four main categories: overrepresentation analysis (ORA), functional class scoring (FCS), pathway topology (PT) - Based and network topology (NT) - Based.

ORA^4^ approaches assess whether the number of genes beyond an arbitrary threshold is significantly over- or under-represented in a pathway just by chance. Unlike ORA, FCS^5^ methods take into consideration all available molecular measurements for pathway analysis, such as GSEA(Gene Set Enrichment Analysis)^6^, ANCOVA(Analysis of Covariance)^7^, etc. PT-Based^8^ methods employ pathway topology between genes in signaling pathways to find which pathway is most impacted by a given phenotype. Moreover, the interaction databases, such as HPRD^9^, FunCoup^10^, STRING^11^, are also available. So, NT-Based^3^ methods extract interactions between genes from interaction databases or literature to compute pathway-level statistics.

Recent functional genomic experiments have found a large number of interactions between intra- and inter-pathways, suggesting more complex relationships between biological pathways than in their traditional representations. Therefore, it is necessary to embed original pathways into many large-scale networks to analyze pathways. Lu *et al*.^12^ embed original pathways within large-scale networks and demonstrate the crosstalk between them. Original pathways are extended by mapping genes of original pathways onto the network of biomolecules. The first neighbors of these genes are considered as new components of the original pathways. Glaab *et al*.^13^ present a methodology for extending original pathways by mapping them onto a protein-protein interaction network, and extending them to include densely interconnected interaction partners. However, these methods only consider network topologies and ignore edge weights of large-scale networks when extending pathways. Zhang *et al*.^14^ calculated the weights of a gene network through integrating DNA methylation and gene expression data to identify disease-associated gene modules. However, the biological roles of the gene modules discovered using the method are not clear. Paradigm^15,16^ integrates diverse high-throughput genomics information with a pathway structure to identify significant pathways. It has a limitation to extract different types of biological entities in the context of biological knowledge. And, this method only employs the pathway topology itself. Hence, how to combine information from multidimensional data, pathway databases and interaction networks is a promising strategy to identify altered pathways which have significant changes in different tissues, such as tumor and normal tissues.

**Figure 1:**
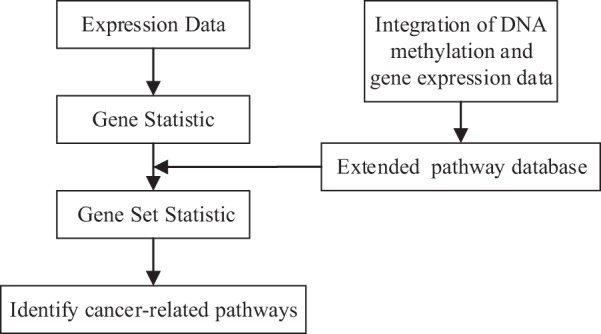
Overview of the method.

DNA methylation is known to be associated with gene transcription by interfering with DNA-binding proteins^17^. Hence we present a novel network-based approach for integrative analysis of DNA methylation and gene expression data to calculate edge weights of the large-scale network for each phenotype. Then, each pathway is extended by adding important neighboring genes based on the limited kWalks algorithm^18^ in weighted phenotype-specific networks. The pathway extended under different phenotypes is united as a final pathway gene list. Finally, by inputting the gene lists of extended pathways into the classical gene set analysis (ORA and FCS), we identify altered pathways which are correlated well with the corresponding cancer. The overview of our method is shown in Fig. 1.

## Materials and Methods

### Data

The PPI(Protein-Protein Interaction) network (version 2.9) was downloaded from the Interologous Interaction Database (I2D) website (http://ophid.utoronto.ca/ophidv2.204/downloads.jsp). Gene expression and DNA methylation data are obtained from TCGA (The Cancer Genome Atlas, https://portal.gdc.cancer.gov/projects). In this study, we have only chose samples that contain both gene expression and methylation data. According to data providers, all methylation data are from Illumina Human Methylation 450k Chip, whereas all gene expression data are downloaded from Agilent G4502A or Illumina HiSeq platform. BRCA (Breast Invasive Carcinoma) includes 33 cancer samples with DNA methylation and gene expression data, and 37 normal tissue samples. LUAD (Lung Adenocarcinoma) dataset consists of 69 samples (20 normal tissue samples and 49 cancer samples with DNA methylation and gene expression data). COAD (Colon Adenocarcinoma) data have 26 cancer samples with DNA methylation and gene expression data and 16 normal tissue samples). Gene expression data of the LUAD and COAD produced by Illumina HiSeq are added a value of 1 (to avoid zeros) and then log2-transformed. Gene sets of biological pathways are from the ConsensusPathDB website. A total of 281 KEGG pathways are obtained and further analyzed in the subsequent experiment.

### Construct the weighted gene-gene interaction network

**Figure 2:**
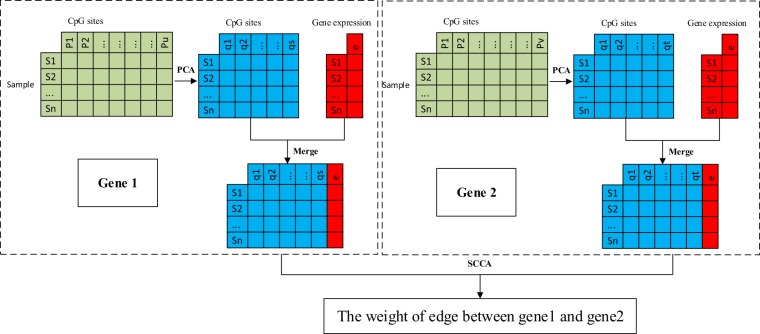
Calculation of gene pair weights in the network.

In this paper, PPI network is chose as a priori network. The edge weight between a pair of genes is calculated according to the PCA(Principal Component Analysis) and SCCA(sparse canonical correlation analysis) through integrating DNA methylation and gene expression data. At first, we do not set the cut-off of the gene expression and DNA methylation and treat each gene equally when building the weighted gene-gene interaction network. When calculating the weight of a gene pair in the network, if one of the two genes does not have the corresponding expression and methylation values, the edge is deleted, otherwise retained. Each gene contains multiple methylated CpG loci, and there is a general correlation between these neighboring CpG loci. In this study, PCA is used for dimensionality reduction of CpG loci for each gene firstly. Then, the selected principal components of CpG loci and gene expression are merged as the matrix of a gene. Finally, SCCA is used to calculate the edge weights of gene pairs in the network based on the principal components of CpG loci and gene expression values (see Fig. 2).

Let $X=({x}_{1}^{m},{x}_{2}^{m},\ldots ,{x}_{u}^{m})$ represent methylation values of gene 1, $Y=({y}_{1}^{m},{y}_{2}^{m},\ldots ,{y}_{v}^{m})$ represent methylation values of gene 2, where u and v are the number of CpG loci in genes 1 and 2 respectively. First, PCA is employed to reduce CpG loci dimension of genes 1 and 2 and calculated principal components of genes 1 and 2, $\bar{X}=({\bar{x}}_{1}^{m},{\bar{x}}_{2}^{m},\ldots ,{\bar{x}}_{s}^{m})$ and $\bar{Y}=({\bar{y}}_{1}^{m},{\bar{y}}_{2}^{m},\ldots ,{\bar{y}}_{t}^{m})$ respectively. Then $\bar{X}$ and the expression data of gene 1 are merged as a matrix. Similarly, $\bar{Y}$ and the expression data of gene 2 are merged as another matrix. As shown in Fig. 2, $\tilde{X}=({\bar{x}}_{1}^{m},{\bar{x}}_{2}^{m},\ldots ,{\bar{x}}_{s}^{m},{x}^{e})$ and $\mathop{Y}\limits^{ \sim }=({\bar{y}}_{1}^{m},{\bar{y}}_{2}^{m},\ldots ,{\bar{y}}_{t}^{m},{y}^{e})$ are matrices of genes 1 and 2 respectively, where ${x}^{e}$ and ${y}^{e}$ represent the expression values of genes 1 and 2 respectively. The edge weight between genes 1 and 2 is calculated as follow,

1 $${W}_{XY}=\frac{{cov}({a}^{T}\cdot \tilde{X},{b}^{T}\cdot \tilde{Y})}{\sqrt{{var}({a}^{T}\cdot \tilde{X})}\cdot \sqrt{{var}({b}^{T}\cdot \tilde{Y})}}$$

here a and b are optimized as follow,

2 $$\begin{array}{ll}{\rm{maximize}} & {a}^{T}{X}^{T}Yb\\ {\rm{subject}}\,{\rm{to}} & \parallel a{\parallel }_{2}^{2}\le 1,\parallel b{\parallel }_{2}^{2}\le 1,\parallel a{\parallel }_{1} < {c}_{1}\sqrt{p},\parallel b{\parallel }_{1} < {c}_{2}\sqrt{q}\end{array}$$

where ||·||_1_ and ||·||_2_ are L1 norm and L2 norm, respectively. *c*_1_ and *c*_2_ are parameters to regulate the amount of shrinkage and restricted to ranges $0 < {c}_{1} < 1$ and $0 < {c}_{2} < 1$, $p=s+1$, $q=t+1$. *W*_*XY*_ is calculated using PMA which is available as a Bioconductor package^19^.

### Extend pathway based on the weighted network

**Figure 3:**
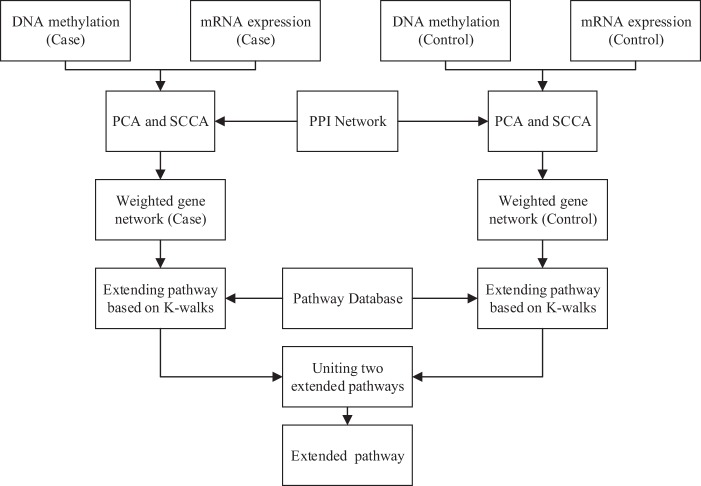
Construction of weighted phenotype-specific networks and extension of original pathways.

We construct the weighted gene-gene interaction networks for different phenotype (such as, normal tissue network and cancer tissue network), as shown in Fig. 3. We not only consider the relations of genes inside a pathway, but also the relation between genes inside and outside of a pathway. Therefore we extend each pathway based on the limited kWalks algorithm^18^ in gene-gene interaction network and the importance neighboring genes are added in the pathway. In the limited kWalks algorithm, the relevance of an edge and a node in relation to the pathway-sets is evaluated by the expected times random walk passes starting from one gene to any of the others. In the interpretation of a graph as a Markov chain, each gene represents a state, and the probability of transition from state i to j is given by

3 $${P}_{ij}=\frac{{W}_{ij}}{{\sum }_{j}\,{W}_{ij}}$$

where *W*_*ij*_ is edge weight of gene i - gene j. More details of the mathematics are available in ref.^20^. Finally, we extract two extended pathways genes from two weighted phenotype-specific networks, respectively. Two extended pathways genes under different phenotypes are united as an extended pathway gene list.

### Identify cancer-related pathways

To illustrate the benefits of our extended pathways, we use ORA and GSEA to analyse gene sets included in the extended pathways and identify the altered pathways which are correlated well with the corresponding cancer. In this paper, for convenience they will be referred to as EP-ORA (Extended Pathway ORA) and EP-GSEA (Extended Pathway GSEA).

Briefly, ORA methods compare sets of genes annotated to pathways and to a list of those genes that are significantly deferentially expressed (DE) between two phenotypes. Then a confidence value is calculated using statistical methods. Here, we calculate a P-value using the hypergeometric distribution.

4 $$P \mbox{-} value=1-\mathop{\sum }\limits_{i=0}^{k-1}\,\frac{(\begin{array}{c}M\\ i\end{array})(\begin{array}{c}N-M\\ n-i\end{array})}{(\begin{array}{c}N\\ i\end{array})}$$

Where N is the total number of genes in the background distribution, M is the number of all DE genes, n is the size of the list of genes of the pathway and k is the number of DE genes within the pathway. Finally, BH (Benjamini-Hochberg) correction for multiple testing is performed^21^.

Another approach, GSEA^6^ is an FCS-type method that determines whether a priori defined set of genes shows statistically significant, concordant differences between two biological states, which uses all available molecular measurements for pathway analysis. GSEA works as follows:

1. Sort genes by signal-to-noise ratio;
2. Calculate enrichment scores;
3. Permute 1000 phenotype labels for significance.

## Results

### Extension of original pathways with large-scale network predicts new pathway components

In general, functionally linked interacting genes have a significantly higher level of coherence in biological systems^22^. The pathway neighboring genes may play important roles in the regulation of disease-related pathways. The inclusion of important neighboring genes will enable us to understand cancer mechanisms with models of pathway activities. One hypothesis of the proposed method is that the genetic interactions are variables between controls and cases which is responsible for different phenotypes varying in cancer. Hence, two weighted gene-gene interaction networks are then achieved based on case samples and control samples, respectively. All genes that interact with the pathway contribute to the regulation of the pathway. So, genes of two extended pathways under different phenotypes are eventually united as a final extended pathway gene set.

**Figure 4:**
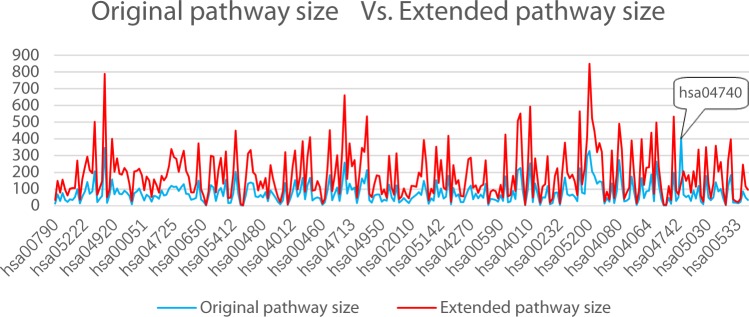
Comparison of the original pathway sizes and the extended pathway sizes.

To test the effectiveness of the proposed method, we first take BRCA dataset for a comparative evaluation. As shown in Fig. 4, the extended pathways can systematically indicate new genes involved in original pathways. The pathway sizes increased on average from 28.30% to 224.56% of the original size except for hsa04740 (Olfactory transduction). The hsa04740 is closely related to multiple protein isoforms and include 405 genes, but only 54 genes are mapped to the weight network. Finally, the extended hsa04740 includes 138 genes.

**Figure 5:**
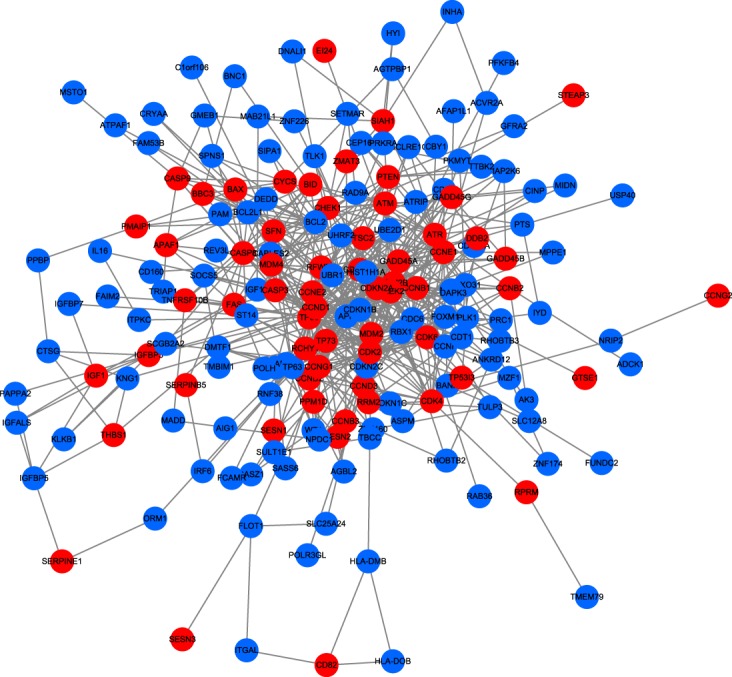
The p53 signaling pathway (hsa04115) is extended in the weighted network. Red nodes denote genes in original pathway and blue nodes denote the extended genes that are most associated with the corresponding pathway.

The extended p53 signaling pathway is illustrated in Fig. 5, because of its importance for cancer analysis. A total of 68 genes in the p53 signaling pathway are mapped onto the large-scale PPI network. The result show that the extension algorithm identifies 120 new genes which are important neighboring genes of the p53 signaling pathway. Hence, the extension of original pathways can provide a basis for discovering new candidate components of the original pathway.

### Pathway identification in breast cancer

One of the important applications of pathway analysis is to identify altered pathways which are correlated well with the corresponding cancer. Here, we firstly take BRCA dataset for a comparative evaluation. We apply ORA and EP-ORA to this dataset with the BH corrected P-value. Using a P-value cutoff of 0.05, ORA and EP-ORA result in picking 6 and 18 pathways as significant, respectively (Supplementary file, Table S1). Both methods have effectively identified Cell cycle and Focal adhesion which have been confirmed by the published literatures to be closely associated with breast cancer (see Table 1). The above results show that the overlapped pathways found by different methods can be used as robust cancer-related pathways. Several pathways well known to be related to breast cancer are only identified by EP-ORA, such as p53 signaling pathway, DNA replication, Pathways in cancer, B cell receptor signaling pathway, etc. Interestingly, the p53 signaling pathway is identified by EP-ORA. Abundant data from mechanistic, molecular pathological and transgenic animal studies support an important role for p53 in mammary carcinogenesis^23^.

**Table 1:** Significant pathways identified in BRCA dataset using ORA and EP-ORA.

Pathway ID	Pathway Name	EP-ORA		ORA	
		Ad. Pvalue	Rank	Ad. Pvalue	Rank
hsa03030	DNA replication^29^	0.001145	1	0.114869	11
hsa04110	Cell cycle^30^	0.004948	2	0.004196	1
hsa05200	Pathways in cancer*	0.004948	2	0.072255	7
hsa00250	Alanine, aspartate and glutamate metabolism^31^	0.0157	4	0.495336	72
hsa04120	Ubiquitin mediated proteolysis^32^	0.024373	5	0.090582	8
hsa00350	Tyrosine metabolism^33^	0.024373	5	0.278437	29
hsa04114	Oocyte meiosis^34^	0.024373	5	0.319667	39
hsa04662	B cell receptor signaling pathway^35^	0.024373	5	0.561115	85
hsa04810	Regulation of actin cytoskeleton^36^	0.024373	5	0.124388	13
hsa05214	Glioma^37^	0.024373	5	0.468975	52
hsa04510	Focal adhesion^38,39^	0.024373	5	0.021221	4
hsa00230	Purine metabolism^40^	0.024373	5	0.468975	52
hsa00240	Pyrimidine metabolism	0.030055	13	0.522621	81
hsa04360	Axon guidance^41^	0.031465	14	0.099764	10
hsa04115	p53 signaling pathway^23^	0.03901	15	0.36821	49
hsa05223	Non-small cell lung cancer^42^	0.040912	16	0.544195	84
hsa04914	Progesterone-mediated oocyte maturation	0.040912	16	0.319667	39
hsa05222	Small cell lung cancer^43^	0.040912	16	0.151603	17

Note: The asterisk-labeled pathways have been confirmed to be associated with cancer by biologists.

We then apply GSEA and EP-GSEA to the BRCA dataset. In standard GSEA, the analysis performs 1000 permutations using case-control gene expression samples (case 33 vs. control 37) and original pathways with an FDR cutoff of 25%. However, no pathway is identified (see Table 2). It is probably a consequence of the low power issue related to GSEA methodology^24^. Subsequently, we use the same expression dataset and extended pathways for EP-GSEA analysis. The results show that 3 pathways are identified (see Table 2). These three pathways are closely related to breast cancer, which have been verified in many published studies. For example, Li *et al*.^25^ point out that the metabolism of xenobiotics by cytochrome P450 and drug metabolism-cytochrome P450 enzymes in breast tissues may play important roles in breast cancer risk.

**Table 2:** Significant pathways identified in BRCA dataset using GSEA and EP-GSEA.

	Pathway ID	Pathway Name	SIZE	ES	NES	NOM p-val	FDR q-val
GSEA	—	—	—	—	—	—	—
	hsa00980	Metabolism of xenobiotics by cytochrome P450^25,44^	123	0.4314	1.7249	0.0061	0.2165
EP-GSEA	hsa00982	Drug metabolism - cytochrome P450^25^	117	0.4245	1.6614	0.0182	0.2251
	hsa03440	Homologous recombination^45^	64	−0.5635	−1.8259	0	0.1725

Taken together, in comparison to ORA and GSEA, EP-ORA and EP-GSEA using extended pathways can more effectively identify cancer-related pathways for breast cancer.

### Examining crosstalk between embedded pathways

Cancer is a complex disease involving a sequence of gene-gene interactions in a progressive process, which cannot occur without dysregulation in multiple biological pathways. From a systems biology perspective, biological pathways are connected together by crosstalk to perform a specific biological function as a system. In biology, the pathway crosstalk means that signal components in signal transduction can be shared between different biological pathways, and responses to a signal inducing condition can activate multiple responses in cells, tissues, or organisms^12^. Therefore, understanding the crosstalk between pathways is important for understanding the function of both cells and more complex diseases. Now, we embed original and extended pathways into large-scale biological networks and show the crosstalk between them.

**Figure 6:**
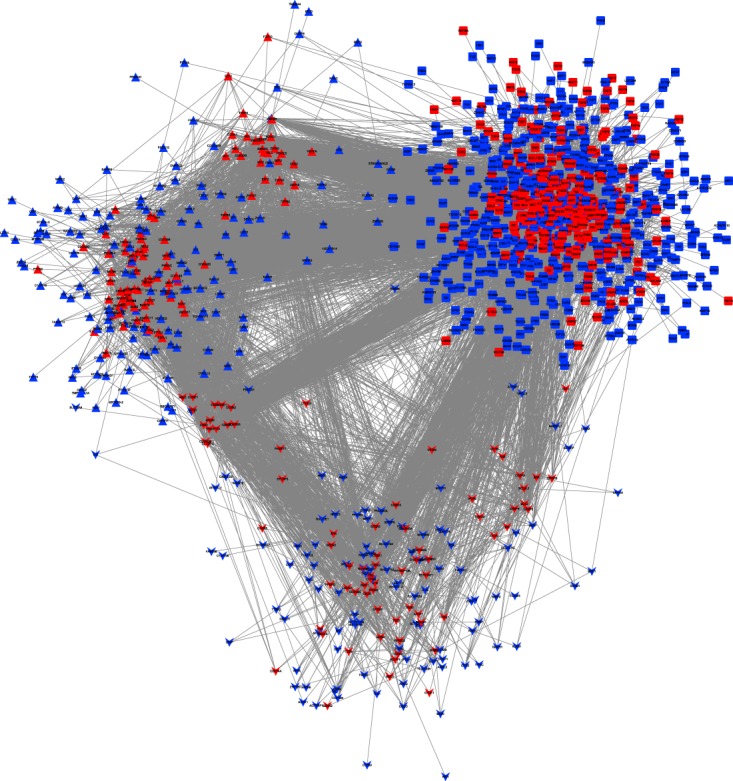
The crosstalk between three extended pathways. The upper triangular shape nodes represent the cell cycle pathway (hsa04110), the lower triangular shape nodes represent the p53 signaling pathway (hsa04115), the square nodes represent the pathways in cancer (hsa05200). Red nodes denote genes in original pathway and blue nodes denote the extended genes that are most associated with the corresponding pathway.

As an example, for these types of connections, we map three pathways, cell cycle, p53 signaling pathway and pathways in cancer, onto the large-scale biological network (see Fig. 6). The crosstalk between the three pathways suggests that they may share similar functions in breast cancer. The above results show that a large number of genes exist as linkers between pathways. Accordingly, a careful examination of these intermediate genes may help reveal the mechanisms underlying the interconnection of different pathways. Many genes in the large-scale network are well connected with different pathways, and may therefore play a functional role in the communication between the pathways.

### Validation of the alternative dataset

To further verify the improvement of EP-ORA, EP-GSEA over ORA, GSEA. Using the same process as above, we apply the method in this article to other two datasets (LUAD and COAD).

The results of lung adenocarcinoma data (LUAD) are shown in Tables 3 and 4 (see Supplementary Tables S3 and S4 for more details). The results show that a total of three pathways are overlapped by EP-ORA and ORA (adjusted P-value ≤ 0.05). The bile secretion pathway related to lung cancer is only identified by EP-ORA. For the bile secretion pathway, Liu *et al*.^26^ reported that bile acid receptor accelerates to the lung cancer process induced by lung fibroblast-tumor cells interaction, with high activation of phosphorylated STAT3 and alteration of cytokine secretion. Compared with GSEA, EP-GSEA identifies more pathways which are closely related to lung cancer (FDR ≤ 25%). Interestingly, the non-small cell lung cancer pathway is only identified by EP-GSEA.

**Table 3:** Significant pathways identified in LUAD dataset using ORA and EP-ORA.

Pathway ID	Pathway Name	EP-ORA		ORA	
		Ad. Pvalue	Rank	Ad. Pvalue	Rank
hsa03030	DNA replication^46^	3.37E-05	1	0.03242	1
hsa04976	Bile secretion^26^	0.006128	2	0.913641	74
hsa03008	Ribosome biogenesis in eukaryotes	0.006128	2	0.03242	1
hsa04110	Cell cycle*	0.035295	4	0.03736	3
hsa03013	RNA transport^47^	0.042657	5	0.260906	8

Note: The asterisk-labeled pathways have been confirmed to be associated with cancer by biologists.

**Table 4:** Significant pathways identified in LUAD dataset using GSEA and EP-GSEA.

Pathway ID	Pathway Name	EP-GSEA		GSEA	
		FDR q-val	Rank	FDR q-val	Rank
hsa03430	Mismatch repair^48^	7.51E-02	1	0.159836	4
hsa03030	DNA replication*	0.13927312	4	0.178407	12
hsa03320	PPAR signaling pathway^49^	0.15703186	8	0.335938	94
hsa04514	Cell adhesion molecules (CAMs)^50^	0.16466248	12	0.327053	81
hsa04390	Hippo signaling pathway^51^	0.16826709	18	0.348485	101
hsa05217	Basal cell carcinoma^52^	0.17509021	40	0.448808	152
hsa04010	MAPK signaling pathway^53^	0.17821518	59	0.287580	51
hsa04310	Wnt signaling pathway^54^	0.17876078	60	0.349692	102
hsa04014	Ras signaling pathway^55^	0.18037082	66	0.327	80
hsa04110	Cell cycle^56^	0.18181872	71	0.105239	2
hsa05200	Pathways in cancer*	0.22708593	94	0.517886	176
hsa05223	Non-small cell lung cancer*	0.24327828	105	0.785674	239

Note: The asterisk-labeled pathways have been confirmed to be associated with cancer by biologists.

It is interesting to check pathways that are ranked top by one approach but not by the other approaches, which should reflect the different effects of the two approaches. Accordingly, corrected P-value is used to rank pathways. Focusing on colon adenocarcinoma (COAD), we apply ORA and EP-ORA to COAD dataset (see Supplementary Table S5 for more details). Here, we deliberately select several pathways related to CRC (Colorectal cancer) that have been widely confirmed in literatures. As shown in Table 5, most of the CRC-related pathways obtained tend to be ranked higher with EP-ORA than with ORA. For example, MicroRNAs in cancer, Cell cycle, Pathways in cancer and p53 signaling pathway, ranked 1, 2, 4 and 20 by EP-ORA, are ranked 9, 6, 27 and 57 by ORA, respectively. Interestingly, the colorectal cancer pathway is ranked 17 by EP-ORA, but ranked only 79 by ORA. The pathways that rank lower in EP-ORA are mostly not associated with the corresponding cancer. For example, the Parkinson’s disease pathway(hsa05012) which has been confirmed by the published literature^27^ to be inversely associated with colon cancer is ranked 2 by ORA, but ranked 53 by EP-ORA(see Supplementary Table S5), and so on.

**Table 5:** Significant pathways identified in COAD dataset using ORA and EP-ORA.

Pathway ID	Pathway Name	EP-ORA		ORA	
		Ad. Pvalue	Rank	Ad. Pvalue	Rank
hsa05206	MicroRNAs in cancer^57^	8.17E-03	1	0.081369	9
hsa04110	Cell cycle^58^	0.033908	2	0.034836	6
hsa05200	Pathways in cancer*	0.139902	4	0.157574	27
hsa05214	Glioma^59^	0.139902	4	0.087544	12
hsa03030	DNA replication^60^	0.139902	4	0.023618	4
hsa03013	RNA transport^61^	0.139902	4	0.087544	12
hsa05210	Colorectal cancer*	0.199796	17	0.457577	79
hsa04115	p53 signaling pathway^62^	0.2504	20	0.328427	57

Note: The asterisk-labeled pathways have been confirmed to be associated with cancer by biologists.

We then apply GSEA and EP-GSEA to the COAD dataset. Most of the CRC-related pathways are also ranked higher in EP-GSEA than in GSEA (see Table 6). The only exception to this is the p53 signaling pathway ranked 7 by the GSEA, but ranked only 137 by EP-GSEA (see Supplementary Table S6 for more details).

**Table 6:** Significant pathways identified in COAD dataset using GSEA and EP-GSEA.

Pathway ID	Pathway Name	EP-GSEA		GSEA	
		FDR q-val	Rank	FDR q-val	Rank
hsa03008	Ribosome biogenesis in eukaryotes^63^	0.057690	1	0.104035	1
hsa03430	Mismatch repair^64^	6.78E-02	2	0.138024	13
hsa03030	DNA replication*	0.082642	3	0.108252	4
hsa04110	Cell cycle*	0.090359	4	0.1261105	11
hsa05210	Colorectal cancer*	0.314139	88	0.5851279	193
hsa04115	p53 signaling pathway*	0.37533	137	0.113597	7
hsa05200	Pathways in cancer*	0.515173	207	0.664264	230

Note: The asterisk-labeled pathways have been confirmed to be associated with cancer by biologists.

The experimental results demonstrate that more and ranked top pathways found by the proposed method are cancer-related pathways which are supported by the published literatures based on biological experiments. In conclusion, compared with ORA and GSEA, EP-ORA and EP-GSEA can more effectively identify cancer-related pathways for different datasets.

## Discussion

The pathway-based analysis is an effective technique that overcomes the limitations of the current single-locus methods. This procedure provides a comprehensive understanding of the molecular mechanisms that cause complex diseases^28^. Currently, a major pathway analysis challenge in the context of cancer research is how to integrate and analyze various types of -omics data and large-scale biological networks to identify cancer-related pathways.

We present a novel network-based approach for integrative analysis of DNA methylation and gene expression data to extend classical pathways. Our method can effectively identify altered pathways which are correlated well with the corresponding cancer by inputting the gene lists of extended pathways into the classical gene set analysis (ORA and FCS) on three datasets (BRCA, LUAD and COAD). By applying the method to the breast cancer dataset, we demonstrate the method’s potential to identify breast cancer-related pathways. The analysis of colorectal cancer and lung adenocarcinoma confirm the proposed method’s ability to correctly identify cancer-related pathways in different cancer datasets. This suggests that the integration of DNA methylation and gene expression through a known gene interactions network is effective in pathway analysis. In the future, we will employ more datasets to assess the validity of our method. Readers can download our code from the website (https://github.com/ZHANGQiaosheng/IaPathway).

## Supplementary information

Supplementary Table S1

Supplementary Table S2

Supplementary Table S3

Supplementary Table S4

Supplementary Table S5

Supplementary Table S6

## Data Availability

The data supporting the findings of this work are contained within the manuscript.
