# Supplementary material for: A network-based pathway-extending approach using DNA methylation and gene expression data to identify altered pathways
Source: Sci Rep. 2019 Aug 14;9:11853. doi: 10.1038/s41598-019-48372-1 (PMC6694157; doi:10.1038/s41598-019-48372-1)
Supplement: Supplementary file 1 — Supplementary Table S1 [file 41598_2019_48372_MOESM1_ESM.pdf]

A network-based pathway-extending approach using DNA methylation and gene expression data to identify altered pathways

Jie Li[1], Qiaosheng Zhang[1,2,*], Zhuo Chen[1], Dechen Xu[1], and Yadong Wang[1]

[1]Harbin Institute of Technology, School of Computer Science and Technology, Harbin, 150001, P.R. China

[2]Heilongjiang Bayi Agricultural University, College of Science, Daqing, 163319, P.R. China

[*]zqs@hit.edu.cn

All results in BRCA dataset by EP-ORA

Pathway ID	Pathway Name	Universe.Size	Gene.Set.Size	Total.Hits	Expected.Hits	Observed.Hits	Pvalue	Adjusted.Pvalue	Rank
hsa03030	DNA replication	17814	88	8057	39.80106	61	4.09E-06	0.001145	1
hsa04110	Cell cycle	17814	299	8057	135.2331	169	5.06E-05	0.004948	2
hsa05200	Pathways in cancer	17814	848	8057	383.5374	439	5.30E-05	0.004948	2
hsa00250	Alanine, aspartate and glutamate metabolism	17814	96	8057	43.41933	61	0.000224	0.0157	4
hsa04120	Ubiquitin mediated proteolysis	17814	320	8057	144.7311	174	0.000573	0.024373	5
hsa00350	Tyrosine metabolism	17814	93	8057	42.06248	58	0.000639	0.024373	5
hsa04114	Oocyte meiosis	17814	293	8057	132.5194	160	0.000725	0.024373	5
hsa04662	B cell receptor signaling pathway	17814	192	8057	86.83867	109	0.000817	0.024373	5
hsa04810	Regulation of actin cytoskeleton	17814	535	8057	241.9723	278	0.000886	0.024373	5
hsa05214	Glioma	17814	175	8057	79.14983	100	0.000974	0.024373	5
hsa04510	Focal adhesion	17814	501	8057	226.5946	261	0.001036	0.024373	5
hsa00230	Purine metabolism	17814	378	8057	170.9636	201	0.001045	0.024373	5
hsa00240	Pyrimidine metabolism	17814	222	8057	100.4072	123	0.001395	0.030055	13
hsa04360	Axon guidance	17814	299	8057	135.2331	161	0.001573	0.031465	14
hsa04115	p53 signaling pathway	17814	182	8057	82.31582	102	0.00209	0.03901	15
hsa05223	Non-small cell lung cancer	17814	159	8057	71.91327	90	0.002496	0.040912	16
hsa04914	Progesterone-mediated oocyte maturation	17814	247	8057	111.7143	134	0.002577	0.040912	16
hsa05222	Small cell lung cancer	17814	233	8057	105.3823	127	0.00263	0.040912	16
hsa04910	Insulin signaling pathway	17814	359	8057	162.3702	188	0.00362	0.050947	19
hsa04015	Rap1 signaling pathway	17814	508	8057	229.7606	260	0.003639	0.050947	19
hsa03440	Homologous recombination	17814	64	8057	28.94622	40	0.004	0.053331	21
hsa00630	Glyoxylate and dicarboxylate metabolism	17814	61	8057	27.58937	38	0.005378	0.06426	22
hsa00480	Glutathione metabolism	17814	107	8057	48.39447	62	0.00543	0.06426	22
hsa00510	N-Glycan biosynthesis	17814	109	8057	49.29903	63	0.005508	0.06426	22
hsa04152	AMPK signaling pathway	17814	314	8057	142.0174	164	0.007105	0.079578	25
hsa00360	Phenylalanine metabolism	17814	49	8057	22.16195	31	0.008328	0.079695	26
hsa04060	Cytokine-cytokine receptor interaction	17814	498	8057	225.2378	252	0.008343	0.079695	26

hsa04064	NF-kappa B signaling pathway	17814	231	8057	104.4778	123	0.008372	0.079695	26
hsa04068	FoxO signaling pathway	17814	373	8057	168.7022	192	0.008384	0.079695	26
hsa04070	Phosphatidylinositol signaling system	17814	225	8057	101.7641	120	0.008539	0.079695	26
hsa04512	ECM-receptor interaction	17814	195	8057	88.19552	105	0.009309	0.083043	31
hsa04012	ErbB signaling pathway	17814	238	8057	107.6438	126	0.009753	0.083043	31
hsa04530	Tight junction	17814	312	8057	141.1128	162	0.009787	0.083043	31
hsa04670	Leukocyte transendothelial migration	17814	272	8057	123.0214	142	0.011803	0.096077	34
hsa03460	Fanconi anemia pathway	17814	106	8057	47.94218	60	0.01201	0.096077	34
hsa04270	Vascular smooth muscle contraction	17814	288	8057	130.258	149	0.014897	0.110702	36
hsa04014	Ras signaling pathway	17814	552	8057	249.6612	275	0.015612	0.110702	36
hsa04978	Mineral absorption	17814	115	8057	52.01274	64	0.015618	0.110702	36
hsa05166	HTLV-I infection	17814	660	8057	298.5079	326	0.015862	0.110702	36
hsa03015	mRNA surveillance pathway	17814	198	8057	89.55237	105	0.01612	0.110702	36
hsa04390	Hippo signaling pathway	17814	353	8057	159.6565	180	0.01621	0.110702	36
hsa00910	Nitrogen metabolism	17814	47	8057	21.25738	29	0.01691	0.11196	42
hsa00030	Pentose phosphate pathway	17814	72	8057	32.5645	42	0.017194	0.11196	42
hsa05205	Proteoglycans in cancer	17814	564	8057	255.0886	280	0.018069	0.114983	44
hsa00640	Propanoate metabolism	17814	88	8057	39.80106	50	0.018868	0.117245	45
hsa05219	Bladder cancer	17814	100	8057	45.22847	56	0.019473	0.117245	45
hsa00970	Aminoacyl-tRNA biosynthesis	17814	114	8057	51.56046	63	0.01968	0.117245	45
hsa04666	Fc gamma R-mediated phagocytosis	17814	226	8057	102.2163	118	0.020114	0.117332	48
hsa04261	Adrenergic signaling in cardiomyocytes	17814	393	8057	177.7479	198	0.021651	0.123721	49
hsa04062	Chemokine signaling pathway	17814	438	8057	198.1007	219	0.023871	0.133677	50
hsa03410	Base excision repair	17814	91	8057	41.15791	51	0.02452	0.134621	51
hsa04010	MAPK signaling pathway	17814	593	8057	268.2048	292	0.025466	0.136042	52
hsa04151	PI3K-Akt signaling pathway	17814	789	8057	356.8526	384	0.025751	0.136042	52
hsa04310	Wnt signaling pathway	17814	334	8057	151.0631	169	0.026691	0.138397	54
hsa04977	Vitamin digestion and absorption	17814	53	8057	23.97109	31	0.035849	0.181371	55

hsa00520	Amino sugar and nucleotide sugar metabolism	17814	125	8057	56.53559	67	0.03646	0.181371	55
hsa03430	Mismatch repair	17814	57	8057	25.78023	33	0.036922	0.181371	55
hsa05206	MicroRNAs in	17814	409	8057	184.9845	203	0.039376	0.190089	58
hsa05211	Renal cell carcinoma	17814	175	8057	79.14983	91	0.041881	0.197436	59
hsa05218	Melanoma	17814	173	8057	78.24526	90	0.042308	0.197436	59
hsa00533	Glycosaminoglycan biosynthesis - keratan sulfate	17814	23	8057	10.40255	15	0.043023	0.197481	61
hsa04150	mTOR signaling pathway	17814	147	8057	66.48585	77	0.048114	0.206664	62
hsa00270	Cysteine and methionine metabolism	17814	98	8057	44.3239	53	0.048376	0.206664	62
hsa00563	Glycosylphosphatidylinositol(GPI)-anchor biosynthesis	17814	54	8057	24.42337	31	0.048376	0.206664	62
hsa05146	Amoebiasis	17814	274	8057	123.926	138	0.048662	0.206664	62
hsa00603	Glycosphingolipid biosynthesis - globo series	17814	27	8057	12.21169	17	0.048714	0.206664	62
hsa04630	Jak-STAT signaling pathway	17814	325	8057	146.9925	162	0.051592	0.215608	67
hsa04370	VEGF signaling pathway	17814	160	8057	72.36556	83	0.053225	0.218282	68
hsa00562	Inositol phosphate metabolism	17814	158	8057	71.46099	82	0.053791	0.218282	68
hsa05168	Herpes simplex infection	17814	398	8057	180.0093	196	0.057515	0.227808	70
hsa04917	Prolactin signaling pathway	17814	223	8057	100.8595	113	0.057766	0.227808	70
hsa04915	Estrogen signaling pathway	17814	278	8057	125.7352	139	0.060768	0.229871	72
hsa00760	Nicotinate and nicotinamide metabolism	17814	45	8057	20.35281	26	0.061667	0.229871	72
hsa00860	Porphyrin and chlorophyll metabolism	17814	85	8057	38.4442	46	0.061944	0.229871	72
hsa04921	Oxytocin signaling pathway	17814	401	8057	181.3662	197	0.062498	0.229871	72
hsa00410	beta-Alanine metabolism	17814	81	8057	36.63506	44	0.062591	0.229871	72
hsa00010	Glycolysis / Gluconeogenesis	17814	184	8057	83.22039	94	0.063215	0.229871	72
hsa05217	Basal cell carcinoma	17814	118	8057	53.3696	62	0.065977	0.23684	78
hsa00260	Glycine, serine and threonine metabolism	17814	108	8057	48.84675	57	0.069191	0.240648	79
hsa05215	Prostate cancer	17814	256	8057	115.7849	128	0.069433	0.240648	79
hsa05220	Chronic myeloid leukemia	17814	214	8057	96.78893	108	0.069684	0.240648	79

hsa03420	Nucleotide excision repair	17814	104	8057	47.03761	55	0.070476	0.240648	79
hsa05221	Acute myeloid leukemia	17814	185	8057	83.67267	94	0.072504	0.244592	83
hsa05169	Epstein-Barr virus infection	17814	533	8057	241.0678	258	0.07343	0.244767	84
hsa00590	Arachidonic acid metabolism	17814	125	8057	56.53559	65	0.075742	0.249501	85
hsa00052	Galactose metabolism	17814	78	8057	35.27821	42	0.078326	0.25386	86
hsa04142	Lysosome	17814	291	8057	131.6149	144	0.079271	0.25386	86
hsa00601	Glycosphingolipid biosynthesis - lacto and neolacto series	17814	38	8057	17.18682	22	0.079979	0.25386	86
hsa03060	Protein export	17814	66	8057	29.85079	36	0.081109	0.25386	86
hsa00280	Valine, leucine and isoleucine degradation	17814	109	8057	49.29903	57	0.082537	0.25386	86
hsa04664	Fc epsilon RI signaling pathway	17814	186	8057	84.12496	94	0.082746	0.25386	86
hsa00620	Pyruvate	17814	107	8057	48.39447	56	0.083411	0.25386	86
hsa05204	Chemical carcinogenesis	17814	128	8057	57.89244	66	0.087822	0.264411	93
hsa05162	Measles	17814	331	8057	149.7062	162	0.094474	0.280785	94
hsa03013	RNA transport	17814	346	8057	156.4905	169	0.095266	0.280785	94
hsa05110	Vibrio cholerae infection	17814	137	8057	61.96301	70	0.097236	0.283606	96
hsa00340	Histidine	17814	67	8057	30.30308	36	0.100874	0.291182	97
hsa00380	Tryptophan metabolism	17814	102	8057	46.13304	53	0.102223	0.292065	98
hsa04520	Adherens junction	17814	209	8057	94.52751	104	0.10503	0.293875	99
hsa05161	Hepatitis B	17814	375	8057	169.6068	182	0.106305	0.293875	99
hsa04614	Renin-angiotensin system	17814	47	8057	21.25738	26	0.106811	0.293875	99
hsa00450	Selenocompound metabolism	17814	45	8057	20.35281	25	0.107055	0.293875	99
hsa04920	Adipocytokine signaling pathway	17814	203	8057	91.8138	101	0.10915	0.296559	103
hsa00982	Drug metabolism - cytochrome P450	17814	117	8057	52.91731	60	0.110151	0.296559	103
hsa05160	Hepatitis C	17814	320	8057	144.7311	156	0.111263	0.296702	105
hsa05132	Salmonella infection	17814	210	8057	94.97979	104	0.117491	0.305615	106
hsa05134	Legionellosis	17814	151	8057	68.29499	76	0.118541	0.305615	106
hsa05100	Bacterial invasion of epithelial cells	17814	208	8057	94.07522	103	0.119018	0.305615	106
hsa05212	Pancreatic cancer	17814	206	8057	93.17065	102	0.120568	0.305615	106
hsa04540	Gap junction	17814	223	8057	100.8595	110	0.121148	0.305615	106
hsa04972	Pancreatic secretion	17814	221	8057	99.95492	109	0.122713	0.305615	106
hsa04668	TNF signaling pathway	17814	302	8057	136.59	147	0.124027	0.305615	106
hsa05210	Colorectal cancer	17814	183	8057	82.7681	91	0.12431	0.305615	106
hsa00561	Glycerolipid metabolism	17814	122	8057	55.17874	62	0.124429	0.305615	106

hsa03320	PPAR signaling pathway	17814	198	8057	89.55237	98	0.12701	0.309241	115
hsa04022	cGMP-PKG signaling pathway	17814	399	8057	180.4616	192	0.130804	0.315735	116
hsa00300	Lysine biosynthesis	17814	5	8057	2.261424	4	0.133489	0.319461	117
hsa00040	Pentose and glucuronate interconversions	17814	48	8057	21.70967	26	0.135643	0.321864	118
hsa04611	Platelet activation	17814	329	8057	148.8017	159	0.139163	0.326023	119
hsa05322	Systemic lupus erythematosus	17814	201	8057	90.90923	99	0.139724	0.326023	119
hsa00564	Glycerophospholipid metabolism	17814	180	8057	81.41125	89	0.143047	0.328354	121
hsa00980	Metabolism of xenobiotics by cytochrome P450	17814	123	8057	55.63102	62	0.143069	0.328354	121
hsa05202	Transcriptional misregulation in cancer	17814	446	8057	201.719	213	0.149481	0.340282	123
hsa05203	Viral carcinogenesis	17814	522	8057	236.0926	248	0.154305	0.341323	124
hsa04975	Fat digestion and absorption	17814	90	8057	40.70562	46	0.154369	0.341323	124
hsa03040	Spliceosome	17814	234	8057	105.8346	114	0.155426	0.341323	124
hsa00290	Valine, leucine and isoleucine biosynthesis	17814	7	8057	3.165993	5	0.155874	0.341323	124
hsa04722	Neurotrophin signaling pathway	17814	339	8057	153.3245	163	0.156033	0.341323	124
hsa04144	Endocytosis	17814	449	8057	203.0758	214	0.158389	0.341824	129
hsa00140	Steroid hormone biosynthesis	17814	86	8057	38.89649	44	0.158704	0.341824	129
hsa04912	GnRH signaling pathway	17814	243	8057	109.9052	118	0.162145	0.346569	131
hsa00670	One carbon pool by folate	17814	53	8057	23.97109	28	0.164654	0.349266	132
hsa00983	Drug metabolism - other enzymes	17814	78	8057	35.27821	40	0.167839	0.351237	133
hsa00400	Phenylalanine, tyrosine and tryptophan biosynthesis	17814	9	8057	4.070562	6	0.169319	0.351237	133
hsa00830	Retinol metabolism	17814	99	8057	44.77619	50	0.169347	0.351237	133
hsa04622	RIG-I-like receptor signaling pathway	17814	154	8057	69.65185	76	0.170762	0.351569	136
hsa04960	Aldosterone-regulated sodium reabsorption	17814	95	8057	42.96705	48	0.174324	0.353984	137
hsa00053	Ascorbate and aldarate metabolism	17814	43	8057	19.44824	23	0.174494	0.353984	137
hsa05014	Amyotrophic lateral sclerosis (ALS)	17814	148	8057	66.93814	73	0.178078	0.353984	137
hsa05216	Thyroid cancer	17814	91	8057	41.15791	46	0.179499	0.353984	137
hsa05144	Malaria	17814	129	8057	58.34473	64	0.179928	0.353984	137

hsa05130	Pathogenic Escherichia coli infection	17814	146	8057	66.03357	72	0.180604	0.353984	137
hsa04750	Inflammatory mediator regulation of TRP channels	17814	270	8057	122.1169	130	0.181419	0.353984	137
hsa03018	RNA degradation	17814	161	8057	72.81784	79	0.182942	0.353984	137
hsa00310	Lysine degradation	17814	108	8057	48.84675	54	0.183313	0.353984	137
hsa00130	Ubiquinone and other terpenoid-quinone	17814	29	8057	13.11626	16	0.186529	0.354005	146
hsa04514	Cell adhesion molecules (CAMs)	17814	294	8057	132.9717	141	0.186774	0.354005	146
hsa04720	Long-term potentiation	17814	174	8057	78.69754	85	0.187117	0.354005	146
hsa00750	Vitamin B6 metabolism	17814	17	8057	7.68884	10	0.188456	0.354146	149
hsa04725	Cholinergic synapse	17814	290	8057	131.1626	139	0.191276	0.357048	150
hsa00020	Citrate cycle (TCA cycle)	17814	79	8057	35.73049	40	0.196352	0.364096	151
hsa04260	Cardiac muscle contraction	17814	179	8057	80.95896	87	0.201319	0.37085	152
hsa04350	TGF-beta signaling pathway	17814	205	8057	92.71837	99	0.207063	0.378939	153
hsa05414	Dilated cardiomyopathy	17814	218	8057	98.59807	105	0.209338	0.380615	154
hsa03010	Ribosome	17814	322	8057	145.6357	153	0.218806	0.395262	155
hsa05213	Endometrial cancer	17814	150	8057	67.84271	73	0.221199	0.397024	156
hsa00740	Riboflavin metabolism	17814	34	8057	15.37768	18	0.231567	0.412986	157
hsa00900	Terpenoid backbone biosynthesis	17814	57	8057	25.78023	29	0.233779	0.414292	158
hsa05133	Pertussis	17814	205	8057	92.71837	98	0.249541	0.439443	159
hsa04930	Type II diabetes mellitus	17814	132	8057	59.70158	64	0.252058	0.440873	160
hsa04660	T cell receptor signaling pathway	17814	283	8057	127.9966	134	0.253502	0.440873	160
hsa04623	Cytosolic DNA-sensing pathway	17814	130	8057	58.79701	63	0.255825	0.442166	162
hsa00232	Caffeine metabolism	17814	16	8057	7.236556	9	0.261857	0.449816	163
hsa00051	Fructose and mannose metabolism	17814	94	8057	42.51476	46	0.266986	0.455588	164
hsa04066	HIF-1 signaling pathway	17814	286	8057	129.3534	135	0.268472	0.455588	164
hsa05131	Shigellosis	17814	165	8057	74.62698	79	0.270938	0.457005	166
hsa05143	African trypanosomiasis	17814	88	8057	39.80106	43	0.280492	0.470286	167
hsa05034	Alcoholism	17814	350	8057	158.2997	164	0.286002	0.476671	168
hsa03050	Proteasome	17814	114	8057	51.56046	55	0.288863	0.478589	169
hsa04650	Natural killer cell mediated cytotoxicity	17814	283	8057	127.9966	133	0.293433	0.483302	170

hsa00650	Butanoate metabolism	17814	63	8057	28.49394	31	0.304611	0.498779	171
hsa02010	ABC transporters	17814	119	8057	53.82188	57	0.309648	0.50151	172
hsa05416	Viral myocarditis	17814	132	8057	59.70158	63	0.310986	0.50151	172
hsa00120	Primary bile acid biosynthesis	17814	44	8057	19.90053	22	0.312754	0.50151	172
hsa05016	Huntington,s	17814	452	8057	204.4327	210	0.313444	0.50151	172
hsa00600	Sphingolipid metabolism	17814	85	8057	38.4442	41	0.325818	0.515532	176
hsa00531	Glycosaminoglycan degradation	17814	40	8057	18.09139	20	0.32589	0.515532	176
hsa00500	Starch and sucrose metabolism	17814	98	8057	44.3239	47	0.328104	0.516118	178
hsa05410	Hypertrophic cardiomyopathy (HCM)	17814	200	8057	90.45694	94	0.331261	0.517116	179
hsa05340	Primary immunodeficiency	17814	96	8057	43.41933	46	0.333537	0.517116	179
hsa05145	Toxoplasmosis	17814	296	8057	133.8763	138	0.334279	0.517116	179
hsa05020	Prion diseases	17814	107	8057	48.39447	51	0.33999	0.523061	182
hsa00062	Fatty acid	17814	51	8057	23.06652	25	0.341978	0.523245	183
hsa04710	Circadian rhythm	17814	64	8057	28.94622	31	0.346835	0.527792	184
hsa04974	Protein digestion and absorption	17814	179	8057	80.95896	84	0.349976	0.529693	185
hsa03020	RNA polymerase	17814	75	8057	33.92135	36	0.355746	0.533426	186
hsa04962	Vasopressin-regulated water reabsorption	17814	114	8057	51.56046	54	0.356253	0.533426	186
hsa00592	alpha-Linolenic acid metabolism	17814	45	8057	20.35281	22	0.364041	0.542189	188
hsa04726	Serotonergic synapse	17814	278	8057	125.7352	129	0.367917	0.545062	189
hsa00532	Glycosaminoglycan biosynthesis - chondroitin sulfate / dermatan sulfate	17814	28	8057	12.66397	14	0.373613	0.546698	190
hsa05142	Chagas disease (American trypanosomiasis)	17814	274	8057	123.926	127	0.375821	0.546698	190
hsa04145	Phagosome	17814	331	8057	149.7062	153	0.377176	0.546698	190
hsa04918	Thyroid hormone synthesis	17814	167	8057	75.53155	78	0.378446	0.546698	190
hsa04713	Circadian entrainment	17814	237	8057	107.1915	110	0.380159	0.546698	190
hsa00330	Arginine and proline metabolism	17814	130	8057	58.79701	61	0.380736	0.546698	190
hsa04340	Hedgehog signaling pathway	17814	117	8057	52.91731	55	0.383065	0.547236	196
hsa00072	Synthesis and degradation of ketone bodies	17814	24	8057	10.85483	12	0.393567	0.559384	197
hsa05010	Alzheimer,s disease	17814	411	8057	185.889	189	0.39621	0.560297	198
hsa04916	Melanogenesis	17814	262	8057	118.4986	121	0.400515	0.563539	199
hsa04122	Sulfur relay system	17814	22	8057	9.950264	11	0.404668	0.566535	200

hsa00512	Mucin type O-Glycan biosynthesis	17814	48	8057	21.70967	23	0.407683	0.567917	201
hsa00785	Lipoic acid metabolism	17814	5	8057	2.261424	3	0.411066	0.568028	202
hsa04621	NOD-like receptor signaling pathway	17814	131	8057	59.2493	61	0.41182	0.568028	202
hsa04020	Calcium signaling pathway	17814	419	8057	189.5073	192	0.420996	0.577838	204
hsa04728	Dopaminergic synapse	17814	329	8057	148.8017	151	0.42407	0.579218	205
hsa05120	Epithelial cell signaling in Helicobacter pylori infection	17814	178	8057	80.50668	82	0.439363	0.597193	206
hsa04971	Gastric acid secretion	17814	187	8057	84.57724	86	0.444924	0.601829	207
hsa00591	Linoleic acid metabolism	17814	51	8057	23.06652	24	0.449725	0.6054	208
hsa04380	Osteoclast differentiation	17814	335	8057	151.5154	153	0.4559	0.609792	209
hsa00604	Glycosphingolipid biosynthesis - ganglio series	17814	38	8057	17.18682	18	0.457344	0.609792	209
hsa05412	Arrhythmogenic right ventricular cardiomyopathy (ARVC)	17814	190	8057	85.9341	87	0.466065	0.618475	211
hsa04913	Ovarian steroidogenesis	17814	133	8057	60.15387	61	0.474792	0.625525	212
hsa04919	Thyroid hormone signaling pathway	17814	336	8057	151.9677	153	0.475846	0.625525	212
hsa04730	Long-term depression	17814	153	8057	69.19956	70	0.479449	0.627317	214
hsa04970	Salivary secretion	17814	207	8057	93.62294	94	0.506014	0.655276	215
hsa05152	Tuberculosis	17814	426	8057	192.6733	193	0.506214	0.655276	215
hsa04932	Non-alcoholic fatty liver disease	17814	373	8057	168.7022	169	0.507839	0.655276	215
hsa00770	Pantothenate and CoA biosynthesis	17814	50	8057	22.61424	23	0.511134	0.656502	218
hsa05323	Rheumatoid arthritis	17814	197	8057	89.10009	89	0.533518	0.682124	219
hsa04973	Carbohydrate digestion and absorption	17814	95	8057	42.96705	43	0.537132	0.683622	220
hsa04724	Glutamatergic synapse	17814	280	8057	126.6397	126	0.554115	0.699077	221
hsa05164	Influenza A	17814	391	8057	176.8433	176	0.554269	0.699077	221
hsa04961	Endocrine and other factor-regulated calcium	17814	127	8057	57.44016	57	0.565675	0.708479	223
hsa04911	Insulin secretion	17814	205	8057	92.71837	92	0.567401	0.708479	223
hsa04976	Bile secretion	17814	165	8057	74.62698	74	0.569314	0.708479	223
hsa04141	Protein processing in endoplasmic reticulum	17814	386	8057	174.5819	173	0.584611	0.724297	226

hsa03008	Ribosome biogenesis in eukaryotes	17814	166	8057	75.07926	74	0.596775	0.735	227
hsa04146	Peroxisome	17814	175	8057	79.14983	78	0.5985	0.735	227
hsa03450	Non-homologous end-joining	17814	36	8057	16.28225	16	0.601257	0.735162	229
hsa00565	Ether lipid metabolism	17814	79	8057	35.73049	35	0.60841	0.740674	230
hsa04964	Proximal tubule bicarbonate reclamation	17814	59	8057	26.6848	26	0.62029	0.751866	231
hsa00100	Steroid biosynthesis	17814	41	8057	18.54367	18	0.626555	0.756187	232
hsa00071	Fatty acid degradation	17814	109	8057	49.29903	48	0.634712	0.761597	233
hsa04210	Apoptosis	17814	228	8057	103.1209	101	0.636477	0.761597	233
hsa00514	Other types of O-glycan biosynthesis	17814	55	8057	24.87566	24	0.643948	0.767258	235
hsa04140	Regulation of autophagy	17814	85	8057	38.4442	37	0.663311	0.786979	236
hsa01040	Biosynthesis of unsaturated fatty acids	17814	42	8057	18.99596	18	0.677119	0.799972	237
hsa04080	Neuroactive ligand-receptor interaction	17814	490	8057	221.6195	217	0.680844	0.800993	238
hsa00780	Biotin metabolism	17814	7	8057	3.165993	3	0.68803	0.805383	239
hsa00430	Taurine and hypotaurine metabolism	17814	19	8057	8.59341	8	0.690328	0.805383	239
hsa00471	D-Glutamine and D-glutamate metabolism	17814	12	8057	5.427417	5	0.701258	0.812228	241
hsa04330	Notch signaling pathway	17814	118	8057	53.3696	51	0.701997	0.812228	241
hsa00511	Other glycan degradation	17814	52	8057	23.51881	22	0.712127	0.820558	243
hsa04130	SNARE interactions in vesicular transport	17814	73	8057	33.01678	31	0.722461	0.826745	244
hsa00790	Folate biosynthesis	17814	36	8057	16.28225	15	0.723402	0.826745	244
hsa04320	Dorso-ventral axis formation	17814	62	8057	28.04165	26	0.741115	0.837897	246
hsa04950	Maturity onset diabetes of the young	17814	69	8057	31.20765	29	0.743288	0.837897	246
hsa04612	Antigen processing and presentation	17814	156	8057	70.55642	67	0.743365	0.837897	246
hsa00524	Butirosin and neomycin biosynthesis	17814	15	8057	6.784271	6	0.74513	0.837897	246
hsa04723	Retrograde endocannabinoid signaling	17814	238	8057	107.6438	103	0.749584	0.839534	250
hsa05140	Leishmaniasis	17814	161	8057	72.81784	69	0.753426	0.840476	251
hsa04966	Collecting duct acid secretion	17814	65	8057	29.39851	27	0.764633	0.847501	252

hsa00534	Glycosaminoglycan biosynthesis - heparan sulfate / heparin	17814	44	8057	19.90053	18	0.765778	0.847501	252
hsa05321	Inflammatory bowel disease (IBD)	17814	146	8057	66.03357	62	0.775068	0.854406	254
hsa05032	Morphine addiction	17814	207	8057	93.62294	88	0.804941	0.883856	255
hsa04620	Toll-like receptor signaling pathway	17814	244	8057	110.3575	104	0.812636	0.888119	256
hsa05031	Amphetamine addiction	17814	187	8057	84.57724	79	0.815167	0.888119	256
hsa05012	Parkinson,s disease	17814	322	8057	145.6357	138	0.820944	0.889084	258
hsa05332	Graft-versus-host disease	17814	81	8057	36.63506	33	0.822403	0.889084	258
hsa04672	Intestinal immune network for IgA production	17814	96	8057	43.41933	39	0.844131	0.905581	260
hsa05150	Staphylococcus aureus infection	17814	96	8057	43.41933	39	0.844131	0.905581	260
hsa04742	Taste transduction	17814	68	8057	30.75536	27	0.850642	0.909083	262
hsa04640	Hematopoietic cell lineage	17814	195	8057	88.19552	81	0.86736	0.923425	263
hsa00920	Sulfur metabolism	17814	25	8057	11.30712	9	0.871074	0.923867	264
hsa04721	Synaptic vesicle	17814	152	8057	68.74728	62	0.882474	0.932425	265
hsa04610	Complement and coagulation cascades	17814	150	8057	67.84271	61	0.887087	0.933776	266
hsa05030	Cocaine addiction	17814	139	8057	62.86758	56	0.89662	0.940275	267
hsa04727	GABAergic synapse	17814	204	8057	92.26608	83	0.916861	0.957915	268
hsa00061	Fatty acid biosynthesis	17814	19	8057	8.59341	6	0.925246	0.963081	269
hsa00460	Cyanoamino acid metabolism	17814	14	8057	6.331986	4	0.938941	0.973717	270
hsa05320	Autoimmune thyroid disease	17814	93	8057	42.06248	35	0.943731	0.975072	271
hsa00190	Oxidative phosphorylation	17814	272	8057	123.0214	108	0.972065	0.998163	272
hsa00730	Thiamine metabolism	17814	7	8057	3.165993	1	0.985227	0.998163	272
hsa03022	Basal transcription factors	17814	103	8057	46.58533	36	0.986803	0.998163	272
hsa05310	Asthma	17814	73	8057	33.01678	24	0.988364	0.998163	272
hsa05330	Allograft rejection	17814	81	8057	36.63506	27	0.989076	0.998163	272
hsa04940	Type I diabetes mellitus	17814	92	8057	41.61019	31	0.990821	0.998163	272
hsa04740	Olfactory transduction	17814	138	8057	62.41529	49	0.992007	0.998163	272
hsa05033	Nicotine addiction	17814	97	8057	43.87162	31	0.997224	0.998163	272
hsa04744	Phototransduction	17814	80	8057	36.18278	24	0.998163	0.998163	272

All results in BRCA dataset by ORA

Pathway ID	Pathway Name	Universe.Size	Gene.Set.Size	Total.Hits	Expected.Hits	Observed.Hits	Pvalue	Adjusted.Pvalue	Rank

hsa04110	Cell cycle	17814	114	8057	51.56046	74	1.72E-05	0.004196	1
hsa05206	MicroRNAs in	17814	143	8057	64.67671	89	2.99E-05	0.004196	1
hsa05034	Alcoholism	17814	164	8057	74.17469	97	0.000223	0.020917	3
hsa04510	Focal adhesion	17814	198	8057	89.55237	114	0.000302	0.021221	4
hsa04390	Hippo signaling pathway	17814	146	8057	66.03357	86	0.000592	0.033266	5
hsa05322	Systemic lupus erythematosus	17814	113	8057	51.10817	68	0.000961	0.045026	6
hsa05200	Pathways in cancer	17814	318	8057	143.8265	170	0.0018	0.072255	7
hsa04120	Ubiquitin mediated proteolysis	17814	126	8057	56.98787	73	0.002721	0.090582	8
hsa04150	mTOR signaling pathway	17814	54	8057	24.42337	35	0.002901	0.090582	8
hsa04360	Axon guidance	17814	125	8057	56.53559	72	0.00355	0.099764	10
hsa03030	DNA replication	17814	35	8057	15.82997	24	0.004497	0.114869	11
hsa00510	N-Glycan biosynthesis	17814	46	8057	20.8051	30	0.004956	0.116046	12
hsa04810	Regulation of actin cytoskeleton	17814	203	8057	91.8138	110	0.006173	0.124388	13
hsa00563	Glycosylphosphatidylinositol(GPI)-anchor biosynthesis	17814	25	8057	11.30712	18	0.006197	0.124388	13
hsa03460	Fanconi anemia pathway	17814	43	8057	19.44824	28	0.006763	0.1267	15
hsa04512	ECM-receptor interaction	17814	85	8057	38.4442	50	0.00798	0.140149	16
hsa05203	Viral carcinogenesis	17814	189	8057	85.48181	102	0.009443	0.151603	17
hsa04530	Tight junction	17814	123	8057	55.63102	69	0.009805	0.151603	17
hsa05222	Small cell lung cancer	17814	84	8057	37.99192	49	0.010615	0.151603	17
hsa04666	Fc gamma R-mediated phagocytosis	17814	86	8057	38.89649	50	0.01079	0.151603	17
hsa00280	Valine, leucine and isoleucine degradation	17814	43	8057	19.44824	27	0.015359	0.205521	21
hsa05130	Pathogenic Escherichia coli infection	17814	51	8057	23.06652	31	0.018275	0.224088	22
hsa05219	Bladder cancer	17814	36	8057	16.28225	23	0.01865	0.224088	22
hsa00360	Phenylalanine metabolism	17814	18	8057	8.141125	13	0.019139	0.224088	22
hsa00071	Fatty acid degradation	17814	40	8057	18.09139	25	0.020897	0.228485	25
hsa00533	Glycosaminoglycan biosynthesis - keratan sulfate	17814	13	8057	5.879701	10	0.021141	0.228485	25
hsa00600	Sphingolipid metabolism	17814	35	8057	15.82997	22	0.027101	0.272225	27
hsa05221	Acute myeloid leukemia	17814	56	8057	25.32794	33	0.027126	0.272225	27
hsa05161	Hepatitis B	17814	138	8057	62.41529	74	0.028771	0.278437	29
hsa00350	Tyrosine metabolism	17814	39	8057	17.6391	24	0.029726	0.278437	29
hsa05166	HTLV-I infection	17814	250	8057	113.0712	128	0.032651	0.295965	31

hsa05202	Transcriptional misregulation in cancer	17814	168	8057	75.98383	88	0.036712	0.300214	32
hsa04370	VEGF signaling pathway	17814	59	8057	26.6848	34	0.037371	0.300214	32
hsa04270	Vascular smooth muscle contraction	17814	113	8057	51.10817	61	0.037772	0.300214	32
hsa04520	Adherens junction	17814	73	8057	33.01678	41	0.039228	0.300214	32
hsa05215	Prostate cancer	17814	87	8057	39.34877	48	0.039501	0.300214	32
hsa04064	NF-kappa B signaling pathway	17814	81	8057	36.63506	45	0.03953	0.300214	32
hsa00620	Pyruvate	17814	40	8057	18.09139	24	0.042971	0.317757	38
hsa05205	Proteoglycans in cancer	17814	215	8057	97.24121	110	0.045781	0.319667	39
hsa03440	Homologous recombination	17814	25	8057	11.30712	16	0.046049	0.319667	39
hsa04114	Oocyte meiosis	17814	106	8057	47.94218	57	0.047277	0.319667	39
hsa05210	Colorectal cancer	17814	62	8057	28.04165	35	0.049711	0.319667	39
hsa04914	Progesterone-mediated oocyte maturation	17814	84	8057	37.99192	46	0.049815	0.319667	39
hsa05212	Pancreatic cancer	17814	66	8057	29.85079	37	0.050055	0.319667	39
hsa04611	Platelet activation	17814	121	8057	54.72645	64	0.054228	0.338626	45
hsa00270	Cysteine and methionine metabolism	17814	35	8057	15.82997	21	0.056479	0.344244	46
hsa04015	Rap1 signaling pathway	17814	200	8057	90.45694	102	0.057578	0.344244	46
hsa04012	ErbB signaling pathway	17814	85	8057	38.4442	46	0.061944	0.362629	48
hsa04115	p53 signaling pathway	17814	63	8057	28.49394	35	0.064207	0.36821	49
hsa00640	Propanoate metabolism	17814	30	8057	13.56854	18	0.074749	0.420091	50
hsa02010	ABC transporters	17814	44	8057	19.90053	25	0.081839	0.450917	51
hsa04144	Endocytosis	17814	176	8057	79.60211	89	0.088056	0.468975	52
hsa04022	cGMP-PKG signaling pathway	17814	160	8057	72.36556	81	0.097372	0.468975	52
hsa00230	Purine metabolism	17814	154	8057	69.65185	78	0.101152	0.468975	52
hsa04062	Chemokine signaling pathway	17814	175	8057	79.14983	88	0.10144	0.468975	52
hsa00010	Glycolysis / Gluconeogenesis	17814	63	8057	28.49394	34	0.102396	0.468975	52
hsa05214	Glioma	17814	63	8057	28.49394	34	0.102396	0.468975	52
hsa05217	Basal cell carcinoma	17814	55	8057	24.87566	30	0.105035	0.468975	52
hsa00330	Arginine and proline metabolism	17814	53	8057	23.97109	29	0.105579	0.468975	52
hsa05134	Legionellosis	17814	53	8057	23.97109	29	0.105579	0.468975	52
hsa03410	Base excision repair	17814	33	8057	14.9254	19	0.105653	0.468975	52
hsa04978	Mineral absorption	17814	49	8057	22.16195	27	0.106476	0.468975	52
hsa03420	Nucleotide excision repair	17814	43	8057	19.44824	24	0.107194	0.468975	52
hsa00310	Lysine degradation	17814	41	8057	18.54367	23	0.107212	0.468975	52

hsa04151	PI3K-Akt signaling pathway	17814	322	8057	145.6357	157	0.109935	0.468975	52
hsa04152	AMPK signaling pathway	17814	117	8057	52.91731	60	0.110151	0.468975	52
hsa04310	Wnt signaling pathway	17814	136	8057	61.51072	69	0.113549	0.476226	67
hsa05100	Bacterial invasion of epithelial cells	17814	74	8057	33.46907	39	0.119683	0.48899	68
hsa04917	Prolactin signaling pathway	17814	72	8057	32.5645	38	0.120974	0.48899	68
hsa05218	Melanoma	17814	70	8057	31.65993	37	0.122264	0.48899	68
hsa03320	PPAR signaling pathway	17814	68	8057	30.75536	36	0.123553	0.48899	68
hsa05131	Shigellosis	17814	60	8057	27.13708	32	0.128636	0.495336	72
hsa05213	Endometrial cancer	17814	52	8057	23.51881	28	0.133444	0.495336	72
hsa00400	Phenylalanine, tyrosine and tryptophan biosynthesis	17814	5	8057	2.261424	4	0.133489	0.495336	72
hsa04540	Gap junction	17814	81	8057	36.63506	42	0.138276	0.495336	72
hsa00601	Glycosphingolipid biosynthesis - lacto and neolacto series	17814	24	8057	10.85483	14	0.138963	0.495336	72
hsa00260	Glycine, serine and threonine metabolism	17814	38	8057	17.18682	21	0.139952	0.495336	72
hsa00030	Pentose phosphate pathway	17814	26	8057	11.7594	15	0.140042	0.495336	72
hsa00250	Alanine, aspartate and glutamate metabolism	17814	34	8057	15.37768	19	0.140872	0.495336	72
hsa00051	Fructose and mannose metabolism	17814	30	8057	13.56854	17	0.141021	0.495336	72
hsa04141	Protein processing in endoplasmic reticulum	17814	153	8057	69.19956	76	0.152043	0.522621	81
hsa04720	Long-term potentiation	17814	65	8057	29.39851	34	0.152948	0.522621	81
hsa00240	Pyrimidine metabolism	17814	90	8057	40.70562	46	0.154369	0.522621	81
hsa05223	Non-small cell lung cancer	17814	55	8057	24.87566	29	0.162678	0.544195	84
hsa00520	Amino sugar and nucleotide sugar metabolism	17814	47	8057	21.25738	25	0.170587	0.561115	85
hsa04662	B cell receptor signaling pathway	17814	70	8057	31.65993	36	0.177643	0.561115	85
hsa00380	Tryptophan metabolism	17814	35	8057	15.82997	19	0.181879	0.561115	85
hsa04068	FoxO signaling pathway	17814	127	8057	57.44016	63	0.182538	0.561115	85

hsa00532	Glycosaminoglycan biosynthesis - chondroitin sulfate / dermatan sulfate	17814	15	8057	6.784271	9	0.186488	0.561115	85
hsa00020	Citrate cycle (TCA cycle)	17814	29	8057	13.11626	16	0.186529	0.561115	85
hsa05216	Thyroid cancer	17814	29	8057	13.11626	16	0.186529	0.561115	85
hsa00910	Nitrogen metabolism	17814	17	8057	7.68884	10	0.188456	0.561115	85
hsa04614	Renin-angiotensin system	17814	17	8057	7.68884	10	0.188456	0.561115	85
hsa03430	Mismatch repair	17814	23	8057	10.40255	13	0.189427	0.561115	85
hsa00630	Glyoxylate and dicarboxylate metabolism	17814	21	8057	9.497979	12	0.189701	0.561115	85
hsa04350	TGF-beta signaling pathway	17814	77	8057	34.82592	39	0.199374	0.574779	96
hsa04915	Estrogen signaling pathway	17814	96	8057	43.41933	48	0.200511	0.574779	96
hsa00564	Glycerophospholipid metabolism	17814	75	8057	33.92135	38	0.202464	0.574779	96
hsa00300	Lysine biosynthesis	17814	2	8057	0.904569	2	0.204548	0.574779	96
hsa00785	Lipoic acid metabolism	17814	2	8057	0.904569	2	0.204548	0.574779	96
hsa03010	Ribosome	17814	126	8057	56.98787	62	0.208599	0.580358	101
hsa04920	Adipocytokine signaling pathway	17814	69	8057	31.20765	35	0.212163	0.583987	102
hsa05144	Malaria	17814	44	8057	19.90053	23	0.214881	0.583987	102
hsa00970	Aminoacyl-tRNA biosynthesis	17814	42	8057	18.99596	22	0.218117	0.583987	102
hsa05146	Amoebiasis	17814	103	8057	46.58533	51	0.218216	0.583987	102
hsa04070	Phosphatidylinositol signaling system	17814	76	8057	34.37364	38	0.234704	0.616985	106
hsa04921	Oxytocin signaling pathway	17814	153	8057	69.19956	74	0.241135	0.616985	106
hsa03013	RNA transport	17814	138	8057	62.41529	67	0.241185	0.616985	106
hsa05220	Chronic myeloid leukemia	17814	72	8057	32.5645	36	0.242585	0.616985	106
hsa00290	Valine, leucine and isoleucine biosynthesis	17814	4	8057	1.809139	3	0.24452	0.616985	106
hsa00340	Histidine	17814	26	8057	11.7594	14	0.245572	0.616985	106
hsa05110	Vibrio cholerae infection	17814	51	8057	23.06652	26	0.245916	0.616985	106
hsa05211	Renal cell carcinoma	17814	66	8057	29.85079	33	0.255216	0.630615	113
hsa00900	Terpenoid backbone biosynthesis	17814	20	8057	9.045694	11	0.255837	0.630615	113
hsa00750	Vitamin B6 metabolism	17814	6	8057	2.713708	4	0.259021	0.632913	115
hsa05132	Salmonella infection	17814	79	8057	35.73049	39	0.264604	0.640981	116
hsa04668	TNF signaling pathway	17814	105	8057	47.4899	51	0.276368	0.663755	117
hsa00565	Ether lipid metabolism	17814	35	8057	15.82997	18	0.284179	0.67673	118

hsa00561	Glycerolipid metabolism	17814	50	8057	22.61424	25	0.294889	0.696334	119
hsa00052	Galactose metabolism	17814	29	8057	13.11626	15	0.301546	0.700286	120
hsa00410	beta-Alanine metabolism	17814	29	8057	13.11626	15	0.301546	0.700286	120
hsa03015	mRNA surveillance pathway	17814	78	8057	35.27821	38	0.305433	0.703498	122
hsa00480	Glutathione metabolism	17814	44	8057	19.90053	22	0.312754	0.713668	123
hsa04912	GnRH signaling pathway	17814	89	8057	40.25334	43	0.314928	0.713668	123
hsa04066	HIF-1 signaling pathway	17814	102	8057	46.13304	49	0.317615	0.713998	125
hsa03040	Spliceosome	17814	100	8057	45.22847	48	0.322798	0.71989	126
hsa04916	Melanogenesis	17814	98	8057	44.3239	47	0.328104	0.724675	127
hsa05160	Hepatitis C	17814	124	8057	56.08331	59	0.330101	0.724675	127
hsa05133	Pertussis	17814	68	8057	30.75536	33	0.334095	0.727712	129
hsa04910	Insulin signaling pathway	17814	133	8057	60.15387	63	0.340044	0.727712	129
hsa04960	Aldosterone-regulated sodium reabsorption	17814	36	8057	16.28225	18	0.340222	0.727712	129
hsa00830	Retinol metabolism	17814	51	8057	23.06652	25	0.341978	0.727712	129
hsa00100	Steroid biosynthesis	17814	17	8057	7.68884	9	0.344433	0.727712	129
hsa03050	Proteasome	17814	43	8057	19.44824	21	0.372037	0.774844	134
hsa04014	Ras signaling pathway	17814	217	8057	98.14578	101	0.37264	0.774844	134
hsa04730	Long-term depression	17814	56	8057	25.32794	27	0.375013	0.774844	134
hsa04670	Leukocyte transendothelial migration	17814	106	8057	47.94218	50	0.379233	0.777842	137
hsa04320	Dorso-ventral axis formation	17814	24	8057	10.85483	12	0.393567	0.801393	138
hsa05140	Leishmaniasis	17814	63	8057	28.49394	30	0.397985	0.804559	139
hsa04977	Vitamin digestion and absorption	17814	22	8057	9.950264	11	0.404668	0.812226	140
hsa05340	Primary immunodeficiency	17814	35	8057	15.82997	17	0.408109	0.813324	141
hsa04918	Thyroid hormone synthesis	17814	68	8057	30.75536	32	0.426494	0.831449	142
hsa00670	One carbon pool by folate	17814	18	8057	8.141125	9	0.429875	0.831449	142
hsa05416	Viral myocarditis	17814	55	8057	24.87566	26	0.431188	0.831449	142
hsa05414	Dilated cardiomyopathy	17814	90	8057	40.70562	42	0.431808	0.831449	142
hsa05031	Amphetamine addiction	17814	66	8057	29.85079	31	0.434673	0.831449	142
hsa04972	Pancreatic secretion	17814	88	8057	39.80106	41	0.4391	0.831449	142
hsa03020	RNA polymerase	17814	29	8057	13.11626	14	0.440875	0.831449	142
hsa05143	African trypanosomiasis	17814	29	8057	13.11626	14	0.440875	0.831449	142
hsa04142	Lysosome	17814	119	8057	53.82188	55	0.449037	0.836906	150

hsa05014	Amyotrophic lateral sclerosis (ALS)	17814	51	8057	23.06652	24	0.449725	0.836906	150
hsa04722	Neurotrophin signaling pathway	17814	117	8057	52.91731	54	0.455644	0.842341	152
hsa04713	Circadian entrainment	17814	93	8057	42.06248	43	0.462322	0.849101	153
hsa05169	Epstein-Barr virus infection	17814	190	8057	85.9341	87	0.466065	0.850418	154
hsa04623	Cytosolic DNA-sensing pathway	17814	56	8057	25.32794	26	0.479873	0.854182	155
hsa00650	Butanoate metabolism	17814	23	8057	10.40255	11	0.481073	0.854182	155
hsa04964	Proximal tubule bicarbonate reclamation	17814	23	8057	10.40255	11	0.481073	0.854182	155
hsa04725	Cholinergic synapse	17814	109	8057	49.29903	50	0.483327	0.854182	155
hsa05145	Toxoplasmosis	17814	109	8057	49.29903	50	0.483327	0.854182	155
hsa00130	Ubiquinone and other terpenoid-quinone	17814	10	8057	4.522847	5	0.50154	0.875255	160
hsa05410	Hypertrophic cardiomyopathy (HCM)	17814	83	8057	37.53963	38	0.502094	0.875255	160
hsa04146	Peroxisome	17814	72	8057	32.5645	33	0.504596	0.875255	160
hsa04971	Gastric acid secretion	17814	70	8057	31.65993	32	0.513808	0.884377	163
hsa04145	Phagosome	17814	139	8057	62.86758	63	0.523991	0.884377	163
hsa00562	Inositol phosphate metabolism	17814	57	8057	25.78023	26	0.528047	0.884377	163
hsa00072	Synthesis and degradation of ketone bodies	17814	8	8057	3.618278	4	0.528385	0.884377	163
hsa04122	Sulfur relay system	17814	8	8057	3.618278	4	0.528385	0.884377	163
hsa00983	Drug metabolism - other enzymes	17814	37	8057	16.73453	17	0.528738	0.884377	163
hsa00120	Primary bile acid biosynthesis	17814	17	8057	7.68884	8	0.533353	0.886818	169
hsa04010	MAPK signaling pathway	17814	244	8057	110.3575	110	0.543413	0.898229	170
hsa00980	Metabolism of xenobiotics by cytochrome P450	17814	62	8057	28.04165	28	0.553407	0.9094	171
hsa04060	Cytokine-cytokine receptor interaction	17814	238	8057	107.6438	107	0.558799	0.912921	172
hsa04130	SNARE interactions in vesicular transport	17814	31	8057	14.02083	14	0.572203	0.921038	173
hsa00514	Other types of O-glycan biosynthesis	17814	22	8057	9.950264	10	0.5736	0.921038	173
hsa03060	Protein export	17814	22	8057	9.950264	10	0.5736	0.921038	173
hsa04660	T cell receptor signaling pathway	17814	103	8057	46.58533	46	0.584074	0.923015	176
hsa00140	Steroid hormone biosynthesis	17814	47	8057	21.25738	21	0.586055	0.923015	176

hsa05120	Epithelial cell signaling in Helicobacter pylori infection	17814	65	8057	29.39851	29	0.58717	0.923015	176
hsa00760	Nicotinate and nicotinamide metabolism	17814	20	8057	9.045694	9	0.593822	0.923015	176
hsa04750	Inflammatory mediator regulation of TRP channels	17814	90	8057	40.70562	40	0.599717	0.923015	176
hsa04261	Adrenergic signaling in cardiomyocytes	17814	146	8057	66.03357	65	0.600046	0.923015	176
hsa05010	Alzheimer,s disease	17814	155	8057	70.10413	69	0.601607	0.923015	176
hsa04380	Osteoclast differentiation	17814	126	8057	56.98787	56	0.604292	0.923015	176
hsa04710	Circadian rhythm	17814	27	8057	12.21169	12	0.60595	0.923015	176
hsa04621	NOD-like receptor signaling pathway	17814	52	8057	23.51881	23	0.610164	0.923015	176
hsa04020	Calcium signaling pathway	17814	171	8057	77.34069	76	0.610964	0.923015	176
hsa00040	Pentose and glucuronate interconversions	17814	25	8057	11.30712	11	0.62464	0.933637	187
hsa00512	Mucin type O-Glycan biosynthesis	17814	25	8057	11.30712	11	0.62464	0.933637	187
hsa03018	RNA degradation	17814	66	8057	29.85079	29	0.629602	0.934625	189
hsa00982	Drug metabolism - cytochrome P450	17814	57	8057	25.78023	25	0.631953	0.934625	189
hsa00450	Selenocompound metabolism	17814	16	8057	7.236556	7	0.641009	0.937079	191
hsa04728	Dopaminergic synapse	17814	125	8057	56.53559	55	0.64223	0.937079	191
hsa04723	Retrograde endocannabinoid signaling	17814	98	8057	44.3239	43	0.643617	0.937079	191
hsa04974	Protein digestion and absorption	17814	78	8057	35.27821	34	0.656167	0.948821	194
hsa04962	Vasopressin-regulated water reabsorption	17814	44	8057	19.90053	19	0.662779	0.948821	194
hsa04340	Hedgehog signaling pathway	17814	51	8057	23.06652	22	0.668989	0.948821	194
hsa00790	Folate biosynthesis	17814	14	8057	6.331986	6	0.669084	0.948821	194
hsa04664	Fc epsilon RI signaling pathway	17814	67	8057	30.30308	29	0.669964	0.948821	194
hsa04672	Intestinal immune network for IgA production	17814	42	8057	18.99596	18	0.677119	0.948821	194
hsa04919	Thyroid hormone signaling pathway	17814	115	8057	52.01274	50	0.680726	0.948821	194
hsa05323	Rheumatoid arthritis	17814	81	8057	36.63506	35	0.68246	0.948821	194
hsa04514	Cell adhesion molecules (CAMs)	17814	131	8057	59.2493	57	0.685111	0.948821	194
hsa00062	Fatty acid	17814	19	8057	8.59341	8	0.690328	0.948821	194

hsa00531	Glycosaminoglycan degradation	17814	19	8057	8.59341	8	0.690328	0.948821	194
hsa03008	Ribosome biogenesis in eukaryotes	17814	63	8057	28.49394	27	0.692201	0.948821	194
hsa04970	Salivary secretion	17814	84	8057	37.99192	36	0.707032	0.958338	206
hsa04930	Type II diabetes mellitus	17814	45	8057	20.35281	19	0.709375	0.958338	206
hsa04961	Endocrine and other factor-regulated calcium	17814	45	8057	20.35281	19	0.709375	0.958338	206
hsa00770	Pantothenate and CoA biosynthesis	17814	17	8057	7.68884	7	0.716368	0.961852	209
hsa04610	Complement and coagulation cascades	17814	66	8057	29.85079	28	0.718821	0.961852	209
hsa04724	Glutamatergic synapse	17814	112	8057	50.65589	48	0.725354	0.965993	211
hsa00604	Glycosphingolipid biosynthesis - ganglio series	17814	15	8057	6.784271	6	0.74513	0.973671	212
hsa00232	Caffeine metabolism	17814	5	8057	2.261424	2	0.747225	0.973671	212
hsa00460	Cyanoamino acid metabolism	17814	5	8057	2.261424	2	0.747225	0.973671	212
hsa00524	Butirosin and neomycin biosynthesis	17814	5	8057	2.261424	2	0.747225	0.973671	212
hsa04210	Apoptosis	17814	83	8057	37.53963	35	0.748445	0.973671	212
hsa04973	Carbohydrate digestion and absorption	17814	39	8057	17.6391	16	0.753452	0.973681	217
hsa04630	Jak-STAT signaling pathway	17814	143	8057	64.67671	61	0.758985	0.973681	217
hsa05016	Huntington,s	17814	166	8057	75.07926	71	0.76303	0.973681	217
hsa05204	Chemical carcinogenesis	17814	65	8057	29.39851	27	0.764633	0.973681	217
hsa00500	Starch and sucrose metabolism	17814	44	8057	19.90053	18	0.765778	0.973681	217
hsa03450	Non-homologous end-joining	17814	13	8057	5.879701	5	0.777155	0.98265	222
hsa04612	Antigen processing and presentation	17814	68	8057	30.75536	28	0.786072	0.98265	222
hsa04976	Bile secretion	17814	68	8057	30.75536	28	0.786072	0.98265	222
hsa05168	Herpes simplex infection	17814	158	8057	71.46099	67	0.786819	0.98265	222
hsa03022	Basal transcription factors	17814	40	8057	18.09139	16	0.79443	0.983351	226
hsa05162	Measles	17814	129	8057	58.34473	54	0.804903	0.983351	226
hsa05142	Chagas disease (American trypanosomiasis)	17814	99	8057	44.77619	41	0.806454	0.983351	226
hsa04913	Ovarian steroidogenesis	17814	50	8057	22.61424	20	0.811863	0.983351	226
hsa05032	Morphine addiction	17814	90	8057	40.70562	37	0.813827	0.983351	226

hsa05412	Arrhythmogenic right ventricular cardiomyopathy (ARVC)	17814	74	8057	33.46907	30	0.82337	0.983351	226
hsa05020	Prion diseases	17814	36	8057	16.28225	14	0.824213	0.983351	226
hsa00053	Ascorbate and aldarate metabolism	17814	19	8057	8.59341	7	0.832551	0.983351	226
hsa00730	Thiamine metabolism	17814	3	8057	1.356854	1	0.835713	0.983351	226
hsa00780	Biotin metabolism	17814	3	8057	1.356854	1	0.835713	0.983351	226
hsa00603	Glycosphingolipid biosynthesis - globo series	17814	14	8057	6.331986	5	0.837194	0.983351	226
hsa04260	Cardiac muscle contraction	17814	70	8057	31.65993	28	0.841572	0.983351	226
hsa04726	Serotonergic synapse	17814	110	8057	49.75132	45	0.843553	0.983351	226
hsa04975	Fat digestion and absorption	17814	39	8057	17.6391	15	0.844024	0.983351	226
hsa04620	Toll-like receptor signaling pathway	17814	96	8057	43.41933	39	0.844131	0.983351	226
hsa04622	RIG-I-like receptor signaling pathway	17814	63	8057	28.49394	25	0.844447	0.983351	226
hsa04727	GABAergic synapse	17814	87	8057	39.34877	35	0.852551	0.983351	226
hsa00592	alpha-Linolenic acid metabolism	17814	22	8057	9.950264	8	0.853403	0.983351	226
hsa05030	Cocaine addiction	17814	49	8057	22.16195	19	0.85387	0.983351	226
hsa05164	Influenza A	17814	158	8057	71.46099	65	0.868301	0.990335	245
hsa00740	Riboflavin metabolism	17814	12	8057	5.427417	4	0.869035	0.990335	245
hsa04140	Regulation of autophagy	17814	35	8057	15.82997	13	0.871658	0.990335	245
hsa04330	Notch signaling pathway	17814	45	8057	20.35281	17	0.876513	0.990335	245
hsa05321	Inflammatory bowel disease (IBD)	17814	62	8057	28.04165	24	0.877556	0.990335	245
hsa00590	Arachidonic acid metabolism	17814	55	8057	24.87566	21	0.882937	0.992421	250
hsa04911	Insulin secretion	17814	82	8057	37.08735	32	0.893469	1	251
hsa00591	Linoleic acid metabolism	17814	26	8057	11.7594	9	0.901844	1	251
hsa00920	Sulfur metabolism	17814	10	8057	4.522847	3	0.903015	1	251
hsa05150	Staphylococcus aureus infection	17814	44	8057	19.90053	16	0.909942	1	251
hsa00471	D-Glutamine and D-glutamate metabolism	17814	4	8057	1.809139	1	0.91003	1	251
hsa00534	Glycosaminoglycan biosynthesis - heparan sulfate / heparin	17814	24	8057	10.85483	8	0.917317	1	251
hsa00860	Porphyrin and chlorophyll metabolism	17814	35	8057	15.82997	12	0.930922	1	251

hsa04721	Synaptic vesicle	17814	63	8057	28.49394	23	0.936748	1	251
hsa00511	Other glycan degradation	17814	17	8057	7.68884	5	0.942698	1	251
hsa04932	Non-alcoholic fatty liver disease	17814	141	8057	63.77215	55	0.943005	1	251
hsa05152	Tuberculosis	17814	168	8057	75.98383	66	0.94932	1	251
hsa04640	Hematopoietic cell lineage	17814	79	8057	35.73049	29	0.950245	1	251
hsa04742	Taste transduction	17814	44	8057	19.90053	15	0.950695	1	251
hsa04966	Collecting duct acid secretion	17814	27	8057	12.21169	8	0.968025	1	251
hsa05033	Nicotine addiction	17814	38	8057	17.18682	12	0.9699	1	251
hsa00061	Fatty acid biosynthesis	17814	6	8057	2.713708	1	0.973021	1	251
hsa05012	Parkinson,s disease	17814	122	8057	55.17874	44	0.984119	1	251
hsa00430	Taurine and hypotaurine metabolism	17814	7	8057	3.165993	1	0.985227	1	251
hsa05310	Asthma	17814	27	8057	12.21169	7	0.988273	1	251
hsa04650	Natural killer cell mediated cytotoxicity	17814	124	8057	56.08331	43	0.993482	1	251
hsa00190	Oxidative phosphorylation	17814	115	8057	52.01274	39	0.99487	1	251
hsa01040	Biosynthesis of unsaturated fatty acids	17814	17	8057	7.68884	3	0.996141	1	251
hsa04744	Phototransduction	17814	27	8057	12.21169	6	0.996436	1	251
hsa04950	Maturity onset diabetes of the young	17814	24	8057	10.85483	5	0.996676	1	251
hsa05332	Graft-versus-host disease	17814	37	8057	16.73453	9	0.99748	1	251
hsa05330	Allograft rejection	17814	34	8057	15.37768	8	0.997481	1	251
hsa05320	Autoimmune thyroid disease	17814	47	8057	21.25738	12	0.998363	1	251
hsa04940	Type I diabetes mellitus	17814	40	8057	18.09139	9	0.999207	1	251
hsa04080	Neuroactive ligand-receptor interaction	17814	251	8057	113.5235	88	0.999604	1	251
hsa00472	D-Arginine and D-ornithine metabolism	17814	1	8057	0.452285	0	1	1	251
hsa04740	Olfactory transduction	17814	303	8057	137.0423	42	1	1	251
