# Supplementary material for: A network-based pathway-extending approach using DNA methylation and gene expression data to identify altered pathways
Source: Sci Rep. 2019 Aug 14;9:11853. doi: 10.1038/s41598-019-48372-1 (PMC6694157; doi:10.1038/s41598-019-48372-1)
Supplement: Supplementary file 2 — Supplementary Table S2 [file 41598_2019_48372_MOESM2_ESM.pdf]

A network-based pathway-extending approach using DNA methylation and gene expression data to identify altered pathways

Jie Li[1], Qiaosheng Zhang[1,2,*], Zhuo Chen[1], Dechen Xu[1], and Yadong Wang[1]

[1]Harbin Institute of Technology, School of Computer Science and Technology, Harbin, 150001, P.R. China

[2]Heilongjiang Bayi Agricultural University, College of Science, Daqing, 163319, P.R. China

[*]zqs@hit.edu.cn

All results in BRCA dataset by EP-GSEA

Pathway ID	Pathway Name	SIZE	ES	NES	NOM p-va	FDR q-val	Rank
has03440	Homologous recombination	64	-0.56353	-1.82585	0	0.172521	1
has00980	Metabolism of xenobiotics by cytochrome P450	123	0.431441	1.724909	0.006073	0.216469	2
has00982	Drug metabolism - cytochrome P450	117	0.424514	1.661364	0.018182	0.225122	3
has04964	Proximal tubule bicarbonate reclamation	59	0.405729	1.628851	0.004	0.250462	4
has04270	Vascular smooth muscle contraction	288	0.364617	1.600132	0.005894	0.272699	5
has00500	Starch and sucrose metabolism	98	0.392904	1.662551	0.004098	0.278454	6
has00300	Lysine biosynthesis	5	0.832002	1.565128	0.014403	0.283687	7
has04022	cGMP-PKG signaling pathway	399	0.34506	1.529012	0.009709	0.286532	8
has05204	Chemical carcinogenesis	128	0.393862	1.576787	0.020661	0.291091	9
has04913	Ovarian steroidogenesis	133	0.365315	1.532847	0.023622	0.300979	10
has04960	Aldosterone-regulated sodium reabsorption	95	0.423841	1.734255	0.003929	0.301026	11
has00140	Steroid hormone biosynthesis	86	0.395807	1.540791	0.025743	0.311832	12
has04015	Rap1 signaling pathway	508	0.255052	1.179715	0.190207	0.346539	13
has04068	FoxO signaling pathway	373	0.25781	1.210147	0.146435	0.347587	14
has00350	Tyrosine metabolism	93	0.286826	1.212547	0.185111	0.347827	15
has05412	Arrhythmogenic right ventricular cardiomyopathy	190	0.27857	1.22047	0.157588	0.34852	16
has00630	Glyoxylate and dicarboxylate metabolism	61	0.325577	1.214265	0.210843	0.349614	17
has04916	Melanogenesis	262	0.260667	1.179951	0.192164	0.350166	18
has04910	Insulin signaling pathway	359	0.267134	1.181772	0.196078	0.351563	19
has05223	Non-small cell lung cancer	159	0.274694	1.186584	0.188785	0.351849	20
has00590	Arachidonic acid metabolism	125	0.365039	1.489975	0.041502	0.352399	21
has00531	Glycosaminoglycan degradation	40	0.352518	1.215561	0.217391	0.352556	22
has05414	Dilated cardiomyopathy	218	0.298151	1.22089	0.21875	0.352735	23
has04310	Wnt signaling pathway	334	0.255354	1.223631	0.143713	0.353383	24
has04976	Bile secretion	165	0.316056	1.449999	0.028902	0.353916	25
has04260	Cardiac muscle contraction	179	0.269568	1.203288	0.166667	0.354452	26
has05205	Proteoglycans in cancer	564	0.257612	1.187358	0.189922	0.354798	27
has04975	Fat digestion and absorption	90	0.295689	1.18246	0.220472	0.354833	28
has04977	Vitamin digestion and absorption	53	0.290109	1.197786	0.177189	0.354963	29
has05215	Prostate cancer	256	0.250831	1.227186	0.106542	0.356894	30
has00640	Propanoate metabolism	88	0.298332	1.191113	0.235669	0.357054	31
has04930	Type II diabetes mellitus	132	0.294284	1.229645	0.159393	0.357683	32
has00020	Citrate cycle (TCA cycle)	79	0.339075	1.224034	0.235043	0.35779	33
has05213	Endometrial cancer	150	0.272068	1.170274	0.210728	0.358383	34
has02010	ABC transporters	119	0.276538	1.187661	0.198842	0.3585	35
has04614	Renin-angiotensin system	47	0.39753	1.456591	0.054767	0.358809	36
has04723	Retrograde endocannabinoid signaling	238	0.274705	1.197846	0.191489	0.359558	37
has04520	Adherens junction	209	0.254126	1.191587	0.159091	0.36075	38
has04340	Hedgehog signaling pathway	117	0.274902	1.158822	0.223969	0.361507	39
has05143	African trypanosomiasis	88	0.32108	1.165718	0.254335	0.361926	40
has00785	Lipoic acid metabolism	5	0.613875	1.230365	0.239407	0.361996	41
has04713	Circadian entrainment	237	0.296213	1.385546	0.017176	0.362135	42

has05222	Small cell lung cancer	233	0.248741	1.16318	0.202268	0.36236	43
has00240	Pyrimidine metabolism	222	-0.36384	-1.57358	0.002105	0.362814	44
has05033	Nicotine addiction	97	0.294963	1.159922	0.260952	0.363488	45
has04728	Dopaminergic synapse	329	0.256075	1.235406	0.097701	0.363683	46
has04152	AMPK signaling pathway	314	0.260362	1.232236	0.122772	0.363854	47
has04921	Oxytocin signaling pathway	401	0.314681	1.463898	0.020755	0.365402	48
has04540	Gap junction	223	0.320658	1.39538	0.046512	0.366594	49
has00510	N-Glycan biosynthesis	109	-0.3919	-1.52725	0.02045	0.368193	50
has04911	Insulin secretion	205	0.263103	1.236012	0.106299	0.368524	51
has05214	Glioma	175	0.321501	1.38814	0.064272	0.370895	52
has04970	Salivary secretion	207	0.304181	1.399311	0.029644	0.372067	53
has04010	MAPK signaling pathway	593	0.24695	1.236456	0.10076	0.373886	54
has00360	Phenylalanine metabolism	49	0.325442	1.239605	0.160569	0.374091	55
has05211	Renal cell carcinoma	175	0.291744	1.242819	0.148297	0.374842	56
has05030	Cocaine addiction	139	0.2829	1.243492	0.141732	0.380007	57
has04725	Cholinergic synapse	290	0.246656	1.139377	0.225	0.382361	58
has03410	Base excision repair	91	-0.35358	-1.50511	0.027254	0.382704	59
has04710	Circadian rhythm	64	0.382118	1.431284	0.041494	0.384906	60
has00430	Taurine and hypotaurine metabolism	19	0.364111	1.139819	0.278226	0.38575	61
has04020	Calcium signaling pathway	419	0.258058	1.243994	0.108779	0.385807	62
has00071	Fatty acid degradation	109	0.357113	1.400191	0.085774	0.386712	63
has04726	Serotonergic synapse	278	0.324806	1.465956	0.023669	0.387228	64
has00380	Tryptophan metabolism	102	0.256479	1.1408	0.224	0.388433	65
has04060	Cytokine-cytokine receptor interaction	498	0.276027	1.120675	0.294455	0.391891	66
has04744	Phototransduction	80	0.292788	1.244255	0.105691	0.392145	67
has05020	Prion diseases	107	0.315031	1.250466	0.184314	0.39289	68
has05217	Basal cell carcinoma	118	0.292515	1.246458	0.149701	0.394471	69
has04510	Focal adhesion	501	0.254459	1.116854	0.287091	0.394751	70
has03460	Fanconi anemia pathway	106	-0.40325	-1.53856	0.062992	0.39484	71
has00480	Glutathione metabolism	107	0.261428	1.120887	0.29106	0.395388	72
has00250	Alanine, aspartate and glutamate metabolism	96	0.25831	1.122719	0.2423	0.396112	73
has04610	Complement and coagulation cascades	150	0.290316	1.123191	0.307985	0.399223	74
has00750	Vitamin B6 metabolism	17	0.453746	1.302921	0.183044	0.399639	75
has05410	Hypertrophic cardiomyopathy (HCM)	200	0.26936	1.124872	0.316794	0.400199	76
has04919	Thyroid hormone signaling pathway	336	0.258532	1.250559	0.087719	0.400276	77
has04014	Ras signaling pathway	552	0.235388	1.12703	0.246777	0.400429	78
has04740	Olfactory transduction	138	0.312468	1.401033	0.01996	0.402602	79
has04151	PI3K-Akt signaling pathway	789	0.235614	1.107476	0.265385	0.403498	80
has05032	Morphine addiction	207	0.250175	1.109514	0.275449	0.403546	81
has05014	Amyotrophic lateral sclerosis (ALS)	148	0.280482	1.251663	0.109827	0.40575	82
has04918	Thyroid hormone synthesis	167	0.28644	1.255518	0.134579	0.405798	83
has00280	Valine, leucine and isoleucine degradation	109	0.336954	1.303823	0.137712	0.40836	84
has04974	Protein digestion and absorption	179	0.304613	1.290897	0.129094	0.408664	85
has04973	Carbohydrate digestion and absorption	95	0.288552	1.257692	0.097196	0.40924	86

has04750	Inflammatory mediator regulation of TRP channels	270	0.279356	1.285722	0.096118	0.41112	87
has04320	Dorso-ventral axis formation	62	0.375438	1.404857	0.092843	0.4125	88
has05200	Pathways in cancer	848	0.248634	1.26367	0.074488	0.413468	89
has04920	Adipocytokine signaling pathway	203	0.290774	1.259345	0.147388	0.414326	90
has05031	Amphetamine addiction	187	0.290891	1.32001	0.061144	0.415756	91
has05206	MicroRNAs in cancer	409	0.273317	1.305279	0.076067	0.415818	92
has04972	Pancreatic secretion	221	0.272633	1.274953	0.106178	0.41644	93
has04720	Long-term potentiation	174	0.292275	1.291484	0.077213	0.417016	94
has05202	Transcriptional misregulation in cancer	446	0.275985	1.278747	0.097328	0.417181	95
has04080	Neuroactive ligand-receptor interaction	490	0.27573	1.265927	0.116142	0.417243	96
has05218	Melanoma	173	0.308812	1.35235	0.060311	0.417596	97
has03320	PPAR signaling pathway	198	0.302808	1.356659	0.082164	0.420759	98
has04261	Adrenergic signaling in cardiomyocytes	393	0.297921	1.409756	0.035156	0.422346	99
has04110	Cell cycle	299	-0.36682	-1.5819	0.033543	0.422701	100
has04360	Axon guidance	299	0.270346	1.266533	0.076628	0.424929	101
has04724	Glutamatergic synapse	280	0.292501	1.30604	0.092486	0.425598	102
has00260	Glycine, serine and threonine metabolism	108	0.305278	1.320933	0.082816	0.42621	103
has04150	mTOR signaling pathway	147	0.296021	1.31067	0.066798	0.426325	104
has00010	Glycolysis / Gluconeogenesis	184	0.244167	1.092344	0.283096	0.426681	105
has04912	GnRH signaling pathway	243	0.234695	1.084723	0.283019	0.437527	106
has04530	Tight junction	312	0.27797	1.321111	0.071291	0.439037	107
has00053	Ascorbate and aldarate metabolism	43	0.309798	1.080339	0.351779	0.441682	108
has04730	Long-term depression	153	0.324517	1.323896	0.095517	0.445799	109
has04971	Gastric acid secretion	187	0.285218	1.329164	0.060078	0.447163	110
has00410	beta-Alanine metabolism	81	0.26094	1.06877	0.337526	0.459244	111
has04390	Hippo signaling pathway	353	0.282102	1.32988	0.052525	0.461004	112
has04810	Regulation of actin cytoskeleton	535	0.22966	1.063982	0.341509	0.463632	113
has03020	RNA polymerase	75	-0.39756	-1.60438	0.006211	0.468645	114
has05216	Thyroid cancer	91	0.247443	1.048257	0.371769	0.472434	115
has00040	Pentose and glucuronate interconversions	48	0.293656	1.050056	0.386454	0.47342	116
has00561	Glycerolipid metabolism	122	0.245661	1.055103	0.368952	0.476948	117
has05210	Colorectal cancer	183	0.224378	1.050125	0.337325	0.477491	118
has00532	Glycosaminoglycan biosynthesis - chondroitin sulfate / dermatan sulfate	28	0.319399	1.052323	0.400763	0.477614	119
has00400	Phenylalanine, tyrosine and tryptophan biosynthesis	9	-0.66319	-1.45375	0.093567	0.49486	120
has00591	Linoleic acid metabolism	51	0.262666	1.032838	0.411647	0.497688	121
has03010	Ribosome	322	0.238268	1.023999	0.399177	0.507351	122
has04978	Mineral absorption	115	0.228737	1.025367	0.354406	0.508699	123
has03030	DNA replication	88	-0.50023	-1.64078	0.038462	0.514901	124
has04915	Estrogen signaling pathway	278	0.217719	1.015417	0.425926	0.516109	125
has04630	Jak-STAT signaling pathway	325	0.237967	1.016214	0.453668	0.519125	126
has04668	TNF signaling pathway	302	0.227367	1.011203	0.418387	0.520177	127
has00650	Butanoate metabolism	63	0.255888	1.005479	0.435789	0.527148	128
has00830	Retinol metabolism	99	0.487452	1.748611	0.008114	0.529909	129

has03430	Mismatch repair	57	-0.44381	-1.4206	0.108527	0.550209	130
has04623	Cytosolic DNA-sensing pathway	130	-0.30906	-1.26839	0.152	0.557227	131
has00860	Porphyrin and chlorophyll metabolism	85	0.248241	0.98621	0.485772	0.563515	132
has00563	Glycosylphosphatidylinositol(GPI)-anchor biosynthesis	54	-0.32875	-1.24867	0.133065	0.570466	133
has00760	Nicotinate and nicotinamide metabolism	45	0.276231	0.980341	0.50813	0.571882	134
has00620	Pyruvate metabolism	107	0.235414	0.977196	0.469474	0.573216	135
has04115	p53 signaling pathway	182	-0.27304	-1.27974	0.088115	0.573294	136
has00061	Fatty acid biosynthesis	19	-0.40594	-1.27133	0.160784	0.573558	137
has00120	Primary bile acid biosynthesis	44	0.28311	0.973199	0.473361	0.57693	138
has00270	Cysteine and methionine metabolism	98	-0.29153	-1.25069	0.115686	0.586509	139
has04120	Ubiquitin mediated proteolysis	320	-0.27037	-1.28386	0.084848	0.586784	140
has05166	HTLV-I infection	660	0.204094	0.959935	0.524085	0.591326	141
has00564	Glycerophospholipid metabolism	180	0.214612	0.964256	0.547284	0.591695	142
has04062	Chemokine signaling pathway	438	0.218409	0.961405	0.49904	0.592695	143
has04012	ErbB signaling pathway	238	0.21439	0.953775	0.537718	0.595232	144
has04727	GABAergic synapse	204	0.214067	0.951014	0.51462	0.596801	145
has04066	HIF-1 signaling pathway	286	0.215253	0.954979	0.519481	0.597553	146
has03018	RNA degradation	161	0.198006	0.945997	0.582822	0.60295	147
has03060	Protein export	66	-0.34341	-1.2876	0.143737	0.602975	148
has00520	Amino sugar and nucleotide sugar metabolism	125	-0.31959	-1.31506	0.111789	0.608724	149
has05322	Systemic lupus erythematosus	201	-0.34821	-1.38938	0.093496	0.60904	150
has00100	Steroid biosynthesis	41	-0.36484	-1.29453	0.148297	0.611164	151
has03420	Nucleotide excision repair	104	-0.33425	-1.33541	0.099404	0.613477	152
has04142	Lysosome	291	-0.28787	-1.18483	0.240964	0.615827	153
has04070	Phosphatidylinositol signaling system	225	-0.24491	-1.10641	0.277778	0.617007	154
has00740	Riboflavin metabolism	34	-0.33471	-1.15426	0.257937	0.617119	155
has00600	Sphingolipid metabolism	85	-0.29041	-1.15825	0.235412	0.622049	156
has04914	Progesterone-mediated oocyte maturation	247	-0.25304	-1.18917	0.169165	0.62253	157
has00533	Glycosaminoglycan biosynthesis - keratan sulfate	23	-0.42154	-1.30073	0.143145	0.622809	158
has05160	Hepatitis C	320	-0.24601	-1.10846	0.267894	0.62538	159
has00601	Glycosphingolipid biosynthesis - lacto and neolacto series	38	-0.3124	-1.16289	0.241517	0.626361	160
has00290	Valine, leucine and isoleucine biosynthesis	7	-0.49197	-1.11315	0.363813	0.627252	161
has00970	Aminoacyl-tRNA biosynthesis	114	-0.28838	-1.19416	0.2	0.627764	162
has03040	Spliceosome	234	-0.2468	-1.09754	0.304436	0.628002	163
has03008	Ribosome biogenesis in eukaryotes	166	-0.26759	-1.12271	0.302714	0.629838	164
has00511	Other glycan degradation	52	-0.3168	-1.13742	0.258824	0.632719	165
has03050	Proteasome	114	-0.29089	-1.1265	0.295316	0.633538	166
has04721	Synaptic vesicle cycle	152	-0.27056	-1.13122	0.240964	0.635241	167
has00051	Fructose and mannose metabolism	94	-0.28144	-1.14141	0.267391	0.637071	168
has04612	Antigen processing and presentation	156	-0.3229	-1.16504	0.302846	0.637957	169

has04622	RIG-I-like receptor signaling pathway	154	-0.27412	-1.11405	0.297787	0.639212	170
has00512	Mucin type O-Glycan biosynthesis	48	-0.34098	-1.33968	0.068273	0.642059	171
has00232	Caffeine metabolism	16	-0.39578	-1.19557	0.225296	0.644242	172
has00230	Purine metabolism	378	-0.23193	-1.16854	0.133733	0.645429	173
has04141	Protein processing in endoplasmic reticulum	386	-0.278	-1.31517	0.066929	0.646314	174
has03015	mRNA surveillance pathway	198	-0.25781	-1.20086	0.140562	0.649538	175
has04917	Prolactin signaling pathway	223	0.203383	0.920871	0.601905	0.652362	176
has03013	RNA transport	346	-0.25549	-1.20557	0.148936	0.657688	177
has00780	Biotin metabolism	7	-0.57326	-1.36068	0.116935	0.660909	178
has05110	Vibrio cholerae infection	137	-0.29073	-1.21152	0.171079	0.66379	179
has05146	Amoebiasis	274	0.214761	0.908789	0.581749	0.663907	180
has05132	Salmonella infection	210	0.198293	0.901991	0.624278	0.664274	181
has00770	Pantothenate and CoA biosynthesis	50	0.236588	0.911868	0.604938	0.667541	182
has00340	Histidine metabolism	67	0.211844	0.909134	0.659229	0.668269	183
has04670	Leukocyte transendothelial migration	272	0.213057	0.902228	0.581132	0.668587	184
has03450	Non-homologous end-joining	36	-0.38075	-1.34496	0.088933	0.67006	185
has04210	Apoptosis	228	0.199746	0.903464	0.655832	0.670517	186
has04611	Platelet activation	329	0.208835	0.893132	0.620818	0.678508	187
has00562	Inositol phosphate metabolism	158	0.211737	0.888094	0.62949	0.684958	188
has05203	Viral carcinogenesis	522	-0.21619	-1.061	0.324895	0.688152	189
has04114	Oocyte meiosis	293	-0.21545	-1.06381	0.335391	0.694253	190
has05164	Influenza A	391	-0.24348	-1.06504	0.353659	0.705005	191
has04144	Endocytosis	449	-0.19674	-0.94292	0.57438	0.727059	192
has00592	alpha-Linolenic acid	45	-0.24569	-0.92431	0.58317	0.728915	193
has05169	Epstein-Barr virus infection	533	-0.21706	-1.02339	0.396341	0.732087	194
has04064	NF-kappa B signaling pathway	231	-0.26058	-1.01858	0.421712	0.732487	195
has00052	Galactose metabolism	78	-0.25994	-1.03108	0.402344	0.735838	196
has00310	Lysine degradation	108	-0.22291	-0.93601	0.542574	0.735913	197
has04662	B cell receptor signaling pathway	192	-0.22635	-0.94316	0.504202	0.736149	198
has05134	Legionellosis	151	-0.23332	-0.92815	0.526839	0.737305	199
has04722	Neurotrophin signaling pathway	339	-0.18655	-0.92438	0.603734	0.737862	200
has00603	Glycosphingolipid biosynthesis - globo series	27	-0.31974	-1.0388	0.41875	0.73939	201
has05152	Tuberculosis	426	-0.22669	-0.94566	0.505198	0.739685	202
has00330	Arginine and proline	130	-0.21843	-0.9302	0.584158	0.741414	203
has00534	Glycosaminoglycan biosynthesis - heparan sulfate / heparin	44	-0.28174	-1.03355	0.405295	0.741846	204
has00460	Cyanoamino acid metabolism	14	-0.39977	-1.02344	0.453416	0.74528	205
has05161	Hepatitis B	375	-0.21566	-1.00488	0.429752	0.745742	206
has05131	Shigellosis	165	-0.21803	-0.97075	0.471933	0.746375	207
has00450	Selenocompound metabolism	45	-0.2747	-1.00807	0.451613	0.749104	208
has05168	Herpes simplex infection	398	-0.21522	-0.94586	0.520408	0.749157	209
has00910	Nitrogen metabolism	47	-0.26039	-0.99927	0.48062	0.749872	210
has04962	Vasopressin-regulated water reabsorption	114	-0.2177	-0.90769	0.585657	0.753263	211
has00604	Glycosphingolipid biosynthesis - ganglio series	38	-0.28107	-0.94807	0.511628	0.753311	212
has04122	Sulfur relay system	22	-0.3293	-0.97229	0.501938	0.753367	213

has05220	Chronic myeloid leukemia	214	-0.21912	-0.95882	0.494577	0.756525	214
has05034	Alcoholism	350	-0.19383	-0.95391	0.537657	0.758868	215
has00030	Pentose phosphate pathway	72	-0.22653	-0.9082	0.609284	0.761202	216
has04512	ECM-receptor interaction	195	-0.24193	-0.94876	0.509317	0.761752	217
has05162	Measles	331	-0.23152	-0.97322	0.46856	0.762183	218
has04145	Phagosome	331	-0.23408	-0.96032	0.477273	0.763672	219
has04146	Peroxisome	175	-0.23096	-0.98869	0.442991	0.767563	220
has05120	Epithelial cell signaling in Helicobacter pylori infection	178	0.181426	0.847484	0.758221	0.769484	221
has04664	Fc epsilon RI signaling pathway	186	-0.21033	-0.89248	0.576842	0.772494	222
has04950	Maturity onset diabetes of the young	69	-0.24976	-0.97374	0.486815	0.772498	223
has04350	TGF-beta signaling pathway	205	-0.20782	-0.98233	0.49789	0.773439	224
has05340	Primary immunodeficiency	96	-0.31195	-0.97726	0.484407	0.775162	225
has05219	Bladder cancer	100	-0.21211	-0.89327	0.629787	0.779745	226
has00983	Drug metabolism - other enzymes	78	0.194833	0.829182	0.769857	0.788441	227
has00072	Synthesis and degradation of ketone bodies	24	0.248666	0.831444	0.738758	0.788765	228
has04514	Cell adhesion molecules (CAMs)	294	0.204883	0.825253	0.652751	0.791516	229
has04380	Osteoclast differentiation	335	0.208239	0.831467	0.656489	0.794266	230
has04742	Taste transduction	68	0.221686	0.83298	0.698842	0.796455	231
has04370	VEGF signaling pathway	160	0.196908	0.810135	0.781784	0.807045	232
has00190	Oxidative phosphorylation	272	-0.21464	-0.86773	0.64497	0.808378	233
has05100	Bacterial invasion of epithelial cells	208	-0.19872	-0.86393	0.662393	0.808967	234
has05140	Leishmaniasis	161	0.232991	0.805392	0.670588	0.811179	235
has04621	NOD-like receptor signaling pathway	131	0.18762	0.81307	0.798058	0.812014	236
has04932	Non-alcoholic fatty liver disease (NAFLD)	373	0.170552	0.810213	0.845679	0.812285	237
has04620	Toll-like receptor signaling pathway	244	-0.20616	-0.86891	0.618557	0.814364	238
has05016	Huntington,s disease	452	-0.17793	-0.85482	0.732673	0.814374	239
has05142	Chagas disease (American trypanosomiasis)	274	-0.20211	-0.87257	0.626556	0.81451	240
has04330	Notch signaling pathway	118	-0.19548	-0.85807	0.76569	0.815157	241
has05010	Alzheimer,s disease	411	0.172895	0.794926	0.823529	0.826421	242
has05212	Pancreatic cancer	206	-0.18071	-0.84183	0.793177	0.836587	243
has05133	Pertussis	205	0.201426	0.786571	0.733333	0.836786	244
has04961	Endocrine and other factor-regulated calcium reabsorption	127	-0.17773	-0.80428	0.885714	0.845979	245
has04666	Fc gamma R-mediated phagocytosis	226	-0.18701	-0.79921	0.7473	0.847839	246
has05221	Acute myeloid leukemia	185	0.183902	0.775947	0.834615	0.850667	247
has00670	One carbon pool by folate	53	-0.20712	-0.80516	0.796813	0.852422	248
has00565	Ether lipid metabolism	79	-0.21275	-0.80643	0.76556	0.858536	249
has04966	Collecting duct acid secretion	65	-0.21681	-0.82098	0.737575	0.859632	250
has03022	Basal transcription factors	103	-0.18682	-0.82425	0.828974	0.861181	251
has05145	Toxoplasmosis	296	-0.18939	-0.82695	0.706967	0.864036	252
has04130	SNARE interactions in vesicular transport	73	-0.19458	-0.80682	0.820408	0.866329	253
has05012	Parkinson,s disease	322	0.175029	0.760698	0.872385	0.866506	254
has04140	Regulation of autophagy	85	0.178316	0.763512	0.895706	0.86703	255

has05130	Pathogenic Escherichia coli infection	146	-0.19158	-0.80827	0.763103	0.872009	256
has00514	Other types of O-glycan biosynthesis	55	-0.20076	-0.80854	0.778672	0.880439	257
has00471	D-Glutamine and D-glutamate metabolism	12	0.271809	0.740062	0.826336	0.888827	258
has01040	Biosynthesis of unsaturated fatty acids	42	-0.23049	-0.77044	0.765625	0.890866	259
has04640	Hematopoietic cell lineage	195	0.211264	0.740545	0.738636	0.893622	260
has04660	T cell receptor signaling pathway	283	-0.18422	-0.7724	0.746362	0.895409	261
has04650	Natural killer cell mediated cytotoxicity	283	0.188006	0.718534	0.796154	0.9136	262
has05416	Viral myocarditis	132	0.207403	0.711753	0.753372	0.916925	263
has00900	Terpenoid backbone biosynthesis	57	-0.19221	-0.75114	0.88932	0.918673	264
has00130	Ubiquinone and other terpenoid-quinone biosynthesis	29	0.224327	0.698305	0.857741	0.927039	265
has05150	Staphylococcus aureus infection	96	0.217176	0.677057	0.779961	0.943017	266
has05144	Malaria	129	-0.21312	-0.70959	0.769231	0.94723	267
has05323	Rheumatoid arthritis	197	-0.18609	-0.71481	0.802419	0.949086	268
has00790	Folate biosynthesis	36	-0.21563	-0.71827	0.841785	0.953076	269
has00062	Fatty acid elongation	51	-0.19887	-0.72363	0.882704	0.953994	270
has05321	Inflammatory bowel disease (IBD)	146	0.176906	0.636911	0.888889	0.966745	271
has05330	Allograft rejection	81	-0.21624	-0.64083	0.77572	0.979192	272
has00920	Sulfur metabolism	25	-0.19768	-0.60477	0.943026	0.981225	273
has05332	Graft-versus-host disease	81	0.207023	0.590494	0.829412	0.9825	274
has05310	Asthma	73	-0.18574	-0.61963	0.863179	0.983264	275
has04940	Type I diabetes mellitus	92	-0.2015	-0.6443	0.808247	0.985558	276
has04672	Intestinal immune network for IgA production	96	0.165266	0.554074	0.923077	0.987021	277
has00524	Butirosin and neomycin biosynthesis	15	-0.23087	-0.66349	0.907975	0.988375	278
has00730	Thiamine metabolism	7	-0.23869	-0.53487	0.970817	0.991578	279
has05320	Autoimmune thyroid disease	93	-0.20147	-0.64454	0.804124	0.994204	280

All results in BRCA dataset by GSEA

Pathway ID	Pathway Name	SIZE	ES	NES	NOM p-va	FDR q-val	Rank
hsa05219	Bladder cancer	36	-0.36532	-2.07693	0.002105	0.451083	1
hsa04142	Lysosome	119	-0.25304	-1.66657	0.107505	0.55353	2
hsa04141	Protein processing in endoplasmic reticulum	153	-0.1931	-1.70167	0.043033	0.573529	3
hsa04623	Cytosolic DNA-sensing pathway	56	-0.24479	-1.67152	0.060041	0.593393	4
hsa03030	DNA replication	35	-0.43115	-1.70768	0.057447	0.622281	5
hsa05212	Pancreatic cancer	66	-0.17844	-1.30226	0.196787	0.626871	6
hsa00230	Purine metabolism	154	-0.09493	-1.2601	0.194861	0.632383	7
hsa05206	MicroRNAs in cancer	143	-0.11091	-1.28368	0.205128	0.632785	8
hsa04740	Olfactory transduction	303	-0.21076	-1.83705	0.053045	0.632823	9
hsa00051	Fructose and mannose metabolism	30	-0.24334	-1.31233	0.178862	0.639357	10
hsa05110	Vibrio cholerae infection	51	-0.20449	-1.28687	0.238866	0.641335	11
hsa05320	Autoimmune thyroid disease	47	-0.27567	-1.30275	0.252101	0.641866	12
hsa00600	Sphingolipid metabolism	35	-0.17771	-1.26191	0.169043	0.643713	13

hsa00120	Primary bile acid biosynthesis	17	0.285018	1.375015	0.111111	0.644543	14
hsa03420	Nucleotide excision repair	43	-0.19997	-1.31828	0.183099	0.645543	15
hsa00500	Starch and sucrose metabolism	44	0.20817	1.366007	0.137525	0.645759	16
hsa05120	Epithelial cell signaling in Helicobacter pylori infection	65	-0.13047	-1.04788	0.393509	0.647113	17
hsa03015	mRNA surveillance pathway	78	-0.12524	-1.05221	0.405242	0.648318	18
hsa00603	Glycosphingolipid biosynthesis - globo series	14	-0.30102	-1.2661	0.189691	0.650968	19
hsa05217	Basal cell carcinoma	55	0.181563	1.354115	0.132673	0.652172	20
hsa04950	Maturity onset diabetes of the young	24	-0.23073	-1.05485	0.366803	0.652505	21
hsa03013	RNA transport	138	-0.11983	-1.07487	0.385859	0.654787	22
hsa00510	N-Glycan biosynthesis	46	-0.30528	-1.93212	0.018036	0.656918	23
hsa03008	Ribosome biogenesis in eukaryotes	63	-0.18277	-1.23983	0.254098	0.658132	24
hsa00190	Oxidative phosphorylation	115	-0.175	-1.08315	0.390476	0.658992	25
hsa05416	Viral myocarditis	55	-0.20652	-1.05586	0.421488	0.659367	26
hsa05160	Hepatitis C	124	-0.1158	-1.07745	0.372745	0.659648	27
hsa04612	Antigen processing and presentation	68	-0.27022	-1.31879	0.254697	0.66221	28
hsa04115	p53 signaling pathway	63	-0.12637	-1.05893	0.39413	0.662746	29
hsa00400	Phenylalanine, tyrosine and tryptophan biosynthesis	5	-0.42711	-1.11357	0.305439	0.664438	30
hsa00040	Pentose and glucuronate interconversions	25	0.290033	1.375063	0.169043	0.664495	31
hsa04662	B cell receptor signaling pathway	70	-0.15943	-1.063	0.384921	0.66532	32
hsa04370	VEGF signaling pathway	59	-0.15083	-1.08488	0.358416	0.665668	33
hsa04622	RIG-I-like receptor signaling pathway	63	-0.14044	-1.09555	0.342159	0.666755	34
hsa03040	Spliceosome	100	-0.18633	-1.40272	0.16805	0.669329	35
hsa05162	Measles	129	-0.12744	-1.10417	0.354839	0.671465	36
hsa05100	Bacterial invasion of epithelial cells	74	-0.14258	-1.02666	0.412574	0.67309	37
hsa04914	Progesterone-mediated oocyte maturation	84	-0.12673	-1.08553	0.349495	0.674209	38
hsa00740	Riboflavin metabolism	12	-0.27679	-1.11383	0.330739	0.674472	39
hsa05211	Renal cell carcinoma	66	-0.14599	-1.09623	0.357002	0.675322	40
hsa05152	Tuberculosis	168	-0.12226	-1.11903	0.346232	0.676138	41
hsa05010	Alzheimer,s disease	155	-0.13437	-1.12489	0.364865	0.676187	42
hsa00524	Butirosin and neomycin biosynthesis	5	-0.37346	-1.01926	0.411417	0.676914	43
hsa05012	Parkinson,s disease	122	-0.15524	-1.01356	0.425856	0.678168	44
hsa00511	Other glycan degradation	17	-0.3263	-1.31953	0.186475	0.679426	45
hsa04966	Collecting duct acid secretion	27	-0.26316	-1.32848	0.159596	0.679596	46
hsa04390	Hippo signaling pathway	146	0.116693	1.323719	0.186139	0.681306	47
hsa05016	Huntington,s disease	166	-0.13347	-1.12717	0.344294	0.682526	48
hsa05323	Rheumatoid arthritis	81	-0.1835	-1.16441	0.318182	0.682557	49
hsa04610	Complement and coagulation cascades	66	0.232446	1.375467	0.19305	0.685304	50
hsa05340	Primary immunodeficiency	35	-0.28405	-1.13112	0.348993	0.685865	51
hsa04110	Cell cycle	114	-0.28397	-1.71258	0.033473	0.687539	52
hsa00534	Glycosaminoglycan biosynthesis - heparan sulfate / heparin	24	-0.19113	-1.00133	0.420432	0.688388	53

hsa00563	Glycosylphosphatidylinositol(GPI)-anchor biosynthesis	25	-0.21665	-1.13545	0.317554	0.689509	54
hsa00290	Valine, leucine and isoleucine biosynthesis	4	-0.64054	-1.40431	0.112676	0.691249	55
hsa05130	Pathogenic Escherichia coli infection	51	-0.18833	-1.16613	0.28145	0.691848	56
hsa05168	Herpes simplex infection	158	-0.14883	-1.33255	0.212	0.692331	57
hsa03060	Protein export	22	-0.27678	-1.21488	0.26494	0.69355	58
hsa05221	Acute myeloid leukemia	56	-0.17118	-1.1389	0.336595	0.694854	59
hsa00100	Steroid biosynthesis	17	-0.29971	-1.15047	0.319588	0.696945	60
hsa05034	Alcoholism	164	-0.22128	-1.5902	0.091977	0.697234	61
hsa00591	Linoleic acid metabolism	26	0.236361	1.324539	0.162055	0.698328	62
hsa00514	Other types of O-glycan biosynthesis	22	-0.23919	-1.20357	0.259481	0.701279	63
hsa04510	Focal adhesion	198	0.146468	1.305672	0.2334	0.702087	64
hsa04940	Type I diabetes mellitus	40	-0.27718	-1.1412	0.327766	0.702465	65
hsa04114	Oocyte meiosis	106	-0.13172	-1.16685	0.275574	0.70356	66
hsa04261	Adrenergic signaling in cardiomyocytes	146	0.110083	1.263813	0.223382	0.704877	67
hsa04360	Axon guidance	125	0.136433	1.375859	0.164751	0.707414	68
hsa00270	Cysteine and methionine metabolism	35	-0.17204	-1.1928	0.244813	0.708281	69
hsa03440	Homologous recombination	25	-0.40183	-1.7364	0.034483	0.709533	70
hsa00970	Aminoacyl-tRNA biosynthesis	42	-0.26488	-1.33394	0.204453	0.710318	71
hsa05161	Hepatitis B	138	-0.10955	-1.17025	0.28373	0.710477	72
hsa04022	cGMP-PKG signaling pathway	160	0.144122	1.428948	0.155925	0.711617	73
hsa03430	Mismatch repair	23	-0.31298	-1.34344	0.179226	0.711774	74
hsa04744	Phototransduction	27	0.209672	1.292085	0.169043	0.713274	75
hsa04721	Synaptic vesicle cycle	63	-0.21285	-1.42861	0.144269	0.718365	76
hsa04913	Ovarian steroidogenesis	50	0.189295	1.403354	0.097363	0.718389	77
hsa04020	Calcium signaling pathway	171	0.11154	1.265243	0.22428	0.718474	78
hsa00531	Glycosaminoglycan degradation	19	-0.33305	-1.40438	0.142857	0.718819	79
hsa03020	RNA polymerase	29	-0.22102	-1.1727	0.290456	0.719321	80
hsa04064	NF-kappa B signaling pathway	81	-0.17984	-1.17983	0.310127	0.719579	81
hsa04120	Ubiquitin mediated proteolysis	126	-0.13336	-1.41544	0.122807	0.719902	82
hsa05169	Epstein-Barr virus infection	190	-0.15926	-1.56237	0.115304	0.72063	83
hsa05166	HTLV-I infection	250	-0.08088	-0.97016	0.455301	0.722367	84
hsa02010	ABC transporters	44	0.187504	1.278867	0.206897	0.724702	85
hsa05310	Asthma	27	-0.2563	-0.97281	0.451346	0.726967	86
hsa00300	Lysine biosynthesis	2	0.834999	1.434615	0.090909	0.727055	87
hsa04620	Toll-like receptor signaling pathway	96	-0.12116	-0.96196	0.461847	0.727166	88
hsa00630	Glyoxylate and dicarboxylate metabolism	21	0.30834	1.268241	0.237895	0.729542	89
hsa05222	Small cell lung cancer	84	0.118673	1.132993	0.310484	0.729679	90
hsa00360	Phenylalanine metabolism	18	0.30623	1.375976	0.129482	0.731418	91
hsa05131	Shigellosis	60	-0.20969	-1.34454	0.190574	0.732717	92
hsa03410	Base excision repair	33	-0.37969	-1.85197	0.018868	0.737591	93
hsa05033	Nicotine addiction	38	0.180841	1.134784	0.285421	0.738581	94
hsa05216	Thyroid cancer	29	-0.22894	-1.35255	0.153543	0.738806	95
hsa00280	Valine, leucine and isoleucine degradation	43	0.291888	1.441535	0.135021	0.740378	96
hsa03320	PPAR signaling pathway	68	0.226508	1.40509	0.175105	0.740702	97
hsa03010	Ribosome	126	0.267284	1.119543	0.364522	0.744919	98

hsa04080	Neuroactive ligand-receptor interaction	251	0.119355	1.235836	0.279749	0.748397	99
hsa05215	Prostate cancer	87	-0.10321	-0.93902	0.532787	0.74901	100
hsa04713	Circadian entrainment	93	0.118469	1.135654	0.322981	0.749592	101
hsa00533	Glycosaminoglycan biosynthesis - keratan sulfate	13	-0.32802	-1.42912	0.110187	0.749958	102
hsa04726	Serotonergic synapse	110	0.121526	1.378724	0.142857	0.750783	103
hsa04964	Proximal tubule bicarbonate reclamation	23	0.237326	1.210455	0.229039	0.751321	104
hsa00760	Nicotinate and nicotinamide metabolism	20	0.222345	1.14102	0.296443	0.751488	105
hsa05218	Melanoma	70	0.132254	1.084709	0.34433	0.754399	106
hsa03450	Non-homologous end-joining	13	-0.2285	-0.93983	0.501887	0.756302	107
hsa04970	Salivary secretion	84	0.129828	1.200814	0.236842	0.756446	108
hsa04520	Adherens junction	73	0.12888	1.089492	0.333333	0.756856	109
hsa00350	Tyrosine metabolism	39	0.201251	1.21562	0.264646	0.756882	110
hsa04728	Dopaminergic synapse	125	0.107248	1.193779	0.27907	0.757167	111
hsa05205	Proteoglycans in cancer	215	0.093521	1.107607	0.334004	0.757621	112
hsa04144	Endocytosis	176	-0.12008	-1.35421	0.183794	0.761047	113
hsa00471	D-Glutamine and D-glutamate metabolism	4	0.501544	1.142446	0.30621	0.761846	114
hsa04960	Aldosterone-regulated sodium reabsorption	36	0.157057	1.091725	0.356855	0.763976	115
hsa00983	Drug metabolism - other enzymes	37	0.176479	1.14782	0.295367	0.764743	116
hsa04270	Vascular smooth muscle contraction	113	0.15383	1.44469	0.123232	0.765192	117
hsa04920	Adipocytokine signaling pathway	69	0.171022	1.182026	0.298539	0.767295	118
hsa04530	Tight junction	123	0.114116	1.217139	0.240079	0.770299	119
hsa05032	Morphine addiction	90	0.129632	1.094777	0.364198	0.770396	120
hsa04710	Circadian rhythm	27	0.221935	1.165423	0.291846	0.771548	121
hsa04921	Oxytocin signaling pathway	153	0.10881	1.158292	0.318458	0.771572	122
hsa00650	Butanoate metabolism	23	0.247111	1.149307	0.288382	0.775649	123
hsa03050	Proteasome	43	-0.34126	-1.43243	0.172199	0.776709	124
hsa04972	Pancreatic secretion	88	0.156815	1.495767	0.081761	0.779744	125
hsa04976	Bile secretion	68	0.138427	1.167298	0.278884	0.783073	126
hsa03018	RNA degradation	66	-0.10659	-0.91176	0.511202	0.786449	127
hsa00910	Nitrogen metabolism	17	0.339991	1.463286	0.11691	0.788471	128
hsa05204	Chemical carcinogenesis	65	0.318688	1.902656	0.036585	0.788664	129
hsa03460	Fanconi anemia pathway	43	-0.27113	-1.44212	0.147368	0.788852	130
hsa04150	mTOR signaling pathway	54	0.183433	1.44819	0.104418	0.792101	131
hsa00330	Arginine and proline	53	0.129174	1.052927	0.395112	0.793047	132
hsa00240	Pyrimidine metabolism	90	-0.22236	-1.74475	0.045267	0.79741	133
hsa04151	PI3K-Akt signaling pathway	322	0.06435	0.865615	0.533865	0.79828	134
hsa04977	Vitamin digestion and absorption	22	0.18542	1.043966	0.373254	0.799077	135
hsa04660	T cell receptor signaling pathway	103	-0.11695	-0.8982	0.532909	0.799795	136
hsa00430	Taurine and hypotaurine metabolism	7	0.324255	1.054616	0.375	0.801458	137
hsa04145	Phagosome	139	-0.19236	-1.45138	0.162	0.802482	138
hsa05030	Cocaine addiction	49	0.110132	0.865812	0.612033	0.805774	139

hsa00520	Amino sugar and nucleotide sugar metabolism	47	-0.25371	-1.51492	0.110879	0.806815	140
hsa00480	Glutathione metabolism	44	0.149501	1.010987	0.416499	0.807375	141
hsa00020	Citrate cycle (TCA cycle)	29	0.207123	0.873215	0.545267	0.809301	142
hsa04630	Jak-STAT signaling pathway	143	0.093418	0.877898	0.547431	0.809697	143
hsa04911	Insulin secretion	82	0.084586	0.85355	0.644764	0.810375	144
hsa04014	Ras signaling pathway	217	0.07221	0.867401	0.563126	0.810937	145
hsa04146	Peroxisome	72	0.119514	0.886272	0.516807	0.812084	146
hsa04971	Gastric acid secretion	70	0.111769	1.002662	0.431953	0.812599	147
hsa00250	Alanine, aspartate and glutamate metabolism	34	0.142192	1.012409	0.406639	0.815667	148
hsa04727	GABAergic synapse	87	0.083907	0.878739	0.581443	0.81644	149
hsa00785	Lipoic acid metabolism	2	0.909331	1.468028	0.058577	0.816818	150
hsa01040	Biosynthesis of unsaturated fatty acids	17	0.213552	0.886443	0.570248	0.820312	151
hsa05414	Dilated cardiomyopathy	90	0.097013	0.841239	0.605263	0.822509	152
hsa05200	Pathways in cancer	318	0.066839	1.014136	0.412574	0.823683	153
hsa00620	Pyruvate metabolism	40	0.296245	1.495778	0.105809	0.825518	154
hsa05202	Transcriptional misregulation in cancer	168	0.079567	0.888168	0.547619	0.825963	155
hsa05213	Endometrial cancer	52	-0.13138	-0.86149	0.590264	0.826015	156
hsa05332	Graft-versus-host disease	37	-0.24193	-0.85587	0.545648	0.826809	157
hsa04512	ECM-receptor interaction	85	0.129973	0.908013	0.532359	0.826875	158
hsa04724	Glutamatergic synapse	112	0.093628	1.017308	0.420408	0.828716	159
hsa04918	Thyroid hormone synthesis	68	0.096554	0.896391	0.52907	0.829275	160
hsa04350	TGF-beta signaling pathway	77	0.11657	1.022138	0.421712	0.830525	161
hsa00232	Caffeine metabolism	5	0.342658	0.890159	0.557344	0.831387	162
hsa05220	Chronic myeloid leukemia	72	-0.12187	-0.86304	0.580321	0.832199	163
hsa05223	Non-small cell lung cancer	55	-0.1234	-0.87302	0.560547	0.832798	164
hsa00062	Fatty acid elongation	19	0.216977	0.899484	0.546219	0.832935	165
hsa04742	Taste transduction	44	0.117742	0.90841	0.585513	0.835294	166
hsa04015	Rap1 signaling pathway	200	0.087759	0.97307	0.416335	0.835428	167
hsa00561	Glycerolipid metabolism	50	0.122238	0.817488	0.664	0.837213	168
hsa04720	Long-term potentiation	65	0.105082	0.928262	0.550102	0.837887	169
hsa04068	FoxO signaling pathway	127	0.080526	0.827063	0.624254	0.838013	170
hsa00061	Fatty acid biosynthesis	6	0.324012	0.982806	0.458586	0.838217	171
hsa04666	Fc gamma R-mediated phagocytosis	86	-0.18465	-1.45407	0.133201	0.839171	172
hsa00472	D-Arginine and D-ornithine metabolism	1	0.695166	0.921751	0.597959	0.839632	173
hsa04614	Renin-angiotensin system	17	0.173466	0.796196	0.693252	0.840006	174
hsa04810	Regulation of actin cytoskeleton	203	0.069183	0.800898	0.625251	0.840106	175
hsa04917	Prolactin signaling pathway	72	-0.10793	-0.86336	0.58517	0.840664	176
hsa00072	Synthesis and degradation of ketone bodies	8	0.222355	0.790623	0.726733	0.841022	177
hsa04723	Retrograde endocannabinoid signaling	98	0.094643	0.97564	0.459746	0.841228	178
hsa04730	Long-term depression	56	0.116262	0.909253	0.519348	0.843037	179
hsa04540	Gap junction	81	0.102207	0.818221	0.63745	0.843809	180
hsa04664	Fc epsilon RI signaling pathway	67	-0.11065	-0.8401	0.624506	0.844473	181
hsa05143	African trypanosomiasis	29	0.163761	0.807905	0.645793	0.844781	182
hsa00770	Pantothenate and CoA biosynthesis	17	-0.18433	-0.82496	0.652087	0.844804	183
hsa04310	Wnt signaling pathway	136	0.083738	0.92941	0.51	0.845305	184

hsa04320	Dorso-ventral axis formation	24	0.172906	0.913032	0.528926	0.845936	185
hsa00140	Steroid hormone biosynthesis	47	0.275943	1.624403	0.064257	0.847122	186
hsa05031	Amphetamine addiction	66	0.193907	1.651075	0.044211	0.847386	187
hsa04916	Melanogenesis	98	0.079692	0.800956	0.682	0.847632	188
hsa04650	Natural killer cell mediated cytotoxicity	124	-0.11865	-0.82791	0.57906	0.848425	189
hsa04060	Cytokine-cytokine receptor interaction	238	0.110792	0.937667	0.47037	0.849908	190
hsa00565	Ether lipid metabolism	35	0.118936	0.780669	0.730924	0.850025	191
hsa05020	Prion diseases	36	0.149248	0.931831	0.512048	0.85061	192
hsa00750	Vitamin B6 metabolism	6	0.291723	0.958882	0.51797	0.851229	193
hsa04152	AMPK signaling pathway	117	0.09983	0.947104	0.492693	0.852393	194
hsa00601	Glycosphingolipid biosynthesis - lacto and neolacto series	24	0.203865	0.952701	0.480734	0.852464	195
hsa00592	alpha-Linolenic acid	22	0.178012	0.941178	0.510978	0.853434	196
hsa04012	ErbB signaling pathway	85	0.097132	0.768167	0.671233	0.854181	197
hsa05203	Viral carcinogenesis	189	-0.24672	-2.08685	0.008639	0.854283	198
hsa05330	Allograft rejection	34	-0.23596	-0.82889	0.578158	0.85553	199
hsa00310	Lysine degradation	41	0.145744	0.770624	0.692308	0.858118	200
hsa00410	beta-Alanine metabolism	29	0.268043	1.501765	0.102881	0.859084	201
hsa00532	Glycosaminoglycan biosynthesis - chondroitin sulfate / dermatan sulfate	15	-0.1854	-0.80941	0.692308	0.861872	202
hsa00340	Histidine metabolism	26	0.279194	1.594987	0.035565	0.873102	203
hsa05164	Influenza A	158	-0.15346	-1.458	0.180556	0.876316	204
hsa04919	Thyroid hormone signaling pathway	115	-0.07858	-0.78857	0.642276	0.877704	205
hsa04330	Notch signaling pathway	45	-0.11034	-0.79307	0.666667	0.878796	206
hsa04140	Regulation of autophagy	35	-0.11538	-0.78182	0.744856	0.879563	207
hsa00260	Glycine, serine and threonine metabolism	38	0.236371	1.50831	0.093555	0.895181	208
hsa05140	Leishmaniasis	63	-0.13729	-0.76012	0.621181	0.8976	209
hsa00450	Selenocompound metabolism	16	0.153262	0.730821	0.794872	0.902077	210
hsa00920	Sulfur metabolism	10	-0.2049	-0.76171	0.748538	0.903772	211
hsa05146	Amoebiasis	103	0.080934	0.702355	0.751004	0.904516	212
hsa00900	Terpenoid backbone biosynthesis	20	-0.16086	-0.74801	0.745833	0.90826	213
hsa05214	Glioma	63	0.088502	0.704862	0.805221	0.908429	214
hsa00010	Glycolysis / Gluconeogenesis	63	0.095018	0.688852	0.798755	0.908753	215
hsa05322	Systemic lupus erythematosus	113	-0.31106	-1.46433	0.145791	0.90941	216
hsa04668	TNF signaling pathway	105	0.090905	0.709567	0.710317	0.909655	217
hsa05410	Hypertrophic cardiomyopathy (HCM)	83	0.081294	0.716917	0.773389	0.914357	218
hsa00130	Ubiquinone and other terpenoid-quinone biosynthesis	10	0.192148	0.678998	0.848485	0.91456	219
hsa00512	Mucin type O-Glycan biosynthesis	25	0.117612	0.710898	0.836066	0.915216	220
hsa05014	Amyotrophic lateral sclerosis (ALS)	51	0.084525	0.689532	0.847599	0.915222	221
hsa05144	Malaria	44	0.134417	0.659139	0.768924	0.917921	222
hsa00460	Cyanoamino acid metabolism	5	0.247751	0.66331	0.876426	0.920246	223
hsa04640	Hematopoietic cell lineage	79	0.110941	0.669015	0.722533	0.920348	224
hsa05210	Colorectal cancer	62	-0.09155	-0.72161	0.75	0.922399	225

hsa04973	Carbohydrate digestion and absorption	39	-0.09673	-0.70332	0.836032	0.924158	226
hsa00780	Biotin metabolism	3	-0.31481	-0.72217	0.826336	0.930183	227
hsa00052	Galactose metabolism	29	-0.12138	-0.70367	0.819957	0.931863	228
hsa05134	Legionellosis	53	-0.09574	-0.68019	0.809816	0.931936	229
hsa00640	Propanoate metabolism	30	0.364462	1.534848	0.113445	0.931974	230
hsa04932	Non-alcoholic fatty liver disease (NAFLD)	141	-0.08646	-0.68544	0.725528	0.932983	231
hsa00564	Glycerophospholipid metabolism	75	-0.07954	-0.72537	0.810445	0.934049	232
hsa04915	Estrogen signaling pathway	96	-0.07655	-0.70506	0.753968	0.938444	233
hsa04066	HIF-1 signaling pathway	102	-0.0751	-0.6873	0.788618	0.938563	234
hsa00380	Tryptophan metabolism	35	0.269895	1.513168	0.104938	0.941795	235
hsa04670	Leukocyte transendothelial migration	106	0.075297	0.632529	0.793037	0.943038	236
hsa04750	Inflammatory mediator regulation of TRP channels	90	0.180971	1.652588	0.043825	0.94704	237
hsa04130	SNARE interactions in vesicular transport	31	-0.11225	-0.65264	0.866157	0.951098	238
hsa04611	Platelet activation	121	-0.07252	-0.65597	0.794059	0.955167	239
hsa00670	One carbon pool by folate	18	-0.12153	-0.62266	0.929134	0.955602	240
hsa00860	Porphyrin and chlorophyll metabolism	35	-0.10895	-0.63589	0.907258	0.955961	241
hsa04974	Protein digestion and absorption	78	0.076351	0.607959	0.87747	0.955966	242
hsa05321	Inflammatory bowel disease (IBD)	62	0.111586	0.611937	0.791016	0.958859	243
hsa04062	Chemokine signaling pathway	175	-0.06511	-0.63781	0.777551	0.961398	244
hsa00030	Pentose phosphate pathway	26	-0.11281	-0.62443	0.903808	0.961789	245
hsa04010	MAPK signaling pathway	244	0.044522	0.582956	0.844211	0.967407	246
hsa04910	Insulin signaling pathway	133	0.0674	0.574715	0.848548	0.968383	247
hsa04912	GnRH signaling pathway	89	0.060132	0.56637	0.913215	0.969079	248
hsa04340	Hedgehog signaling pathway	51	0.071086	0.588263	0.942688	0.969191	249
hsa00980	Metabolism of xenobiotics by cytochrome P450	62	0.305665	1.800713	0.045267	0.969227	250
hsa05412	Arrhythmogenic right ventricular cardiomyopathy	74	0.070225	0.55579	0.936975	0.9711	251
hsa04961	Endocrine and other factor-regulated calcium reabsorption	45	0.059711	0.445407	0.998028	0.976317	252
hsa05133	Pertussis	68	0.06874	0.454715	0.963671	0.979213	253
hsa04070	Phosphatidylinositol signaling system	76	0.053992	0.468438	0.97996	0.980211	254
hsa04930	Type II diabetes mellitus	45	0.068107	0.494093	0.978814	0.980301	255
hsa04975	Fat digestion and absorption	39	0.086362	0.530826	0.940206	0.983929	256
hsa00604	Glycosphingolipid biosynthesis - ganglio series	15	0.107051	0.474532	0.998051	0.984151	257
hsa04725	Cholinergic synapse	109	0.045579	0.495739	0.976987	0.986241	258
hsa00790	Folate biosynthesis	14	0.120819	0.505237	0.983333	0.987729	259
hsa05132	Salmonella infection	79	-0.05967	-0.45168	0.959839	0.988198	260
hsa00982	Drug metabolism - cytochrome P450	57	0.361924	1.939595	0.036364	0.990457	261
hsa04621	NOD-like receptor signaling pathway	52	-0.06904	-0.46028	0.977413	0.992039	262

hsa05142	Chagas disease (American trypanosomiasis)	99	0.062724	0.508621	0.933198	0.992456	263
hsa04210	Apoptosis	83	-0.04557	-0.37786	0.995951	0.992916	264
hsa04380	Osteoclast differentiation	126	-0.07048	-0.47101	0.906931	0.994488	265
hsa04978	Mineral absorption	49	0.249861	1.538158	0.068571	0.996215	266
hsa04722	Neurotrophin signaling pathway	117	-0.04303	-0.38957	0.997988	0.998358	267
hsa00053	Ascorbate and aldarate metabolism	19	0.427953	1.711912	0.04499	1	268
hsa00071	Fatty acid degradation	40	0.356557	1.663568	0.08	1	268
hsa00562	Inositol phosphate metabolism	57	-0.07561	-0.55198	0.941414	1	268
hsa00590	Arachidonic acid metabolism	55	0.245164	1.694354	0.051485	1	268
hsa00730	Thiamine metabolism	3	-0.23171	-0.52175	0.990119	1	268
hsa00830	Retinol metabolism	51	0.363389	1.99425	0.010438	1	268
hsa03022	Basal transcription factors	40	-0.0815	-0.53131	0.974659	1	268
hsa04122	Sulfur relay system	8	-0.15135	-0.47316	0.984962	1	268
hsa04260	Cardiac muscle contraction	70	-0.06894	-0.54779	0.918489	1	268
hsa04514	Cell adhesion molecules (CAMs)	131	-0.06145	-0.48682	0.91828	1	268
hsa04672	Intestinal immune network for IgA production	42	-0.10755	-0.48785	0.90364	1	268
hsa04962	Vasopressin-regulated water reabsorption	44	-0.07963	-0.53157	0.943715	1	268
hsa05145	Toxoplasmosis	109	-0.05912	-0.50746	0.94012	1	268
hsa05150	Staphylococcus aureus infection	44	-0.13132	-0.48695	0.887029	1	268
