# Supplementary material for: A network-based pathway-extending approach using DNA methylation and gene expression data to identify altered pathways
Source: Sci Rep. 2019 Aug 14;9:11853. doi: 10.1038/s41598-019-48372-1 (PMC6694157; doi:10.1038/s41598-019-48372-1)
Supplement: Supplementary file 3 — Supplementary Table S3 [file 41598_2019_48372_MOESM3_ESM.pdf]

A network-based pathway-extending approach using DNA methylation and gene expression data to identify altered pathways

Jie Li[1], Qiaosheng Zhang[1,2,*], Zhuo Chen[1], Dechen Xu[1], and Yadong Wang[1]

[1]Harbin Institute of Technology, School of Computer Science and Technology, Harbin, 150001, P.R. China

[2]Heilongjiang Bayi Agricultural University, College of Science, Daqing, 163319, P.R. China

[*]zqs@hit.edu.cn

All results in LUAD dataset by EP-ORA

Pathway ID	Pathway Name	Universe.Size	Gene.Set.Size	Total.Hits	Expected.Hits	Observed.Hits	Pvalue	Adjusted.Pvalue	Rank
hsa03030	DNA replication	17589	94	8410	44.94514	70	1.20E-07	3.37E-05	1
hsa04976	Bile secretion	17589	136	8410	65.02701	88	5.04E-05	6.13E-03	2
hsa03008	Ribosome biogenesis in eukaryotes	17589	180	8410	86.06515	112	6.57E-05	6.13E-03	2
hsa04110	Cell cycle	17589	315	8410	150.614	180	0.000504	0.035295	4
hsa03013	RNA transport	17589	367	8410	175.4773	206	0.000762	0.042657	5
hsa04810	Regulation of actin cytoskeleton	17589	503	8410	240.5043	274	0.001408	0.065729	6
hsa05166	HTLV-I infection	17589	655	8410	313.1815	348	0.003124	0.124963	7
hsa03430	Mismatch repair	17589	61	8410	29.16652	40	0.00386	0.135087	8
hsa03440	Homologous recombination	17589	62	8410	29.64466	40	0.005897	0.179185	9
hsa05202	Transcriptional misregulation in cancer	17589	419	8410	200.3406	226	0.006399	0.179185	9
hsa04974	Protein digestion and absorption	17589	147	8410	70.28654	85	0.009204	0.234287	11
hsa00970	Aminoacyl-tRNA biosynthesis	17589	123	8410	58.81119	72	0.010739	0.240158	12
hsa03040	Spliceosome	17589	288	8410	137.7042	157	0.012717	0.240158	12
hsa05134	Legionellosis	17589	166	8410	79.3712	94	0.013705	0.240158	12
hsa00450	Selenocompound metabolism	17589	41	8410	19.60373	27	0.015139	0.240158	12
hsa00512	Mucin type O-Glycan biosynthesis	17589	52	8410	24.86327	33	0.016665	0.240158	12
hsa05203	Viral carcinogenesis	17589	526	8410	251.5015	276	0.01676	0.240158	12
hsa05140	Leishmaniasis	17589	169	8410	80.80562	95	0.017055	0.240158	12
hsa05211	Renal cell carcinoma	17589	175	8410	83.67446	98	0.017761	0.240158	12
hsa00860	Porphyrin and chlorophyll metabolism	17589	65	8410	31.07908	40	0.017961	0.240158	12
hsa03015	mRNA surveillance pathway	17589	210	8410	100.4093	116	0.018012	0.240158	12
hsa00510	N-Glycan biosynthesis	17589	120	8410	57.37677	69	0.020676	0.263154	22
hsa04970	Salivary secretion	17589	180	8410	86.06515	100	0.021977	0.267551	23
hsa00533	Glycosaminoglycan biosynthesis - keratan sulfate	17589	28	8410	13.38791	19	0.025954	0.291554	24
hsa04114	Oocyte meiosis	17589	281	8410	134.3573	151	0.026032	0.291554	24
hsa04914	Progesterone-mediated oocyte maturation	17589	236	8410	112.841	128	0.027272	0.293701	26
hsa05020	Prion diseases	17589	93	8410	44.467	54	0.030017	0.309511	27
hsa04380	Osteoclast differentiation	17589	326	8410	155.8736	173	0.031444	0.309511	27
hsa05146	Amoebiasis	17589	259	8410	123.8382	139	0.03313	0.309511	27
hsa03460	Fanconi anemia pathway	17589	103	8410	49.24839	59	0.033597	0.309511	27
hsa05143	African trypanosomiasis	17589	105	8410	50.20467	60	0.034267	0.309511	27
hsa00140	Steroid hormone biosynthesis	17589	71	8410	33.94792	42	0.036033	0.312151	32
hsa04978	Mineral absorption	17589	113	8410	54.02979	64	0.0368	0.312151	32
hsa04512	ECM-receptor interaction	17589	189	8410	90.36841	103	0.037904	0.312151	32

hsa05150	Staphylococcus aureus infection	17589	98	8410	46.8577	56	0.039837	0.312181	35
hsa05169	Epstein-Barr virus	17589	537	8410	256.761	277	0.04171	0.312181	35
hsa05205	Proteoglycans in cancer	17589	521	8410	249.1108	269	0.042209	0.312181	35
hsa04726	Serotonergic synapse	17589	241	8410	115.2317	129	0.042518	0.312181	35
hsa05322	Systemic lupus erythematosus	17589	182	8410	87.02143	99	0.043482	0.312181	35
hsa05200	Pathways in cancer	17589	843	8410	403.0718	427	0.048965	0.331254	40
hsa00750	Vitamin B6 metabolism	17589	17	8410	8.128376	12	0.049968	0.331254	40
hsa02010	ABC transporters	17589	101	8410	48.29211	57	0.050552	0.331254	40
hsa04611	Platelet activation	17589	340	8410	162.5675	178	0.050871	0.331254	40
hsa05100	Bacterial invasion of epithelial cells	17589	201	8410	96.10609	108	0.052878	0.336499	44
hsa00061	Fatty acid biosynthesis	17589	19	8410	9.084655	13	0.057704	0.359045	45
hsa05133	Pertussis	17589	202	8410	96.58423	108	0.061064	0.371696	46
hsa05132	Salmonella infection	17589	226	8410	108.0596	120	0.062662	0.372969	47
hsa05131	Shigellosis	17589	177	8410	84.63074	95	0.067836	0.372969	47
hsa04144	Endocytosis	17589	494	8410	236.201	253	0.068291	0.372969	47
hsa04510	Focal adhesion	17589	492	8410	235.2448	252	0.068432	0.372969	47
hsa00230	Purine metabolism	17589	381	8410	182.1712	197	0.068738	0.372969	47
hsa05414	Dilated cardiomyopathy	17589	197	8410	94.19353	105	0.069726	0.372969	47
hsa04080	Neuroactive ligand-receptor interaction	17589	337	8410	161.1331	175	0.070598	0.372969	47
hsa04145	Phagosome	17589	344	8410	164.4801	178	0.077975	0.391429	54
hsa05210	Colorectal cancer	17589	176	8410	84.1526	94	0.078212	0.391429	54
hsa05206	MicroRNAs in cancer	17589	435	8410	207.9908	223	0.0793	0.391429	54
hsa04012	ErbB signaling pathway	17589	236	8410	112.841	124	0.081057	0.391429	54
hsa04666	Fc gamma R-mediated phagocytosis	17589	240	8410	114.7535	126	0.081082	0.391429	54
hsa00601	Glycosphingolipid biosynthesis - lacto and neolacto series	17589	40	8410	19.12559	24	0.082771	0.392809	59
hsa00650	Butanoate metabolism	17589	63	8410	30.1228	36	0.087137	0.402591	60
hsa00040	Pentose and glucuronate interconversions	17589	44	8410	21.03815	26	0.088748	0.402591	60
hsa05152	Tuberculosis	17589	404	8410	193.1685	207	0.08964	0.402591	60
hsa04640	Hematopoietic cell lineage	17589	187	8410	89.41213	99	0.090583	0.402591	60
hsa04614	Renin-angiotensin system	17589	50	8410	23.90699	29	0.096419	0.418364	64
hsa05030	Cocaine addiction	17589	126	8410	60.24561	68	0.09712	0.418364	64
hsa04270	Vascular smooth muscle contraction	17589	284	8410	135.7917	147	0.099896	0.41845	66
hsa04260	Cardiac muscle contraction	17589	144	8410	68.85212	77	0.100129	0.41845	66
hsa03320	PPAR signaling pathway	17589	170	8410	81.28376	90	0.102513	0.418848	68
hsa04120	Ubiquitin mediated proteolysis	17589	351	8410	167.8271	180	0.103895	0.418848	68
hsa04015	Rap1 signaling pathway	17589	487	8410	232.8541	247	0.104712	0.418848	68
hsa03410	Base excision repair	17589	105	8410	50.20467	57	0.108723	0.428767	71
hsa00072	Synthesis and degradation of ketone bodies	17589	26	8410	12.43163	16	0.113937	0.443088	72
hsa00360	Phenylalanine metabolism	17589	53	8410	25.34141	30	0.126067	0.476489	73
hsa04060	Cytokine-cytokine receptor interaction	17589	463	8410	221.3787	234	0.126575	0.476489	73
hsa00670	One carbon pool by folate	17589	57	8410	27.25397	32	0.129743	0.476489	73

hsa05144	Malaria	17589	126	8410	60.24561	67	0.131504	0.476489	73
hsa04622	RIG-I-like receptor signaling pathway	17589	148	8410	70.76468	78	0.132877	0.476489	73
hsa00983	Drug metabolism - other enzymes	17589	73	8410	34.9042	40	0.140299	0.476489	73
hsa00240	Pyrimidine metabolism	17589	239	8410	114.2754	123	0.141803	0.476489	73
hsa04020	Calcium signaling	17589	373	8410	178.3461	189	0.143715	0.476489	73
hsa05220	Chronic myeloid leukemia	17589	207	8410	98.97493	107	0.146122	0.476489	73
hsa00270	Cysteine and methionine metabolism	17589	93	8410	44.467	50	0.147433	0.476489	73
hsa04977	Vitamin digestion and absorption	17589	44	8410	21.03815	25	0.147773	0.476489	73
hsa00380	Tryptophan metabolism	17589	97	8410	46.37956	52	0.14833	0.476489	73
hsa04664	Fc epsilon RI signaling pathway	17589	183	8410	87.49957	95	0.148858	0.476489	73
hsa05412	Arrhythmogenic right ventricular cardiomyopathy (ARVC)	17589	177	8410	84.63074	92	0.149441	0.476489	73
hsa03420	Nucleotide excision repair	17589	109	8410	52.11723	58	0.150263	0.476489	73
hsa04913	Ovarian steroidogenesis	17589	115	8410	54.98607	61	0.150878	0.476489	73
hsa04610	Complement and coagulation cascades	17589	147	8410	70.28654	77	0.151456	0.476489	73
hsa03060	Protein export	17589	64	8410	30.60094	35	0.164164	0.509247	90
hsa05410	Hypertrophic cardiomyopathy (HCM)	17589	182	8410	87.02143	94	0.166917	0.509247	90
hsa05332	Graft-versus-host disease	17589	80	8410	38.25118	43	0.170246	0.509247	90
hsa04514	Cell adhesion molecules (CAMs)	17589	310	8410	148.2233	157	0.171176	0.509247	90
hsa00051	Fructose and mannose metabolism	17589	88	8410	42.0763	47	0.171954	0.509247	90
hsa00590	Arachidonic acid metabolism	17589	136	8410	65.02701	71	0.17278	0.509247	90
hsa04062	Chemokine signaling pathway	17589	441	8410	210.8596	221	0.175974	0.513256	96
hsa00562	Inositol phosphate metabolism	17589	173	8410	82.71818	89	0.188209	0.531517	97
hsa04310	Wnt signaling pathway	17589	344	8410	164.4801	173	0.190961	0.531517	97
hsa04750	Inflammatory mediator regulation of TRP	17589	236	8410	112.841	120	0.191116	0.531517	97
hsa00400	Phenylalanine, tyrosine and tryptophan	17589	7	8410	3.346978	5	0.192238	0.531517	97
hsa00780	Biotin metabolism	17589	7	8410	3.346978	5	0.192238	0.531517	97
hsa05130	Pathogenic Escherichia coli infection	17589	147	8410	70.28654	76	0.193624	0.531517	97
hsa05219	Bladder cancer	17589	115	8410	54.98607	60	0.198878	0.537873	103
hsa04973	Carbohydrate digestion and absorption	17589	91	8410	43.51072	48	0.200573	0.537873	103
hsa04921	Oxytocin signaling pathway	17589	357	8410	170.6959	179	0.201702	0.537873	103
hsa04261	Adrenergic signaling in cardiomyocytes	17589	337	8410	161.1331	169	0.208575	0.550952	106
hsa04540	Gap junction	17589	223	8410	106.6252	113	0.21393	0.559817	107
hsa04520	Adherens junction	17589	211	8410	100.8875	107	0.21815	0.565575	108

hsa04022	cGMP-PKG signaling pathway	17589	389	8410	185.9964	194	0.220526	0.566488	109
hsa04950	Maturity onset diabetes of the young	17589	40	8410	19.12559	22	0.225809	0.574787	110
hsa04141	Protein processing in endoplasmic reticulum	17589	417	8410	199.3843	207	0.240006	0.602105	111
hsa04725	Cholinergic synapse	17589	249	8410	119.0568	125	0.243262	0.602105	111
hsa04066	HIF-1 signaling pathway	17589	288	8410	137.7042	144	0.245193	0.602105	111
hsa05110	Vibrio cholerae infection	17589	133	8410	63.59259	68	0.247839	0.602105	111
hsa04115	p53 signaling pathway	17589	190	8410	90.84655	96	0.248276	0.602105	111
hsa05416	Viral myocarditis	17589	129	8410	61.68003	66	0.249443	0.602105	111
hsa00480	Glutathione metabolism	17589	115	8410	54.98607	59	0.255067	0.610416	117
hsa04630	Jak-STAT signaling pathway	17589	254	8410	121.4475	127	0.261186	0.619234	118
hsa00020	Citrate cycle (TCA cycle)	17589	89	8410	42.55444	46	0.265257	0.619234	118
hsa00830	Retinol metabolism	17589	85	8410	40.64188	44	0.26674	0.619234	118
hsa00920	Sulfur metabolism	17589	25	8410	11.95349	14	0.267598	0.619234	118
hsa04964	Proximal tubule bicarbonate reclamation	17589	53	8410	25.34141	28	0.27587	0.633143	122
hsa00310	Lysine degradation	17589	116	8410	55.46421	59	0.285432	0.649763	123
hsa05168	Herpes simplex infection	17589	410	8410	196.0373	202	0.292222	0.659857	124
hsa05161	Hepatitis B	17589	386	8410	184.5619	190	0.305295	0.675387	125
hsa00350	Tyrosine metabolism	17589	82	8410	39.20746	42	0.305441	0.675387	125
hsa04530	Tight junction	17589	285	8410	136.2698	141	0.306336	0.675387	125
hsa04151	PI3K-Akt signaling pathway	17589	755	8410	360.9955	368	0.313926	0.68257	128
hsa04010	MAPK signaling pathway	17589	575	8410	274.9304	281	0.318044	0.68257	128
hsa05310	Asthma	17589	60	8410	28.68838	31	0.319192	0.68257	128
hsa04612	Antigen processing and presentation	17589	154	8410	73.63352	77	0.320904	0.68257	128
hsa00563	Glycosylphosphatidylinositol(GPI)-anchor biosynthesis	17589	50	8410	23.90699	26	0.325389	0.68257	128
hsa01040	Biosynthesis of unsaturated fatty acids	17589	46	8410	21.99443	24	0.327782	0.68257	128
hsa04730	Long-term depression	17589	144	8410	68.85212	72	0.328407	0.68257	128
hsa05321	Inflammatory bowel disease (IBD)	17589	140	8410	66.93956	70	0.331519	0.68257	128
hsa00910	Nitrogen metabolism	17589	32	8410	15.30047	17	0.335025	0.68257	128
hsa00053	Ascorbate and aldarate metabolism	17589	32	8410	15.30047	17	0.335025	0.68257	128
hsa00130	Ubiquinone and other terpenoid-quinone biosynthesis	17589	28	8410	13.38791	15	0.336409	0.68257	128
hsa04360	Axon guidance	17589	299	8410	142.9638	147	0.339614	0.684115	139
hsa04070	Phosphatidylinositol signaling system	17589	219	8410	104.7126	108	0.351918	0.703836	140
hsa04320	Dorso-ventral axis formation	17589	67	8410	32.03536	34	0.359403	0.713709	141
hsa00062	Fatty acid elongation	17589	53	8410	25.34141	27	0.374324	0.738104	142
hsa04971	Gastric acid secretion	17589	156	8410	74.5898	77	0.378876	0.741854	143
hsa04140	Regulation of autophagy	17589	84	8410	40.16374	42	0.384471	0.747582	144
hsa05222	Small cell lung cancer	17589	237	8410	113.3191	116	0.387327	0.747942	145

hsa04919	Thyroid hormone signaling pathway	17589	314	8410	150.1359	153	0.39351	0.748407	146
hsa05323	Rheumatoid arthritis	17589	200	8410	95.62795	98	0.394595	0.748407	146
hsa04972	Pancreatic secretion	17589	198	8410	94.67167	97	0.39649	0.748407	146
hsa00010	Glycolysis / Gluconeogenesis	17589	167	8410	79.84934	82	0.398259	0.748407	146
hsa04960	Aldosterone-regulated sodium reabsorption	17589	101	8410	48.29211	50	0.404206	0.754518	150
hsa00603	Glycosphingolipid biosynthesis - globo	17589	27	8410	12.90977	14	0.409213	0.755826	151
hsa04920	Adipocytokine signaling pathway	17589	184	8410	87.97771	90	0.410305	0.755826	151
hsa00900	Terpenoid backbone biosynthesis	17589	60	8410	28.68838	30	0.416195	0.761664	153
hsa04918	Thyroid hormone	17589	158	8410	75.54608	77	0.438941	0.798074	154
hsa04370	VEGF signaling pathway	17589	179	8410	85.58701	87	0.445	0.798367	155
hsa03020	RNA polymerase	17589	71	8410	33.94792	35	0.447105	0.798367	155
hsa00982	Drug metabolism - cytochrome P450	17589	98	8410	46.8577	48	0.447656	0.798367	155
hsa04014	Ras signaling pathway	17589	517	8410	247.1982	249	0.453461	0.799067	158
hsa05213	Endometrial cancer	17589	146	8410	69.8084	71	0.453756	0.799067	158
hsa03018	RNA degradation	17589	163	8410	77.93678	79	0.464222	0.812389	160
hsa05014	Amyotrophic lateral sclerosis (ALS)	17589	134	8410	64.07073	65	0.469821	0.81708	161
hsa00520	Amino sugar and nucleotide sugar metabolism	17589	130	8410	62.15817	63	0.475493	0.82184	162
hsa04068	FoxO signaling pathway	17589	366	8410	174.9991	176	0.478591	0.82212	163
hsa04740	Olfactory transduction	17589	51	8410	24.38513	25	0.486341	0.828401	164
hsa04122	Sulfur relay system	17589	26	8410	12.43163	13	0.488165	0.828401	164
hsa04668	TNF signaling pathway	17589	304	8410	145.3545	146	0.492953	0.831487	166
hsa00770	Pantothenate and CoA biosynthesis	17589	47	8410	22.47257	23	0.495953	0.831537	167
hsa04620	Toll-like receptor signaling pathway	17589	225	8410	107.5814	108	0.503981	0.831777	168
hsa03050	Proteasome	17589	106	8410	50.68281	51	0.513648	0.831777	168
hsa04621	NOD-like receptor signaling pathway	17589	148	8410	70.76468	71	0.516962	0.831777	168
hsa04916	Melanogenesis	17589	236	8410	112.841	113	0.517464	0.831777	168
hsa05142	Chagas disease (American trypanosomiasis)	17589	280	8410	133.8791	134	0.51789	0.831777	168
hsa00532	Glycosaminoglycan biosynthesis - chondroitin sulfate / dermatan sulfate	17589	39	8410	18.64745	19	0.517893	0.831777	168
hsa00591	Linoleic acid metabolism	17589	58	8410	27.73211	28	0.523568	0.831777	168
hsa00410	beta-Alanine metabolism	17589	79	8410	37.77304	38	0.523897	0.831777	168
hsa05217	Basal cell carcinoma	17589	119	8410	56.89863	57	0.528711	0.831777	168
hsa05215	Prostate cancer	17589	247	8410	118.1005	118	0.530333	0.831777	168
hsa04130	SNARE interactions in vesicular transport	17589	75	8410	35.86048	36	0.532574	0.831777	168
hsa04911	Insulin secretion	17589	180	8410	86.06515	86	0.533336	0.831777	168
hsa04623	Cytosolic DNA-sensing pathway	17589	115	8410	54.98607	55	0.53571	0.831777	168
hsa04152	AMPK signaling pathway	17589	304	8410	145.3545	145	0.539087	0.831777	168

hsa04150	mTOR signaling pathway	17589	153	8410	73.15538	73	0.54194	0.831777	168
hsa00564	Glycerophospholipid metabolism	17589	193	8410	92.28097	92	0.544625	0.831777	168
hsa04940	Type I diabetes mellitus	17589	88	8410	42.0763	42	0.548412	0.831777	168
hsa05032	Morphine addiction	17589	168	8410	80.32748	80	0.550634	0.831777	168
hsa00471	D-Glutamine and D-glutamate metabolism	17589	12	8410	5.737677	6	0.552538	0.831777	168
hsa04742	Taste transduction	17589	61	8410	29.16652	29	0.567137	0.849117	187
hsa03450	Non-homologous end-joining	17589	40	8410	19.12559	19	0.577538	0.849117	187
hsa04912	GnRH signaling pathway	17589	222	8410	106.147	105	0.58773	0.849117	187
hsa00500	Starch and sucrose metabolism	17589	89	8410	42.55444	42	0.588098	0.849117	187
hsa00600	Sphingolipid metabolism	17589	89	8410	42.55444	42	0.588098	0.849117	187
hsa00340	Histidine metabolism	17589	53	8410	25.34141	25	0.590746	0.849117	187
hsa05214	Glioma	17589	178	8410	85.10887	84	0.595424	0.849117	187
hsa04650	Natural killer cell mediated cytotoxicity	17589	273	8410	130.5322	129	0.597651	0.849117	187
hsa00790	Folate biosynthesis	17589	34	8410	16.25675	16	0.601443	0.849117	187
hsa04917	Prolactin signaling pathway	17589	189	8410	90.36841	89	0.607369	0.849117	187
hsa00620	Pyruvate metabolism	17589	115	8410	54.98607	54	0.60907	0.849117	187
hsa05218	Melanoma	17589	149	8410	71.24282	70	0.61249	0.849117	187
hsa04340	Hedgehog signaling pathway	17589	113	8410	54.02979	53	0.613166	0.849117	187
hsa00260	Glycine, serine and threonine metabolism	17589	111	8410	53.07351	52	0.61733	0.849117	187
hsa04961	Endocrine and other factor-regulated calcium reabsorption	17589	111	8410	53.07351	52	0.61733	0.849117	187
hsa00460	Cyanoamino acid metabolism	17589	17	8410	8.128376	8	0.618089	0.849117	187
hsa04350	TGF-beta signaling pathway	17589	200	8410	95.62795	94	0.618642	0.849117	187
hsa05120	Epithelial cell signaling in Helicobacter pylori infection	17589	200	8410	95.62795	94	0.618642	0.849117	187
hsa00592	alpha-Linolenic acid metabolism	17589	58	8410	27.73211	27	0.626374	0.855535	205
hsa05204	Chemical carcinogenesis	17589	118	8410	56.42049	55	0.638232	0.862742	206
hsa05320	Autoimmune thyroid disease	17589	69	8410	32.99164	32	0.639933	0.862742	206
hsa05223	Non-small cell lung	17589	167	8410	79.84934	78	0.642279	0.862742	206
hsa05212	Pancreatic cancer	17589	201	8410	96.10609	94	0.643975	0.862742	206
hsa00120	Primary bile acid biosynthesis	17589	37	8410	17.69117	17	0.651587	0.86565	210
hsa00232	Caffeine metabolism	17589	13	8410	6.215817	6	0.652329	0.86565	210
hsa04720	Long-term potentiation	17589	159	8410	76.02422	74	0.655951	0.866351	212
hsa03022	Basal transcription factors	17589	108	8410	51.63909	50	0.659797	0.867338	213
hsa00071	Fatty acid degradation	17589	102	8410	48.77025	47	0.6736	0.878347	214
hsa05160	Hepatitis C	17589	302	8410	144.3982	141	0.674445	0.878347	214
hsa05016	Huntington,s disease	17589	442	8410	211.3378	207	0.679381	0.88068	216
hsa00100	Steroid biosynthesis	17589	42	8410	20.08187	19	0.686774	0.886159	217
hsa04713	Circadian entrainment	17589	220	8410	105.1907	102	0.691609	0.888305	218

hsa04146	Peroxisome	17589	184	8410	87.97771	85	0.696726	0.888366	219
hsa00290	Valine, leucine and isoleucine biosynthesis	17589	9	8410	4.303258	4	0.701285	0.888366	219
hsa05340	Primary immunodeficiency	17589	90	8410	43.03258	41	0.703448	0.888366	219
hsa04064	NF-kappa B signaling pathway	17589	225	8410	107.5814	104	0.707956	0.888366	219
hsa04210	Apoptosis	17589	208	8410	99.45307	96	0.709233	0.888366	219
hsa05164	Influenza A	17589	404	8410	193.1685	188	0.715872	0.888366	219
hsa00250	Alanine, aspartate and glutamate metabolism	17589	84	8410	40.16374	38	0.719645	0.888366	219
hsa00640	Propanoate metabolism	17589	95	8410	45.42328	43	0.725997	0.888366	219
hsa05162	Measles	17589	326	8410	155.8736	151	0.726013	0.888366	219
hsa04915	Estrogen signaling pathway	17589	243	8410	116.188	112	0.727587	0.888366	219
hsa00280	Valine, leucine and isoleucine degradation	17589	121	8410	57.85491	55	0.729595	0.888366	219
hsa05031	Amphetamine addiction	17589	164	8410	78.41492	75	0.730383	0.888366	219
hsa04724	Glutamatergic synapse	17589	237	8410	113.3191	109	0.735732	0.888366	219
hsa00730	Thiamine metabolism	17589	7	8410	3.346978	3	0.736075	0.888366	219
hsa05033	Nicotine addiction	17589	50	8410	23.90699	22	0.751959	0.894853	233
hsa04662	B cell receptor signaling pathway	17589	195	8410	93.23725	89	0.752451	0.894853	233
hsa04390	Hippo signaling pathway	17589	362	8410	173.0866	167	0.757978	0.894853	233
hsa04670	Leukocyte transendothelial migration	17589	251	8410	120.0131	115	0.758373	0.894853	233
hsa00785	Lipoic acid metabolism	17589	12	8410	5.737677	5	0.761226	0.894853	233
hsa04723	Retrograde endocannabinoid	17589	187	8410	89.41213	85	0.764947	0.894853	233
hsa04930	Type II diabetes mellitus	17589	131	8410	62.63631	59	0.765885	0.894853	233
hsa04672	Intestinal immune network for IgA production	17589	92	8410	43.98886	41	0.767017	0.894853	233
hsa04975	Fat digestion and absorption	17589	64	8410	30.60094	28	0.781198	0.907549	241
hsa03010	Ribosome	17589	321	8410	153.4829	147	0.784382	0.907549	241
hsa00630	Glyoxylate and dicarboxylate metabolism	17589	71	8410	33.94792	31	0.793871	0.911183	243
hsa05221	Acute myeloid leukemia	17589	169	8410	80.80562	76	0.794031	0.911183	243
hsa04728	Dopaminergic synapse	17589	307	8410	146.7889	140	0.799505	0.91372	245
hsa00524	Butirosin and neomycin biosynthesis	17589	15	8410	7.172096	6	0.805642	0.916991	246
hsa04727	GABAergic synapse	17589	170	8410	81.28376	76	0.813815	0.922543	247
hsa05010	Alzheimer,s disease	17589	403	8410	192.6903	184	0.823047	0.929247	248
hsa04966	Collecting duct acid secretion	17589	61	8410	29.16652	26	0.826659	0.929576	249
hsa00565	Ether lipid metabolism	17589	95	8410	45.42328	41	0.844708	0.94454	250
hsa05034	Alcoholism	17589	300	8410	143.4419	135	0.851418	0.94454	250
hsa00604	Glycosphingolipid biosynthesis - ganglio series	17589	37	8410	17.69117	15	0.85355	0.94454	250
hsa00514	Other types of O-glycan biosynthesis	17589	62	8410	29.64466	26	0.854507	0.94454	250
hsa00052	Galactose metabolism	17589	80	8410	38.25118	34	0.856832	0.94454	250
hsa04910	Insulin signaling pathway	17589	338	8410	161.6112	152	0.866928	0.948255	255

hsa00430	Taurine and hypotaurine metabolism	17589	21	8410	10.04093	8	0.866976	0.948255	255
hsa00330	Arginine and proline metabolism	17589	141	8410	67.4177	61	0.879336	0.956269	257
hsa04962	Vasopressin-regulated water reabsorption	17589	117	8410	55.94235	50	0.884396	0.956269	257
hsa00740	Riboflavin metabolism	17589	31	8410	14.82233	12	0.884549	0.956269	257
hsa05145	Toxoplasmosis	17589	297	8410	142.0075	132	0.890954	0.958265	260
hsa04330	Notch signaling pathway	17589	122	8410	58.33305	52	0.89324	0.958265	260
hsa00561	Glycerolipid metabolism	17589	109	8410	52.11723	46	0.898713	0.959863	262
hsa04744	Phototransduction	17589	48	8410	22.95071	19	0.901586	0.959863	262
hsa04932	Non-alcoholic fatty liver disease (NAFLD)	17589	366	8410	174.9991	163	0.907004	0.961974	264
hsa04722	Neurotrophin signaling pathway	17589	316	8410	151.0922	139	0.92396	0.9755	265
hsa00980	Metabolism of xenobiotics by cytochrome P450	17589	100	8410	47.81397	41	0.929419	0.9755	265
hsa04710	Circadian rhythm	17589	75	8410	35.86048	30	0.930208	0.9755	265
hsa04721	Synaptic vesicle cycle	17589	132	8410	63.11445	55	0.934413	0.976253	268
hsa05330	Allograft rejection	17589	76	8410	36.33862	30	0.942814	0.980925	269
hsa00531	Glycosaminoglycan degradation	17589	41	8410	19.60373	15	0.945892	0.980925	269
hsa05216	Thyroid cancer	17589	87	8410	41.59816	34	0.959858	0.991735	271
hsa04660	T cell receptor signaling pathway	17589	269	8410	128.6196	114	0.96879	0.99371	272
hsa00760	Nicotinate and nicotinamide metabolism	17589	58	8410	27.73211	21	0.972362	0.99371	272
hsa00511	Other glycan degradation	17589	49	8410	23.42885	17	0.977297	0.99371	272
hsa05012	Parkinson,s disease	17589	306	8410	146.3108	129	0.980333	0.99371	272
hsa00030	Pentose phosphate pathway	17589	79	8410	37.77304	29	0.982423	0.99371	272
hsa00534	Glycosaminoglycan biosynthesis - heparan sulfate / heparin	17589	38	8410	18.16931	12	0.985953	0.99371	272
hsa00190	Oxidative phosphorylation	17589	264	8410	126.2289	108	0.990154	0.99371	272
hsa04142	Lysosome	17589	309	8410	147.7452	128	0.990161	0.99371	272
hsa00300	Lysine biosynthesis	17589	4	8410	1.912559	0	1	1	280

All results in LUAD dataset by ORA

Pathway ID	Pathway Name	Univers e.Size	Gene .Set.S ize	Total.H its	Expected. Hits	Obse rved. Hits	Pvalue	Adjusted. Pvalue	Ran k
hsa03008	Ribosome biogenesis in eukaryotes	17589	70	8410	33.46978	49	0.000139	3.24E-02	1
hsa03030	DNA replication	17589	36	8410	17.21303	28	0.000231	3.24E-02	1
hsa04110	Cell cycle	17589	118	8410	56.42049	75	0.000399	0.037359	3
hsa00970	Aminoacyl-tRNA biosynthesis	17589	43	8410	20.56001	31	0.001069	0.075107	4
hsa00230	Purine metabolism	17589	155	8410	74.11166	92	0.002472	0.138913	5
hsa05322	Systemic lupus erythematosus	17589	97	8410	46.37956	60	0.003689	0.172745	6
hsa05144	Malaria	17589	44	8410	21.03815	30	0.005054	0.202879	7
hsa03013	RNA transport	17589	148	8410	70.76468	86	0.007428	0.260906	8
hsa03430	Mismatch repair	17589	23	8410	10.99721	17	0.010105	0.265207	9

hsa05150	Staphylococcus aureus infection	17589	49	8410	23.42885	32	0.010181	0.265207	9
hsa05166	HTLV-I infection	17589	252	8410	120.4912	139	0.011103	0.265207	9
hsa00350	Tyrosine metabolism	17589	37	8410	17.69117	25	0.012092	0.265207	9
hsa03460	Fanconi anemia pathway	17589	46	8410	21.99443	30	0.013017	0.265207	9
hsa03450	Non-homologous end-joining	17589	12	8410	5.737677	10	0.013213	0.265207	9
hsa00360	Phenylalanine metabolism	17589	17	8410	8.128376	13	0.015812	0.277717	15
hsa05203	Viral carcinogenesis	17589	186	8410	88.93399	104	0.015813	0.277717	15
hsa05140	Leishmaniasis	17589	69	8410	32.99164	42	0.019849	0.328089	17
hsa00510	N-Glycan biosynthesis	17589	49	8410	23.42885	31	0.021214	0.33117	18
hsa00400	Phenylalanine, tyrosine and tryptophan	17589	5	8410	2.390699	5	0.024975	0.369366	19
hsa05206	MicroRNAs in cancer	17589	149	8410	71.24282	83	0.031892	0.442127	20
hsa05412	Arrhythmogenic right ventricular cardiomyopathy (ARVC)	17589	69	8410	32.99164	41	0.034835	0.442127	20
hsa05020	Prion diseases	17589	34	8410	16.25675	22	0.035448	0.442127	20
hsa03040	Spliceosome	17589	111	8410	53.07351	63	0.036188	0.442127	20
hsa04145	Phagosome	17589	143	8410	68.37398	79	0.044408	0.519945	24
hsa04270	Vascular smooth muscle contraction	17589	111	8410	53.07351	62	0.054145	0.572743	25
hsa03440	Homologous recombination	17589	26	8410	12.43163	17	0.054596	0.572743	25
hsa05219	Bladder cancer	17589	37	8410	17.69117	23	0.05637	0.572743	25
hsa05200	Pathways in cancer	17589	313	8410	149.6577	164	0.05707	0.572743	25
hsa04974	Protein digestion and absorption	17589	75	8410	35.86048	43	0.062013	0.600886	29
hsa05202	Transcriptional misregulation in cancer	17589	159	8410	76.02422	86	0.065423	0.612798	30
hsa00240	Pyrimidine metabolism	17589	99	8410	47.33583	55	0.074198	0.670024	31
hsa04614	Renin-angiotensin system	17589	16	8410	7.650236	11	0.076302	0.670024	31
hsa00750	Vitamin B6 metabolism	17589	6	8410	2.868838	5	0.090161	0.767732	33
hsa04512	ECM-receptor interaction	17589	85	8410	40.64188	47	0.101175	0.805224	34
hsa00780	Biotin metabolism	17589	3	8410	1.434419	3	0.109291	0.805224	34
hsa04611	Platelet activation	17589	127	8410	60.72375	68	0.113547	0.805224	34
hsa05034	Alcoholism	17589	139	8410	66.46142	74	0.115133	0.805224	34
hsa05410	Hypertrophic cardiomyopathy (HCM)	17589	74	8410	35.38234	41	0.116372	0.805224	34
hsa04141	Protein processing in endoplasmic reticulum	17589	159	8410	76.02422	84	0.116621	0.805224	34
hsa03420	Nucleotide excision repair	17589	45	8410	21.51629	26	0.116957	0.805224	34
hsa04970	Salivary secretion	17589	76	8410	36.33862	42	0.117488	0.805224	34
hsa05132	Salmonella infection	17589	82	8410	39.20746	45	0.120475	0.806037	42
hsa05161	Hepatitis B	17589	130	8410	62.15817	69	0.131892	0.828635	43
hsa05143	African trypanosomiasis	17589	34	8410	16.25675	20	0.132523	0.828635	43
hsa04610	Complement and coagulation cascades	17589	61	8410	29.16652	34	0.132945	0.828635	43
hsa00130	Ubiquinone and other terpenoid-quinone biosynthesis	17589	10	8410	4.781397	7	0.138288	0.828635	43
hsa05133	Pertussis	17589	71	8410	33.94792	39	0.139272	0.828635	43
hsa05414	Dilated cardiomyopathy	17589	81	8410	38.72932	44	0.143769	0.828635	43

hsa03015	mRNA surveillance pathway	17589	83	8410	39.6856	45	0.144495	0.828635	43
hsa00601	Glycosphingolipid biosynthesis - lacto and neolacto series	17589	25	8410	11.95349	15	0.153821	0.840694	50
hsa05130	Pathogenic Escherichia coli infection	17589	52	8410	24.86327	29	0.155991	0.840694	50
hsa04621	NOD-like receptor signaling pathway	17589	54	8410	25.81955	30	0.157649	0.840694	50
hsa00860	Porphyrin and chlorophyll metabolism	17589	27	8410	12.90977	16	0.158998	0.840694	50
hsa04810	Regulation of actin cytoskeleton	17589	194	8410	92.75911	100	0.164959	0.840694	50
hsa00533	Glycosaminoglycan biosynthesis - keratan sulfate	17589	14	8410	6.693956	9	0.166998	0.840694	50
hsa04261	Adrenergic signaling in cardiomyocytes	17589	136	8410	65.02701	71	0.17278	0.840694	50
hsa04380	Osteoclast differentiation	17589	128	8410	61.20189	67	0.173356	0.840694	50
hsa04114	Oocyte meiosis	17589	102	8410	48.77025	54	0.173524	0.840694	50
hsa04950	Maturity onset diabetes of the young	17589	16	8410	7.650236	10	0.177303	0.844443	59
hsa03050	Proteasome	17589	43	8410	20.56001	24	0.184405	0.858168	60
hsa04978	Mineral absorption	17589	45	8410	21.51629	25	0.186293	0.858168	60
hsa05321	Inflammatory bowel disease (IBD)	17589	57	8410	27.25397	31	0.194269	0.880476	62
hsa04520	Adherens junction	17589	73	8410	34.9042	39	0.199201	0.882229	63
hsa04510	Focal adhesion	17589	202	8410	96.58423	103	0.200935	0.882229	63
hsa00512	Mucin type O-Glycan biosynthesis	17589	26	8410	12.43163	15	0.208209	0.900104	65
hsa04151	PI3K-Akt signaling pathway	17589	305	8410	145.8326	153	0.220283	0.91098	66
hsa04120	Ubiquitin mediated proteolysis	17589	134	8410	64.07073	69	0.220874	0.91098	66
hsa04666	Fc gamma R-mediated phagocytosis	17589	90	8410	43.03258	47	0.231484	0.91098	66
hsa05416	Viral myocarditis	17589	54	8410	25.81955	29	0.232166	0.91098	66
hsa04914	Progesterone-mediated oocyte maturation	17589	82	8410	39.20746	43	0.232695	0.91098	66
hsa04640	Hematopoietic cell lineage	17589	80	8410	38.25118	42	0.232935	0.91098	66
hsa05210	Colorectal cancer	17589	62	8410	29.64466	33	0.233418	0.91098	66
hsa04022	cGMP-PKG signaling pathway	17589	155	8410	74.11166	79	0.239115	0.913641	73
hsa04514	Cell adhesion molecules (CAMs)	17589	135	8410	64.54887	69	0.247039	0.913641	73
hsa00900	Terpenoid backbone biosynthesis	17589	21	8410	10.04093	12	0.261716	0.913641	73
hsa03060	Protein export	17589	23	8410	10.99721	13	0.264969	0.913641	73
hsa00051	Fructose and mannose metabolism	17589	31	8410	14.82233	17	0.272808	0.913641	73
hsa00983	Drug metabolism - other enzymes	17589	31	8410	14.82233	17	0.272808	0.913641	73
hsa03020	RNA polymerase	17589	31	8410	14.82233	17	0.272808	0.913641	73

hsa00250	Alanine, aspartate and glutamate metabolism	17589	33	8410	15.77861	18	0.273897	0.913641	73
hsa03410	Base excision repair	17589	33	8410	15.77861	18	0.273897	0.913641	73
hsa04976	Bile secretion	17589	61	8410	29.16652	32	0.274314	0.913641	73
hsa05332	Graft-versus-host disease	17589	37	8410	17.69117	20	0.275389	0.913641	73
hsa00330	Arginine and proline metabolism	17589	55	8410	26.29769	29	0.275555	0.913641	73
hsa02010	ABC transporters	17589	43	8410	20.56001	23	0.276368	0.913641	73
hsa05205	Proteoglycans in cancer	17589	208	8410	99.45307	104	0.285825	0.933916	86
hsa04080	Neuroactive ligand-receptor interaction	17589	198	8410	94.67167	99	0.291742	0.942292	87
hsa00061	Fatty acid biosynthesis	17589	6	8410	2.868838	4	0.303678	0.947502	88
hsa05100	Bacterial invasion of epithelial cells	17589	74	8410	35.38234	38	0.310401	0.947502	88
hsa04630	Jak-STAT signaling pathway	17589	123	8410	58.81119	62	0.312846	0.947502	88
hsa04115	p53 signaling pathway	17589	66	8410	31.55722	34	0.315419	0.947502	88
hsa05211	Renal cell carcinoma	17589	66	8410	31.55722	34	0.315419	0.947502	88
hsa04260	Cardiac muscle contraction	17589	62	8410	29.64466	32	0.317935	0.947502	88
hsa00053	Ascorbate and aldarate metabolism	17589	12	8410	5.737677	7	0.329452	0.947502	88
hsa00140	Steroid hormone biosynthesis	17589	36	8410	17.21303	19	0.333234	0.947502	88
hsa00450	Selenocompound metabolism	17589	16	8410	7.650236	9	0.33485	0.947502	88
hsa00040	Pentose and glucuronate interconversions	17589	18	8410	8.606515	10	0.336161	0.947502	88
hsa04977	Vitamin digestion and absorption	17589	20	8410	9.562795	11	0.33689	0.947502	88
hsa00650	Butanoate metabolism	17589	22	8410	10.51907	12	0.337189	0.947502	88
hsa04320	Dorso-ventral axis formation	17589	22	8410	10.51907	12	0.337189	0.947502	88
hsa04540	Gap junction	17589	83	8410	39.6856	42	0.344342	0.953607	101
hsa04530	Tight junction	17589	120	8410	57.37677	60	0.348179	0.953607	101
hsa05168	Herpes simplex infection	17589	155	8410	74.11166	77	0.349543	0.953607	101
hsa04066	HIF-1 signaling pathway	17589	102	8410	48.77025	51	0.36509	0.983178	104
hsa04015	Rap1 signaling pathway	17589	197	8410	94.19353	97	0.370088	0.983178	104
hsa00590	Arachidonic acid metabolism	17589	55	8410	26.29769	28	0.37207	0.983178	104
hsa00480	Glutathione metabolism	17589	47	8410	22.47257	24	0.381375	0.983178	104
hsa00520	Amino sugar and nucleotide sugar metabolism	17589	47	8410	22.47257	24	0.381375	0.983178	104
hsa04330	Notch signaling pathway	17589	47	8410	22.47257	24	0.381375	0.983178	104
hsa00270	Cysteine and methionine metabolism	17589	37	8410	17.69117	19	0.394293	0.998165	110
hsa04960	Aldosterone-regulated sodium reabsorption	17589	37	8410	17.69117	19	0.394293	0.998165	110
hsa03320	PPAR signaling pathway	17589	62	8410	29.64466	31	0.413196	1	112
hsa00062	Fatty acid elongation	17589	21	8410	10.04093	11	0.419582	1	112
hsa04152	AMPK signaling pathway	17589	118	8410	56.42049	58	0.420443	1	112
hsa05217	Basal cell carcinoma	17589	54	8410	25.81955	27	0.425713	1	112
hsa04668	TNF signaling pathway	17589	110	8410	52.59537	54	0.430765	1	112

hsa00604	Glycosphingolipid biosynthesis - ganglio series	17589	15	8410	7.172096	8	0.431591	1	112
hsa00790	Folate biosynthesis	17589	13	8410	6.215817	7	0.436115	1	112
hsa00072	Synthesis and degradation of ketone bodies	17589	9	8410	4.303258	5	0.446328	1	112
hsa04360	Axon guidance	17589	123	8410	58.81119	60	0.449876	1	112
hsa04612	Antigen processing and presentation	17589	67	8410	32.03536	33	0.454035	1	112
hsa04917	Prolactin signaling pathway	17589	67	8410	32.03536	33	0.454035	1	112
hsa00232	Caffeine metabolism	17589	5	8410	2.390699	3	0.45906	1	112
hsa04973	Carbohydrate digestion and absorption	17589	36	8410	17.21303	18	0.460947	1	112
hsa04130	SNARE interactions in vesicular transport	17589	34	8410	16.25675	17	0.465776	1	112
hsa00730	Thiamine metabolism	17589	3	8410	1.434419	2	0.467229	1	112
hsa00472	D-Arginine and D-ornithine metabolism	17589	1	8410	0.47814	1	0.47814	1	112
hsa05310	Asthma	17589	24	8410	11.47535	12	0.494787	1	112
hsa05212	Pancreatic cancer	17589	66	8410	31.55722	32	0.504912	1	112
hsa05010	Alzheimer,s disease	17589	154	8410	73.63352	74	0.508162	1	112
hsa00532	Glycosaminoglycan biosynthesis - chondroitin sulfate / dermatan sulfate	17589	20	8410	9.562795	10	0.509843	1	112
hsa04725	Cholinergic synapse	17589	106	8410	50.68281	51	0.513648	1	112
hsa04940	Type I diabetes mellitus	17589	39	8410	18.64745	19	0.517893	1	112
hsa04150	mTOR signaling pathway	17589	58	8410	27.73211	28	0.523568	1	112
hsa00260	Glycine, serine and threonine metabolism	17589	37	8410	17.69117	18	0.524092	1	112
hsa05162	Measles	17589	119	8410	56.89863	57	0.528711	1	112
hsa04911	Insulin secretion	17589	75	8410	35.86048	36	0.532574	1	112
hsa05134	Legionellosis	17589	54	8410	25.81955	26	0.533898	1	112
hsa04919	Thyroid hormone signaling pathway	17589	115	8410	54.98607	55	0.53571	1	112
hsa00603	Glycosphingolipid biosynthesis - globo	17589	14	8410	6.693956	7	0.539498	1	112
hsa00020	Citrate cycle (TCA cycle)	17589	29	8410	13.86605	14	0.552941	1	112
hsa00410	beta-Alanine metabolism	17589	29	8410	13.86605	14	0.552941	1	112
hsa05216	Thyroid cancer	17589	29	8410	13.86605	14	0.552941	1	112
hsa04921	Oxytocin signaling pathway	17589	145	8410	69.33026	69	0.554622	1	112
hsa04920	Adipocytokine signaling pathway	17589	65	8410	31.07908	31	0.556499	1	112
hsa05030	Cocaine addiction	17589	46	8410	21.99443	22	0.557149	1	112
hsa04975	Fat digestion and absorption	17589	27	8410	12.90977	13	0.561477	1	112
hsa05218	Melanoma	17589	61	8410	29.16652	29	0.567137	1	112
hsa04122	Sulfur relay system	17589	10	8410	4.781397	5	0.568237	1	112
hsa04930	Type II diabetes mellitus	17589	42	8410	20.08187	20	0.570405	1	112
hsa00563	Glycosylphosphatidylinositol(GPI)-anchor biosynthesis	17589	25	8410	11.95349	12	0.570722	1	112
hsa05131	Shigellosis	17589	59	8410	28.21025	28	0.572728	1	112

hsa00380	Tryptophan metabolism	17589	38	8410	18.16931	18	0.585057	1	112
hsa04750	Inflammatory mediator regulation of TRP	17589	89	8410	42.55444	42	0.588098	1	112
hsa00982	Drug metabolism - cytochrome P450	17589	51	8410	24.38513	24	0.597223	1	112
hsa05222	Small cell lung cancer	17589	85	8410	40.64188	40	0.597481	1	112
hsa05169	Epstein-Barr virus	17589	195	8410	93.23725	92	0.598467	1	112
hsa04918	Thyroid hormone	17589	66	8410	31.55722	31	0.60219	1	112
hsa00564	Glycerophospholipid metabolism	17589	83	8410	39.6856	39	0.602337	1	112
hsa05323	Rheumatoid arthritis	17589	83	8410	39.6856	39	0.602337	1	112
hsa00670	One carbon pool by folate	17589	19	8410	9.084655	9	0.604225	1	112
hsa01040	Biosynthesis of unsaturated fatty acids	17589	19	8410	9.084655	9	0.604225	1	112
hsa04390	Hippo signaling pathway	17589	147	8410	70.28654	69	0.615997	1	112
hsa00770	Pantothenate and CoA biosynthesis	17589	17	8410	8.128376	8	0.618089	1	112
hsa05220	Chronic myeloid leukemia	17589	73	8410	34.9042	34	0.628481	1	112
hsa05223	Non-small cell lung	17589	56	8410	26.77583	26	0.632944	1	112
hsa00120	Primary bile acid biosynthesis	17589	15	8410	7.172096	7	0.633916	1	112
hsa00910	Nitrogen metabolism	17589	15	8410	7.172096	7	0.633916	1	112
hsa00071	Fatty acid degradation	17589	41	8410	19.60373	19	0.63413	1	112
hsa04144	Endocytosis	17589	197	8410	94.19353	92	0.650007	1	112
hsa04350	TGF-beta signaling pathway	17589	80	8410	38.25118	37	0.652144	1	112
hsa04972	Pancreatic secretion	17589	76	8410	36.33862	35	0.663256	1	112
hsa04964	Proximal tubule bicarbonate reclamation	17589	22	8410	10.51907	10	0.666837	1	112
hsa00830	Retinol metabolism	17589	46	8410	21.99443	21	0.669767	1	112
hsa04012	ErbB signaling pathway	17589	87	8410	41.59816	40	0.67359	1	112
hsa04713	Circadian entrainment	17589	87	8410	41.59816	40	0.67359	1	112
hsa00310	Lysine degradation	17589	42	8410	20.08187	19	0.686774	1	112
hsa04062	Chemokine signaling pathway	17589	182	8410	87.02143	84	0.699976	1	112
hsa05110	Vibrio cholerae infection	17589	51	8410	24.38513	23	0.700933	1	112
hsa04310	Wnt signaling pathway	17589	133	8410	63.59259	61	0.704623	1	112
hsa00600	Sphingolipid metabolism	17589	38	8410	18.16931	17	0.705444	1	112
hsa00620	Pyruvate metabolism	17589	38	8410	18.16931	17	0.705444	1	112
hsa00340	Histidine metabolism	17589	27	8410	12.90977	12	0.705493	1	112
hsa00562	Inositol phosphate metabolism	17589	60	8410	28.68838	27	0.713864	1	112
hsa04623	Cytosolic DNA-sensing pathway	17589	47	8410	22.47257	21	0.71723	1	112
hsa03018	RNA degradation	17589	71	8410	33.94792	32	0.719431	1	112
hsa00460	Cyanoamino acid metabolism	17589	7	8410	3.346978	3	0.736075	1	112
hsa05142	Chagas disease (American trypanosomiasis)	17589	102	8410	48.77025	46	0.741821	1	112
hsa05214	Glioma	17589	63	8410	30.1228	28	0.745744	1	112
hsa00630	Glyoxylate and dicarboxylate metabolism	17589	21	8410	10.04093	9	0.748607	1	112
hsa04010	MAPK signaling pathway	17589	234	8410	111.8847	107	0.760787	1	112
hsa05221	Acute myeloid leukemia	17589	57	8410	27.25397	25	0.767304	1	112

hsa05146	Amoebiasis	17589	103	8410	49.24839	46	0.770544	1	112
hsa04664	Fc epsilon RI signaling pathway	17589	66	8410	31.55722	29	0.774445	1	112
hsa00280	Valine, leucine and isoleucine degradation	17589	44	8410	21.03815	19	0.777986	1	112
hsa04961	Endocrine and other factor-regulated calcium reabsorption	17589	44	8410	21.03815	19	0.777986	1	112
hsa04726	Serotonergic synapse	17589	99	8410	47.33583	44	0.780267	1	112
hsa00100	Steroid biosynthesis	17589	17	8410	8.128376	7	0.784601	1	112
hsa05340	Primary immunodeficiency	17589	33	8410	15.77861	14	0.786119	1	112
hsa00740	Riboflavin metabolism	17589	10	8410	4.781397	4	0.789983	1	112
hsa00920	Sulfur metabolism	17589	10	8410	4.781397	4	0.789983	1	112
hsa05152	Tuberculosis	17589	158	8410	75.54608	71	0.790092	1	112
hsa04916	Melanogenesis	17589	95	8410	45.42328	42	0.79023	1	112
hsa05016	Huntington,s disease	17589	169	8410	80.80562	76	0.794031	1	112
hsa04210	Apoptosis	17589	82	8410	39.20746	36	0.7941	1	112
hsa00010	Glycolysis / Gluconeogenesis	17589	60	8410	28.68838	26	0.795154	1	112
hsa04070	Phosphatidylinositol signaling system	17589	78	8410	37.2949	34	0.8055	1	112
hsa04971	Gastric acid secretion	17589	67	8410	32.03536	29	0.806636	1	112
hsa00565	Ether lipid metabolism	17589	38	8410	18.16931	16	0.806937	1	112
hsa04740	Olfactory transduction	17589	38	8410	18.16931	16	0.806937	1	112
hsa00561	Glycerolipid metabolism	17589	45	8410	21.51629	19	0.816077	1	112
hsa04912	GnRH signaling pathway	17589	85	8410	40.64188	37	0.816223	1	112
hsa04730	Long-term depression	17589	54	8410	25.81955	23	0.817287	1	112
hsa04020	Calcium signaling	17589	166	8410	79.3712	74	0.820255	1	112
hsa05213	Endometrial cancer	17589	52	8410	24.86327	22	0.825001	1	112
hsa04672	Intestinal immune network for IgA production	17589	43	8410	20.56001	18	0.825044	1	112
hsa05031	Amphetamine addiction	17589	61	8410	29.16652	26	0.826659	1	112
hsa04068	FoxO signaling pathway	17589	125	8410	59.76747	55	0.828012	1	112
hsa04146	Peroxisome	17589	77	8410	36.81676	33	0.838153	1	112
hsa04670	Leukocyte transendothelial migration	17589	108	8410	51.63909	47	0.83964	1	112
hsa05014	Amyotrophic lateral sclerosis (ALS)	17589	48	8410	22.95071	20	0.840954	1	112
hsa04060	Cytokine-cytokine receptor interaction	17589	228	8410	109.0159	102	0.842065	1	112
hsa04140	Regulation of autophagy	17589	23	8410	10.99721	9	0.851634	1	112
hsa05012	Parkinson,s disease	17589	124	8410	59.28933	54	0.851908	1	112
hsa00785	Lipoic acid metabolism	17589	3	8410	1.434419	1	0.8579	1	112
hsa05164	Influenza A	17589	151	8410	72.1991	66	0.863557	1	112
hsa04913	Ovarian steroidogenesis	17589	42	8410	20.08187	17	0.866225	1	112
hsa00592	alpha-Linolenic acid metabolism	17589	21	8410	10.04093	8	0.866976	1	112
hsa04340	Hedgehog signaling pathway	17589	49	8410	23.42885	20	0.869958	1	112
hsa05032	Morphine addiction	17589	74	8410	35.38234	31	0.872746	1	112
hsa05320	Autoimmune thyroid disease	17589	33	8410	15.77861	13	0.87394	1	112
hsa00030	Pentose phosphate pathway	17589	26	8410	12.43163	10	0.875716	1	112

hsa04650	Natural killer cell mediated cytotoxicity	17589	110	8410	52.59537	47	0.878581	1	112
hsa04064	NF-kappa B signaling pathway	17589	88	8410	42.0763	37	0.883785	1	112
hsa04724	Glutamatergic synapse	17589	106	8410	50.68281	45	0.886266	1	112
hsa00430	Taurine and hypotaurine metabolism	17589	9	8410	4.303258	3	0.886763	1	112
hsa04622	RIG-I-like receptor signaling pathway	17589	52	8410	24.86327	21	0.887808	1	112
hsa03022	Basal transcription factors	17589	43	8410	20.56001	17	0.893152	1	112
hsa04910	Insulin signaling pathway	17589	131	8410	62.63631	56	0.895086	1	112
hsa04142	Lysosome	17589	120	8410	57.37677	51	0.896584	1	112
hsa05215	Prostate cancer	17589	87	8410	41.59816	36	0.905596	1	112
hsa04014	Ras signaling pathway	17589	203	8410	97.06237	88	0.911922	1	112
hsa04620	Toll-like receptor signaling pathway	17589	90	8410	43.03258	37	0.916914	1	112
hsa00591	Linoleic acid metabolism	17589	25	8410	11.95349	9	0.917828	1	112
hsa04966	Collecting duct acid secretion	17589	25	8410	11.95349	9	0.917828	1	112
hsa00290	Valine, leucine and isoleucine biosynthesis	17589	4	8410	1.912559	1	0.925855	1	112
hsa04720	Long-term potentiation	17589	61	8410	29.16652	24	0.927758	1	112
hsa05330	Allograft rejection	17589	33	8410	15.77861	12	0.933232	1	112
hsa04662	B cell receptor signaling pathway	17589	71	8410	33.94792	28	0.938201	1	112
hsa04962	Vasopressin-regulated water reabsorption	17589	43	8410	20.56001	16	0.939912	1	112
hsa04744	Phototransduction	17589	21	8410	10.04093	7	0.940756	1	112
hsa00980	Metabolism of xenobiotics by cytochrome P450	17589	55	8410	26.29769	21	0.942241	1	112
hsa00500	Starch and sucrose metabolism	17589	36	8410	17.21303	13	0.943321	1	112
hsa04723	Retrograde endocannabinoid	17589	83	8410	39.6856	33	0.943803	1	112
hsa05145	Toxoplasmosis	17589	115	8410	54.98607	47	0.944444	1	112
hsa05160	Hepatitis C	17589	113	8410	54.02979	46	0.946914	1	112
hsa04932	Non-alcoholic fatty liver disease (NAFLD)	17589	138	8410	65.98329	57	0.948034	1	112
hsa04710	Circadian rhythm	17589	29	8410	13.86605	10	0.949167	1	112
hsa04728	Dopaminergic synapse	17589	121	8410	57.85491	49	0.956663	1	112
hsa00052	Galactose metabolism	17589	27	8410	12.90977	9	0.956922	1	112
hsa04915	Estrogen signaling pathway	17589	94	8410	44.94514	37	0.960351	1	112
hsa04370	VEGF signaling pathway	17589	59	8410	28.21025	22	0.960882	1	112
hsa00524	Butirosin and neomycin biosynthesis	17589	5	8410	2.390699	1	0.961315	1	112
hsa04721	Synaptic vesicle cycle	17589	57	8410	27.25397	21	0.964392	1	112
hsa05204	Chemical carcinogenesis	17589	60	8410	28.68838	22	0.969412	1	112
hsa04727	GABAergic synapse	17589	72	8410	34.42606	27	0.9702	1	112
hsa00514	Other types of O-glycan biosynthesis	17589	26	8410	12.43163	8	0.975239	1	112
hsa04722	Neurotrophin signaling pathway	17589	118	8410	56.42049	46	0.978715	1	112
hsa00640	Propanoate metabolism	17589	32	8410	15.30047	10	0.981309	1	112

hsa04660	T cell receptor signaling pathway	17589	101	8410	48.29211	38	0.984917	1	112
hsa05120	Epithelial cell signaling in Helicobacter pylori infection	17589	66	8410	31.55722	23	0.987936	1	112
hsa04742	Taste transduction	17589	31	8410	14.82233	9	0.989678	1	112
hsa00534	Glycosaminoglycan biosynthesis - heparan sulfate / heparin	17589	23	8410	10.99721	6	0.990608	1	112
hsa00190	Oxidative phosphorylation	17589	114	8410	54.50793	42	0.993108	1	112
hsa00531	Glycosaminoglycan degradation	17589	18	8410	8.606515	4	0.993647	1	112
hsa05033	Nicotine addiction	17589	24	8410	11.47535	6	0.993912	1	112
hsa00760	Nicotinate and nicotinamide metabolism	17589	22	8410	10.51907	5	0.996013	1	112
hsa00511	Other glycan degradation	17589	17	8410	8.128376	3	0.997944	1	112
hsa03010	Ribosome	17589	128	8410	61.20189	43	0.999606	1	112
hsa00300	Lysine biosynthesis	17589	2	8410	0.956279	0	1	1	112
hsa00471	D-Glutamine and D-glutamate metabolism	17589	4	8410	1.912559	0	1	1	112
