# Supplementary material for: A network-based pathway-extending approach using DNA methylation and gene expression data to identify altered pathways
Source: Sci Rep. 2019 Aug 14;9:11853. doi: 10.1038/s41598-019-48372-1 (PMC6694157; doi:10.1038/s41598-019-48372-1)
Supplement: Supplementary file 4 — Supplementary Table S4 [file 41598_2019_48372_MOESM4_ESM.pdf]

A network-based pathway-extending approach using DNA methylation and gene expression data to identify altered pathways

Jie Li[1], Qiaosheng Zhang[1,2,*], Zhuo Chen[1], Dechen Xu[1], and Yadong Wang[1]

[1]Harbin Institute of Technology, School of Computer Science and Technology, Harbin, 150001, P.R. China

[2]Heilongjiang Bayi Agricultural University, College of Science, Daqing, 163319, P.R. China

[*]zqs@hit.edu.cn

All results in LUAD dataset by EP-GSEA

Pathway ID	Pathway Name	SIZE	ES	NES	NOM p-va	FDR q-val	Rank
hsa03430	Mismatch repair	61	-0.59082	-1.76421	0.001898	0.07508	1
hsa03420	Nucleotide excision repair	109	-0.48705	-1.71862	0	0.093178	2
hsa00970	Aminoacyl-tRNA biosynthesis	123	-0.4556	-1.76877	0	0.107051	3
hsa03030	DNA replication	94	-0.61419	-1.80422	0.009766	0.139273	4
hsa00270	Cysteine and methionine metabolism	93	-0.41996	-1.65669	0.003831	0.141785	5
hsa00240	Pyrimidine metabolism	239	-0.41065	-1.60669	0.003861	0.142341	6
hsa03460	Fanconi anemia pathway	103	-0.48726	-1.61242	0.020408	0.153562	7
hsa03320	PPAR signaling pathway	170	0.350489	1.580217	0.003868	0.157032	8
hsa00590	Arachidonic acid metabolism	136	0.39689	1.580744	0.015748	0.162589	9
hsa04742	Taste transduction	61	0.434476	1.507743	0.032193	0.163324	10
hsa04611	Platelet activation	340	0.430566	1.569673	0.022472	0.16435	11
hsa04514	Cell adhesion molecules (CAMs)	310	0.437101	1.509754	0.089524	0.164662	12
hsa04670	Leukocyte transendothelial migration	251	0.407816	1.493387	0.055762	0.16575	13
hsa04614	Renin-angiotensin system	50	0.448429	1.51117	0.049702	0.166555	14
hsa04713	Circadian entrainment	220	0.395384	1.49542	0.025794	0.16677	15
hsa03440	Homologous recombination	62	-0.54522	-1.62006	0.035503	0.167695	16
hsa04744	Phototransduction	48	0.426814	1.581203	0.015873	0.168265	17
hsa04390	Hippo signaling pathway	362	0.36697	1.512573	0.015414	0.168267	18
hsa05414	Dilated cardiomyopathy	197	0.44787	1.56221	0.021236	0.168324	19
hsa04022	cGMP-PKG signaling pathway	389	0.361642	1.500473	0.017274	0.168615	20
hsa04917	Prolactin signaling pathway	189	0.386459	1.497017	0.040153	0.168701	21
hsa05144	Malaria	126	0.526736	1.514929	0.08577	0.169567	22
hsa04530	Tight junction	285	0.378807	1.584519	0.00188	0.170474	23
hsa04066	HIF-1 signaling pathway	288	0.345822	1.436488	0.053131	0.171362	24
hsa04728	Dopaminergic synapse	307	0.339742	1.457851	0.029183	0.17156	25
hsa04921	Oxytocin signaling pathway	357	0.42175	1.637993	0	0.171754	26
hsa05204	Chemical carcinogenesis	118	0.41134	1.534256	0.033932	0.171876	27
hsa05120	Epithelial cell signaling in Helicobacter pylori infection	200	0.338445	1.437763	0.035573	0.172259	28
hsa04722	Neurotrophin signaling pathway	316	0.329783	1.439896	0.035647	0.172326	29
hsa05150	Staphylococcus aureus infection	98	0.594764	1.541775	0.133462	0.172568	30
hsa04144	Endocytosis	494	0.3579	1.515665	0.007449	0.172723	31
hsa04750	Inflammatory mediator regulation of TRP channels	236	0.359164	1.459034	0.028302	0.172795	32
hsa04650	Natural killer cell mediated cytotoxicity	273	0.434679	1.453551	0.130518	0.173049	33
hsa05219	Bladder cancer	115	0.366914	1.441463	0.034816	0.173172	34
hsa05310	Asthma	60	0.575813	1.55352	0.10099	0.173384	35
hsa00601	Glycosphingolipid biosynthesis - lacto and neolacto series	40	-0.45129	-1.53509	0.029228	0.17351	36
hsa04015	Rap1 signaling pathway	487	0.397438	1.548833	0.015009	0.174103	37
hsa04724	Glutamatergic synapse	237	0.39687	1.517553	0.017143	0.17483	38
hsa04910	Insulin signaling pathway	338	0.324142	1.428778	0.020833	0.174869	39
hsa05217	Basal cell carcinoma	119	0.398283	1.442169	0.061538	0.17509	40
hsa00982	Drug metabolism - cytochrome P450	98	0.393182	1.45962	0.062	0.175104	41
hsa05143	African trypanosomiasis	105	0.467037	1.535364	0.068762	0.175303	42
hsa04971	Gastric acid secretion	156	0.451857	1.752293	0	0.175543	43

hsa00512	Mucin type O-Glycan biosynthesis	52	-0.44256	-1.54443	0.035433	0.175858	44
hsa03060	Protein export	64	-0.43442	-1.52135	0.027559	0.176036	45
hsa04972	Pancreatic secretion	198	0.405914	1.613781	0.007767	0.176492	46
hsa05332	Graft-versus-host disease	80	0.543868	1.429192	0.169291	0.17697	47
hsa00500	Starch and sucrose metabolism	89	0.338936	1.41565	0.037736	0.176979	48
hsa05133	Pertussis	202	0.435247	1.460599	0.116505	0.177181	49
hsa03410	Base excision repair	105	-0.41489	-1.55581	0.037328	0.177218	50
hsa00592	alpha-Linolenic acid	58	0.37875	1.42174	0.070881	0.177257	51
hsa04610	Complement and coagulation cascades	147	0.46045	1.542132	0.052427	0.177285	52
hsa04261	Adrenergic signaling in cardiomyocytes	337	0.372036	1.584846	0.003839	0.177307	53
hsa04130	SNARE interactions in vesicular transport	75	0.347527	1.41754	0.042644	0.177553	54
hsa05320	Autoimmune thyroid disease	69	0.562162	1.442373	0.158103	0.17767	55
hsa04640	Hematopoietic cell lineage	187	0.474925	1.412956	0.17433	0.177689	56
hsa04912	GnRH signaling pathway	222	0.344089	1.446719	0.014599	0.177844	57
hsa05146	Amoebiasis	259	0.44163	1.518579	0.032015	0.178115	58
hsa04010	MAPK signaling pathway	575	0.344364	1.478949	0.019011	0.178215	59
hsa04310	Wnt signaling pathway	344	0.321912	1.422564	0.032197	0.178761	60
hsa04540	Gap junction	223	0.362542	1.461754	0.036969	0.178855	61
hsa04962	Vasopressin-regulated water reabsorption	117	0.423307	1.648575	0.001965	0.179134	62
hsa04911	Insulin secretion	180	0.369088	1.464069	0.040936	0.179151	63
hsa04920	Adipocytokine signaling pathway	184	0.346723	1.41783	0.058594	0.179376	64
hsa05211	Renal cell carcinoma	175	0.380869	1.442954	0.05303	0.17972	65
hsa04014	Ras signaling pathway	517	0.363544	1.46587	0.026667	0.180371	66
hsa04727	GABAergic synapse	170	0.415897	1.639514	0.003854	0.180697	67
hsa05213	Endometrial cancer	146	0.388769	1.471012	0.031128	0.180713	68
hsa04080	Neuroactive ligand-receptor interaction	337	0.398522	1.617647	0.001957	0.180803	69
hsa05410	Hypertrophic cardiomyopathy (HCM)	182	0.440601	1.519911	0.02947	0.180988	70
hsa04110	Cell cycle	315	-0.35222	-1.50685	0.048117	0.181819	71
hsa04062	Chemokine signaling pathway	441	0.381304	1.466824	0.083333	0.182405	72
hsa04810	Regulation of actin cytoskeleton	503	0.338544	1.398927	0.058824	0.182447	73
hsa04726	Serotonergic synapse	241	0.396801	1.58677	0.003906	0.182508	74
hsa04961	Endocrine and other factor-regulated calcium reabsorption	111	0.431881	1.623268	0.003817	0.182757	75
hsa00980	Metabolism of xenobiotics by cytochrome P450	100	0.402686	1.40281	0.102941	0.183097	76
hsa05205	Proteoglycans in cancer	521	0.360468	1.471506	0.035055	0.18372	77
hsa04725	Cholinergic synapse	249	0.34573	1.399808	0.059048	0.18387	78
hsa04666	Fc gamma R-mediated phagocytosis	240	0.384591	1.52165	0.039848	0.183959	79
hsa04916	Melanogenesis	236	0.385852	1.590706	0.001949	0.184665	80
hsa04664	Fc epsilon RI signaling pathway	183	0.374792	1.403318	0.087891	0.185034	81
hsa04960	Aldosterone-regulated sodium reabsorption	101	0.487447	1.593894	0.02729	0.188416	82
hsa04730	Long-term depression	144	0.448742	1.651127	0.007491	0.188491	83
hsa00591	Linoleic acid metabolism	58	0.445991	1.59931	0.022	0.190591	84
hsa04068	FoxO signaling pathway	366	0.328108	1.388318	0.039623	0.191512	85

hsa03020	RNA polymerase	71	-0.45527	-1.5587	0.017717	0.192834	86
hsa04723	Retrograde endocannabinoid signaling	187	0.420128	1.655015	0.001931	0.197807	87
hsa04913	Ovarian steroidogenesis	115	0.432852	1.761572	0	0.199472	88
hsa04020	Calcium signaling pathway	373	0.417125	1.658599	0.001957	0.208282	89
hsa00830	Retinol metabolism	85	0.496546	1.665224	0.014085	0.214895	90
hsa04720	Long-term potentiation	159	0.444183	1.713951	0	0.215601	91
hsa04918	Thyroid hormone synthesis	158	0.340314	1.359237	0.063492	0.224519	92
hsa05142	Chagas disease (American trypanosomiasis)	280	0.365623	1.342308	0.145315	0.225688	93
hsa05200	Pathways in cancer	843	0.303222	1.338969	0.079336	0.227086	94
hsa04380	Osteoclast differentiation	326	0.393138	1.342749	0.199627	0.227444	95
hsa04973	Carbohydrate digestion and absorption	91	0.386962	1.344774	0.145349	0.227479	96
hsa04915	Estrogen signaling pathway	243	0.318044	1.346404	0.067416	0.22796	97
hsa04710	Circadian rhythm	75	0.347659	1.33641	0.106343	0.228149	98
hsa02010	ABC transporters	101	0.304093	1.3482	0.0501	0.228233	99
hsa04930	Type II diabetes mellitus	131	0.360654	1.353918	0.084479	0.228833	100
hsa04510	Focal adhesion	492	0.365307	1.348635	0.156425	0.230177	101
hsa04145	Phagosome	344	0.354375	1.349963	0.174081	0.230882	102
hsa05032	Morphine addiction	168	0.455708	1.667481	0.003883	0.234493	103
hsa04740	Olfactory transduction	51	0.473506	1.691456	0.001972	0.239355	104
hsa05223	Non-small cell lung cancer	167	0.318241	1.318591	0.071984	0.243278	105
hsa00140	Steroid hormone biosynthesis	71	0.330204	1.322238	0.086694	0.24337	106
hsa05140	Leishmaniasis	169	0.424099	1.319984	0.250478	0.243885	107
hsa04976	Bile secretion	136	0.422946	1.674525	0.002024	0.246765	108
hsa05330	Allograft rejection	76	0.49477	1.312783	0.232422	0.247862	109
hsa04120	Ubiquitin mediated proteolysis	351	-0.30126	-1.43849	0.012048	0.252599	110
hsa05020	Prion diseases	93	0.504925	1.764517	0	0.255157	111
hsa03013	RNA transport	367	-0.34329	-1.44708	0.020619	0.255377	112
hsa05031	Amphetamine addiction	164	0.307205	1.304683	0.106299	0.25603	113
hsa04012	ErbB signaling pathway	236	0.308333	1.300043	0.133581	0.257254	114
hsa04630	Jak-STAT signaling pathway	254	0.368785	1.293739	0.200385	0.257935	115
hsa04977	Vitamin digestion and absorption	44	0.351336	1.296934	0.137097	0.258666	116
hsa05221	Acute myeloid leukemia	169	0.339667	1.30092	0.139048	0.258764	117
hsa05322	Systemic lupus erythematosus	182	0.339136	1.290991	0.196819	0.25898	118
hsa04360	Axon guidance	299	0.296381	1.294284	0.111732	0.259822	119
hsa04320	Dorso-ventral axis formation	67	0.355992	1.288416	0.136095	0.260143	120
hsa04370	VEGF signaling pathway	179	0.309464	1.282288	0.121857	0.263248	121
hsa04350	TGF-beta signaling pathway	200	0.294551	1.279428	0.113806	0.264534	122
hsa04151	PI3K-Akt signaling pathway	755	0.307539	1.282861	0.147114	0.264921	123
hsa00790	Folate biosynthesis	34	0.362365	1.274658	0.168959	0.266222	124
hsa04070	Phosphatidylinositol signaling system	219	0.312324	1.276137	0.139216	0.266388	125
hsa00670	One carbon pool by folate	57	-0.38659	-1.41522	0.063136	0.274103	126
hsa04919	Thyroid hormone signaling pathway	314	0.290103	1.266183	0.108738	0.274659	127
hsa00510	N-Glycan biosynthesis	120	-0.3692	-1.39546	0.078799	0.276536	128
hsa03008	Ribosome biogenesis in eukaryotes	180	-0.36686	-1.40432	0.07085	0.276712	129
hsa00430	Taurine and hypotaurine metabolism	21	0.378491	1.262114	0.157143	0.277539	130

hsa04974	Protein digestion and absorption	147	0.338365	1.260096	0.206049	0.277679	131
hsa05132	Salmonella infection	226	0.297036	1.255921	0.164093	0.280612	132
hsa05412	Arrhythmogenic right ventricular cardiomyopathy	177	0.335012	1.236946	0.213052	0.282514	133
hsa00564	Glycerophospholipid metabolism	193	0.277161	1.246323	0.106931	0.283605	134
hsa05131	Shigellosis	177	0.290651	1.234273	0.161943	0.283895	135
hsa04060	Cytokine-cytokine receptor interaction	463	0.339705	1.232382	0.260536	0.284328	136
hsa00350	Tyrosine metabolism	82	0.342693	1.237029	0.195789	0.284727	137
hsa05321	Inflammatory bowel disease (IBD)	140	0.417043	1.246652	0.270115	0.285456	138
hsa04978	Mineral absorption	113	0.310732	1.239339	0.173387	0.286422	139
hsa00565	Ether lipid metabolism	95	0.316714	1.240784	0.1611	0.286859	140
hsa05416	Viral myocarditis	129	0.368864	1.237132	0.27704	0.286905	141
hsa04150	mTOR signaling pathway	153	0.285162	1.242258	0.144814	0.287081	142
hsa05202	Transcriptional misregulation in cancer	419	0.299899	1.247338	0.185321	0.28711	143
hsa05210	Colorectal cancer	176	0.283318	1.226451	0.158582	0.287654	144
hsa04660	T cell receptor signaling pathway	269	0.349998	1.248619	0.237192	0.287917	145
hsa04330	Notch signaling pathway	122	0.293464	1.22736	0.154851	0.28869	146
hsa04520	Adherens junction	211	0.293793	1.222894	0.171698	0.290278	147
hsa05323	Rheumatoid arthritis	200	0.374147	1.22001	0.279297	0.292104	148
hsa04612	Antigen processing and presentation	154	0.342055	1.216004	0.254438	0.295304	149
hsa04340	Hedgehog signaling pathway	113	0.306834	1.206925	0.193548	0.304889	150
hsa05206	MicroRNAs in cancer	435	0.27663	1.20415	0.194444	0.306306	151
hsa00230	Purine metabolism	381	-0.28214	-1.36744	0.024242	0.309356	152
hsa04152	AMPK signaling pathway	304	0.246135	1.19752	0.126679	0.31344	153
hsa05164	Influenza A	404	0.288485	1.194809	0.236893	0.314776	154
hsa04672	Intestinal immune network for IgA production	92	0.413634	1.185308	0.339695	0.324773	155
hsa00562	Inositol phosphate metabolism	173	0.280737	1.18011	0.207294	0.329658	156
hsa05030	Cocaine addiction	126	0.29662	1.176179	0.232283	0.330516	157
hsa05100	Bacterial invasion of epithelial cells	201	0.290154	1.177419	0.25046	0.330938	158
hsa00250	Alanine, aspartate and glutamate metabolism	84	-0.33485	-1.34105	0.09919	0.339563	159
hsa05152	Tuberculosis	404	0.295052	1.157017	0.293666	0.352854	160
hsa04975	Fat digestion and absorption	64	0.323529	1.157032	0.277132	0.355326	161
hsa01040	Biosynthesis of unsaturated fatty acids	46	0.33885	1.14787	0.276768	0.363411	162
hsa04260	Cardiac muscle contraction	144	0.281536	1.145243	0.287234	0.364574	163
hsa00380	Tryptophan metabolism	97	0.272269	1.136415	0.265625	0.374583	164
hsa00524	Butirosin and neomycin biosynthesis	15	0.388679	1.130402	0.303263	0.375706	165
hsa05166	HTLV-I infection	655	0.252656	1.131769	0.26834	0.37613	166
hsa00040	Pentose and glucuronate interconversions	44	0.325137	1.12829	0.291405	0.376328	167
hsa05034	Alcoholism	300	0.236968	1.13332	0.234405	0.376464	168
hsa04141	Protein processing in endoplasmic reticulum	417	-0.28647	-1.29602	0.100604	0.377601	169

hsa03040	Spliceosome	288	-0.33346	-1.31232	0.153398	0.37831	170
hsa00400	Phenylalanine, tyrosine and tryptophan biosynthesis	7	-0.62118	-1.287	0.190476	0.37956	171
hsa04270	Vascular smooth muscle contraction	284	0.475727	1.764787	0	0.382735	172
hsa05145	Toxoplasmosis	297	0.304274	1.120475	0.330739	0.384959	173
hsa00770	Pantothenate and CoA biosynthesis	47	-0.36223	-1.27499	0.162698	0.387385	174
hsa05168	Herpes simplex infection	410	0.263777	1.114722	0.297456	0.391549	175
hsa05218	Melanoma	149	0.277799	1.112639	0.306839	0.392332	176
hsa04115	p53 signaling pathway	190	-0.27126	-1.29685	0.07362	0.392454	177
hsa04668	TNF signaling pathway	304	0.280606	1.109054	0.322266	0.394748	178
hsa00290	Valine, leucine and isoleucine biosynthesis	9	-0.57674	-1.26255	0.217308	0.396614	179
hsa00120	Primary bile acid biosynthesis	37	0.34797	1.098172	0.334025	0.408006	180
hsa05134	Legionellosis	166	0.296282	1.095194	0.358416	0.409759	181
hsa05340	Primary immunodeficiency	90	0.34921	1.088966	0.374257	0.413766	182
hsa05161	Hepatitis B	386	0.245428	1.089566	0.307836	0.415541	183
hsa05220	Chronic myeloid leukemia	207	0.265891	1.084797	0.328923	0.41746	184
hsa00533	Glycosaminoglycan biosynthesis - keratan sulfate	28	-0.36521	-1.23279	0.176829	0.422796	185
hsa00360	Phenylalanine metabolism	53	0.276049	1.0729	0.339251	0.427205	186
hsa05214	Glioma	178	0.252363	1.073775	0.322097	0.428576	187
hsa00051	Fructose and mannose metabolism	88	-0.28744	-1.23664	0.144898	0.430706	188
hsa05215	Prostate cancer	247	0.244261	1.073814	0.36	0.431205	189
hsa05014	Amyotrophic lateral sclerosis (ALS)	134	0.252391	1.060856	0.363636	0.442561	190
hsa05160	Hepatitis C	302	0.241624	1.05595	0.343811	0.447384	191
hsa05130	Pathogenic Escherichia coli infection	147	0.246256	1.034572	0.392857	0.477191	192
hsa00620	Pyruvate metabolism	115	-0.28775	-1.17268	0.227444	0.483108	193
hsa04950	Maturity onset diabetes of the young	40	-0.32025	-1.14369	0.271154	0.485212	194
hsa03022	Basal transcription factors	108	-0.27705	-1.16361	0.23002	0.487181	195
hsa03015	mRNA surveillance pathway	210	-0.27129	-1.17589	0.215842	0.491482	196
hsa03018	RNA degradation	163	-0.26157	-1.18238	0.161654	0.49414	197
hsa04142	Lysosome	309	0.241802	1.020476	0.39802	0.494778	198
hsa05216	Thyroid cancer	87	0.236076	1.021731	0.42155	0.495613	199
hsa00450	Selenocompound metabolism	41	-0.30618	-1.14515	0.253876	0.495744	200
hsa04122	Sulfur relay system	26	-0.37786	-1.14871	0.294466	0.50293	201
hsa00520	Amino sugar and nucleotide sugar metabolism	130	-0.28857	-1.18601	0.210317	0.503098	202
hsa00534	Glycosaminoglycan biosynthesis - heparan sulfate / heparin	38	-0.30716	-1.1186	0.284519	0.507562	203
hsa00603	Glycosphingolipid biosynthesis - globo series	27	0.327916	1.008972	0.439759	0.507786	204
hsa04940	Type I diabetes mellitus	88	0.333496	1.010542	0.453307	0.508372	205
hsa03450	Non-homologous end-joining	40	-0.30335	-1.10335	0.316306	0.511195	206
hsa00983	Drug metabolism - other enzymes	73	-0.26853	-1.10619	0.300403	0.518387	207
hsa04114	Oocyte meiosis	281	-0.2346	-1.1191	0.285141	0.519999	208
hsa04662	B cell receptor signaling pathway	195	0.263398	0.999631	0.435606	0.520596	209

hsa04512	ECM-receptor interaction	189	0.293413	0.994852	0.477099	0.525307	210
hsa04620	Toll-like receptor signaling pathway	225	0.258482	0.985544	0.448473	0.534886	211
hsa00760	Nicotinate and nicotinamide metabolism	58	0.239359	0.985674	0.492032	0.537843	212
hsa04964	Proximal tubule bicarbonate reclamation	53	0.234258	0.98078	0.520325	0.539853	213
hsa05162	Measles	326	0.243805	0.975107	0.457627	0.539995	214
hsa05033	Nicotine addiction	50	0.275433	0.976839	0.508097	0.540151	215
hsa05110	Vibrio cholerae infection	133	0.236292	0.977008	0.509054	0.542943	216
hsa00600	Sphingolipid metabolism	89	0.235052	0.963967	0.492337	0.555943	217
hsa04210	Apoptosis	208	0.233105	0.953353	0.508704	0.570806	218
hsa00071	Fatty acid degradation	102	0.244378	0.950157	0.511156	0.573217	219
hsa00260	Glycine, serine and threonine metabolism	111	0.206168	0.941446	0.566731	0.578869	220
hsa05222	Small cell lung cancer	237	0.221934	0.941914	0.52907	0.581246	221
hsa00020	Citrate cycle (TCA cycle)	89	0.262565	0.94305	0.503198	0.582467	222
hsa04064	NF-kappa B signaling pathway	225	0.262115	0.931074	0.522472	0.589946	223
hsa00780	Biotin metabolism	7	0.381124	0.93168	0.56067	0.591998	224
hsa00514	Other types of O-glycan biosynthesis	62	-0.25776	-1.05041	0.353659	0.603216	225
hsa04140	Regulation of autophagy	84	0.21079	0.916993	0.625767	0.604736	226
hsa00604	Glycosphingolipid biosynthesis - ganglio series	37	0.248224	0.919459	0.56746	0.607197	227
hsa00531	Glycosaminoglycan degradation	41	0.274836	0.917219	0.545825	0.607526	228
hsa04621	NOD-like receptor signaling pathway	148	0.2252	0.910445	0.549793	0.613128	229
hsa00920	Sulfur metabolism	25	-0.32296	-1.03658	0.410058	0.617081	230
hsa03050	Proteasome	106	-0.30258	-1.01872	0.452427	0.62593	231
hsa04970	Salivary secretion	180	0.504357	1.784807	0	0.627461	232
hsa04914	Progesterone-mediated oocyte maturation	236	-0.21644	-1.01124	0.427435	0.627921	233
hsa00100	Steroid biosynthesis	42	-0.28739	-1.0239	0.415686	0.629141	234
hsa04721	Synaptic vesicle cycle	132	0.207925	0.897573	0.641237	0.632429	235
hsa00010	Glycolysis / Gluconeogenesis	167	0.186633	0.86624	0.758333	0.683693	236
hsa05203	Viral carcinogenesis	526	0.185256	0.860616	0.6826	0.690259	237
hsa00340	Histidine metabolism	53	0.220287	0.852046	0.686117	0.701365	238
hsa00310	Lysine degradation	116	-0.21391	-0.92458	0.569767	0.709862	239
hsa00650	Butanoate metabolism	63	-0.24406	-0.9356	0.544379	0.714215	240
hsa05016	Huntington,s disease	442	-0.21022	-0.93799	0.53876	0.722862	241
hsa03010	Ribosome	321	-0.28432	-0.92481	0.54224	0.723072	242
hsa00053	Ascorbate and aldarate metabolism	32	-0.26952	-0.95129	0.519337	0.723998	243
hsa00480	Glutathione metabolism	115	-0.23098	-0.90976	0.567568	0.727335	244
hsa00061	Fatty acid biosynthesis	19	-0.30776	-0.95547	0.520154	0.73077	245
hsa00860	Porphyrin and chlorophyll metabolism	65	-0.25304	-0.9409	0.535985	0.731586	246
hsa00532	Glycosaminoglycan biosynthesis - chondroitin sulfate / dermatan sulfate	39	0.249328	0.824792	0.696565	0.742111	247
hsa04622	RIG-I-like receptor signaling pathway	148	0.196308	0.825799	0.717308	0.744148	248
hsa00410	beta-Alanine metabolism	79	-0.2251	-0.89406	0.621928	0.746196	249
hsa00030	Pentose phosphate pathway	79	-0.23119	-0.87187	0.636719	0.750535	250

hsa00330	Arginine and proline	141	-0.20788	-0.86385	0.674651	0.753633	251
hsa00730	Thiamine metabolism	7	-0.36009	-0.87589	0.618	0.756375	252
hsa00640	Propanoate metabolism	95	0.194097	0.805706	0.785567	0.759628	253
hsa00910	Nitrogen metabolism	32	0.218759	0.80797	0.806584	0.759751	254
hsa00072	Synthesis and degradation of ketone bodies	26	0.248911	0.811569	0.722222	0.761338	255
hsa00740	Riboflavin metabolism	31	0.253668	0.808879	0.718884	0.761948	256
hsa05010	Alzheimer,s disease	403	0.178488	0.80041	0.766807	0.764323	257
hsa00471	D-Glutamine and D-glutamate metabolism	12	-0.31731	-0.87865	0.595041	0.764456	258
hsa05212	Pancreatic cancer	201	0.178517	0.79189	0.85098	0.774886	259
hsa00232	Caffeine metabolism	13	0.286071	0.785046	0.772467	0.778418	260
hsa00561	Glycerolipid metabolism	109	0.173038	0.786773	0.895299	0.779471	261
hsa05169	Epstein-Barr virus infection	537	-0.18151	-0.84315	0.735294	0.781213	262
hsa04146	Peroxisome	184	0.182106	0.772592	0.81761	0.794957	263
hsa00280	Valine, leucine and isoleucine degradation	121	0.196097	0.757375	0.768612	0.814694	264
hsa00750	Vitamin B6 metabolism	17	-0.28694	-0.80196	0.711656	0.831122	265
hsa04932	Non-alcoholic fatty liver disease (NAFLD)	366	-0.17301	-0.78417	0.787476	0.83718	266
hsa00130	Ubiquinone and other terpenoid-quinone biosynthesis	28	-0.25475	-0.80472	0.710425	0.839578	267
hsa00062	Fatty acid elongation	53	-0.2081	-0.78921	0.794118	0.841041	268
hsa04966	Collecting duct acid secretion	61	0.199673	0.729291	0.84898	0.852307	269
hsa00900	Terpenoid backbone biosynthesis	60	-0.19906	-0.75633	0.849903	0.873187	270
hsa04623	Cytosolic DNA-sensing pathway	115	0.168484	0.701495	0.918812	0.884676	271
hsa00785	Lipoic acid metabolism	12	-0.25375	-0.73521	0.834016	0.892975	272
hsa00563	Glycosylphosphatidylinositol(GPI)-anchor biosynthesis	50	-0.19328	-0.71851	0.88189	0.903588	273
hsa00190	Oxidative phosphorylation	264	-0.19894	-0.67261	0.790927	0.947248	274
hsa00460	Cyanoamino acid metabolism	17	0.212622	0.618236	0.944231	0.949466	275
hsa00052	Galactose metabolism	80	0.165008	0.628486	0.97446	0.952019	276
hsa00630	Glyoxylate and dicarboxylate metabolism	71	0.170604	0.619601	0.960417	0.95311	277
hsa05012	Parkinson,s disease	306	-0.15876	-0.62393	0.920543	0.961359	278
hsa00511	Other glycan degradation	49	-0.16768	-0.62929	0.972	0.9711	279
hsa00300	Lysine biosynthesis	4	-0.31246	-0.56817	0.954357	0.975665	280

All results in LUAD dataset by GSEA

Pathway ID	Pathway Name	SIZE	ES	NES	NOM p-va	FDR q-val	Rank
hsa00970	Aminoacyl-tRNA biosynthesis	43	-0.71072	-1.85335	0	0.098314	1
hsa04110	Cell cycle	118	-0.57211	-1.81421	0.017647	0.105239	2
hsa00670	One carbon pool by folate	19	-0.66749	-1.88176	0.001996	0.123157	3
hsa03430	Mismatch repair	23	-0.68871	-1.64053	0.023669	0.159836	4
hsa00601	Glycosphingolipid biosynthesis - lacto and neolacto series	25	-0.54412	-1.74652	0.010352	0.163422	5
hsa00250	Alanine, aspartate and glutamate metabolism	33	-0.52107	-1.72128	0.008032	0.169854	6
hsa03013	RNA transport	148	-0.52725	-1.62121	0.028513	0.170806	7
hsa00240	Pyrimidine metabolism	99	-0.50202	-1.64249	0.018908	0.172485	8
hsa03008	Ribosome biogenesis in eukaryotes	70	-0.60886	-1.69974	0.020367	0.17346	9
hsa03022	Basal transcription factors	43	-0.52546	-1.57985	0.036511	0.174794	10

hsa03460	Fanconi anemia pathway	46	-0.59518	-1.58828	0.068966	0.175029	11
hsa03030	DNA replication	36	-0.72496	-1.67979	0.021654	0.178407	12
hsa03040	Spliceosome	111	-0.54835	-1.56741	0.063265	0.17966	13
hsa00270	Cysteine and methionine metabolism	37	-0.486	-1.55581	0.025052	0.184025	14
hsa03420	Nucleotide excision repair	45	-0.5371	-1.58848	0.044625	0.187404	15
hsa03060	Protein export	23	-0.61391	-1.54321	0.064646	0.189179	16
hsa03410	Base excision repair	33	-0.61934	-1.64388	0.012448	0.189273	17
hsa03440	Homologous recombination	26	-0.665	-1.59506	0.032258	0.192206	18
hsa05410	Hypertrophic cardiomyopathy (HCM)	74	0.512193	1.641103	0.024948	0.200272	19
hsa04080	Neuroactive ligand-receptor interaction	198	0.441597	1.634797	0.010917	0.200944	20
hsa03450	Non-homologous end-joining	12	-0.72335	-1.65073	0.012121	0.201445	21
hsa04912	GnRH signaling pathway	85	0.450802	1.649525	0.010373	0.206111	22
hsa04713	Circadian entrainment	87	0.471823	1.643358	0.010941	0.206596	23
hsa04916	Melanogenesis	95	0.41356	1.614943	0.006122	0.207834	24
hsa05032	Morphine addiction	74	0.481068	1.615289	0.017241	0.216142	25
hsa04970	Salivary secretion	76	0.500881	1.649865	0.015217	0.217527	26
hsa04972	Pancreatic secretion	76	0.495627	1.65618	0.013015	0.2178	27
hsa05414	Dilated cardiomyopathy	81	0.544737	1.661284	0.015086	0.220232	28
hsa04976	Bile secretion	61	0.488098	1.618112	0.022869	0.220433	29
hsa04022	cGMP-PKG signaling pathway	155	0.46005	1.685044	0.008811	0.221972	30
hsa04270	Vascular smooth muscle contraction	111	0.568496	1.85266	0	0.225689	31
hsa04144	Endocytosis	197	0.422179	1.673521	0.004132	0.228786	32
hsa04730	Long-term depression	54	0.491746	1.690084	0.010616	0.229328	33
hsa04723	Retrograde endocannabinoid signaling	83	0.458689	1.664103	0.008639	0.230505	34
hsa03018	RNA degradation	71	-0.45205	-1.49001	0.068041	0.23127	35
hsa04120	Ubiquitin mediated proteolysis	134	-0.38146	-1.49708	0.039683	0.231511	36
hsa03020	RNA polymerase	31	-0.54569	-1.50364	0.093361	0.232767	37
hsa04750	Inflammatory mediator regulation of TRP channels	89	0.422017	1.586947	0.029046	0.236101	38
hsa04020	Calcium signaling pathway	166	0.450372	1.697063	0.002101	0.236284	39
hsa04724	Glutamatergic synapse	106	0.43026	1.590928	0.014862	0.237564	40
hsa04913	Ovarian steroidogenesis	42	0.536593	1.746462	0.006383	0.243184	41
hsa04720	Long-term potentiation	61	0.445093	1.702751	0	0.250371	42
hsa04740	Olfactory transduction	38	0.509183	1.726806	0.002212	0.255129	43
hsa04115	p53 signaling pathway	66	-0.41007	-1.46341	0.060729	0.261403	44
hsa04921	Oxytocin signaling pathway	145	0.459188	1.708981	0.002141	0.263684	45
hsa04530	Tight junction	120	0.436919	1.753557	0.002188	0.269748	46
hsa04261	Adrenergic signaling in cardiomyocytes	136	0.473736	1.794877	0	0.272959	47
hsa00591	Linoleic acid metabolism	25	0.54652	1.550259	0.028807	0.275259	48
hsa04725	Cholinergic synapse	106	0.42026	1.554615	0.025532	0.277434	49
hsa04015	Rap1 signaling pathway	197	0.428706	1.556657	0.028866	0.283086	50
hsa04010	MAPK signaling pathway	234	0.401829	1.526935	0.028986	0.28758	51
hsa04670	Leukocyte transendothelial migration	108	0.468027	1.530328	0.043659	0.289236	52
hsa04977	Vitamin digestion and absorption	20	0.512589	1.532883	0.062112	0.293619	53
hsa04141	Protein processing in endoplasmic reticulum	159	-0.39426	-1.39924	0.123173	0.295904	54

hsa04971	Gastric acid secretion	67	0.497751	1.765646	0	0.296846	55
hsa04610	Complement and coagulation cascades	61	0.532782	1.534524	0.087129	0.299795	56
hsa00400	Phenylalanine, tyrosine and tryptophan biosynthesis	5	-0.75421	-1.40337	0.07551	0.299874	57
hsa00230	Purine metabolism	155	-0.33768	-1.41654	0.044355	0.301079	58
hsa00510	N-Glycan biosynthesis	49	-0.44938	-1.42343	0.097046	0.301675	59
hsa00512	Mucin type O-Glycan biosynthesis	26	-0.46733	-1.40877	0.116832	0.302449	60
hsa03015	mRNA surveillance pathway	83	-0.41954	-1.38736	0.139535	0.303955	61
hsa00020	Citrate cycle (TCA cycle)	29	-0.52606	-1.38068	0.147541	0.304092	62
hsa04726	Serotonergic synapse	99	0.406856	1.513763	0.028139	0.304578	63
hsa00830	Retinol metabolism	46	0.508946	1.508064	0.093686	0.307178	64
hsa03050	Proteasome	43	-0.61914	-1.42443	0.153684	0.312718	65
hsa00982	Drug metabolism - cytochrome P450	51	0.503464	1.448969	0.105051	0.313417	66
hsa04540	Gap junction	83	0.375922	1.41829	0.079332	0.315072	67
hsa05144	Malaria	44	0.5754	1.424106	0.158635	0.315818	68
hsa04728	Dopaminergic synapse	121	0.339377	1.454511	0.025316	0.316754	69
hsa00071	Fatty acid degradation	41	0.486675	1.419183	0.111336	0.318827	70
hsa04930	Type II diabetes mellitus	42	0.448443	1.425428	0.089936	0.319269	71
hsa05150	Staphylococcus aureus infection	49	0.657787	1.449089	0.162835	0.319712	72
hsa04722	Neurotrophin signaling pathway	118	0.387765	1.456807	0.047414	0.319771	73
hsa05203	Viral carcinogenesis	186	-0.33584	-1.36131	0.073171	0.323169	74
hsa04727	GABAergic synapse	72	0.386741	1.405679	0.067941	0.324582	75
hsa04960	Aldosterone-regulated sodium reabsorption	37	0.475358	1.43557	0.109914	0.324684	76
hsa05412	Arrhythmogenic right ventricular cardiomyopathy	69	0.428358	1.425517	0.116564	0.324992	77
hsa05204	Chemical carcinogenesis	60	0.46583	1.38958	0.149284	0.325192	78
hsa04920	Adipocytokine signaling pathway	65	0.39492	1.456905	0.053719	0.326657	79
hsa04014	Ras signaling pathway	203	0.386387	1.430833	0.050526	0.327	80
hsa04514	Cell adhesion molecules (CAMs)	135	0.468905	1.438105	0.132673	0.327053	81
hsa05332	Graft-versus-host disease	37	0.661725	1.391456	0.161224	0.327093	82
hsa04614	Renin-angiotensin system	16	0.551528	1.460152	0.100418	0.32765	83
hsa04744	Phototransduction	21	0.472513	1.426889	0.090336	0.328266	84
hsa04961	Endocrine and other factor-regulated calcium reabsorption	44	0.419627	1.463919	0.054737	0.328602	85
hsa05132	Salmonella infection	82	0.436016	1.406272	0.117043	0.329237	86
hsa05020	Prion diseases	34	0.501666	1.488072	0.070393	0.329393	87
hsa04380	Osteoclast differentiation	128	0.474993	1.392691	0.189555	0.330176	88
hsa05143	African trypanosomiasis	34	0.525866	1.467171	0.103792	0.330422	89
hsa00140	Steroid hormone biosynthesis	36	0.477493	1.469814	0.089027	0.333208	90
hsa05205	Proteoglycans in cancer	208	0.356088	1.393038	0.083333	0.334867	91
hsa05133	Pertussis	71	0.502	1.489635	0.096838	0.335213	92
hsa00590	Arachidonic acid metabolism	55	0.452633	1.472972	0.059406	0.335238	93
hsa03320	PPAR signaling pathway	62	0.569572	1.877196	0	0.335938	94
hsa04975	Fat digestion and absorption	27	0.437082	1.395626	0.072835	0.336176	95
hsa00051	Fructose and mannose metabolism	31	-0.41816	-1.34341	0.154989	0.341085	96
hsa04611	Platelet activation	127	0.443458	1.474254	0.092672	0.341411	97
hsa00380	Tryptophan metabolism	38	0.412648	1.37357	0.13125	0.345746	98
hsa00592	alpha-Linolenic acid	21	0.539982	1.47562	0.065511	0.347734	99

hsa05416	Viral myocarditis	54	0.502008	1.360068	0.211321	0.348185	100
hsa04390	Hippo signaling pathway	147	0.3325	1.353662	0.083857	0.348485	101
hsa04310	Wnt signaling pathway	133	0.319827	1.356021	0.066381	0.349692	102
hsa05310	Asthma	24	0.67081	1.361685	0.177778	0.350535	103
hsa05140	Leishmaniasis	69	0.513407	1.345992	0.228628	0.352051	104
hsa04114	Oocyte meiosis	102	-0.33458	-1.32917	0.138462	0.353423	105
hsa04911	Insulin secretion	75	0.372856	1.363052	0.121277	0.353487	106
hsa04664	Fc epsilon RI signaling pathway	66	0.420191	1.346169	0.149378	0.356565	107
hsa04350	TGF-beta signaling pathway	80	0.364606	1.36313	0.092593	0.358638	108
hsa04640	Hematopoietic cell lineage	80	0.512384	1.323496	0.205231	0.379996	109
hsa00910	Nitrogen metabolism	15	0.502736	1.324229	0.161157	0.383758	110
hsa00533	Glycosaminoglycan biosynthesis - keratan sulfate	14	-0.46036	-1.29722	0.175676	0.394362	111
hsa04340	Hedgehog signaling pathway	49	0.388772	1.301871	0.173077	0.402059	112
hsa05142	Chagas disease (American trypanosomiasis)	102	0.397183	1.306913	0.186	0.403416	113
hsa04260	Cardiac muscle contraction	62	0.385863	1.302355	0.198428	0.406334	114
hsa04152	AMPK signaling pathway	118	0.302474	1.286856	0.09636	0.407473	115
hsa04810	Regulation of actin cytoskeleton	194	0.346823	1.282979	0.182903	0.408837	116
hsa04062	Chemokine signaling pathway	182	0.393137	1.288399	0.202505	0.410016	117
hsa04360	Axon guidance	123	0.328989	1.225609	0.214286	0.410151	118
hsa04621	NOD-like receptor signaling pathway	54	0.395086	1.227135	0.243243	0.411574	119
hsa00790	Folate biosynthesis	13	0.477131	1.289064	0.161905	0.413917	120
hsa02010	ABC transporters	43	0.371975	1.227982	0.232877	0.413994	121
hsa04650	Natural killer cell mediated cytotoxicity	110	0.412043	1.241043	0.285149	0.414543	122
hsa04520	Adherens junction	73	0.345242	1.214118	0.241667	0.415514	123
hsa04630	Jak-STAT signaling pathway	123	0.379338	1.219267	0.263692	0.415517	124
hsa04917	Prolactin signaling pathway	67	0.365114	1.29096	0.17759	0.41566	125
hsa04666	Fc gamma R-mediated phagocytosis	90	0.363537	1.229352	0.218814	0.415931	126
hsa00531	Glycosaminoglycan degradation	18	0.520297	1.275922	0.230315	0.416417	127
hsa05330	Allograft rejection	33	0.598859	1.250268	0.315369	0.417062	128
hsa05152	Tuberculosis	158	0.380182	1.244524	0.270115	0.417363	129
hsa00330	Arginine and proline	55	-0.3586	-1.27028	0.164583	0.41763	130
hsa05320	Autoimmune thyroid disease	33	0.567054	1.214859	0.344423	0.418355	131
hsa04145	Phagosome	143	0.392649	1.241098	0.276718	0.418713	132
hsa05321	Inflammatory bowel disease (IBD)	57	0.496843	1.251851	0.272189	0.419061	133
hsa04070	Phosphatidylinositol signaling system	78	0.351985	1.229971	0.217204	0.419285	134
hsa00564	Glycerophospholipid metabolism	83	0.316462	1.246075	0.162055	0.419524	135
hsa04742	Taste transduction	31	0.383331	1.235051	0.198745	0.419532	136
hsa04962	Vasopressin-regulated water reabsorption	43	0.353755	1.252948	0.143154	0.42147	137
hsa04510	Focal adhesion	202	0.365805	1.255019	0.244	0.4226	138
hsa00565	Ether lipid metabolism	38	0.383713	1.230264	0.204724	0.423156	139
hsa00750	Vitamin B6 metabolism	6	-0.53356	-1.27354	0.172414	0.423568	140
hsa05031	Amphetamine addiction	61	0.326715	1.267825	0.157447	0.425142	141
hsa00350	Tyrosine metabolism	37	0.399457	1.255833	0.228395	0.425653	142
hsa04910	Insulin signaling pathway	131	0.301193	1.256889	0.143478	0.428589	143
hsa04710	Circadian rhythm	29	0.378328	1.260923	0.154004	0.431588	144

hsa04964	Proximal tubule bicarbonate reclamation	22	0.423958	1.257065	0.180873	0.433142	145
hsa05146	Amoebiasis	103	0.358913	1.192312	0.285421	0.441515	146
hsa00980	Metabolism of xenobiotics by cytochrome P450	55	0.406316	1.19425	0.291498	0.442431	147
hsa05014	Amyotrophic lateral sclerosis (ALS)	48	0.308158	1.169268	0.219512	0.44264	148
hsa00471	D-Glutamine and D-glutamate metabolism	4	0.638632	1.169919	0.284569	0.445112	149
hsa00472	D-Arginine and D-ornithine metabolism	1	0.881169	1.172062	0.254989	0.445738	150
hsa05323	Rheumatoid arthritis	83	0.419827	1.172969	0.345776	0.448029	151
hsa05217	Basal cell carcinoma	54	0.356621	1.179643	0.268398	0.448808	152
hsa04068	FoxO signaling pathway	125	0.294247	1.177096	0.243697	0.448866	153
hsa00072	Synthesis and degradation of ketone bodies	9	0.483392	1.184749	0.273256	0.449095	154
hsa05030	Cocaine addiction	46	0.335297	1.174404	0.259657	0.449353	155
hsa04142	Lysosome	120	0.363996	1.162162	0.326255	0.449493	156
hsa04320	Dorso-ventral axis formation	22	0.405645	1.181574	0.263158	0.449833	157
hsa00604	Glycosphingolipid biosynthesis - ganglio series	15	0.458573	1.153624	0.311637	0.458213	158
hsa00120	Primary bile acid biosynthesis	15	0.44705	1.150055	0.315789	0.46009	159
hsa05211	Renal cell carcinoma	66	0.308171	1.142744	0.25567	0.463729	160
hsa05145	Toxoplasmosis	115	0.357381	1.144843	0.35259	0.46418	161
hsa04915	Estrogen signaling pathway	94	0.289233	1.138877	0.247401	0.465841	162
hsa01040	Biosynthesis of unsaturated fatty acids	19	0.415111	1.12344	0.329365	0.466522	163
hsa04672	Intestinal immune network for IgA production	43	0.48328	1.118426	0.397661	0.466766	164
hsa00061	Fatty acid biosynthesis	6	0.519907	1.120687	0.338947	0.466895	165
hsa00920	Sulfur metabolism	10	0.482955	1.125281	0.350598	0.467517	166
hsa00450	Selenocompound metabolism	16	0.371758	1.130011	0.295499	0.46792	167
hsa04978	Mineral absorption	45	0.35436	1.127273	0.322772	0.468349	168
hsa00600	Sphingolipid metabolism	38	0.329273	1.130825	0.285714	0.470427	169
hsa05120	Epithelial cell signaling in Helicobacter pylori infection	66	0.304968	1.132754	0.292017	0.470979	170
hsa04151	PI3K-Akt signaling pathway	305	0.276081	1.110944	0.314	0.473823	171
hsa00360	Phenylalanine metabolism	17	0.381776	1.085649	0.391732	0.504558	172
hsa00500	Starch and sucrose metabolism	36	0.319712	1.087973	0.328482	0.50458	173
hsa04973	Carbohydrate digestion and absorption	36	0.353932	1.075876	0.371951	0.51539	174
hsa04130	SNARE interactions in vesicular transport	34	0.318619	1.066989	0.354528	0.517715	175
hsa05200	Pathways in cancer	313	0.249897	1.064498	0.358871	0.517886	176
hsa04060	Cytokine-cytokine receptor interaction	228	0.337273	1.061525	0.40668	0.51848	177
hsa04370	VEGF signaling pathway	59	0.297832	1.071145	0.357741	0.518843	178
hsa00983	Drug metabolism - other enzymes	31	-0.35427	-1.2064	0.209677	0.520229	179
hsa00562	Inositol phosphate metabolism	60	0.306967	1.067096	0.362473	0.521263	180
hsa00030	Pentose phosphate pathway	26	-0.39257	-1.17298	0.268924	0.555399	181
hsa05169	Epstein-Barr virus infection	195	-0.28836	-1.17775	0.234927	0.56027	182
hsa05166	HTLV-I infection	252	0.258504	1.017934	0.447581	0.573823	183
hsa04918	Thyroid hormone synthesis	66	0.266767	1.02223	0.417373	0.574956	184

hsa05100	Bacterial invasion of epithelial cells	74	0.301272	1.019564	0.422	0.574995	185
hsa00563	Glycosylphosphatidylinositol(GPI)-anchor biosynthesis	25	-0.4146	-1.13212	0.34127	0.578025	186
hsa00532	Glycosaminoglycan biosynthesis - chondroitin sulfate / dermatan sulfate	20	-0.42054	-1.13498	0.317269	0.58608	187
hsa05210	Colorectal cancer	62	0.266533	0.99555	0.445629	0.592285	188
hsa04066	HIF-1 signaling pathway	102	0.25612	0.996841	0.451485	0.594268	189
hsa05202	Transcriptional misregulation in cancer	159	0.25187	0.999181	0.452479	0.594681	190
hsa04919	Thyroid hormone signaling pathway	115	0.256292	1.001391	0.426724	0.595246	191
hsa04950	Maturity onset diabetes of the young	16	-0.40994	-1.13585	0.309886	0.5983	192
hsa05221	Acute myeloid leukemia	57	0.28603	0.988558	0.456432	0.599109	193
hsa00520	Amino sugar and nucleotide sugar metabolism	47	-0.34822	-1.11101	0.347732	0.603755	194
hsa05034	Alcoholism	139	-0.27649	-1.14029	0.297087	0.60456	195
hsa04012	ErbB signaling pathway	87	0.250573	0.976827	0.451754	0.613309	196
hsa04974	Protein digestion and absorption	75	0.283395	0.973985	0.471074	0.613612	197
hsa04914	Progesterone-mediated oocyte maturation	82	-0.27543	-1.08696	0.332024	0.63421	198
hsa03010	Ribosome	128	-0.4754	-1.0687	0.467641	0.654116	199
hsa04330	Notch signaling pathway	47	0.253639	0.926526	0.569593	0.660906	200
hsa00524	Butirosin and neomycin biosynthesis	5	0.465602	0.936426	0.547619	0.662727	201
hsa04150	mTOR signaling pathway	58	0.241157	0.927465	0.587992	0.6636	202
hsa04721	Synaptic vesicle cycle	57	0.251354	0.929681	0.535433	0.664445	203
hsa04612	Antigen processing and presentation	67	0.337711	0.936891	0.526214	0.666383	204
hsa04512	ECM-receptor interaction	85	0.307986	0.930962	0.531828	0.666824	205
hsa00430	Taurine and hypotaurine metabolism	9	0.367526	0.908608	0.575758	0.680407	206
hsa04940	Type I diabetes mellitus	39	0.387736	0.909217	0.591716	0.683658	207
hsa00514	Other types of O-glycan biosynthesis	26	-0.31793	-1.04509	0.410405	0.685065	208
hsa00340	Histidine metabolism	27	0.286831	0.889937	0.593688	0.700812	209
hsa04668	TNF signaling pathway	110	0.267907	0.892455	0.552326	0.701184	210
hsa05164	Influenza A	151	0.247783	0.881811	0.580078	0.704708	211
hsa00640	Propanoate metabolism	32	0.304887	0.878835	0.603272	0.705145	212
hsa05110	Vibrio cholerae infection	51	-0.28073	-1.01226	0.430279	0.705699	213
hsa04966	Collecting duct acid secretion	25	0.309932	0.881994	0.618661	0.708746	214
hsa00410	beta-Alanine metabolism	29	0.259909	0.872771	0.638132	0.710552	215
hsa00260	Glycine, serine and threonine metabolism	37	0.252437	0.869353	0.619691	0.711848	216
hsa00785	Lipoic acid metabolism	3	-0.66369	-1.02332	0.501044	0.712458	217
hsa00310	Lysine degradation	42	-0.28603	-0.99315	0.449331	0.714169	218
hsa00534	Glycosaminoglycan biosynthesis - heparan sulfate / heparin	23	-0.35518	-0.99968	0.462626	0.715489	219
hsa04623	Cytosolic DNA-sensing pathway	47	-0.28967	-1.01315	0.405797	0.717989	220
hsa04122	Sulfur relay system	10	-0.43032	-0.97074	0.504149	0.741467	221
hsa05131	Shigellosis	59	0.244306	0.843288	0.655706	0.744512	222

hsa05216	Thyroid cancer	29	0.246822	0.844084	0.690229	0.747625	223
hsa05016	Huntington,s disease	169	-0.29353	-0.95776	0.505285	0.752519	224
hsa05213	Endometrial cancer	52	0.225361	0.83503	0.708423	0.752589	225
hsa05130	Pathogenic Escherichia coli infection	52	-0.2716	-0.89199	0.591195	0.755688	226
hsa04660	T cell receptor signaling pathway	101	0.259257	0.829528	0.636	0.756702	227
hsa00561	Glycerolipid metabolism	45	-0.23892	-0.9018	0.625	0.762548	228
hsa00130	Ubiquinone and other terpenoid-quinone biosynthesis	10	-0.39498	-0.89238	0.613779	0.767079	229
hsa00603	Glycosphingolipid biosynthesis - globo series	14	-0.32701	-0.87812	0.622363	0.767828	230
hsa00480	Glutathione metabolism	47	-0.29118	-0.91209	0.538776	0.769529	231
hsa00040	Pentose and glucuronate interconversions	18	-0.31359	-0.90422	0.573965	0.771045	232
hsa05219	Bladder cancer	37	-0.25236	-0.86092	0.686888	0.773935	233
hsa00010	Glycolysis / Gluconeogenesis	60	-0.24522	-0.91646	0.561224	0.774612	234
hsa00730	Thiamine metabolism	3	-0.46766	-0.86601	0.661734	0.776977	235
hsa04210	Apoptosis	82	0.229134	0.808983	0.703422	0.779106	236
hsa05218	Melanoma	61	0.224564	0.811136	0.705285	0.780668	237
hsa00860	Porphyrin and chlorophyll metabolism	27	-0.32744	-0.91907	0.551579	0.783056	238
hsa05223	Non-small cell lung cancer	56	0.213827	0.801456	0.764831	0.785674	239
hsa00740	Riboflavin metabolism	10	-0.36859	-0.92012	0.549894	0.794605	240
hsa05206	MicroRNAs in cancer	149	-0.25429	-0.92564	0.559289	0.798197	241
hsa04662	B cell receptor signaling pathway	71	0.249581	0.788164	0.651303	0.801383	242
hsa05160	Hepatitis C	113	0.193064	0.782085	0.799591	0.806125	243
hsa05222	Small cell lung cancer	85	0.212612	0.773496	0.747012	0.809895	244
hsa00100	Steroid biosynthesis	17	0.296708	0.775624	0.665979	0.811125	245
hsa04146	Peroxisome	77	0.234061	0.765471	0.732422	0.816533	246
hsa05134	Legionellosis	54	0.239081	0.755587	0.750469	0.817311	247
hsa04620	Toll-like receptor signaling pathway	90	0.23859	0.760984	0.700587	0.818216	248
hsa00650	Butanoate metabolism	22	0.26293	0.756341	0.763747	0.820787	249
hsa05220	Chronic myeloid leukemia	73	0.200081	0.73386	0.86875	0.834307	250
hsa00460	Cyanoamino acid metabolism	7	0.318922	0.738943	0.778689	0.836676	251
hsa00280	Valine, leucine and isoleucine degradation	44	0.251091	0.734762	0.759921	0.837556	252
hsa05214	Glioma	63	0.192903	0.721352	0.893528	0.846103	253
hsa00900	Terpenoid backbone biosynthesis	21	0.262919	0.716531	0.790291	0.847591	254
hsa05162	Measles	119	0.207228	0.701445	0.779468	0.857544	255
hsa04140	Regulation of autophagy	23	0.213649	0.70348	0.872299	0.85959	256
hsa00062	Fatty acid elongation	21	0.243381	0.692309	0.819802	0.864297	257
hsa05161	Hepatitis B	130	-0.21118	-0.79673	0.750503	0.867996	258
hsa00232	Caffeine metabolism	5	-0.3805	-0.77998	0.741874	0.869394	259
hsa05322	Systemic lupus erythematosus	97	-0.23778	-0.78316	0.665971	0.876961	260
hsa00190	Oxidative phosphorylation	114	-0.29747	-0.75408	0.66457	0.896903	261
hsa00053	Ascorbate and aldarate metabolism	12	0.249329	0.649682	0.895299	0.906211	262
hsa05168	Herpes simplex infection	155	0.171484	0.635811	0.904398	0.914927	263
hsa05212	Pancreatic cancer	66	0.170493	0.62567	0.956863	0.919533	264
hsa00620	Pyruvate metabolism	38	-0.23629	-0.72942	0.796781	0.921127	265

hsa05012	Parkinson,s disease	124	-0.24933	-0.71746	0.677686	0.926065	266
hsa05033	Nicotine addiction	24	0.205132	0.597459	0.946121	0.933211	267
hsa00780	Biotin metabolism	3	0.350878	0.60228	0.910646	0.934301	268
hsa05215	Prostate cancer	87	-0.17991	-0.69845	0.906796	0.939796	269
hsa00770	Pantothenate and CoA biosynthesis	17	-0.22256	-0.68167	0.881607	0.949057	270
hsa05010	Alzheimer,s disease	154	-0.19755	-0.67054	0.745935	0.949706	271
hsa00630	Glyoxylate and dicarboxylate metabolism	21	0.199025	0.535013	0.957317	0.965172	272
hsa00052	Galactose metabolism	27	-0.21072	-0.64165	0.900208	0.970006	273
hsa00511	Other glycan degradation	17	0.187031	0.504435	0.962891	0.972692	274
hsa04064	NF-kappa B signaling pathway	88	-0.16587	-0.5082	0.961145	0.985092	275
hsa00300	Lysine biosynthesis	2	-0.27459	-0.41357	0.997955	0.993772	276
hsa00290	Valine, leucine and isoleucine biosynthesis	4	-0.282	-0.51587	0.962882	0.994309	277
hsa00760	Nicotinate and nicotinamide metabolism	22	-0.18335	-0.57854	0.964637	1	278
hsa05340	Primary immunodeficiency	33	-0.22095	-0.56145	0.851464	1	278
hsa04622	RIG-I-like receptor signaling pathway	52	-0.14553	-0.51857	0.993789	1	278
hsa04932	Non-alcoholic fatty liver disease (NAFLD)	138	-0.15016	-0.51844	0.962343	1	278
