# Supplementary material for: A network-based pathway-extending approach using DNA methylation and gene expression data to identify altered pathways
Source: Sci Rep. 2019 Aug 14;9:11853. doi: 10.1038/s41598-019-48372-1 (PMC6694157; doi:10.1038/s41598-019-48372-1)
Supplement: Supplementary file 5 — Supplementary Table S5 [file 41598_2019_48372_MOESM5_ESM.pdf]

A network-based pathway-extending approach using DNA methylation and gene expression data to identify altered pathways

Jie Li[1], Qiaosheng Zhang[1,2,*], Zhuo Chen[1], Dechen Xu[1], and Yadong Wang[1]

[1]Harbin Institute of Technology, School of Computer Science and Technology, Harbin, 150001, P.R. China

[2]Heilongjiang Bayi Agricultural University, College of Science, Daqing, 163319, P.R. China

[*]zqs@hit.edu.cn

All results in COAD dataset by EP-ORA

Pathway ID	Pathway Name	Universe.Size	Gene.Set.Size	Total.Hits	Expected.Hits	Observed.Hits	Pvalue	Adjusted.Pvalue	Rank
hsa05206	MicroRNAs in cancer	17577	421	10265	245.8648	286	2.92E-05	8.17E-03	1
hsa04110	Cell cycle	17577	305	10265	178.1206	208	0.000242	3.39E-02	2
hsa03015	mRNA surveillance pathway	17577	204	10265	119.1364	142	0.000586	5.47E-02	3
hsa05200	Pathways in cancer	17577	834	10265	487.0575	526	0.002708	0.139902	4
hsa05214	Glioma	17577	177	10265	103.3683	121	0.003917	0.139902	4
hsa00190	Oxidative phosphorylatio	17577	265	10265	154.7605	176	0.004274	0.139902	4
hsa03030	DNA replication	17577	90	10265	52.56016	65	0.004536	0.139902	4
hsa04974	Protein digestion and absorption	17577	165	10265	96.3603	113	0.004743	0.139902	4
hsa04932	Non-alcoholic fatty liver disease	17577	364	10265	212.5767	237	0.00479	0.139902	4
hsa03013	RNA transport	17577	348	10265	203.2326	227	0.004997	0.139902	4
hsa00240	Pyrimidine metabolism	17577	234	10265	136.6564	156	0.005528	0.140712	11
hsa05213	Endometrial cancer	17577	139	10265	81.17625	96	0.006068	0.141585	12
hsa00380	Tryptophan metabolism	17577	94	10265	54.89617	67	0.006635	0.14291	13
hsa00020	Citrate cycle (TCA cycle)	17577	78	10265	45.55214	56	0.009895	0.184945	14
hsa05010	Alzheimer,s disease	17577	404	10265	235.9367	259	0.01022	0.184945	14
hsa03020	RNA polymerase	17577	72	10265	42.04813	52	0.010568	0.184945	14
hsa05210	Colorectal cancer	17577	186	10265	108.6243	124	0.012368	0.199796	17
hsa00983	Drug metabolism - other enzymes	17577	74	10265	43.21613	53	0.012844	0.199796	17
hsa05215	Prostate cancer	17577	251	10265	146.5845	164	0.01397	0.205873	19
hsa03008	Ribosome biogenesis in eukaryotes	17577	175	10265	102.2003	116	0.019361	0.2504	20
hsa04020	Calcium signaling pathway	17577	400	10265	233.6007	254	0.020076	0.2504	20
hsa00860	Porphyrin and chlorophyll metabolism	17577	69	10265	40.29613	49	0.020808	0.2504	20
hsa00510	N-Glycan biosynthesis	17577	121	10265	70.66422	82	0.021389	0.2504	20

hsa04115	p53 signaling pathway	17577	169	10265	98.69631	112	0.021463	0.2504	20
hsa04918	Thyroid hormone synthesis	17577	160	10265	93.44029	106	0.025071	0.279741	25
hsa04722	Neurotrophin signaling pathway	17577	323	10265	188.6326	206	0.026702	0.279741	25
hsa01040	Biosynthesis of unsaturated fatty acids	17577	45	10265	26.28008	33	0.027664	0.279741	25
hsa04012	ErbB signaling pathway	17577	235	10265	137.2404	152	0.027974	0.279741	25
hsa04728	Dopaminergic synapse	17577	317	10265	185.1286	202	0.029252	0.282432	29
hsa04114	Oocyte meiosis	17577	278	10265	162.3525	178	0.030907	0.283922	30
hsa05217	Basal cell carcinoma	17577	116	10265	67.74421	78	0.031434	0.283922	30
hsa03430	Mismatch repair	17577	61	10265	35.62411	43	0.035011	0.297065	32
hsa04744	Phototransducti on	17577	61	10265	35.62411	43	0.035011	0.297065	32
hsa00562	Inositol phosphate metabolism	17577	164	10265	95.7763	107	0.042972	0.344366	34
hsa00620	Pyruvate metabolism	17577	114	10265	66.57621	76	0.043267	0.344366	34
hsa04914	Progesterone-mediated oocyte maturation	17577	231	10265	134.9044	148	0.044504	0.344366	34
hsa03440	Homologous recombination	17577	68	10265	39.71212	47	0.045506	0.344366	34
hsa04610	Complement and coagulation cascades	17577	145	10265	84.68026	95	0.047309	0.348595	38
hsa05212	Pancreatic cancer	17577	181	10265	105.7043	117	0.049995	0.355458	39
hsa04070	Phosphatidylino sitol signaling system	17577	222	10265	129.6484	142	0.051408	0.355458	39
hsa04916	Melanogenesis	17577	255	10265	148.9205	162	0.052998	0.355458	39
hsa05219	Bladder cancer	17577	110	10265	64.2402	73	0.053319	0.355458	39
hsa04390	Hippo signaling pathway	17577	368	10265	214.9127	230	0.058927	0.383373	43
hsa00600	Sphingolipid metabolism	17577	80	10265	46.72015	54	0.060244	0.383373	43
hsa05204	Chemical carcinogenesis	17577	119	10265	69.49622	78	0.066613	0.403012	45
hsa04710	Circadian rhythm	17577	74	10265	43.21613	50	0.067391	0.403012	45
hsa04970	Salivary secretion	17577	204	10265	119.1364	130	0.068608	0.403012	45
hsa05221	Acute myeloid leukemia	17577	168	10265	98.11231	108	0.069088	0.403012	45

hsa00062	Fatty acid elongation	17577	52	10265	30.36809	36	0.07247	0.410023	49
hsa00982	Drug metabolism - cytochrome P450	17577	100	10265	58.40018	66	0.073218	0.410023	49
hsa05033	Nicotine addiction	17577	68	10265	39.71212	46	0.075494	0.411683	51
hsa05223	Non-small cell lung cancer	17577	162	10265	94.60829	104	0.076455	0.411683	51
hsa05012	Parkinson,s disease	17577	316	10265	184.5446	197	0.083782	0.44262	53
hsa04512	ECM-receptor interaction	17577	194	10265	113.2964	123	0.088201	0.442889	54
hsa04964	Proximal tubule bicarbonate reclamation	17577	51	10265	29.78409	35	0.088479	0.442889	54
hsa05222	Small cell lung cancer	17577	227	10265	132.5684	143	0.088578	0.442889	54
hsa00232	Caffeine metabolism	17577	8	10265	4.672015	7	0.090585	0.442984	57
hsa00830	Retinol metabolism	17577	85	10265	49.64015	56	0.097184	0.442984	57
hsa05031	Amphetamine addiction	17577	175	10265	102.2003	111	0.099847	0.442984	57
hsa04010	MAPK signaling pathway	17577	558	10265	325.873	341	0.100575	0.442984	57
hsa03040	Spliceosome	17577	271	10265	158.2645	169	0.101393	0.442984	57
hsa00280	Valine, leucine and isoleucine degradation	17577	121	10265	70.66422	78	0.102247	0.442984	57
hsa04921	Oxytocin signaling pathway	17577	376	10265	219.5847	232	0.103456	0.442984	57
hsa00601	Glycosphingolipid biosynthesis - lacto and neolacto series	17577	42	10265	24.52808	29	0.105399	0.442984	57
hsa05016	Huntington,s disease	17577	438	10265	255.7928	269	0.105814	0.442984	57
hsa04978	Mineral absorption	17577	100	10265	58.40018	65	0.106601	0.442984	57
hsa04919	Thyroid hormone signaling pathway	17577	318	10265	185.7126	197	0.107411	0.442984	57
hsa04310	Wnt signaling pathway	17577	338	10265	197.3926	209	0.107582	0.442984	57
hsa05323	Rheumatoid arthritis	17577	202	10265	117.9684	127	0.109873	0.44586	69
hsa05020	Prion diseases	17577	89	10265	51.97616	58	0.116234	0.464935	70
hsa04810	Regulation of actin cytoskeleton	17577	508	10265	296.6729	310	0.120464	0.47507	71

hsa04960	Aldosterone-regulated sodium reabsorption	17577	96	10265	56.06417	62	0.129116	0.491106	72
hsa00360	Phenylalanine metabolism	17577	49	10265	28.61609	33	0.129221	0.491106	72
hsa05205	Proteoglycans in cancer	17577	524	10265	306.017	319	0.130523	0.491106	72
hsa04976	Bile secretion	17577	147	10265	85.84827	93	0.131546	0.491106	72
hsa00460	Cyanoamino acid metabolism	17577	16	10265	9.344029	12	0.136144	0.501582	76
hsa00730	Thiamine metabolism	17577	7	10265	4.088013	6	0.138643	0.504155	77
hsa00670	One carbon pool by folate	17577	59	10265	34.45611	39	0.142025	0.504418	78
hsa00480	Glutathione metabolism	17577	108	10265	63.0722	69	0.143718	0.504418	78
hsa04977	Vitamin digestion and absorption	17577	51	10265	29.78409	34	0.144939	0.504418	78
hsa04080	Neuroactive ligand-receptor interaction	17577	391	10265	228.3447	239	0.145921	0.504418	78
hsa04950	Maturity onset diabetes of the young	17577	53	10265	30.9521	35	0.161072	0.550003	82
hsa04261	Adrenergic signaling in cardiomyocytes	17577	337	10265	196.8086	206	0.166095	0.557119	83
hsa00270	Cysteine and methionine metabolism	17577	94	10265	54.89617	60	0.167136	0.557119	83
hsa03022	Basal transcription factors	17577	104	10265	60.73619	66	0.171068	0.558524	85
hsa05134	Legionellosis	17577	152	10265	88.76828	95	0.171867	0.558524	85
hsa05211	Renal cell carcinoma	17577	172	10265	100.4483	107	0.173542	0.558524	85
hsa03018	RNA degradation	17577	159	10265	92.85629	99	0.181049	0.563237	88
hsa05220	Chronic myeloid	17577	204	10265	119.1364	126	0.181809	0.563237	88
hsa00750	Vitamin B6 metabolism	17577	15	10265	8.760027	11	0.181978	0.563237	88
hsa03450	Non-homologous end-joining	17577	39	10265	22.77607	26	0.188491	0.563237	88
hsa05146	Amoebiasis	17577	263	10265	153.5925	161	0.192204	0.563237	88
hsa00230	Purine metabolism	17577	379	10265	221.3367	230	0.195094	0.563237	88
hsa05216	Thyroid cancer	17577	85	10265	49.64015	54	0.197795	0.563237	88
hsa04320	Dorso-ventral axis formation	17577	62	10265	36.20811	40	0.198401	0.563237	88

hsa05169	Epstein-Barr virus infection	17577	541	10265	315.945	326	0.198808	0.563237	88
hsa04750	Inflammatory mediator regulation of TRP channels	17577	240	10265	140.1604	147	0.201917	0.563237	88
hsa00053	Ascorbate and aldarate metabolism	17577	36	10265	21.02407	24	0.201986	0.563237	88
hsa00532	Glycosaminoglycan biosynthesis - chondroitin sulfate / dermatan	17577	36	10265	21.02407	24	0.201986	0.563237	88
hsa00512	Mucin type O-Glycan biosynthesis	17577	49	10265	28.61609	32	0.20211	0.563237	88
hsa00533	Glycosaminoglycan biosynthesis -	17577	28	10265	16.35205	19	0.206178	0.563237	88
hsa05133	Pertussis	17577	190	10265	110.9603	117	0.206575	0.563237	88
hsa04920	Adipocytokine signaling pathway	17577	180	10265	105.1203	111	0.207191	0.563237	88
hsa00410	beta-Alanine metabolism	17577	82	10265	47.88815	52	0.209349	0.563632	104
hsa00630	Glyoxylate and dicarboxylate metabolism	17577	69	10265	40.29613	44	0.21739	0.579707	105
hsa05142	Chagas disease (American trypanosomiasis)	17577	271	10265	158.2645	165	0.219752	0.580476	106
hsa03460	Fanconi anemia pathway	17577	94	10265	54.89617	59	0.225618	0.581697	107
hsa03420	Nucleotide excision repair	17577	104	10265	60.73619	65	0.227133	0.581697	107
hsa00310	Lysine degradation	17577	109	10265	63.6562	68	0.227638	0.581697	107
hsa04145	Phagosome	17577	327	10265	190.9686	198	0.230186	0.581697	107
hsa04210	Apoptosis	17577	206	10265	120.3044	126	0.230601	0.581697	107
hsa04015	Rap1 signaling pathway	17577	496	10265	289.6649	298	0.234893	0.587231	112
hsa05218	Melanoma	17577	156	10265	91.10428	96	0.237354	0.588133	113
hsa00072	Synthesis and degradation of ketone bodies	17577	22	10265	12.84804	15	0.239679	0.588686	114
hsa04917	Prolactin signaling pathway	17577	178	10265	103.9523	109	0.244237	0.592043	115
hsa04150	mTOR signaling pathway	17577	148	10265	86.43227	91	0.248709	0.592043	115

hsa00350	Tyrosine metabolism	17577	83	10265	48.47215	52	0.250758	0.592043	115
hsa04961	Endocrine and other factor-regulated calcium reabsorption	17577	128	10265	74.75223	79	0.25093	0.592043	115
hsa00603	Glycosphingolipid biosynthesis - globo series	17577	27	10265	15.76805	18	0.251618	0.592043	115
hsa05412	Arrhythmogenic right ventricular cardiomyopathy (ARVC)	17577	175	10265	102.2003	107	0.254572	0.594001	120
hsa00563	Glycosylphosphatidylinositol(GPI)-anchor biosynthesis	17577	55	10265	32.1201	35	0.258802	0.598511	121
hsa05166	HTLV-I	17577	650	10265	379.6012	388	0.261335	0.598511	121
hsa00590	Arachidonic acid metabolism	17577	125	10265	73.00023	77	0.263009	0.598511	121
hsa05150	Staphylococcus aureus infection	17577	105	10265	61.32019	65	0.265055	0.598511	121
hsa05152	Tuberculosis	17577	405	10265	236.5207	243	0.271571	0.608319	125
hsa05120	Epithelial cell signaling in Helicobacter pylori infection	17577	179	10265	104.5363	109	0.27382	0.608489	126
hsa04971	Gastric acid secretion	17577	169	10265	98.69631	103	0.276417	0.609424	127
hsa04640	Hematopoietic cell lineage	17577	191	10265	111.5443	116	0.280609	0.613832	128
hsa00400	Phenylalanine, tyrosine and tryptophan biosynthesis	17577	8	10265	4.672015	6	0.282816	0.613864	129
hsa04913	Ovarian steroidogenesis	17577	104	10265	60.73619	64	0.292089	0.620467	130
hsa00010	Glycolysis / Gluconeogenesis	17577	178	10265	103.9523	108	0.294913	0.620467	130
hsa00920	Sulfur metabolism	17577	21	10265	12.26404	14	0.295685	0.620467	130
hsa00650	Butanoate metabolism	17577	59	10265	34.45611	37	0.296349	0.620467	130
hsa04972	Pancreatic secretion	17577	222	10265	129.6484	134	0.299808	0.620467	130
hsa00970	Aminoacyl-tRNA biosynthesis	17577	121	10265	70.66422	74	0.301236	0.620467	130
hsa04530	Tight junction	17577	310	10265	181.0406	186	0.302909	0.620467	130
hsa04510	Focal adhesion	17577	510	10265	297.8409	304	0.303586	0.620467	130

hsa00250	Alanine, aspartate and glutamate metabolism	17577	91	10265	53.14417	56	0.309406	0.620911	138
hsa04151	PI3K-Akt signaling pathway	17577	755	10265	440.9214	448	0.310289	0.620911	138
hsa05164	Influenza A	17577	402	10265	234.7687	240	0.314849	0.620911	138
hsa04066	HIF-1 signaling pathway	17577	275	10265	160.6005	165	0.316266	0.620911	138
hsa04022	cGMP-PKG signaling pathway	17577	397	10265	231.8487	237	0.316733	0.620911	138
hsa04962	Vasopressin-regulated water reabsorption	17577	113	10265	65.99221	69	0.317108	0.620911	138
hsa04370	VEGF signaling pathway	17577	162	10265	94.60829	98	0.322968	0.626119	144
hsa04660	T cell receptor signaling pathway	17577	277	10265	161.7685	166	0.324284	0.626119	144
hsa04912	GnRH signaling pathway	17577	228	10265	133.1524	137	0.326476	0.626119	144
hsa05320	Autoimmune thyroid disease	17577	73	10265	42.63213	45	0.330443	0.626544	147
hsa05310	Asthma	17577	63	10265	36.79211	39	0.333223	0.626544	147
hsa04122	Sulfur relay system	17577	28	10265	16.35205	18	0.333411	0.626544	147
hsa00040	Pentose and glucuronate interconversion	17577	43	10265	25.11208	27	0.336483	0.627458	150
hsa04670	Leukocyte transendothelial migration	17577	259	10265	151.2565	155	0.341284	0.627458	150
hsa05160	Hepatitis C	17577	310	10265	181.0406	185	0.344775	0.627458	150
hsa00980	Metabolism of xenobiotics by cytochrome P450	17577	112	10265	65.4082	68	0.345478	0.627458	150
hsa05014	Amyotrophic lateral sclerosis (ALS)	17577	139	10265	81.17625	84	0.345591	0.627458	150
hsa03320	PPAR signaling pathway	17577	183	10265	106.8723	110	0.347343	0.627458	150
hsa00260	Glycine, serine and threonine metabolism	17577	114	10265	66.57621	69	0.358704	0.641365	156
hsa00430	Taurine and hypotaurine metabolism	17577	20	10265	11.68004	13	0.359622	0.641365	156
hsa04910	Insulin signaling pathway	17577	355	10265	207.3206	211	0.365719	0.644106	158
hsa04068	FoxO signaling pathway	17577	372	10265	217.2487	221	0.36576	0.644106	158

hsa04672	Intestinal immune network for IgA production	17577	101	10265	58.98418	61	0.381498	0.667143	160
hsa04614	Renin-angiotensin system	17577	42	10265	24.52808	26	0.383607	0.667143	160
hsa04975	Fat digestion and absorption	17577	81	10265	47.30415	49	0.395858	0.684199	162
hsa05202	Transcriptional misregulation in cancer	17577	428	10265	249.9528	253	0.401149	0.685813	163
hsa03410	Base excision repair	17577	93	10265	54.31217	56	0.403293	0.685813	163
hsa04713	Circadian entrainment	17577	234	10265	136.6564	139	0.40414	0.685813	163
hsa04620	Toll-like receptor signaling pathway	17577	229	10265	133.7364	136	0.407324	0.687052	166
hsa00511	Other glycan degradation	17577	51	10265	29.78409	31	0.422432	0.70033	167
hsa04966	Collecting duct acid secretion	17577	51	10265	29.78409	31	0.422432	0.70033	167
hsa05144	Malaria	17577	124	10265	72.41623	74	0.423418	0.70033	167
hsa04260	Cardiac muscle contraction	17577	148	10265	86.43227	88	0.430838	0.70033	167
hsa04144	Endocytosis	17577	501	10265	292.5849	295	0.431038	0.70033	167
hsa04152	AMPK signaling pathway	17577	327	10265	190.9686	193	0.432337	0.70033	167
hsa04720	Long-term potentiation	17577	160	10265	93.44029	95	0.433942	0.70033	167
hsa04650	Natural killer cell mediated cytotoxicity	17577	264	10265	154.1765	156	0.435205	0.70033	167
hsa05322	Systemic lupus erythematosus	17577	201	10265	117.3844	119	0.437757	0.700411	175
hsa04623	Cytosolic DNA-sensing pathway	17577	116	10265	67.74421	69	0.445278	0.708397	176
hsa04727	GABAergic synapse	17577	186	10265	108.6243	110	0.449525	0.711113	177
hsa04360	Axon guidance	17577	292	10265	170.5285	172	0.454985	0.712776	178
hsa00561	Glycerolipid metabolism	17577	118	10265	68.91221	70	0.458213	0.712776	178
hsa00071	Fatty acid degradation	17577	118	10265	68.91221	70	0.458213	0.712776	178
hsa00592	alpha-Linolenic acid metabolism	17577	55	10265	32.1201	33	0.461633	0.714128	181
hsa00140	Steroid hormone biosynthesis	17577	79	10265	46.13614	47	0.469388	0.722135	182
hsa05162	Measles	17577	320	10265	186.8806	188	0.472971	0.723671	183

hsa04662	B cell receptor signaling pathway	17577	192	10265	112.1283	113	0.479797	0.730127	184
hsa04270	Vascular smooth muscle contraction	17577	276	10265	161.1845	162	0.485847	0.735336	185
hsa05130	Pathogenic Escherichia coli infection	17577	136	10265	79.42425	80	0.496652	0.747649	186
hsa04064	NF-kappa B signaling pathway	17577	220	10265	128.4804	129	0.500426	0.749302	187
hsa04911	Insulin secretion	17577	191	10265	111.5443	112	0.504227	0.750977	188
hsa05203	Viral carcinogenesis	17577	508	10265	296.6729	297	0.507262	0.7515	189
hsa05340	Primary immunodeficiency	17577	85	10265	49.64015	50	0.514769	0.75523	190
hsa00052	Galactose metabolism	17577	73	10265	42.63213	43	0.515175	0.75523	190
hsa00514	Other types of O-glycan biosynthesis	17577	56	10265	32.7041	33	0.525089	0.761893	192
hsa04721	Synaptic vesicle cycle	17577	147	10265	85.84827	86	0.525162	0.761893	192
hsa05168	Herpes simplex infection	17577	396	10265	231.2647	231	0.532512	0.768574	194
hsa00740	Riboflavin metabolism	17577	27	10265	15.76805	16	0.545921	0.783887	195
hsa04666	Fc gamma R-mediated phagocytosis	17577	237	10265	138.4084	138	0.549367	0.78481	196
hsa04725	Cholinergic synapse	17577	263	10265	153.5925	153	0.556076	0.78674	197
hsa00534	Glycosaminoglycan biosynthesis - heparan sulfate	17577	41	10265	23.94407	24	0.559355	0.78674	197
hsa05161	Hepatitis B	17577	380	10265	221.9207	221	0.560491	0.78674	197
hsa00500	Starch and sucrose metabolism	17577	98	10265	57.23218	57	0.561957	0.78674	197
hsa04014	Ras signaling pathway	17577	513	10265	299.5929	298	0.576347	0.800408	201
hsa04668	TNF signaling pathway	17577	300	10265	175.2005	174	0.580797	0.800408	201
hsa00640	Propanoate metabolism	17577	95	10265	55.48017	55	0.583154	0.800408	201
hsa05143	African trypanosomiasis	17577	95	10265	55.48017	55	0.583154	0.800408	201
hsa00531	Glycosaminoglycan degradation	17577	38	10265	22.19207	22	0.593413	0.810515	205

hsa04621	NOD-like receptor signaling pathway	17577	137	10265	80.00825	79	0.605184	0.82258	206
hsa04140	Regulation of autophagy	17577	73	10265	42.63213	42	0.608444	0.823016	207
hsa04940	Type I diabetes mellitus	17577	87	10265	50.80816	50	0.614312	0.826958	208
hsa04724	Glutamatergic synapse	17577	255	10265	148.9205	147	0.622798	0.830588	209
hsa05110	Vibrio cholerae infection	17577	134	10265	78.25624	77	0.622941	0.830588	209
hsa04726	Serotonergic synapse	17577	257	10265	150.0885	148	0.630435	0.836596	211
hsa04062	Chemokine signaling pathway	17577	430	10265	251.1208	248	0.640917	0.846494	212
hsa04740	Olfactory transduction	17577	72	10265	42.04813	41	0.646659	0.850068	213
hsa04915	Estrogen signaling pathway	17577	258	10265	150.6725	148	0.657805	0.854612	214
hsa05032	Morphine addiction	17577	194	10265	113.2964	111	0.660119	0.854612	214
hsa04350	TGF-beta signaling pathway	17577	220	10265	128.4804	126	0.660288	0.854612	214
hsa04612	Antigen processing and presentation	17577	142	10265	82.92826	81	0.662324	0.854612	214
hsa04514	Cell adhesion molecules (CAMs)	17577	286	10265	167.0245	164	0.666001	0.854629	218
hsa04142	Lysosome	17577	302	10265	176.3685	173	0.676524	0.854629	218
hsa05030	Cocaine addiction	17577	132	10265	77.08824	75	0.67811	0.854629	218
hsa00785	Lipoic acid metabolism	17577	7	10265	4.088013	4	0.678684	0.854629	218
hsa00450	Selenocompound metabolism	17577	41	10265	23.94407	23	0.678819	0.854629	218
hsa04622	RIG-I-like receptor signaling pathway	17577	146	10265	85.26427	83	0.680651	0.854629	218
hsa04060	Cytokine-cytokine receptor interaction	17577	486	10265	283.8249	279	0.691028	0.863785	224
hsa04723	Retrograde endocannabinoid signaling	17577	216	10265	126.1444	123	0.694587	0.864375	225
hsa00780	Biotin metabolism	17577	9	10265	5.256016	5	0.69918	0.866241	226

hsa04730	Long-term depression	17577	145	10265	84.68026	82	0.7058	0.870446	227
hsa03050	Proteasome	17577	112	10265	65.4082	63	0.713178	0.870446	227
hsa04340	Hedgehog signaling pathway	17577	112	10265	65.4082	63	0.713178	0.870446	227
hsa00290	Valine, leucine and isoleucine biosynthesis	17577	11	10265	6.42402	6	0.71711	0.870446	227
hsa05034	Alcoholism	17577	335	10265	195.6406	191	0.718118	0.870446	227
hsa04973	Carbohydrate digestion and absorption	17577	86	10265	50.22416	48	0.726081	0.876305	232
hsa05321	Inflammatory bowel disease (IBD)	17577	139	10265	81.17625	78	0.738188	0.887093	233
hsa05330	Allograft rejection	17577	76	10265	44.38414	42	0.750428	0.894127	234
hsa05332	Graft-versus-host disease	17577	76	10265	44.38414	42	0.750428	0.894127	234
hsa04630	Jak-STAT signaling pathway	17577	283	10265	165.2725	160	0.759105	0.900633	236
hsa04611	Platelet activation	17577	324	10265	189.2166	183	0.777962	0.915822	237
hsa04146	Peroxisome	17577	193	10265	112.7124	108	0.778448	0.915822	237
hsa00330	Arginine and proline metabolism	17577	144	10265	84.09626	80	0.782882	0.917184	239
hsa00520	Amino sugar and nucleotide sugar metabolism	17577	125	10265	73.00023	69	0.794191	0.926556	240
hsa04540	Gap junction	17577	215	10265	125.5604	120	0.800873	0.929124	241
hsa00564	Glycerophosph olipid metabolism	17577	187	10265	109.2083	104	0.803028	0.929124	241
hsa05100	Bacterial invasion of epithelial cells	17577	210	10265	122.6404	117	0.806688	0.929517	243
hsa00790	Folate biosynthesis	17577	38	10265	22.19207	20	0.812892	0.932609	244
hsa05132	Salmonella infection	17577	228	10265	133.1524	127	0.816033	0.932609	244
hsa04930	Type II diabetes mellitus	17577	123	10265	71.83222	67	0.836234	0.938068	246
hsa04141	Protein processing in endoplasmic reticulum	17577	420	10265	245.2808	236	0.836496	0.938068	246
hsa00061	Fatty acid biosynthesis	17577	20	10265	11.68004	10	0.838872	0.938068	246
hsa00100	Steroid biosynthesis	17577	46	10265	26.86408	24	0.843183	0.938068	246

hsa00030	Pentose phosphate pathway	17577	75	10265	43.80014	40	0.843634	0.938068	246
hsa05416	Viral	17577	134	10265	78.25624	73	0.84439	0.938068	246
hsa04520	Adherens junction	17577	198	10265	115.6324	109	0.849429	0.938068	246
hsa00120	Primary bile acid	17577	37	10265	21.60807	19	0.850207	0.938068	246
hsa05145	Toxoplasmosis	17577	295	10265	172.2805	164	0.852161	0.938068	246
hsa04120	Ubiquitin mediated proteolysis	17577	332	10265	193.8886	185	0.854312	0.938068	246
hsa00340	Histidine metabolism	17577	63	10265	36.79211	33	0.863894	0.944884	256
hsa05140	Leishmaniasis	17577	162	10265	94.60829	88	0.87228	0.947826	257
hsa00130	Ubiquinone and other terpenoid-quinone biosynthesis	17577	32	10265	18.68806	16	0.873354	0.947826	257
hsa03060	Protein export	17577	58	10265	33.87211	30	0.877925	0.949108	259
hsa04664	Fc epsilon RI signaling pathway	17577	177	10265	103.3683	96	0.885777	0.953913	260
hsa04742	Taste transduction	17577	64	10265	37.37612	33	0.891781	0.9567	261
hsa00300	Lysine biosynthesis	17577	5	10265	2.920009	2	0.900126	0.958586	262
hsa00591	Linoleic acid metabolism	17577	57	10265	33.2881	29	0.900638	0.958586	262
hsa05414	Dilated cardiomyopathy	17577	196	10265	114.4644	106	0.903885	0.958586	262
hsa00565	Ether lipid metabolism	17577	83	10265	48.47215	43	0.908149	0.958586	262
hsa00604	Glycosphingolipid biosynthesis - ganglio series	17577	37	10265	21.60807	18	0.91408	0.958586	262
hsa00910	Nitrogen metabolism	17577	37	10265	21.60807	18	0.91408	0.958586	262
hsa02010	ABC transporters	17577	111	10265	64.8242	58	0.920832	0.960324	268
hsa00770	Pantothenate and CoA biosynthesis	17577	43	10265	25.11208	21	0.922597	0.960324	268
hsa00471	D-Glutamine and D-glutamate metabolism	17577	12	10265	7.008022	5	0.928106	0.961372	270
hsa03010	Ribosome	17577	337	10265	196.8086	184	0.930829	0.961372	270
hsa05410	Hypertrophic cardiomyopathy (HCM)	17577	177	10265	103.3683	94	0.934235	0.961372	270

hsa00900	Terpenoid backbone biosynthesis	17577	55	10265	32.1201	27	0.937338	0.961372	270
hsa00051	Fructose and mannose metabolism	17577	85	10265	49.64015	43	0.94161	0.962229	274
hsa00760	Nicotinate and nicotinamide metabolism	17577	50	10265	29.20009	24	0.948357	0.9656	275
hsa04130	SNARE interactions in vesicular transport	17577	73	10265	42.63213	36	0.954342	0.968173	276
hsa04330	Notch signaling pathway	17577	117	10265	68.32821	59	0.967142	0.977616	277
hsa04380	Osteoclast differentiation	17577	313	10265	182.7926	165	0.982476	0.988459	278
hsa00524	Butirosin and neomycin biosynthesis	17577	17	10265	9.928031	6	0.984929	0.988459	278
hsa05131	Shigellosis	17577	169	10265	98.69631	82	0.9963	0.9963	280

All results in COAD dataset by ORA

Pathway ID	Pathway Name	Universe.Size	Gene.Set.Size	Total.Hits	Expected.Hits	Observed.Hits	Pvalue	Adjusted.Pvalue	Rank
hsa00190	Oxidative phosphorylatio	17577	112	10265	65.4082	88	5.13E-06	1.44E-03	1
hsa05012	Parkinson,s disease	17577	124	10265	72.41623	95	1.53E-05	0.002147	2
hsa05010	Alzheimer,s disease	17577	155	10265	90.52028	114	5.81E-05	0.005447	3
hsa03030	DNA replication	17577	36	10265	21.02407	31	0.000336	0.023618	4
hsa04932	Non-alcoholic fatty liver disease	17577	140	10265	81.76025	101	0.000496	0.027852	5
hsa04110	Cell cycle	17577	118	10265	68.91221	86	0.000744	0.034836	6
hsa00020	Citrate cycle (TCA cycle)	17577	29	10265	16.93605	25	0.00128	0.051381	7
hsa04914	Progesterone-mediated oocyte maturation	17577	82	10265	47.88815	61	0.001855	0.065172	8
hsa00280	Valine, leucine and isoleucine degradation	17577	44	10265	25.69608	35	0.002608	0.081369	9
hsa05206	MicroRNAs in cancer	17577	148	10265	86.43227	103	0.003156	0.081369	9
hsa03320	PPAR signaling pathway	17577	62	10265	36.20811	47	0.003185	0.081369	9
hsa05214	Glioma	17577	64	10265	37.37612	48	0.004195	0.087544	12
hsa03013	RNA transport	17577	146	10265	85.26427	101	0.004598	0.087544	12
hsa00920	Sulfur metabolism	17577	10	10265	5.840018	10	0.004606	0.087544	12

hsa00071	Fatty acid degradation	17577	39	10265	22.77607	31	0.004673	0.087544	12
hsa03008	Ribosome biogenesis in eukaryotes	17577	69	10265	40.29613	51	0.005344	0.093848	16
hsa04114	Oocyte meiosis	17577	103	10265	60.15219	73	0.005923	0.094567	17
hsa00983	Drug metabolism - other enzymes	17577	35	10265	20.44006	28	0.006058	0.094567	17
hsa03040	Spliceosome	17577	111	10265	64.8242	78	0.00645	0.095387	19
hsa05213	Endometrial cancer	17577	52	10265	30.36809	39	0.009521	0.132403	20
hsa04974	Protein digestion and absorption	17577	78	10265	45.55214	56	0.009895	0.132403	20
hsa00350	Tyrosine metabolism	17577	36	10265	21.02407	28	0.012111	0.151707	22
hsa03430	Mismatch repair	17577	23	10265	13.43204	19	0.012957	0.151707	22
hsa00630	Glyoxylate and dicarboxylate metabolism	17577	23	10265	13.43204	19	0.012957	0.151707	22
hsa03440	Homologous recombination	17577	26	10265	15.18405	21	0.014337	0.154999	25
hsa00230	Purine metabolism	17577	153	10265	89.35228	103	0.014342	0.154999	25
hsa05223	Non-small cell lung cancer	17577	56	10265	32.7041	41	0.015499	0.157574	27
hsa05200	Pathways in cancer	17577	315	10265	183.9606	203	0.015701	0.157574	27
hsa00510	N-Glycan biosynthesis	17577	49	10265	28.61609	36	0.020925	0.18897	29
hsa00410	beta-Alanine metabolism	17577	28	10265	16.35205	22	0.021216	0.18897	29
hsa05212	Pancreatic cancer	17577	66	10265	38.54412	47	0.021672	0.18897	29
hsa03020	RNA polymerase	17577	31	10265	18.10406	24	0.021835	0.18897	29
hsa05219	Bladder cancer	17577	37	10265	21.60807	28	0.022192	0.18897	29
hsa00982	Drug metabolism - cytochrome P450	17577	54	10265	31.5361	39	0.025203	0.204401	34
hsa04666	Fc gamma R-mediated phagocytosis	17577	90	10265	52.56016	62	0.026288	0.204401	34
hsa00830	Retinol metabolism	17577	48	10265	28.03209	35	0.02689	0.204401	34
hsa04012	ErbB signaling pathway	17577	87	10265	50.80816	60	0.027641	0.204401	34
hsa05215	Prostate cancer	17577	87	10265	50.80816	60	0.027641	0.204401	34
hsa04976	Bile secretion	17577	59	10265	34.45611	42	0.029386	0.211731	39
hsa03015	mRNA surveillance pathway	17577	81	10265	47.30415	56	0.030558	0.214666	40

hsa00360	Phenylalanine metabolism	17577	17	10265	9.928031	14	0.034991	0.239819	41
hsa00620	Pyruvate metabolism	17577	38	10265	22.19207	28	0.037723	0.252387	42
hsa04970	Salivary secretion	17577	77	10265	44.96814	53	0.038944	0.25363	43
hsa00052	Galactose metabolism	17577	29	10265	16.93605	22	0.039714	0.25363	43
hsa00500	Starch and sucrose metabolism	17577	43	10265	25.11208	31	0.045356	0.283226	45
hsa04020	Calcium signaling pathway	17577	166	10265	96.9443	108	0.046516	0.284152	46
hsa04960	Aldosterone-regulated sodium reabsorption	17577	37	10265	21.60807	27	0.048801	0.288541	47
hsa05016	Huntington,s disease	17577	168	10265	98.11231	109	0.050235	0.288541	47
hsa04750	Inflammatory mediator regulation of TRP channels	17577	89	10265	51.97616	60	0.051038	0.288541	47
hsa00640	Propanoate metabolism	17577	31	10265	18.10406	23	0.051784	0.288541	47
hsa01040	Biosynthesis of unsaturated fatty acids	17577	19	10265	11.09603	15	0.05276	0.288541	47
hsa04070	Phosphatidylinositol signaling system	17577	78	10265	45.55214	53	0.053396	0.288541	47
hsa03420	Nucleotide excision repair	17577	45	10265	26.28008	32	0.054932	0.291242	53
hsa04080	Neuroactive ligand-receptor interaction	17577	205	10265	119.7204	131	0.061446	0.319747	54
hsa04721	Synaptic vesicle cycle	17577	58	10265	33.87211	40	0.064967	0.327926	55
hsa00561	Glycerolipid metabolism	17577	47	10265	27.44809	33	0.065352	0.327926	55
hsa04115	p53 signaling pathway	17577	66	10265	38.54412	45	0.06662	0.328427	57
hsa00040	Pentose and glucuronate interconversion	17577	24	10265	14.01604	18	0.071708	0.341682	58
hsa04614	Renin-angiotensin system	17577	15	10265	8.760027	12	0.071833	0.341682	58
hsa04744	Phototransduction	17577	21	10265	12.26404	16	0.072957	0.341682	58
hsa04360	Axon guidance	17577	125	10265	73.00023	81	0.085173	0.387289	61
hsa04740	Olfactory transduction	17577	43	10265	25.11208	30	0.085452	0.387289	61

hsa00980	Metabolism of xenobiotics by cytochrome P450	17577	59	10265	34.45611	40	0.089794	0.39433	63
hsa00240	Pyrimidine metabolism	17577	96	10265	56.06417	63	0.089812	0.39433	63
hsa04640	Hematopoietic cell lineage	17577	80	10265	46.72015	53	0.093446	0.403976	65
hsa05031	Amphetamine addiction	17577	64	10265	37.37612	43	0.095412	0.406223	66
hsa04977	Vitamin digestion and absorption	17577	20	10265	11.68004	15	0.098137	0.407506	67
hsa04975	Fat digestion and absorption	17577	34	10265	19.85606	24	0.10074	0.407506	67
hsa00533	Glycosaminoglycan biosynthesis -	17577	14	10265	8.176025	11	0.101205	0.407506	67
hsa04972	Pancreatic secretion	17577	82	10265	47.88815	54	0.102964	0.407506	67
hsa04210	Apoptosis	17577	82	10265	47.88815	54	0.102964	0.407506	67
hsa05033	Nicotine addiction	17577	31	10265	18.10406	22	0.10636	0.415101	72
hsa04720	Long-term potentiation	17577	63	10265	36.79211	42	0.113206	0.427137	73
hsa05204	Chemical carcinogenesis	17577	63	10265	36.79211	42	0.113206	0.427137	73
hsa04978	Mineral absorption	17577	47	10265	27.44809	32	0.114005	0.427137	73
hsa00270	Cysteine and methionine metabolism	17577	36	10265	21.02407	25	0.118714	0.438929	76
hsa04964	Proximal tubule bicarbonate reclamation	17577	22	10265	12.84804	16	0.124412	0.454022	77
hsa00860	Porphyrin and chlorophyll metabolism	17577	33	10265	19.27206	23	0.126082	0.454219	78
hsa04728	Dopaminergic synapse	17577	124	10265	72.41623	79	0.132685	0.457577	79
hsa05218	Melanoma	17577	62	10265	36.20811	41	0.133528	0.457577	79
hsa04260	Cardiac muscle contraction	17577	62	10265	36.20811	41	0.133528	0.457577	79
hsa05210	Colorectal cancer	17577	62	10265	36.20811	41	0.133528	0.457577	79
hsa00600	Sphingolipid metabolism	17577	38	10265	22.19207	26	0.137385	0.460383	83
hsa03460	Fanconi anemia pathway	17577	46	10265	26.86408	31	0.137623	0.460383	83
hsa04610	Complement and coagulation cascades	17577	59	10265	34.45611	39	0.142025	0.461171	85
hsa04122	Sulfur relay system	17577	10	10265	5.840018	8	0.142783	0.461171	85

hsa00740	Riboflavin metabolism	17577	10	10265	5.840018	8	0.142783	0.461171	85
hsa00970	Aminoacyl-tRNA biosynthesis	17577	43	10265	25.11208	29	0.146718	0.464821	88
hsa04916	Melanogenesis	17577	95	10265	55.48017	61	0.147221	0.464821	88
hsa00601	Glycosphingolipid biosynthesis - lacto and neolacto series	17577	24	10265	14.01604	17	0.151453	0.472871	90
hsa04146	Peroxisome	17577	79	10265	46.13614	51	0.159055	0.476481	91
hsa04014	Ras signaling pathway	17577	208	10265	121.4724	129	0.159995	0.476481	91
hsa04066	HIF-1 signaling pathway	17577	102	10265	59.56819	65	0.16022	0.476481	91
hsa04950	Maturity onset diabetes of the young	17577	21	10265	12.26404	15	0.161088	0.476481	91
hsa00062	Fatty acid elongation	17577	21	10265	12.26404	15	0.161088	0.476481	91
hsa04022	cGMP-PKG signaling pathway	17577	155	10265	90.52028	97	0.163892	0.479727	96
hsa00010	Glycolysis / Gluconeogenesis	17577	60	10265	35.04011	39	0.182403	0.528405	97
hsa05323	Rheumatoid arthritis	17577	83	10265	48.47215	53	0.184663	0.529433	98
hsa05120	Epithelial cell signaling in Helicobacter pylori infection	17577	65	10265	37.96012	42	0.186526	0.529433	98
hsa03450	Non-homologous end-joining	17577	12	10265	7.008022	9	0.192796	0.534362	100
hsa05221	Acute myeloid leukemia	17577	57	10265	33.2881	37	0.194269	0.534362	100
hsa05222	Small cell lung cancer	17577	85	10265	49.64015	54	0.197795	0.534362	100
hsa00564	Glycerophospholipid metabolism	17577	85	10265	49.64015	54	0.197795	0.534362	100
hsa00730	Thiamine metabolism	17577	3	10265	1.752005	3	0.199154	0.534362	100
hsa04713	Circadian entrainment	17577	90	10265	52.56016	57	0.199673	0.534362	100
hsa05217	Basal cell carcinoma	17577	54	10265	31.5361	35	0.20709	0.540943	106
hsa04911	Insulin secretion	17577	77	10265	44.96814	49	0.207266	0.540943	106
hsa04921	Oxytocin signaling pathway	17577	145	10265	84.68026	90	0.207907	0.540943	106

hsa04060	Cytokine-cytokine receptor interaction	17577	227	10265	132.5684	139	0.211121	0.544267	109
hsa00480	Glutathione metabolism	17577	46	10265	26.86408	30	0.216006	0.551797	110
hsa00260	Glycine, serine and threonine metabolism	17577	38	10265	22.19207	25	0.22492	0.569391	111
hsa05211	Renal cell carcinoma	17577	66	10265	38.54412	42	0.230883	0.575357	112
hsa04510	Focal adhesion	17577	201	10265	117.3844	123	0.231371	0.575357	112
hsa04725	Cholinergic synapse	17577	106	10265	61.90419	66	0.239559	0.580603	114
hsa00650	Butanoate metabolism	17577	22	10265	12.84804	15	0.239679	0.580603	114
hsa04966	Collecting duct acid secretion	17577	22	10265	12.84804	15	0.239679	0.580603	114
hsa04971	Gastric acid secretion	17577	68	10265	39.71212	43	0.247265	0.593859	117
hsa04930	Type II diabetes mellitus	17577	45	10265	26.28008	29	0.252443	0.601156	118
hsa05014	Amyotrophic lateral sclerosis (ALS)	17577	50	10265	29.20009	32	0.256041	0.604601	119
hsa04919	Thyroid hormone signaling pathway	17577	115	10265	67.16021	71	0.264186	0.618634	120
hsa03050	Proteasome	17577	42	10265	24.52808	27	0.27035	0.627836	121
hsa05216	Thyroid cancer	17577	29	10265	16.93605	19	0.28047	0.646002	122
hsa00450	Selenocompound metabolism	17577	16	10265	9.344029	11	0.282813	0.646102	123
hsa00565	Ether lipid metabolism	17577	39	10265	22.77607	25	0.289971	0.657111	124
hsa05134	Legionellosis	17577	54	10265	31.5361	34	0.295658	0.662014	125
hsa04261	Adrenergic signaling in cardiomyocytes	17577	136	10265	79.42425	83	0.296846	0.662014	125
hsa04270	Vascular smooth muscle contraction	17577	111	10265	64.8242	68	0.304088	0.672824	127
hsa00400	Phenylalanine, tyrosine and tryptophan biosynthesis	17577	5	10265	2.920009	4	0.309846	0.678668	128
hsa00380	Tryptophan metabolism	17577	36	10265	21.02407	23	0.311559	0.678668	128
hsa04722	Neurotrophin signaling pathway	17577	118	10265	68.91221	72	0.315365	0.681673	130
hsa00053	Ascorbate and aldarate metabolism	17577	18	10265	10.51203	12	0.322756	0.692324	131

hsa04810	Regulation of actin cytoskeleton	17577	196	10265	114.4644	118	0.330325	0.699491	132
hsa05020	Prion diseases	17577	33	10265	19.27206	21	0.335429	0.699491	132
hsa03410	Base excision repair	17577	33	10265	19.27206	21	0.335429	0.699491	132
hsa04910	Insulin signaling pathway	17577	132	10265	77.08824	80	0.336055	0.699491	132
hsa04350	TGF-beta signaling pathway	17577	80	10265	46.72015	49	0.344959	0.712746	136
hsa05110	Vibrio cholerae infection	17577	50	10265	29.20009	31	0.357193	0.730789	137
hsa04961	Endocrine and other factor-regulated calcium reabsorption	17577	45	10265	26.28008	28	0.358893	0.730789	137
hsa04723	Retrograde endocannabinoid signaling	17577	89	10265	51.97616	54	0.373388	0.754834	139
hsa00140	Steroid hormone biosynthesis	17577	42	10265	24.52808	26	0.383607	0.762927	140
hsa04142	Lysosome	17577	118	10265	68.91221	71	0.384909	0.762927	140
hsa00460	Cyanoamino acid metabolism	17577	7	10265	4.088013	5	0.385536	0.762927	140
hsa05160	Hepatitis C	17577	113	10265	65.99221	68	0.388348	0.763118	143
hsa04370	VEGF signaling pathway	17577	59	10265	34.45611	36	0.393983	0.768815	144
hsa04920	Adipocytokine signaling pathway	17577	66	10265	38.54412	40	0.408169	0.791003	145
hsa05143	African trypanosomiasis	17577	34	10265	19.85606	21	0.415139	0.793783	146
hsa04310	Wnt signaling pathway	17577	134	10265	78.25624	80	0.415253	0.793783	146
hsa05220	Chronic myeloid	17577	73	10265	42.63213	44	0.420777	0.795318	148
hsa00670	One carbon pool by folate	17577	19	10265	11.09603	12	0.430788	0.795318	148
hsa05032	Morphine addiction	17577	80	10265	46.72015	48	0.432126	0.795318	148
hsa04727	GABAergic synapse	17577	75	10265	43.80014	45	0.437331	0.795318	148
hsa04530	Tight junction	17577	121	10265	70.66422	72	0.440553	0.795318	148
hsa00072	Synthesis and degradation of ketone bodies	17577	9	10265	5.256016	6	0.442781	0.795318	148
hsa00430	Taurine and hypotaurine metabolism	17577	9	10265	5.256016	6	0.442781	0.795318	148

hsa03018	RNA degradation	17577	70	10265	40.88013	42	0.442834	0.795318	148
hsa05205	Proteoglycans in cancer	17577	208	10265	121.4724	123	0.44371	0.795318	148
hsa04151	PI3K-Akt signaling pathway	17577	307	10265	179.2886	181	0.44497	0.795318	148
hsa00232	Caffeine metabolism	17577	4	10265	2.336007	3	0.44774	0.795318	148
hsa05166	HTLV-I	17577	251	10265	146.5845	148	0.454387	0.795318	148
hsa00030	Pentose phosphate pathway	17577	26	10265	15.18405	16	0.454525	0.795318	148
hsa04672	Intestinal immune network for IgA production	17577	43	10265	25.11208	26	0.45568	0.795318	148
hsa00330	Arginine and proline metabolism	17577	55	10265	32.1201	33	0.461633	0.795821	162
hsa04730	Long-term depression	17577	55	10265	32.1201	33	0.461633	0.795821	162
hsa04068	FoxO signaling pathway	17577	125	10265	73.00023	74	0.465753	0.798029	164
hsa05320	Autoimmune thyroid disease	17577	33	10265	19.27206	20	0.471927	0.803065	165
hsa00770	Pantothenate and CoA biosynthesis	17577	16	10265	9.344029	10	0.474409	0.803065	165
hsa04320	Dorso-ventral axis formation	17577	23	10265	13.43204	14	0.493374	0.819021	167
hsa00534	Glycosaminoglycan biosynthesis - heparan sulfate	17577	23	10265	13.43204	14	0.493374	0.819021	167
hsa05146	Amoebiasis	17577	100	10265	58.40018	59	0.494161	0.819021	167
hsa00562	Inositol phosphate metabolism	17577	59	10265	34.45611	35	0.498332	0.819021	167
hsa04962	Vasopressin-regulated water reabsorption	17577	42	10265	24.52808	25	0.507025	0.819021	167
hsa05164	Influenza A	17577	150	10265	87.60027	88	0.508483	0.819021	167
hsa00750	Vitamin B6 metabolism	17577	6	10265	3.504011	4	0.511171	0.819021	167
hsa04670	Leukocyte transendothelial migration	17577	109	10265	63.6562	64	0.514292	0.819021	167
hsa04340	Hedgehog signaling pathway	17577	49	10265	28.61609	29	0.516663	0.819021	167
hsa04015	Rap1 signaling pathway	17577	200	10265	116.8004	117	0.518851	0.819021	167

hsa00514	Other types of O-glycan biosynthesis	17577	25	10265	14.60005	15	0.520735	0.819021	167
hsa05169	Epstein-Barr virus infection	17577	195	10265	113.8804	114	0.52374	0.819021	167
hsa00603	Glycosphingolipid biosynthesis - globo series	17577	13	10265	7.592024	8	0.526966	0.819021	167
hsa04152	AMPK signaling pathway	17577	118	10265	68.91221	69	0.532819	0.819021	167
hsa00532	Glycosaminoglycan biosynthesis - chondroitin sulfate / dermatan	17577	20	10265	11.68004	12	0.537536	0.819021	167
hsa04726	Serotonergic synapse	17577	101	10265	58.98418	59	0.541225	0.819021	167
hsa04918	Thyroid hormone synthesis	17577	65	10265	37.96012	38	0.548871	0.819021	167
hsa04912	GnRH signaling pathway	17577	84	10265	49.05615	49	0.55147	0.819021	167
hsa05152	Tuberculosis	17577	158	10265	92.27229	92	0.551539	0.819021	167
hsa00250	Alanine, aspartate and glutamate metabolism	17577	34	10265	19.85606	20	0.553054	0.819021	167
hsa00310	Lysine degradation	17577	41	10265	23.94407	24	0.559355	0.819021	167
hsa04390	Hippo signaling pathway	17577	148	10265	86.43227	86	0.563779	0.819021	167
hsa00592	alpha-Linolenic acid metabolism	17577	22	10265	12.84804	13	0.564381	0.819021	167
hsa04611	Platelet activation	17577	124	10265	72.41623	72	0.568413	0.819021	167
hsa04710	Circadian rhythm	17577	29	10265	16.93605	17	0.569194	0.819021	167
hsa03022	Basal transcription factors	17577	43	10265	25.11208	25	0.578337	0.819021	167
hsa04724	Glutamatergic synapse	17577	107	10265	62.48819	62	0.579072	0.819021	167
hsa00472	D-Arginine and D-ornithine metabolism	17577	1	10265	0.584002	1	0.584002	0.819021	167
hsa00590	Arachidonic acid metabolism	17577	57	10265	33.2881	33	0.586686	0.819021	167
hsa05142	Chagas disease (American trypanosomiasis)	17577	102	10265	59.56819	59	0.587211	0.819021	167

hsa00511	Other glycan degradation	17577	17	10265	9.928031	10	0.588281	0.819021	167
hsa04612	Antigen processing and presentation	17577	64	10265	37.37612	37	0.590592	0.819021	167
hsa04650	Natural killer cell mediated cytotoxicity	17577	109	10265	63.6562	63	0.591091	0.819021	167
hsa05202	Transcriptional misregulation in cancer	17577	166	10265	96.9443	96	0.591938	0.819021	167
hsa00130	Ubiquinone and other terpenoid-quinone biosynthesis	17577	10	10265	5.840018	6	0.592522	0.819021	167
hsa04662	B cell receptor signaling pathway	17577	71	10265	41.46413	41	0.59434	0.819021	167
hsa04514	Cell adhesion molecules (CAMs)	17577	135	10265	78.84025	78	0.594591	0.819021	167
hsa04120	Ubiquitin mediated proteolysis	17577	135	10265	78.84025	78	0.594591	0.819021	167
hsa04512	ECM-receptor interaction	17577	85	10265	49.64015	49	0.601423	0.82439	205
hsa04664	Fc epsilon RI signaling pathway	17577	66	10265	38.54412	38	0.605438	0.824691	206
hsa00512	Mucin type O-Glycan biosynthesis	17577	26	10265	15.18405	15	0.611022	0.824691	206
hsa00340	Histidine metabolism	17577	26	10265	15.18405	15	0.611022	0.824691	206
hsa04623	Cytosolic DNA-sensing pathway	17577	47	10265	27.44809	27	0.613382	0.824691	206
hsa05412	Arrhythmogenic right ventricular cardiomyopathy (ARVC)	17577	68	10265	39.71212	39	0.619716	0.825635	210
hsa04660	T cell receptor signaling pathway	17577	101	10265	58.98418	58	0.619961	0.825635	210
hsa00780	Biotin metabolism	17577	3	10265	1.752005	2	0.624824	0.828187	212
hsa03010	Ribosome	17577	129	10265	75.33623	74	0.630602	0.831921	213
hsa00760	Nicotinate and nicotinamide metabolism	17577	21	10265	12.26404	12	0.636222	0.835413	214
hsa04150	mTOR signaling pathway	17577	58	10265	33.87211	33	0.645106	0.841468	215
hsa05162	Measles	17577	119	10265	69.49622	68	0.646822	0.841468	215

hsa00061	Fatty acid biosynthesis	17577	5	10265	2.920009	3	0.654582	0.84214	217
hsa00524	Butirosin and neomycin biosynthesis	17577	5	10265	2.920009	3	0.654582	0.84214	217
hsa04140	Regulation of autophagy	17577	23	10265	13.43204	13	0.65669	0.84214	217
hsa00520	Amino sugar and nucleotide sugar metabolism	17577	46	10265	26.86408	26	0.660916	0.84214	217
hsa04145	Phagosome	17577	142	10265	82.92826	81	0.662324	0.84214	217
hsa05416	Viral	17577	55	10265	32.1201	31	0.673473	0.850971	222
hsa00563	Glycosylphosphatidylinositol(GPI)-anchor biosynthesis	17577	25	10265	14.60005	14	0.675326	0.850971	222
hsa04062	Chemokine signaling pathway	17577	179	10265	104.5363	102	0.679365	0.852239	224
hsa05332	Graft-versus-host disease	17577	34	10265	19.85606	19	0.684033	0.854282	225
hsa05130	Pathogenic Escherichia coli infection	17577	52	10265	30.36809	29	0.702453	0.873404	226
hsa04621	NOD-like receptor signaling pathway	17577	54	10265	31.5361	30	0.714886	0.884947	227
hsa04742	Taste transduction	17577	31	10265	18.10406	17	0.722657	0.890643	228
hsa05150	Staphylococcus aureus infection	17577	49	10265	28.61609	27	0.731911	0.896875	229
hsa04915	Estrogen signaling pathway	17577	95	10265	55.48017	53	0.734097	0.896875	229
hsa00604	Glycosphingolipid biosynthesis - ganglio series	17577	15	10265	8.760027	8	0.747499	0.905301	231
hsa00120	Primary bile acid	17577	15	10265	8.760027	8	0.747499	0.905301	231
hsa05144	Malaria	17577	44	10265	25.69608	24	0.750659	0.905301	231
hsa04668	TNF signaling pathway	17577	110	10265	64.2402	61	0.766721	0.906351	234
hsa04940	Type I diabetes mellitus	17577	39	10265	22.77607	21	0.771474	0.906351	234
hsa04973	Carbohydrate digestion and absorption	17577	39	10265	22.77607	21	0.771474	0.906351	234
hsa04010	MAPK signaling pathway	17577	233	10265	136.0724	131	0.772505	0.906351	234
hsa04520	Adherens junction	17577	73	10265	42.63213	40	0.772738	0.906351	234

hsa05100	Bacterial invasion of epithelial cells	17577	73	10265	42.63213	40	0.772738	0.906351	234
hsa05132	Salmonella infection	17577	82	10265	47.88815	45	0.777333	0.906351	234
hsa04540	Gap junction	17577	82	10265	47.88815	45	0.777333	0.906351	234
hsa00900	Terpenoid backbone biosynthesis	17577	21	10265	12.26404	11	0.783821	0.91014	242
hsa00471	D-Glutamine and D-glutamate metabolism	17577	4	10265	2.336007	2	0.801908	0.915907	243
hsa00290	Valine, leucine and isoleucine biosynthesis	17577	4	10265	2.336007	2	0.801908	0.915907	243
hsa04144	Endocytosis	17577	194	10265	113.2964	108	0.802349	0.915907	243
hsa00591	Linoleic acid metabolism	17577	25	10265	14.60005	13	0.803766	0.915907	243
hsa04141	Protein processing in endoplasmic reticulum	17577	159	10265	92.85629	88	0.806963	0.915907	243
hsa04620	Toll-like receptor signaling pathway	17577	90	10265	52.56016	49	0.808345	0.915907	243
hsa04917	Prolactin signaling pathway	17577	67	10265	39.12812	36	0.816508	0.920354	249
hsa00790	Folate biosynthesis	17577	14	10265	8.176025	7	0.81882	0.920354	249
hsa00300	Lysine biosynthesis	17577	2	10265	1.168004	1	0.826959	0.920847	251
hsa04913	Ovarian steroidogenesis	17577	42	10265	24.52808	22	0.828908	0.920847	251
hsa00051	Fructose and mannose metabolism	17577	31	10265	18.10406	16	0.82909	0.920847	251
hsa05161	Hepatitis B	17577	130	10265	75.92024	71	0.833571	0.921835	254
hsa05330	Allograft rejection	17577	33	10265	19.27206	17	0.83654	0.921835	254
hsa05030	Cocaine addiction	17577	48	10265	28.03209	25	0.849752	0.932735	256
hsa04064	NF-kappa B signaling pathway	17577	88	10265	51.39216	47	0.85546	0.935348	257
hsa05133	Pertussis	17577	72	10265	42.04813	38	0.86187	0.938703	258
hsa02010	ABC transporters	17577	43	10265	25.11208	22	0.86809	0.941827	259
hsa05340	Primary immunodeficiency	17577	34	10265	19.85606	17	0.878287	0.943824	260

hsa00910	Nitrogen metabolism	17577	15	10265	8.760027	7	0.881227	0.943824	260
hsa05414	Dilated cardiomyopathy	17577	80	10265	46.72015	42	0.881953	0.943824	260
hsa00100	Steroid biosynthesis	17577	17	10265	9.928031	8	0.883366	0.943824	260
hsa03060	Protein export	17577	23	10265	13.43204	11	0.89202	0.949461	264
hsa05168	Herpes simplex infection	17577	153	10265	89.35228	82	0.90166	0.951892	265
hsa05321	Inflammatory bowel disease (IBD)	17577	59	10265	34.45611	30	0.904467	0.951892	265
hsa05131	Shigellosis	17577	59	10265	34.45611	30	0.904467	0.951892	265
hsa05310	Asthma	17577	24	10265	14.01604	11	0.92646	0.969426	268
hsa00785	Lipoic acid metabolism	17577	3	10265	1.752005	1	0.928027	0.969426	268
hsa05410	Hypertrophic cardiomyopathy (HCM)	17577	74	10265	43.21613	37	0.942972	0.98139	270
hsa05140	Leishmaniasis	17577	69	10265	40.29613	34	0.951026	0.98612	271
hsa04622	RIG-I-like receptor signaling pathway	17577	51	10265	29.78409	24	0.962224	0.991364	272
hsa04630	Jak-STAT signaling pathway	17577	122	10265	71.24822	62	0.96314	0.991364	272
hsa04380	Osteoclast differentiation	17577	128	10265	74.75223	65	0.966844	0.991544	274
hsa04330	Notch signaling pathway	17577	47	10265	27.44809	21	0.979537	0.999927	275
hsa04130	SNARE interactions in vesicular transport	17577	34	10265	19.85606	14	0.985995	0.999927	275
hsa05145	Toxoplasmosis	17577	115	10265	67.16021	55	0.991496	0.999927	275
hsa05034	Alcoholism	17577	153	10265	89.35228	75	0.992471	0.999927	275
hsa05203	Viral carcinogenesis	17577	188	10265	109.7923	88	0.9995	0.999927	275
hsa00531	Glycosaminoglycan degradation	17577	18	10265	10.51203	4	0.999642	0.999927	275
hsa05322	Systemic lupus erythematosus	17577	108	10265	63.0722	44	0.999927	0.999927	275
