# Supplementary material for: A network-based pathway-extending approach using DNA methylation and gene expression data to identify altered pathways
Source: Sci Rep. 2019 Aug 14;9:11853. doi: 10.1038/s41598-019-48372-1 (PMC6694157; doi:10.1038/s41598-019-48372-1)
Supplement: Supplementary file 6 — Supplementary Table S6 [file 41598_2019_48372_MOESM6_ESM.pdf]

# A network-based pathway-extending approach using DNA methylation and gene expression data to identify altered pathways

Jie Li[1], Qiaosheng Zhang[1,2,*], Zhuo Chen[1], Dechen Xu[1], and Yadong Wang[1]

[1]Harbin Institute of Technology, School of Computer Science and Technology, Harbin, 150001, P.R. China

[2]Heilongjiang Bayi Agricultural University, College of Science, Daqing, 163319, P.R. China

[*]zqs@hit.edu.cn

# All results in COAD dataset by EP-GSEA

| Pathway ID | Pathway Name | SIZE | ES | NES | NOM p-va | FDR q-val | Rank |
|---|---|---|---|---|---|---|---|
| hsa03008 | Ribosome biogenesis in eukaryotes | 175 | -0.5115 | -1.80697 | 0 | 0.05769 | 1 |
| hsa03430 | Mismatch repair | 61 | -0.61012 | -1.85449 | 0.001969 | 0.067842 | 2 |
| hsa03030 | DNA replication | 90 | -0.61849 | -1.70599 | 0.013889 | 0.082642 | 3 |
| hsa04110 | Cell cycle | 305 | -0.39263 | -1.65285 | 0.013752 | 0.090359 | 4 |
| hsa03440 | Homologous recombination | 68 | -0.50387 | -1.63363 | 0.023077 | 0.092937 | 5 |
| hsa03460 | Fanconi anemia pathway | 94 | -0.53435 | -1.65433 | 0.013917 | 0.107043 | 6 |
| hsa03410 | Base excision repair | 93 | -0.48905 | -1.70759 | 0.005803 | 0.109113 | 7 |
| hsa03420 | Nucleotide excision repair | 104 | -0.46206 | -1.56211 | 0.017544 | 0.149247 | 8 |
| hsa03020 | RNA polymerase | 72 | -0.43714 | -1.51899 | 0.027505 | 0.16789 | 9 |
| hsa04960 | Aldosterone-regulated sodium reabsorption | 96 | 0.551739 | 1.766655 | 0 | 0.170759 | 10 |
| hsa03013 | RNA transport | 348 | -0.37445 | -1.52786 | 0.011765 | 0.174726 | 11 |
| hsa00071 | Fatty acid degradation | 118 | 0.476277 | 1.781004 | 0.004149 | 0.197407 | 12 |
| hsa04978 | Mineral absorption | 100 | 0.454031 | 1.5776 | 0.008081 | 0.228104 | 13 |
| hsa03040 | Spliceosome | 271 | -0.36667 | -1.44374 | 0.035019 | 0.237013 | 14 |
| hsa00240 | Pyrimidine metabolism | 234 | -0.38079 | -1.43057 | 0.05814 | 0.23838 | 15 |
| hsa04614 | Renin-angiotensin system | 42 | 0.447096 | 1.578798 | 0.016461 | 0.23972 | 16 |
| hsa05032 | Morphine addiction | 194 | 0.431241 | 1.563405 | 0.013592 | 0.242692 | 17 |
| hsa03018 | RNA degradation | 159 | -0.32882 | -1.4533 | 0.011881 | 0.243121 | 18 |
| hsa04713 | Circadian entrainment | 234 | 0.433583 | 1.554618 | 0.024 | 0.247138 | 19 |
| hsa04720 | Long-term potentiation | 160 | 0.395656 | 1.456456 | 0.046185 | 0.249491 | 20 |
| hsa00980 | Metabolism of xenobiotics by cytochrome P450 | 112 | 0.415093 | 1.579029 | 0.016 | 0.254523 | 21 |
| hsa04918 | Thyroid hormone synthesis | 160 | 0.378137 | 1.464456 | 0.057312 | 0.255354 | 22 |
| hsa04962 | Vasopressin-regulated water reabsorption | 113 | 0.398385 | 1.4565 | 0.052941 | 0.25568 | 23 |
| hsa04730 | Long-term depression | 145 | 0.397745 | 1.442088 | 0.081081 | 0.255955 | 24 |
| hsa04725 | Cholinergic synapse | 263 | 0.39324 | 1.467131 | 0.05315 | 0.25659 | 25 |
| hsa00270 | Cysteine and methionine metabolism | 94 | -0.34411 | -1.39518 | 0.06203 | 0.258183 | 26 |
| hsa04961 | Endocrine and other factor-regulated calcium reabsorption | 128 | 0.404014 | 1.536875 | 0.012195 | 0.258446 | 27 |
| hsa05031 | Amphetamine addiction | 175 | 0.380462 | 1.458517 | 0.062753 | 0.258894 | 28 |
| hsa04920 | Adipocytokine signaling pathway | 180 | 0.357787 | 1.436347 | 0.034343 | 0.260337 | 29 |
| hsa00062 | Fatty acid elongation | 52 | 0.398455 | 1.485293 | 0.03992 | 0.260932 | 30 |
| hsa04080 | Neuroactive ligand-receptor interaction | 391 | 0.395567 | 1.442206 | 0.085828 | 0.261772 | 31 |
| hsa04726 | Serotonergic synapse | 257 | 0.371689 | 1.493765 | 0.031311 | 0.262335 | 32 |
| hsa04970 | Salivary secretion | 204 | 0.397615 | 1.467336 | 0.049407 | 0.263385 | 33 |
| hsa03015 | mRNA surveillance pathway | 204 | -0.31713 | -1.40157 | 0.022495 | 0.265544 | 34 |
| hsa00280 | Valine, leucine and isoleucine degradation | 121 | 0.399612 | 1.486846 | 0.064795 | 0.266238 | 35 |
| hsa04916 | Melanogenesis | 255 | 0.400967 | 1.538531 | 0.013861 | 0.266564 | 36 |
| hsa00650 | Butanoate metabolism | 59 | 0.377187 | 1.442915 | 0.069959 | 0.266725 | 37 |
| hsa04971 | Gastric acid secretion | 169 | 0.437632 | 1.58126 | 0.015717 | 0.267278 | 38 |
| hsa00020 | Citrate cycle (TCA cycle) | 78 | 0.577991 | 1.849658 | 0.004032 | 0.267342 | 39 |
| hsa04919 | Thyroid hormone signaling pathway | 318 | 0.347096 | 1.429726 | 0.054369 | 0.267517 | 40 |
| hsa05216 | Thyroid cancer | 85 | 0.339881 | 1.405374 | 0.040404 | 0.267857 | 41 |

| | | | | | | | |
|---|---|---|---|---|---|---|---|
| hsa04750 | Inflammatory mediator regulation of TRP channels | 240 | 0.412043 | 1.495534 | 0.034 | 0.268296 | 42 |
| hsa04972 | Pancreatic secretion | 222 | 0.392447 | 1.472837 | 0.05 | 0.268319 | 43 |
| hsa00040 | Pentose and glucuronate interconversions | 43 | 0.52965 | 1.668058 | 0.016327 | 0.268639 | 44 |
| hsa04964 | Proximal tubule bicarbonate reclamation | 51 | 0.556914 | 1.791213 | 0.001961 | 0.26882 | 45 |
| hsa04070 | Phosphatidylinositol signaling system | 222 | 0.378863 | 1.500272 | 0.02 | 0.26919 | 46 |
| hsa04911 | Insulin secretion | 191 | 0.412025 | 1.476547 | 0.07874 | 0.269515 | 47 |
| hsa04913 | Ovarian steroidogenesis | 104 | 0.390138 | 1.467827 | 0.039683 | 0.269911 | 48 |
| hsa04740 | Olfactory transduction | 72 | 0.473943 | 1.68161 | 0.005929 | 0.270526 | 49 |
| hsa04270 | Vascular smooth muscle contraction | 276 | 0.379137 | 1.406625 | 0.064386 | 0.270793 | 50 |
| hsa04727 | GABAergic synapse | 186 | 0.362212 | 1.414822 | 0.060547 | 0.271546 | 51 |
| hsa03320 | PPAR signaling pathway | 183 | 0.329114 | 1.40821 | 0.021739 | 0.272894 | 52 |
| hsa00982 | Drug metabolism - cytochrome P450 | 100 | 0.442123 | 1.650607 | 0.002037 | 0.273396 | 53 |
| hsa00053 | Ascorbate and aldarate metabolism | 36 | 0.424389 | 1.517691 | 0.047325 | 0.274943 | 54 |
| hsa00350 | Tyrosine metabolism | 83 | 0.369463 | 1.409855 | 0.063136 | 0.275039 | 55 |
| hsa00140 | Steroid hormone biosynthesis | 79 | 0.341057 | 1.386725 | 0.041037 | 0.275339 | 56 |
| hsa04022 | cGMP-PKG signaling pathway | 397 | 0.372696 | 1.415662 | 0.091977 | 0.27543 | 57 |
| hsa04320 | Dorso-ventral axis formation | 62 | 0.425354 | 1.500912 | 0.032692 | 0.277754 | 58 |
| hsa00340 | Histidine metabolism | 63 | 0.37227 | 1.388181 | 0.083495 | 0.277936 | 59 |
| hsa04744 | Phototransduction | 61 | 0.429788 | 1.623912 | 0.012146 | 0.278202 | 60 |
| hsa04921 | Oxytocin signaling pathway | 376 | 0.373117 | 1.395598 | 0.107632 | 0.279464 | 61 |
| hsa00562 | Inositol phosphate metabolism | 164 | 0.349929 | 1.416033 | 0.059524 | 0.280391 | 62 |
| hsa04724 | Glutamatergic synapse | 255 | 0.400802 | 1.509317 | 0.026 | 0.280642 | 63 |
| hsa04728 | Dopaminergic synapse | 317 | 0.349469 | 1.392053 | 0.083168 | 0.280786 | 64 |
| hsa04020 | Calcium signaling pathway | 400 | 0.360836 | 1.388956 | 0.111984 | 0.281474 | 65 |
| hsa05010 | Alzheimer,s disease | 404 | 0.379135 | 1.503257 | 0.040568 | 0.282688 | 66 |
| hsa05204 | Chemical carcinogenesis | 119 | 0.424167 | 1.590496 | 0.008247 | 0.28319 | 67 |
| hsa00830 | Retinol metabolism | 85 | 0.439633 | 1.582448 | 0.014433 | 0.283691 | 68 |
| hsa00640 | Propanoate metabolism | 95 | 0.367497 | 1.518136 | 0.022727 | 0.285813 | 69 |
| hsa04975 | Fat digestion and absorption | 81 | 0.395513 | 1.416177 | 0.079523 | 0.286048 | 70 |
| hsa04976 | Bile secretion | 147 | 0.440409 | 1.694731 | 0.001923 | 0.288524 | 71 |
| hsa05414 | Dilated cardiomyopathy | 196 | 0.394652 | 1.370009 | 0.150097 | 0.300019 | 72 |
| hsa05214 | Glioma | 177 | 0.338622 | 1.366806 | 0.084291 | 0.300903 | 73 |
| hsa04742 | Taste transduction | 64 | 0.51025 | 1.626434 | 0.007937 | 0.302045 | 74 |
| hsa04261 | Adrenergic signaling in cardiomyocytes | 337 | 0.413105 | 1.591822 | 0.01002 | 0.303259 | 75 |
| hsa04912 | GnRH signaling pathway | 228 | 0.351384 | 1.341697 | 0.107143 | 0.306536 | 76 |
| hsa05012 | Parkinson,s disease | 316 | 0.35307 | 1.359696 | 0.147239 | 0.308171 | 77 |
| hsa04932 | Non-alcoholic fatty liver disease (NAFLD) | 364 | 0.305826 | 1.342639 | 0.097561 | 0.309638 | 78 |
| hsa05215 | Prostate cancer | 251 | 0.318628 | 1.350716 | 0.078695 | 0.30993 | 79 |
| hsa04660 | T cell receptor signaling pathway | 277 | 0.365266 | 1.344777 | 0.160643 | 0.310369 | 80 |
| hsa00532 | Glycosaminoglycan biosynthesis - chondroitin sulfate / dermatan sulfate | 36 | -0.4163 | -1.35155 | 0.121673 | 0.311382 | 81 |
| hsa04917 | Prolactin signaling pathway | 178 | 0.342993 | 1.312334 | 0.133466 | 0.311607 | 82 |
| hsa00750 | Vitamin B6 metabolism | 15 | 0.504952 | 1.334052 | 0.145129 | 0.311888 | 83 |

| | | | | | | | |
|---|---|---|---|---|---|---|---|
| hsa05218 | Melanoma | 156 | 0.344741 | 1.335834 | 0.117761 | 0.313115 | 84 |
| hsa00130 | Ubiquinone and other terpenoid-quinone biosynthesis | 32 | 0.402732 | 1.309151 | 0.14862 | 0.313291 | 85 |
| hsa00561 | Glycerolipid metabolism | 118 | 0.323309 | 1.313618 | 0.067901 | 0.313391 | 86 |
| hsa00592 | alpha-Linolenic acid | 55 | 0.356273 | 1.306281 | 0.126679 | 0.314121 | 87 |
| hsa05210 | Colorectal cancer | 186 | 0.327708 | 1.35102 | 0.076321 | 0.314139 | 88 |
| hsa05030 | Cocaine addiction | 132 | 0.370578 | 1.344784 | 0.144578 | 0.315071 | 89 |
| hsa04210 | Apoptosis | 206 | 0.324804 | 1.326663 | 0.104208 | 0.315892 | 90 |
| hsa04666 | Fc gamma R-mediated phagocytosis | 237 | 0.33365 | 1.298691 | 0.152475 | 0.315951 | 91 |
| hsa04930 | Type II diabetes mellitus | 123 | 0.364714 | 1.294 | 0.162476 | 0.316008 | 92 |
| hsa00920 | Sulfur metabolism | 21 | 0.450399 | 1.31416 | 0.163934 | 0.31653 | 93 |
| hsa04146 | Peroxisome | 193 | 0.323078 | 1.295745 | 0.144 | 0.31667 | 94 |
| hsa00460 | Cyanoamino acid metabolism | 16 | 0.432403 | 1.290928 | 0.130019 | 0.317135 | 95 |
| hsa00500 | Starch and sucrose metabolism | 98 | 0.351673 | 1.352049 | 0.086 | 0.317652 | 96 |
| hsa04810 | Regulation of actin cytoskeleton | 508 | 0.321359 | 1.286214 | 0.157996 | 0.317658 | 97 |
| hsa00072 | Synthesis and degradation of ketone bodies | 22 | 0.391383 | 1.299709 | 0.114754 | 0.318085 | 98 |
| hsa05016 | Huntington,s disease | 438 | 0.299275 | 1.327934 | 0.13442 | 0.318364 | 99 |
| hsa04973 | Carbohydrate digestion and absorption | 86 | 0.368887 | 1.301371 | 0.162055 | 0.318821 | 100 |
| hsa05213 | Endometrial cancer | 139 | 0.329858 | 1.287257 | 0.138672 | 0.319468 | 101 |
| hsa05014 | Amyotrophic lateral sclerosis (ALS) | 139 | 0.331855 | 1.314377 | 0.081081 | 0.319994 | 102 |
| hsa00380 | Tryptophan metabolism | 94 | 0.316015 | 1.316252 | 0.094142 | 0.320789 | 103 |
| hsa05033 | Nicotine addiction | 68 | 0.362383 | 1.319467 | 0.15251 | 0.324127 | 104 |
| hsa05143 | African trypanosomiasis | 95 | 0.391653 | 1.316609 | 0.171657 | 0.324533 | 105 |
| hsa04723 | Retrograde endocannabinoid signaling | 216 | 0.433848 | 1.593949 | 0.011788 | 0.325749 | 106 |
| hsa04062 | Chemokine signaling pathway | 430 | 0.33713 | 1.276327 | 0.194779 | 0.330253 | 107 |
| hsa04014 | Ras signaling pathway | 513 | 0.300309 | 1.272128 | 0.159004 | 0.333637 | 108 |
| hsa04260 | Cardiac muscle contraction | 148 | 0.335133 | 1.26667 | 0.166998 | 0.334921 | 109 |
| hsa00010 | Glycolysis / Gluconeogenesis | 178 | 0.273326 | 1.248518 | 0.140206 | 0.337066 | 110 |
| hsa04130 | SNARE interactions in vesicular transport | 73 | 0.324923 | 1.266998 | 0.151822 | 0.337999 | 111 |
| hsa00260 | Glycine, serine and threonine metabolism | 114 | 0.303222 | 1.262102 | 0.132265 | 0.339076 | 112 |
| hsa04152 | AMPK signaling pathway | 327 | 0.273521 | 1.25121 | 0.110895 | 0.339424 | 113 |
| hsa04910 | Insulin signaling pathway | 355 | 0.29487 | 1.249192 | 0.154455 | 0.339431 | 114 |
| hsa04540 | Gap junction | 215 | 0.341533 | 1.25727 | 0.186508 | 0.33975 | 115 |
| hsa05142 | Chagas disease (American trypanosomiasis) | 271 | 0.334742 | 1.252747 | 0.20122 | 0.340312 | 116 |
| hsa04611 | Platelet activation | 324 | 0.344093 | 1.258992 | 0.203593 | 0.340323 | 117 |
| hsa05211 | Renal cell carcinoma | 172 | 0.322589 | 1.253482 | 0.180077 | 0.342748 | 118 |
| hsa00760 | Nicotinate and nicotinamide metabolism | 50 | 0.347909 | 1.241811 | 0.179688 | 0.344153 | 119 |
| hsa00590 | Arachidonic acid metabolism | 125 | 0.301177 | 1.237809 | 0.151261 | 0.346765 | 120 |
| hsa05223 | Non-small cell lung cancer | 162 | 0.292904 | 1.22918 | 0.14611 | 0.347374 | 121 |
| hsa00860 | Porphyrin and chlorophyll metabolism | 69 | 0.321639 | 1.232322 | 0.179641 | 0.348867 | 122 |
| hsa01040 | Biosynthesis of unsaturated fatty acids | 45 | 0.313115 | 1.229681 | 0.15 | 0.349847 | 123 |
| hsa05412 | Arrhythmogenic right ventricular cardiomyopathy | 175 | 0.337047 | 1.233468 | 0.234951 | 0.350392 | 124 |

| | | | | | | | |
|---|---|---|---|---|---|---|---|
| hsa05212 | Pancreatic cancer | 181 | 0.286695 | 1.225292 | 0.198039 | 0.350816 | 125 |
| hsa04015 | Rap1 signaling pathway | 496 | 0.309801 | 1.217231 | 0.224206 | 0.354283 | 126 |
| hsa05020 | Prion diseases | 89 | 0.322881 | 1.218565 | 0.219008 | 0.355562 | 127 |
| hsa00601 | Glycosphingolipid biosynthesis - lacto and neolacto series | 42 | 0.332376 | 1.219026 | 0.161554 | 0.357977 | 128 |
| hsa04664 | Fc epsilon RI signaling pathway | 177 | 0.314265 | 1.210692 | 0.224609 | 0.358636 | 129 |
| hsa05410 | Hypertrophic cardiomyopathy (HCM) | 177 | 0.337669 | 1.211568 | 0.2643 | 0.360461 | 130 |
| hsa00531 | Glycosaminoglycan degradation | 38 | -0.3615 | -1.24993 | 0.175097 | 0.36409 | 131 |
| hsa00100 | Steroid biosynthesis | 46 | -0.35627 | -1.25498 | 0.166337 | 0.371749 | 132 |
| hsa04012 | ErbB signaling pathway | 235 | 0.281117 | 1.200868 | 0.169261 | 0.372066 | 133 |
| hsa04120 | Ubiquitin mediated proteolysis | 332 | -0.25408 | -1.26347 | 0.050485 | 0.374538 | 134 |
| hsa03450 | Non-homologous end-joining | 39 | -0.35927 | -1.30565 | 0.123932 | 0.374811 | 135 |
| hsa00970 | Aminoacyl-tRNA biosynthesis | 121 | -0.32167 | -1.2948 | 0.128655 | 0.374906 | 136 |
| hsa04115 | p53 signaling pathway | 169 | -0.28066 | -1.2731 | 0.099609 | 0.37533 | 137 |
| hsa00052 | Galactose metabolism | 73 | 0.309385 | 1.197078 | 0.201629 | 0.375392 | 138 |
| hsa00230 | Purine metabolism | 379 | -0.26615 | -1.27869 | 0.083984 | 0.384635 | 139 |
| hsa05146 | Amoebiasis | 263 | 0.313495 | 1.184068 | 0.293204 | 0.386264 | 140 |
| hsa00600 | Sphingolipid metabolism | 80 | 0.314008 | 1.171891 | 0.246888 | 0.387464 | 141 |
| hsa04722 | Neurotrophin signaling pathway | 323 | 0.284465 | 1.173503 | 0.244576 | 0.387879 | 142 |
| hsa04010 | MAPK signaling pathway | 558 | 0.262867 | 1.165434 | 0.219067 | 0.388284 | 143 |
| hsa04144 | Endocytosis | 501 | 0.263715 | 1.169256 | 0.2251 | 0.38833 | 144 |
| hsa04066 | HIF-1 signaling pathway | 275 | 0.288631 | 1.184466 | 0.226453 | 0.388915 | 145 |
| hsa00410 | beta-Alanine metabolism | 82 | 0.265197 | 1.163122 | 0.205567 | 0.389068 | 146 |
| hsa04150 | mTOR signaling pathway | 148 | 0.284338 | 1.158824 | 0.234496 | 0.38947 | 147 |
| hsa00780 | Biotin metabolism | 9 | 0.452694 | 1.174286 | 0.252083 | 0.389847 | 148 |
| hsa00360 | Phenylalanine metabolism | 49 | 0.308338 | 1.166072 | 0.221328 | 0.39048 | 149 |
| hsa04630 | Jak-STAT signaling pathway | 283 | 0.296699 | 1.160137 | 0.283433 | 0.390483 | 150 |
| hsa04610 | Complement and coagulation cascades | 145 | 0.326494 | 1.185294 | 0.272177 | 0.39076 | 151 |
| hsa05221 | Acute myeloid leukemia | 168 | 0.307692 | 1.174721 | 0.273267 | 0.392258 | 152 |
| hsa00290 | Valine, leucine and isoleucine biosynthesis | 11 | 0.44316 | 1.175326 | 0.272152 | 0.394256 | 153 |
| hsa04370 | VEGF signaling pathway | 162 | 0.287347 | 1.176295 | 0.241107 | 0.396167 | 154 |
| hsa02010 | ABC transporters | 111 | 0.277373 | 1.143929 | 0.266402 | 0.398144 | 155 |
| hsa04068 | FoxO signaling pathway | 372 | 0.250554 | 1.147429 | 0.23301 | 0.398457 | 156 |
| hsa00232 | Caffeine metabolism | 8 | 0.517705 | 1.148921 | 0.316973 | 0.39898 | 157 |
| hsa04360 | Axon guidance | 292 | 0.282441 | 1.140507 | 0.296367 | 0.400848 | 158 |
| hsa04662 | B cell receptor signaling pathway | 192 | 0.302257 | 1.143931 | 0.306773 | 0.401077 | 159 |
| hsa04530 | Tight junction | 310 | 0.257571 | 1.149238 | 0.222868 | 0.401363 | 160 |
| hsa05145 | Toxoplasmosis | 295 | 0.298992 | 1.137039 | 0.323353 | 0.403562 | 161 |
| hsa00591 | Linoleic acid metabolism | 57 | 0.303388 | 1.128961 | 0.278226 | 0.411547 | 162 |
| hsa05160 | Hepatitis C | 310 | 0.256976 | 1.125249 | 0.284848 | 0.412085 | 163 |
| hsa04151 | PI3K-Akt signaling pathway | 755 | 0.268001 | 1.126853 | 0.314066 | 0.412183 | 164 |
| hsa00620 | Pyruvate metabolism | 114 | 0.259762 | 1.129152 | 0.258947 | 0.414088 | 165 |
| hsa05133 | Pertussis | 190 | 0.307202 | 1.117477 | 0.33871 | 0.422279 | 166 |
| hsa05034 | Alcoholism | 335 | 0.239767 | 1.112664 | 0.290837 | 0.427139 | 167 |
| hsa04670 | Leukocyte transendothelial migration | 259 | 0.295217 | 1.107979 | 0.338491 | 0.431645 | 168 |
| hsa04915 | Estrogen signaling pathway | 258 | 0.263201 | 1.10395 | 0.304264 | 0.435429 | 169 |
| hsa04974 | Protein digestion and absorption | 165 | 0.300746 | 1.096147 | 0.363107 | 0.445229 | 170 |
| hsa04310 | Wnt signaling pathway | 338 | 0.239361 | 1.09414 | 0.299257 | 0.44554 | 171 |

| | | | | | | | |
|---|---|---|---|---|---|---|---|
| hsa04510 | Focal adhesion | 510 | 0.285448 | 1.085626 | 0.366603 | 0.448067 | 172 |
| hsa00190 | Oxidative phosphorylation | 265 | 0.309018 | 1.086799 | 0.380457 | 0.448751 | 173 |
| hsa00604 | Glycosphingolipid biosynthesis - ganglio series | 37 | 0.288355 | 1.083163 | 0.350775 | 0.448914 | 174 |
| hsa00910 | Nitrogen metabolism | 37 | 0.30708 | 1.079595 | 0.351579 | 0.449167 | 175 |
| hsa05219 | Bladder cancer | 110 | 0.240918 | 1.080592 | 0.304979 | 0.450287 | 176 |
| hsa04672 | Intestinal immune network for IgA production | 101 | 0.338414 | 1.087551 | 0.384615 | 0.45053 | 177 |
| hsa04640 | Hematopoietic cell lineage | 191 | 0.32827 | 1.076898 | 0.39 | 0.45057 | 178 |
| hsa04514 | Cell adhesion molecules (CAMs) | 286 | 0.316597 | 1.075161 | 0.418327 | 0.450729 | 179 |
| hsa05205 | Proteoglycans in cancer | 524 | 0.263411 | 1.073326 | 0.358238 | 0.450799 | 180 |
| hsa05222 | Small cell lung cancer | 227 | 0.241366 | 1.088067 | 0.323529 | 0.452644 | 181 |
| hsa04721 | Synaptic vesicle cycle | 147 | 0.238521 | 1.064289 | 0.343195 | 0.460094 | 182 |
| hsa00630 | Glyoxylate and dicarboxylate metabolism | 69 | 0.295818 | 1.065751 | 0.385714 | 0.460317 | 183 |
| hsa00030 | Pentose phosphate pathway | 75 | -0.31043 | -1.18943 | 0.212806 | 0.46214 | 184 |
| hsa04710 | Circadian rhythm | 74 | 0.263755 | 1.055441 | 0.366279 | 0.465223 | 185 |
| hsa04650 | Natural killer cell mediated cytotoxicity | 264 | 0.287142 | 1.056843 | 0.395112 | 0.466056 | 186 |
| hsa00400 | Phenylalanine, tyrosine and tryptophan biosynthesis | 8 | 0.467437 | 1.058316 | 0.37797 | 0.466888 | 187 |
| hsa03022 | Basal transcription factors | 104 | -0.2709 | -1.1726 | 0.188605 | 0.475769 | 188 |
| hsa00564 | Glycerophospholipid metabolism | 187 | 0.24038 | 1.039724 | 0.391393 | 0.480965 | 189 |
| hsa04620 | Toll-like receptor signaling pathway | 229 | 0.273235 | 1.039734 | 0.404908 | 0.483841 | 190 |
| hsa04142 | Lysosome | 302 | 0.235004 | 1.041464 | 0.390196 | 0.483854 | 191 |
| hsa00900 | Terpenoid backbone biosynthesis | 55 | 0.267452 | 1.042193 | 0.395833 | 0.485591 | 192 |
| hsa05161 | Hepatitis B | 380 | 0.230508 | 1.030762 | 0.420949 | 0.492677 | 193 |
| hsa05220 | Chronic myeloid leukemia | 204 | 0.237299 | 1.023817 | 0.426386 | 0.498299 | 194 |
| hsa04380 | Osteoclast differentiation | 313 | 0.288727 | 1.023861 | 0.433468 | 0.501177 | 195 |
| hsa04914 | Progesterone-mediated oocyte maturation | 231 | 0.21814 | 1.01983 | 0.430255 | 0.501377 | 196 |
| hsa04060 | Cytokine-cytokine receptor interaction | 486 | 0.265473 | 1.008252 | 0.470588 | 0.505426 | 197 |
| hsa04977 | Vitamin digestion and absorption | 51 | 0.252821 | 1.00953 | 0.436508 | 0.506148 | 198 |
| hsa05152 | Tuberculosis | 405 | 0.248575 | 1.003659 | 0.439516 | 0.507156 | 199 |
| hsa04950 | Maturity onset diabetes of the young | 53 | 0.26733 | 1.00968 | 0.409962 | 0.508875 | 200 |
| hsa00740 | Riboflavin metabolism | 27 | 0.328972 | 1.003977 | 0.43787 | 0.509519 | 201 |
| hsa05320 | Autoimmune thyroid disease | 73 | 0.359004 | 1.010974 | 0.464503 | 0.509662 | 202 |
| hsa00790 | Folate biosynthesis | 38 | 0.30362 | 1.011567 | 0.438272 | 0.511615 | 203 |
| hsa04390 | Hippo signaling pathway | 368 | 0.220342 | 0.998861 | 0.43797 | 0.512893 | 204 |
| hsa05110 | Vibrio cholerae infection | 134 | 0.231837 | 0.993159 | 0.460317 | 0.513718 | 205 |
| hsa05340 | Primary immunodeficiency | 85 | 0.316654 | 0.995964 | 0.461538 | 0.515138 | 206 |
| hsa05200 | Pathways in cancer | 834 | 0.218072 | 0.994049 | 0.441509 | 0.515173 | 207 |
| hsa00514 | Other types of O-glycan biosynthesis | 56 | 0.239731 | 0.986447 | 0.48 | 0.522006 | 208 |
| hsa05164 | Influenza A | 402 | 0.234586 | 0.97855 | 0.481781 | 0.532601 | 209 |
| hsa00511 | Other glycan degradation | 51 | 0.264056 | 0.975998 | 0.489152 | 0.533841 | 210 |
| hsa05132 | Salmonella infection | 228 | 0.231752 | 0.972001 | 0.507968 | 0.534913 | 211 |
| hsa04114 | Oocyte meiosis | 278 | 0.205538 | 0.969658 | 0.502033 | 0.535829 | 212 |

| | | | | | | | |
|---|---|---|---|---|---|---|---|
| hsa05321 | Inflammatory bowel disease (IBD) | 139 | 0.289329 | 0.972854 | 0.477318 | 0.536353 | 213 |
| hsa05144 | Malaria | 124 | 0.301759 | 0.964113 | 0.516 | 0.542646 | 214 |
| hsa05310 | Asthma | 63 | 0.332487 | 0.959877 | 0.522772 | 0.547044 | 215 |
| hsa05330 | Allograft rejection | 76 | 0.325375 | 0.948047 | 0.509018 | 0.564115 | 216 |
| hsa00565 | Ether lipid metabolism | 83 | 0.23463 | 0.945921 | 0.552529 | 0.564735 | 217 |
| hsa04668 | TNF signaling pathway | 300 | 0.222699 | 0.940463 | 0.541502 | 0.570689 | 218 |
| hsa05203 | Viral carcinogenesis | 508 | 0.199949 | 0.933256 | 0.554241 | 0.580522 | 219 |
| hsa05206 | MicroRNAs in cancer | 421 | 0.207286 | 0.931341 | 0.572243 | 0.580791 | 220 |
| hsa05134 | Legionellosis | 152 | 0.230033 | 0.924842 | 0.54491 | 0.588515 | 221 |
| hsa00520 | Amino sugar and nucleotide sugar metabolism | 125 | 0.213092 | 0.911782 | 0.603696 | 0.60805 | 222 |
| hsa05202 | Transcriptional misregulation in cancer | 428 | 0.20314 | 0.893342 | 0.637081 | 0.637185 | 223 |
| hsa00250 | Alanine, aspartate and glutamate metabolism | 91 | -0.25644 | -1.08462 | 0.304428 | 0.645685 | 224 |
| hsa05100 | Bacterial invasion of epithelial cells | 210 | 0.209957 | 0.879945 | 0.629771 | 0.653766 | 225 |
| hsa04520 | Adherens junction | 198 | 0.210018 | 0.880092 | 0.653257 | 0.656813 | 226 |
| hsa04064 | NF-kappa B signaling pathway | 220 | 0.231615 | 0.87479 | 0.603922 | 0.660113 | 227 |
| hsa05168 | Herpes simplex infection | 396 | 0.200746 | 0.858213 | 0.619239 | 0.683692 | 228 |
| hsa05131 | Shigellosis | 169 | 0.198782 | 0.859343 | 0.719298 | 0.685005 | 229 |
| hsa00330 | Arginine and proline | 144 | 0.18334 | 0.846411 | 0.782427 | 0.693925 | 230 |
| hsa03060 | Protein export | 58 | -0.29423 | -1.0425 | 0.400387 | 0.695021 | 231 |
| hsa04140 | Regulation of autophagy | 73 | 0.196151 | 0.847372 | 0.747012 | 0.695737 | 232 |
| hsa05332 | Graft-versus-host disease | 76 | 0.282432 | 0.847978 | 0.589286 | 0.697922 | 233 |
| hsa03050 | Proteasome | 112 | -0.29968 | -1.05069 | 0.417476 | 0.701387 | 234 |
| hsa04623 | Cytosolic DNA-sensing pathway | 116 | 0.208106 | 0.827911 | 0.707566 | 0.713622 | 235 |
| hsa04145 | Phagosome | 327 | 0.207702 | 0.829137 | 0.671371 | 0.71463 | 236 |
| hsa03010 | Ribosome | 337 | -0.29209 | -0.9549 | 0.512428 | 0.715197 | 237 |
| hsa04350 | TGF-beta signaling pathway | 220 | 0.185648 | 0.830109 | 0.756098 | 0.716452 | 238 |
| hsa05140 | Leishmaniasis | 162 | 0.247161 | 0.830534 | 0.612648 | 0.719134 | 239 |
| hsa00471 | D-Glutamine and D-glutamate metabolism | 12 | -0.34273 | -0.96138 | 0.518219 | 0.721927 | 240 |
| hsa00670 | One carbon pool by folate | 59 | -0.24451 | -1.01682 | 0.41954 | 0.730472 | 241 |
| hsa05162 | Measles | 320 | 0.190714 | 0.811433 | 0.701826 | 0.738698 | 242 |
| hsa00603 | Glycosphingolipid biosynthesis - globo series | 27 | -0.28718 | -0.97223 | 0.487952 | 0.73968 | 243 |
| hsa00533 | Glycosaminoglycan biosynthesis - keratan sulfate | 28 | -0.29266 | -0.9622 | 0.496063 | 0.741129 | 244 |
| hsa00450 | Selenocompound metabolism | 41 | -0.27749 | -0.9781 | 0.490272 | 0.748657 | 245 |
| hsa05322 | Systemic lupus erythematosus | 201 | -0.229 | -0.91229 | 0.550403 | 0.749406 | 246 |
| hsa00785 | Lipoic acid metabolism | 7 | -0.35859 | -0.8778 | 0.636905 | 0.75062 | 247 |
| hsa00730 | Thiamine metabolism | 7 | 0.352401 | 0.800462 | 0.711694 | 0.754361 | 248 |
| hsa05217 | Basal cell carcinoma | 116 | -0.23718 | -0.89142 | 0.582418 | 0.757449 | 249 |
| hsa04330 | Notch signaling pathway | 117 | 0.18985 | 0.796024 | 0.821569 | 0.758609 | 250 |
| hsa05416 | Viral myocarditis | 134 | 0.22556 | 0.792937 | 0.679436 | 0.760209 | 251 |
| hsa00512 | Mucin type O-Glycan biosynthesis | 49 | 0.198493 | 0.789019 | 0.822266 | 0.763019 | 252 |
| hsa00983 | Drug metabolism - other enzymes | 74 | 0.183681 | 0.785658 | 0.884298 | 0.765169 | 253 |
| hsa05166 | HTLV-I infection | 650 | -0.18634 | -0.87872 | 0.663244 | 0.766519 | 254 |
| hsa00310 | Lysine degradation | 109 | -0.20251 | -0.91318 | 0.620482 | 0.76739 | 255 |
| hsa00061 | Fatty acid biosynthesis | 20 | -0.27387 | -0.84252 | 0.653992 | 0.767751 | 256 |

| | | | | | | | |
|---|---|---|---|---|---|---|---|
| hsa04966 | Collecting duct acid secretion | 51 | -0.26149 | -0.97937 | 0.497984 | 0.769901 | 257 |
| hsa04340 | Hedgehog signaling pathway | 112 | -0.22915 | -0.89174 | 0.559653 | 0.775382 | 258 |
| hsa05120 | Epithelial cell signaling in Helicobacter pylori infection | 179 | -0.18666 | -0.84665 | 0.757937 | 0.77688 | 259 |
| hsa04122 | Sulfur relay system | 28 | -0.24143 | -0.85512 | 0.705773 | 0.777565 | 260 |
| hsa00510 | N-Glycan biosynthesis | 121 | -0.21486 | -0.91505 | 0.603884 | 0.783867 | 261 |
| hsa00120 | Primary bile acid biosynthesis | 37 | -0.22293 | -0.82388 | 0.744813 | 0.786983 | 262 |
| hsa00534 | Glycosaminoglycan biosynthesis - heparan sulfate / heparin | 41 | -0.2554 | -0.98266 | 0.462322 | 0.787382 | 263 |
| hsa05169 | Epstein-Barr virus infection | 541 | -0.17591 | -0.80383 | 0.750996 | 0.79101 | 264 |
| hsa00430 | Taurine and hypotaurine metabolism | 20 | -0.25776 | -0.80592 | 0.736634 | 0.803449 | 265 |
| hsa04141 | Protein processing in endoplasmic reticulum | 420 | -0.15923 | -0.78455 | 0.925197 | 0.809547 | 266 |
| hsa00051 | Fructose and mannose metabolism | 85 | 0.174987 | 0.746011 | 0.927152 | 0.824697 | 267 |
| hsa00770 | Pantothenate and CoA biosynthesis | 43 | -0.21742 | -0.761 | 0.777778 | 0.834395 | 268 |
| hsa04621 | NOD-like receptor signaling pathway | 137 | 0.173995 | 0.736124 | 0.879837 | 0.836258 | 269 |
| hsa00563 | Glycosylphosphatidylinositol(GPI)-anchor biosynthesis | 55 | -0.19232 | -0.74897 | 0.849699 | 0.837733 | 270 |
| hsa04940 | Type I diabetes mellitus | 87 | 0.223212 | 0.732324 | 0.702756 | 0.837964 | 271 |
| hsa00480 | Glutathione metabolism | 108 | 0.179204 | 0.717174 | 0.883333 | 0.856479 | 272 |
| hsa05130 | Pathogenic Escherichia coli infection | 136 | 0.166964 | 0.703768 | 0.935798 | 0.871166 | 273 |
| hsa05323 | Rheumatoid arthritis | 202 | -0.1981 | -0.7144 | 0.761523 | 0.873726 | 274 |
| hsa04612 | Antigen processing and presentation | 142 | 0.181541 | 0.694023 | 0.811175 | 0.879428 | 275 |
| hsa05150 | Staphylococcus aureus infection | 105 | 0.22257 | 0.672488 | 0.735922 | 0.901032 | 276 |
| hsa04622 | RIG-I-like receptor signaling pathway | 146 | 0.152333 | 0.650913 | 0.972056 | 0.918907 | 277 |
| hsa00300 | Lysine biosynthesis | 5 | 0.307131 | 0.611434 | 0.913758 | 0.947274 | 278 |
| hsa04512 | ECM-receptor interaction | 194 | -0.17488 | -0.58913 | 0.83195 | 0.962653 | 279 |
| hsa00524 | Butirosin and neomycin biosynthesis | 17 | -0.20057 | -0.59323 | 0.968944 | 0.977905 | 280 |

# All results in COAD dataset by GSEA

| Pathway ID | Pathway Name | SIZE | ES | NES | NOM p-va | FDR q-val | Rank |
|---|---|---|---|---|---|---|---|
| hsa03008 | Ribosome biogenesis in eukaryotes | 69 | -0.75651 | -1.86578 | 0 | 0.104035 | 1 |
| hsa00670 | One carbon pool by folate | 19 | -0.54934 | -1.62001 | 0.021825 | 0.104702 | 2 |
| hsa03020 | RNA polymerase | 31 | -0.6816 | -1.60275 | 0.016194 | 0.104793 | 3 |
| hsa03030 | DNA replication | 36 | -0.73746 | -1.62583 | 0.027451 | 0.108252 | 4 |
| hsa03040 | Spliceosome | 111 | -0.61186 | -1.60703 | 0.029297 | 0.108336 | 5 |
| hsa00970 | Aminoacyl-tRNA biosynthesis | 43 | -0.59783 | -1.57918 | 0.061475 | 0.11171 | 6 |
| hsa04115 | p53 signaling pathway | 66 | -0.45274 | -1.64077 | 0.011673 | 0.113597 | 7 |
| hsa00240 | Pyrimidine metabolism | 96 | -0.49819 | -1.58375 | 0.040936 | 0.114281 | 8 |
| hsa03460 | Fanconi anemia pathway | 46 | -0.62967 | -1.62945 | 0.016598 | 0.114521 | 9 |
| hsa03420 | Nucleotide excision repair | 45 | -0.61687 | -1.64212 | 0.011742 | 0.124655 | 10 |
| hsa04110 | Cell cycle | 118 | -0.5908 | -1.75427 | 0.019531 | 0.126111 | 11 |
| hsa03013 | RNA transport | 146 | -0.6084 | -1.72557 | 0.001996 | 0.127518 | 12 |
| hsa03430 | Mismatch repair | 23 | -0.68988 | -1.64355 | 0.027944 | 0.138024 | 13 |
| hsa03410 | Base excision repair | 33 | -0.65903 | -1.6922 | 0.007859 | 0.143359 | 14 |

| | | | | | | | |
|---|---|---|---|---|---|---|---|
| hsa03022 | Basal transcription factors | 43 | -0.57468 | -1.65432 | 0.011788 | 0.144558 | 15 |
| hsa03440 | Homologous recombination | 26 | -0.67293 | -1.67165 | 0.019841 | 0.14503 | 16 |
| hsa04960 | Aldosterone-regulated sodium reabsorption | 37 | 0.60803 | 1.688319 | 0.008016 | 0.148057 | 17 |
| hsa03018 | RNA degradation | 70 | -0.58974 | -1.7743 | 0 | 0.152312 | 18 |
| hsa00650 | Butanoate metabolism | 22 | 0.686753 | 1.689906 | 0.023207 | 0.155142 | 19 |
| hsa00982 | Drug metabolism - cytochrome P450 | 54 | 0.559611 | 1.651563 | 0.02621 | 0.155911 | 20 |
| hsa00562 | Inositol phosphate metabolism | 59 | 0.482669 | 1.647393 | 0.006061 | 0.156019 | 21 |
| hsa05032 | Morphine addiction | 80 | 0.495978 | 1.63823 | 0.006061 | 0.156048 | 22 |
| hsa00860 | Porphyrin and chlorophyll metabolism | 33 | 0.560949 | 1.63343 | 0.03268 | 0.15712 | 23 |
| hsa04971 | Gastric acid secretion | 68 | 0.531096 | 1.695087 | 0.002012 | 0.1574 | 24 |
| hsa02010 | ABC transporters | 43 | 0.518176 | 1.654867 | 0.006061 | 0.15829 | 25 |
| hsa00830 | Retinol metabolism | 48 | 0.565299 | 1.663917 | 0.03125 | 0.159066 | 26 |
| hsa04022 | cGMP-PKG signaling pathway | 155 | 0.492835 | 1.624479 | 0.02454 | 0.159249 | 27 |
| hsa05012 | Parkinson,s disease | 124 | 0.603306 | 1.612429 | 0.087045 | 0.159702 | 28 |
| hsa04725 | Cholinergic synapse | 106 | 0.494283 | 1.638396 | 0.012121 | 0.161766 | 29 |
| hsa04750 | Inflammatory mediator regulation of TRP channels | 89 | 0.461321 | 1.614292 | 0.005988 | 0.162101 | 30 |
| hsa00380 | Tryptophan metabolism | 36 | 0.539032 | 1.624982 | 0.022449 | 0.163543 | 31 |
| hsa00020 | Citrate cycle (TCA cycle) | 29 | 0.663336 | 1.616058 | 0.058315 | 0.164781 | 32 |
| hsa04730 | Long-term depression | 55 | 0.518111 | 1.696745 | 0.004167 | 0.165133 | 33 |
| hsa05204 | Chemical carcinogenesis | 63 | 0.563437 | 1.655055 | 0.019068 | 0.165442 | 34 |
| hsa00053 | Ascorbate and aldarate metabolism | 18 | 0.679548 | 1.664938 | 0.014463 | 0.165447 | 35 |
| hsa04740 | Olfactory transduction | 43 | 0.519529 | 1.602382 | 0 | 0.168055 | 36 |
| hsa00062 | Fatty acid elongation | 21 | 0.575275 | 1.698906 | 0.010352 | 0.172572 | 37 |
| hsa00640 | Propanoate metabolism | 31 | 0.63592 | 1.665802 | 0.031381 | 0.173571 | 38 |
| hsa01040 | Biosynthesis of unsaturated fatty acids | 19 | 0.510915 | 1.58409 | 0.020913 | 0.173673 | 39 |
| hsa04964 | Proximal tubule bicarbonate reclamation | 22 | 0.589689 | 1.567762 | 0.031185 | 0.174679 | 40 |
| hsa04972 | Pancreatic secretion | 82 | 0.483642 | 1.560742 | 0.020325 | 0.175185 | 41 |
| hsa04724 | Glutamatergic synapse | 107 | 0.433151 | 1.562784 | 0.013807 | 0.17664 | 42 |
| hsa00980 | Metabolism of xenobiotics by cytochrome P450 | 59 | 0.513786 | 1.57146 | 0.041754 | 0.17742 | 43 |
| hsa04260 | Cardiac muscle contraction | 62 | 0.523124 | 1.568397 | 0.044872 | 0.177782 | 44 |
| hsa00280 | Valine, leucine and isoleucine degradation | 44 | 0.666023 | 1.733024 | 0.023109 | 0.177814 | 45 |
| hsa04713 | Circadian entrainment | 90 | 0.507473 | 1.584108 | 0.016495 | 0.178308 | 46 |
| hsa05010 | Alzheimer,s disease | 155 | 0.484418 | 1.55503 | 0.111111 | 0.178871 | 47 |
| hsa04261 | Adrenergic signaling in cardiomyocytes | 136 | 0.460786 | 1.589828 | 0.02444 | 0.180356 | 48 |
| hsa04720 | Long-term potentiation | 63 | 0.468006 | 1.57148 | 0.02079 | 0.181827 | 49 |
| hsa04932 | Non-alcoholic fatty liver disease (NAFLD) | 140 | 0.490776 | 1.584667 | 0.099391 | 0.182416 | 50 |
| hsa04728 | Dopaminergic synapse | 124 | 0.462448 | 1.700927 | 0.004 | 0.18275 | 51 |
| hsa00072 | Synthesis and degradation of ketone bodies | 9 | 0.747843 | 1.573355 | 0.026694 | 0.183917 | 52 |
| hsa04912 | GnRH signaling pathway | 84 | 0.480138 | 1.719188 | 0.003953 | 0.184686 | 53 |
| hsa04664 | Fc epsilon RI signaling pathway | 66 | 0.439651 | 1.517569 | 0.037698 | 0.193946 | 54 |
| hsa04744 | Phototransduction | 21 | 0.524644 | 1.533935 | 0.035644 | 0.194562 | 55 |
| hsa04146 | Peroxisome | 79 | 0.489113 | 1.51901 | 0.098 | 0.195669 | 56 |

| | | | | | | | |
|---|---|---|---|---|---|---|---|
| hsa04978 | Mineral absorption | 47 | 0.582585 | 1.702184 | 0.004228 | 0.196573 | 57 |
| hsa04742 | Taste transduction | 31 | 0.546093 | 1.520985 | 0.058704 | 0.196601 | 58 |
| hsa04913 | Ovarian steroidogenesis | 42 | 0.527666 | 1.733591 | 0.004057 | 0.196839 | 59 |
| hsa04070 | Phosphatidylinositol signaling system | 78 | 0.434513 | 1.523669 | 0.029644 | 0.196965 | 60 |
| hsa04911 | Insulin secretion | 77 | 0.446337 | 1.529375 | 0.045908 | 0.197024 | 61 |
| hsa00920 | Sulfur metabolism | 10 | 0.68529 | 1.526111 | 0.055202 | 0.197471 | 62 |
| hsa04020 | Calcium signaling pathway | 166 | 0.460376 | 1.534349 | 0.041068 | 0.19795 | 63 |
| hsa04666 | Fc gamma R-mediated phagocytosis | 90 | 0.427803 | 1.535644 | 0.047228 | 0.200594 | 64 |
| hsa04540 | Gap junction | 82 | 0.446799 | 1.502942 | 0.051125 | 0.209852 | 65 |
| hsa04726 | Serotonergic synapse | 101 | 0.50348 | 1.737195 | 0 | 0.213078 | 66 |
| hsa00350 | Tyrosine metabolism | 36 | 0.454909 | 1.488211 | 0.044625 | 0.214378 | 67 |
| hsa05031 | Amphetamine addiction | 64 | 0.408472 | 1.485611 | 0.035644 | 0.214708 | 68 |
| hsa03320 | PPAR signaling pathway | 62 | 0.483456 | 1.482552 | 0.056962 | 0.214792 | 69 |
| hsa00140 | Steroid hormone biosynthesis | 42 | 0.473428 | 1.495318 | 0.057269 | 0.215971 | 70 |
| hsa04080 | Neuroactive ligand-receptor interaction | 205 | 0.448496 | 1.489429 | 0.057082 | 0.216219 | 71 |
| hsa04270 | Vascular smooth muscle contraction | 111 | 0.455344 | 1.489754 | 0.079108 | 0.219715 | 72 |
| hsa00620 | Pyruvate metabolism | 38 | 0.488631 | 1.474172 | 0.103093 | 0.220007 | 73 |
| hsa00600 | Sphingolipid metabolism | 38 | 0.472393 | 1.476255 | 0.078947 | 0.220551 | 74 |
| hsa04723 | Retrograde endocannabinoid signaling | 89 | 0.520131 | 1.742771 | 0 | 0.229027 | 75 |
| hsa04962 | Vasopressin-regulated water reabsorption | 42 | 0.439548 | 1.456134 | 0.055446 | 0.236106 | 76 |
| hsa00360 | Phenylalanine metabolism | 17 | 0.481623 | 1.457459 | 0.060543 | 0.237687 | 77 |
| hsa00500 | Starch and sucrose metabolism | 43 | 0.572271 | 1.769816 | 0.002075 | 0.240927 | 78 |
| hsa04970 | Salivary secretion | 77 | 0.426455 | 1.439063 | 0.061983 | 0.254762 | 79 |
| hsa03015 | mRNA surveillance pathway | 81 | -0.4235 | -1.43822 | 0.074004 | 0.256277 | 80 |
| hsa00232 | Caffeine metabolism | 4 | 0.85021 | 1.440284 | 0.04908 | 0.256746 | 81 |
| hsa00040 | Pentose and glucuronate interconversions | 24 | 0.684057 | 1.74626 | 0.014056 | 0.257386 | 82 |
| hsa04012 | ErbB signaling pathway | 87 | 0.394212 | 1.431066 | 0.07014 | 0.258322 | 83 |
| hsa04910 | Insulin signaling pathway | 132 | 0.370109 | 1.420517 | 0.032787 | 0.259131 | 84 |
| hsa05033 | Nicotine addiction | 31 | 0.437238 | 1.423085 | 0.083333 | 0.259195 | 85 |
| hsa04722 | Neurotrophin signaling pathway | 118 | 0.387018 | 1.424632 | 0.072727 | 0.260394 | 86 |
| hsa00760 | Nicotinate and nicotinamide metabolism | 21 | 0.507169 | 1.42648 | 0.076613 | 0.261092 | 87 |
| hsa04921 | Oxytocin signaling pathway | 145 | 0.405189 | 1.431583 | 0.081633 | 0.261421 | 88 |
| hsa00910 | Nitrogen metabolism | 15 | 0.712446 | 1.782745 | 0.00202 | 0.262044 | 89 |
| hsa00100 | Steroid biosynthesis | 17 | -0.57131 | -1.44341 | 0.122371 | 0.262734 | 90 |
| hsa00230 | Purine metabolism | 153 | -0.33596 | -1.42361 | 0.069632 | 0.263929 | 91 |
| hsa00511 | Other glycan degradation | 17 | 0.56466 | 1.409492 | 0.125 | 0.265254 | 92 |
| hsa04919 | Thyroid hormone signaling pathway | 115 | 0.375429 | 1.411557 | 0.094118 | 0.265696 | 93 |
| hsa00561 | Glycerolipid metabolism | 47 | 0.392489 | 1.411637 | 0.06986 | 0.268942 | 94 |
| hsa04920 | Adipocytokine signaling pathway | 66 | 0.371095 | 1.404211 | 0.03992 | 0.269748 | 95 |
| hsa00983 | Drug metabolism - other enzymes | 35 | 0.438291 | 1.401841 | 0.126556 | 0.270002 | 96 |
| hsa04961 | Endocrine and other factor-regulated calcium reabsorption | 45 | 0.603805 | 1.802677 | 0 | 0.277083 | 97 |
| hsa05016 | Huntington,s disease | 168 | 0.417515 | 1.394181 | 0.181818 | 0.277447 | 98 |

| | | | | | | | |
|---|---|---|---|---|---|---|---|
| hsa00250 | Alanine, aspartate and glutamate metabolism | 34 | -0.3844 | -1.39896 | 0.065476 | 0.288272 | 99 |
| hsa04915 | Estrogen signaling pathway | 95 | 0.373349 | 1.375719 | 0.09761 | 0.296829 | 100 |
| hsa04975 | Fat digestion and absorption | 34 | 0.469936 | 1.37749 | 0.117271 | 0.297698 | 101 |
| hsa00400 | Phenylalanine, tyrosine and tryptophan biosynthesis | 5 | 0.632011 | 1.367857 | 0.116773 | 0.305418 | 102 |
| hsa04015 | Rap1 signaling pathway | 200 | 0.369729 | 1.361374 | 0.134653 | 0.307665 | 103 |
| hsa00565 | Ether lipid metabolism | 39 | 0.387169 | 1.362823 | 0.073469 | 0.309103 | 104 |
| hsa00290 | Valine, leucine and isoleucine biosynthesis | 4 | 0.741276 | 1.355492 | 0.111801 | 0.309653 | 105 |
| hsa00601 | Glycosphingolipid biosynthesis - lacto and neolacto series | 24 | 0.460747 | 1.356138 | 0.145383 | 0.312254 | 106 |
| hsa00190 | Oxidative phosphorylation | 112 | 0.526471 | 1.344455 | 0.258586 | 0.31343 | 107 |
| hsa04721 | Synaptic vesicle cycle | 58 | 0.398437 | 1.345446 | 0.1417 | 0.315315 | 108 |
| hsa04140 | Regulation of autophagy | 23 | 0.452658 | 1.34958 | 0.150101 | 0.315399 | 109 |
| hsa00071 | Fatty acid degradation | 39 | 0.733972 | 1.886367 | 0 | 0.316736 | 110 |
| hsa00010 | Glycolysis / Gluconeogenesis | 60 | 0.381945 | 1.346449 | 0.161426 | 0.317277 | 111 |
| hsa04530 | Tight junction | 121 | 0.350541 | 1.329275 | 0.11609 | 0.318139 | 112 |
| hsa00260 | Glycine, serine and threonine metabolism | 38 | 0.395094 | 1.333096 | 0.116466 | 0.319855 | 113 |
| hsa05014 | Amyotrophic lateral sclerosis (ALS) | 50 | 0.372782 | 1.325815 | 0.103376 | 0.320285 | 114 |
| hsa04062 | Chemokine signaling pathway | 179 | 0.368722 | 1.3235 | 0.17 | 0.320562 | 115 |
| hsa05142 | Chagas disease (American trypanosomiasis) | 102 | 0.370443 | 1.329979 | 0.15748 | 0.320679 | 116 |
| hsa05216 | Thyroid cancer | 29 | 0.396169 | 1.320874 | 0.129817 | 0.321372 | 117 |
| hsa04810 | Regulation of actin cytoskeleton | 196 | 0.371405 | 1.336598 | 0.111111 | 0.321471 | 118 |
| hsa00472 | D-Arginine and D-ornithine metabolism | 1 | 0.998976 | 1.333218 | 0 | 0.323144 | 119 |
| hsa04727 | GABAergic synapse | 75 | 0.361595 | 1.317058 | 0.13189 | 0.323669 | 120 |
| hsa04014 | Ras signaling pathway | 208 | 0.340917 | 1.308234 | 0.158513 | 0.330364 | 121 |
| hsa05211 | Renal cell carcinoma | 66 | 0.364628 | 1.308372 | 0.122881 | 0.333499 | 122 |
| hsa04144 | Endocytosis | 194 | 0.320038 | 1.297241 | 0.104418 | 0.342266 | 123 |
| hsa04916 | Melanogenesis | 95 | 0.344745 | 1.28582 | 0.1639 | 0.352391 | 124 |
| hsa04614 | Renin-angiotensin system | 15 | 0.469748 | 1.286884 | 0.173038 | 0.354239 | 125 |
| hsa00052 | Galactose metabolism | 29 | 0.396538 | 1.276212 | 0.171134 | 0.363029 | 126 |
| hsa00410 | beta-Alanine metabolism | 28 | 0.404714 | 1.270001 | 0.185804 | 0.365386 | 127 |
| hsa04640 | Hematopoietic cell lineage | 80 | 0.433359 | 1.271784 | 0.282869 | 0.366062 | 128 |
| hsa04120 | Ubiquitin mediated proteolysis | 135 | -0.33657 | -1.3406 | 0.079523 | 0.366187 | 129 |
| hsa04611 | Platelet activation | 124 | 0.349717 | 1.253433 | 0.206061 | 0.382334 | 130 |
| hsa05223 | Non-small cell lung cancer | 56 | 0.345514 | 1.25364 | 0.177419 | 0.385472 | 131 |
| hsa05034 | Alcoholism | 153 | 0.316794 | 1.244998 | 0.207819 | 0.39179 | 132 |
| hsa00564 | Glycerophospholipid metabolism | 85 | 0.310859 | 1.224072 | 0.180527 | 0.395286 | 133 |
| hsa05214 | Glioma | 64 | 0.333407 | 1.221698 | 0.222453 | 0.395291 | 134 |
| hsa04152 | AMPK signaling pathway | 118 | 0.303941 | 1.240052 | 0.142 | 0.395867 | 135 |
| hsa05160 | Hepatitis C | 113 | 0.309307 | 1.227015 | 0.182004 | 0.397872 | 136 |
| hsa04976 | Bile secretion | 59 | 0.547198 | 1.80631 | 0.00813 | 0.397988 | 137 |
| hsa00340 | Histidine metabolism | 26 | 0.390437 | 1.215523 | 0.23494 | 0.398082 | 138 |
| hsa04662 | B cell receptor signaling pathway | 71 | 0.369256 | 1.224244 | 0.238956 | 0.398333 | 139 |
| hsa04672 | Intestinal immune network for IgA production | 43 | 0.504898 | 1.23298 | 0.29065 | 0.399648 | 140 |
| hsa04010 | MAPK signaling pathway | 233 | 0.301273 | 1.228103 | 0.183673 | 0.399721 | 141 |

| | | | | | | | |
|---|---|---|---|---|---|---|---|
| hsa00592 | alpha-Linolenic acid | 22 | 0.394208 | 1.216385 | 0.223762 | 0.40003 | 142 |
| hsa00900 | Terpenoid backbone biosynthesis | 21 | 0.438367 | 1.20915 | 0.273684 | 0.401032 | 143 |
| hsa05310 | Asthma | 24 | 0.563356 | 1.234258 | 0.299603 | 0.401371 | 144 |
| hsa00785 | Lipoic acid metabolism | 3 | 0.734754 | 1.206188 | 0.276986 | 0.402226 | 145 |
| hsa00310 | Lysine degradation | 41 | 0.34302 | 1.22849 | 0.201961 | 0.40255 | 146 |
| hsa04670 | Leukocyte transendothelial migration | 109 | 0.346331 | 1.209761 | 0.235529 | 0.40337 | 147 |
| hsa05030 | Cocaine addiction | 48 | 0.325281 | 1.201667 | 0.189516 | 0.406261 | 148 |
| hsa03050 | Proteasome | 42 | -0.58159 | -1.3074 | 0.235405 | 0.406603 | 149 |
| hsa00520 | Amino sugar and nucleotide sugar metabolism | 46 | 0.352881 | 1.182123 | 0.261603 | 0.427865 | 150 |
| hsa04370 | VEGF signaling pathway | 59 | 0.30504 | 1.183355 | 0.195021 | 0.429299 | 151 |
| hsa00532 | Glycosaminoglycan biosynthesis - chondroitin sulfate / dermatan sulfate | 20 | -0.4886 | -1.28422 | 0.228216 | 0.430646 | 152 |
| hsa04917 | Prolactin signaling pathway | 67 | 0.323453 | 1.170876 | 0.224742 | 0.434234 | 153 |
| hsa04210 | Apoptosis | 82 | 0.301018 | 1.158488 | 0.264887 | 0.435614 | 154 |
| hsa05100 | Bacterial invasion of epithelial cells | 73 | 0.333063 | 1.174568 | 0.267465 | 0.435662 | 155 |
| hsa05143 | African trypanosomiasis | 34 | 0.384071 | 1.162823 | 0.3 | 0.435795 | 156 |
| hsa04142 | Lysosome | 118 | 0.327757 | 1.159998 | 0.276191 | 0.436385 | 157 |
| hsa05020 | Prion diseases | 33 | 0.368184 | 1.17172 | 0.255102 | 0.43644 | 158 |
| hsa04068 | FoxO signaling pathway | 125 | 0.285928 | 1.164308 | 0.222445 | 0.436824 | 159 |
| hsa04360 | Axon guidance | 125 | 0.313917 | 1.165931 | 0.278978 | 0.438028 | 160 |
| hsa00630 | Glyoxylate and dicarboxylate metabolism | 23 | 0.416307 | 1.139042 | 0.33547 | 0.461901 | 161 |
| hsa05416 | Viral myocarditis | 55 | 0.367914 | 1.121052 | 0.331384 | 0.486403 | 162 |
| hsa05145 | Toxoplasmosis | 115 | 0.315078 | 1.115925 | 0.320158 | 0.490893 | 163 |
| hsa03450 | Non-homologous end-joining | 12 | -0.5108 | -1.24207 | 0.249493 | 0.492821 | 164 |
| hsa04660 | T cell receptor signaling pathway | 101 | 0.316948 | 1.111855 | 0.320487 | 0.493612 | 165 |
| hsa05414 | Dilated cardiomyopathy | 80 | 0.335554 | 1.086745 | 0.366337 | 0.497019 | 166 |
| hsa00790 | Folate biosynthesis | 14 | 0.392804 | 1.090895 | 0.341513 | 0.497593 | 167 |
| hsa04973 | Carbohydrate digestion and absorption | 39 | 0.348378 | 1.087847 | 0.36646 | 0.498761 | 168 |
| hsa04150 | mTOR signaling pathway | 58 | 0.278832 | 1.082762 | 0.343689 | 0.499706 | 169 |
| hsa00460 | Cyanoamino acid metabolism | 7 | 0.462101 | 1.090958 | 0.360887 | 0.500861 | 170 |
| hsa05412 | Arrhythmogenic right ventricular cardiomyopathy | 68 | 0.352615 | 1.091299 | 0.366795 | 0.503723 | 171 |
| hsa04114 | Oocyte meiosis | 103 | -0.30251 | -1.22444 | 0.186538 | 0.50583 | 172 |
| hsa05213 | Endometrial cancer | 52 | 0.329904 | 1.091854 | 0.343254 | 0.506336 | 173 |
| hsa04320 | Dorso-ventral axis formation | 23 | 0.384762 | 1.093121 | 0.344898 | 0.507922 | 174 |
| hsa04066 | HIF-1 signaling pathway | 102 | 0.274153 | 1.09974 | 0.317073 | 0.508637 | 175 |
| hsa00450 | Selenocompound metabolism | 16 | -0.41019 | -1.21291 | 0.207767 | 0.509182 | 176 |
| hsa00590 | Arachidonic acid metabolism | 57 | 0.31117 | 1.096566 | 0.335378 | 0.509919 | 177 |
| hsa04623 | Cytosolic DNA-sensing pathway | 47 | -0.3664 | -1.19216 | 0.248521 | 0.510101 | 178 |
| hsa04950 | Maturity onset diabetes of the young | 21 | 0.38777 | 1.093146 | 0.34413 | 0.511447 | 179 |
| hsa05132 | Salmonella infection | 82 | 0.280538 | 1.07086 | 0.359244 | 0.513723 | 180 |
| hsa04930 | Type II diabetes mellitus | 45 | 0.326301 | 1.067482 | 0.378486 | 0.515564 | 181 |
| hsa00770 | Pantothenate and CoA biosynthesis | 16 | 0.395003 | 1.058752 | 0.404082 | 0.518329 | 182 |

| | | | | | | | |
|---|---|---|---|---|---|---|---|
| hsa00604 | Glycosphingolipid biosynthesis - ganglio series | 15 | 0.403453 | 1.059449 | 0.391389 | 0.520624 | 183 |
| hsa04918 | Thyroid hormone synthesis | 65 | 0.278348 | 1.061113 | 0.345382 | 0.521628 | 184 |
| hsa05206 | MicroRNAs in cancer | 148 | -0.29877 | -1.19399 | 0.23092 | 0.524741 | 185 |
| hsa05330 | Allograft rejection | 33 | 0.445298 | 1.047975 | 0.439516 | 0.531333 | 186 |
| hsa04151 | PI3K-Akt signaling pathway | 307 | 0.240038 | 1.014133 | 0.406015 | 0.57547 | 187 |
| hsa05205 | Proteoglycans in cancer | 208 | 0.266142 | 1.014429 | 0.434524 | 0.578746 | 188 |
| hsa04514 | Cell adhesion molecules (CAMs) | 135 | 0.314562 | 0.997943 | 0.493097 | 0.582542 | 189 |
| hsa05222 | Small cell lung cancer | 85 | -0.26084 | -1.05603 | 0.377778 | 0.582927 | 190 |
| hsa00730 | Thiamine metabolism | 3 | 0.562105 | 1.000069 | 0.502041 | 0.583174 | 191 |
| hsa05166 | HTLV-I infection | 251 | -0.24965 | -1.07394 | 0.329939 | 0.584379 | 192 |
| hsa05210 | Colorectal cancer | 62 | 0.258138 | 0.994072 | 0.46493 | 0.585128 | 193 |
| hsa00591 | Linoleic acid metabolism | 25 | 0.326821 | 1.000378 | 0.47166 | 0.586189 | 194 |
| hsa00471 | D-Glutamine and D-glutamate metabolism | 4 | -0.66642 | -1.14106 | 0.338086 | 0.586995 | 195 |
| hsa05215 | Prostate cancer | 87 | 0.251944 | 1.004094 | 0.45102 | 0.587515 | 196 |
| hsa05221 | Acute myeloid leukemia | 57 | 0.289787 | 0.990429 | 0.474438 | 0.587521 | 197 |
| hsa05169 | Epstein-Barr virus infection | 195 | -0.28413 | -1.13066 | 0.282051 | 0.587902 | 198 |
| hsa05131 | Shigellosis | 59 | 0.264898 | 1.000996 | 0.445629 | 0.588908 | 199 |
| hsa00130 | Ubiquinone and other terpenoid-quinone biosynthesis | 10 | 0.416938 | 0.984468 | 0.495727 | 0.589376 | 200 |
| hsa04914 | Progesterone-mediated oocyte maturation | 82 | -0.27999 | -1.10849 | 0.286561 | 0.590888 | 201 |
| hsa04977 | Vitamin digestion and absorption | 20 | 0.334365 | 0.984812 | 0.488095 | 0.592267 | 202 |
| hsa05410 | Hypertrophic cardiomyopathy (HCM) | 74 | 0.297909 | 0.979459 | 0.47619 | 0.59313 | 203 |
| hsa00603 | Glycosphingolipid biosynthesis - globo series | 13 | -0.39243 | -1.04041 | 0.402439 | 0.594667 | 204 |
| hsa00270 | Cysteine and methionine metabolism | 36 | -0.29414 | -1.05798 | 0.36553 | 0.594809 | 205 |
| hsa00524 | Butirosin and neomycin biosynthesis | 5 | 0.46158 | 0.975209 | 0.485656 | 0.596246 | 206 |
| hsa04310 | Wnt signaling pathway | 134 | -0.24933 | -1.02985 | 0.39243 | 0.597358 | 207 |
| hsa05203 | Viral carcinogenesis | 188 | -0.24656 | -1.07512 | 0.343874 | 0.5987 | 208 |
| hsa05133 | Pertussis | 72 | 0.284558 | 0.970542 | 0.471058 | 0.599525 | 209 |
| hsa05134 | Legionellosis | 54 | 0.277858 | 0.968156 | 0.496907 | 0.599539 | 210 |
| hsa00430 | Taurine and hypotaurine metabolism | 9 | -0.42729 | -1.11333 | 0.297741 | 0.600636 | 211 |
| hsa05219 | Bladder cancer | 37 | -0.31108 | -1.08261 | 0.342155 | 0.602018 | 212 |
| hsa00512 | Mucin type O-Glycan biosynthesis | 26 | 0.309866 | 0.957505 | 0.457926 | 0.60492 | 213 |
| hsa00030 | Pentose phosphate pathway | 26 | -0.37632 | -1.08959 | 0.364326 | 0.607226 | 214 |
| hsa04622 | RIG-I-like receptor signaling pathway | 51 | 0.267219 | 0.958038 | 0.510121 | 0.607659 | 215 |
| hsa05161 | Hepatitis B | 130 | 0.221601 | 0.960214 | 0.537374 | 0.6079 | 216 |
| hsa05320 | Autoimmune thyroid disease | 33 | 0.399245 | 0.949945 | 0.522449 | 0.609227 | 217 |
| hsa05332 | Graft-versus-host disease | 34 | 0.379583 | 0.951503 | 0.511066 | 0.610075 | 218 |
| hsa00780 | Biotin metabolism | 3 | 0.526477 | 0.945809 | 0.535934 | 0.61141 | 219 |
| hsa05217 | Basal cell carcinoma | 54 | -0.30927 | -1.00542 | 0.444444 | 0.623755 | 220 |
| hsa04510 | Focal adhesion | 201 | 0.272734 | 0.931804 | 0.53937 | 0.628891 | 221 |
| hsa04710 | Circadian rhythm | 29 | 0.280022 | 0.92215 | 0.558704 | 0.636417 | 222 |
| hsa04650 | Natural killer cell mediated cytotoxicity | 109 | 0.260024 | 0.924242 | 0.519763 | 0.636785 | 223 |

| | | | | | | | |
|---|---|---|---|---|---|---|---|
| hsa05152 | Tuberculosis | 158 | 0.247778 | 0.913328 | 0.521042 | 0.645865 | 224 |
| hsa04966 | Collecting duct acid secretion | 22 | 0.310527 | 0.906625 | 0.545267 | 0.652383 | 225 |
| hsa05212 | Pancreatic cancer | 66 | 0.227459 | 0.901491 | 0.612903 | 0.652852 | 226 |
| hsa05202 | Transcriptional misregulation in cancer | 166 | 0.211878 | 0.902261 | 0.598095 | 0.65531 | 227 |
| hsa04620 | Toll-like receptor signaling pathway | 90 | 0.248667 | 0.879852 | 0.593626 | 0.661277 | 228 |
| hsa04380 | Osteoclast differentiation | 128 | 0.260941 | 0.884158 | 0.534113 | 0.661438 | 229 |
| hsa05200 | Pathways in cancer | 315 | 0.204851 | 0.884588 | 0.617647 | 0.664264 | 230 |
| hsa04390 | Hippo signaling pathway | 148 | -0.23163 | -0.9534 | 0.495918 | 0.664307 | 231 |
| hsa05340 | Primary immunodeficiency | 34 | 0.335662 | 0.879922 | 0.571721 | 0.664631 | 232 |
| hsa03010 | Ribosome | 129 | -0.42893 | -0.97178 | 0.558935 | 0.664735 | 233 |
| hsa04060 | Cytokine-cytokine receptor interaction | 227 | 0.252949 | 0.888669 | 0.565657 | 0.665144 | 234 |
| hsa05146 | Amoebiasis | 100 | 0.245927 | 0.885586 | 0.584139 | 0.666399 | 235 |
| hsa05218 | Melanoma | 62 | 0.252437 | 0.889458 | 0.605691 | 0.667498 | 236 |
| hsa00740 | Riboflavin metabolism | 10 | 0.352673 | 0.868151 | 0.633065 | 0.674931 | 237 |
| hsa00750 | Vitamin B6 metabolism | 6 | -0.39916 | -0.95586 | 0.493827 | 0.67498 | 238 |
| hsa05321 | Inflammatory bowel disease (IBD) | 59 | 0.290396 | 0.850925 | 0.604167 | 0.698285 | 239 |
| hsa04122 | Sulfur relay system | 10 | 0.335519 | 0.846327 | 0.666667 | 0.701857 | 240 |
| hsa03060 | Protein export | 23 | -0.39244 | -0.90516 | 0.567194 | 0.71288 | 241 |
| hsa05144 | Malaria | 44 | 0.300056 | 0.832082 | 0.612648 | 0.71614 | 242 |
| hsa04610 | Complement and coagulation cascades | 59 | 0.263628 | 0.833995 | 0.650407 | 0.717018 | 243 |
| hsa00531 | Glycosaminoglycan degradation | 18 | 0.29832 | 0.827715 | 0.67784 | 0.718494 | 244 |
| hsa05220 | Chronic myeloid leukemia | 73 | 0.207708 | 0.824036 | 0.736196 | 0.720515 | 245 |
| hsa04340 | Hedgehog signaling pathway | 49 | -0.27 | -0.90964 | 0.538302 | 0.721054 | 246 |
| hsa04668 | TNF signaling pathway | 110 | 0.21438 | 0.816661 | 0.730612 | 0.724198 | 247 |
| hsa00480 | Glutathione metabolism | 46 | 0.245846 | 0.818348 | 0.706967 | 0.725198 | 248 |
| hsa04330 | Notch signaling pathway | 47 | 0.231212 | 0.807499 | 0.721894 | 0.734413 | 249 |
| hsa05110 | Vibrio cholerae infection | 50 | -0.23503 | -0.87428 | 0.624769 | 0.748121 | 250 |
| hsa00510 | N-Glycan biosynthesis | 49 | -0.2445 | -0.86304 | 0.616766 | 0.750742 | 251 |
| hsa05120 | Epithelial cell signaling in Helicobacter pylori infection | 65 | 0.199078 | 0.782632 | 0.819672 | 0.767447 | 252 |
| hsa04350 | TGF-beta signaling pathway | 80 | 0.192812 | 0.7642 | 0.847656 | 0.786943 | 253 |
| hsa05164 | Influenza A | 150 | 0.187766 | 0.758647 | 0.806122 | 0.787361 | 254 |
| hsa04974 | Protein digestion and absorption | 78 | 0.238099 | 0.766135 | 0.724335 | 0.787993 | 255 |
| hsa00051 | Fructose and mannose metabolism | 31 | 0.24061 | 0.755059 | 0.779736 | 0.788652 | 256 |
| hsa04630 | Jak-STAT signaling pathway | 122 | 0.20519 | 0.751469 | 0.756487 | 0.790163 | 257 |
| hsa04520 | Adherens junction | 73 | 0.221648 | 0.759237 | 0.757261 | 0.790324 | 258 |
| hsa00300 | Lysine biosynthesis | 2 | -0.52405 | -0.7845 | 0.775599 | 0.813704 | 259 |
| hsa00061 | Fatty acid biosynthesis | 5 | 0.334371 | 0.721297 | 0.84265 | 0.821781 | 260 |
| hsa04064 | NF-kappa B signaling pathway | 88 | 0.216601 | 0.721872 | 0.799163 | 0.824825 | 261 |
| hsa00330 | Arginine and proline | 55 | -0.22204 | -0.79564 | 0.758242 | 0.827419 | 262 |
| hsa05162 | Measles | 119 | -0.20295 | -0.78516 | 0.74269 | 0.828274 | 263 |
| hsa04141 | Protein processing in endoplasmic reticulum | 159 | -0.21291 | -0.79924 | 0.701349 | 0.838534 | 264 |
| hsa04130 | SNARE interactions in vesicular transport | 34 | 0.210397 | 0.702979 | 0.868369 | 0.841798 | 265 |
| hsa04512 | ECM-receptor interaction | 85 | -0.25318 | -0.75505 | 0.679916 | 0.843308 | 266 |

| | | | | | | | |
|---|---|---|---|---|---|---|---|
| hsa00533 | Glycosaminoglycan biosynthesis - keratan sulfate | 14 | -0.27507 | -0.73808 | 0.790099 | 0.852361 | 267 |
| hsa05130 | Pathogenic Escherichia coli infection | 52 | 0.188985 | 0.680917 | 0.902748 | 0.865162 | 268 |
| hsa00563 | Glycosylphosphatidylinositol(GPI)-anchor biosynthesis | 25 | 0.22273 | 0.675756 | 0.854839 | 0.86712 | 269 |
| hsa00514 | Other types of O-glycan biosynthesis | 25 | 0.218286 | 0.67212 | 0.898239 | 0.867155 | 270 |
| hsa00120 | Primary bile acid biosynthesis | 15 | -0.25385 | -0.69646 | 0.824351 | 0.892173 | 271 |
| hsa04145 | Phagosome | 142 | 0.181585 | 0.639797 | 0.849398 | 0.897047 | 272 |
| hsa05323 | Rheumatoid arthritis | 83 | -0.21175 | -0.64702 | 0.788618 | 0.919916 | 273 |
| hsa00534 | Glycosaminoglycan biosynthesis - heparan sulfate / heparin | 23 | -0.19876 | -0.60621 | 0.942623 | 0.927254 | 274 |
| hsa04621 | NOD-like receptor signaling pathway | 54 | -0.18616 | -0.65431 | 0.897331 | 0.927953 | 275 |
| hsa05140 | Leishmaniasis | 69 | 0.198148 | 0.594322 | 0.854582 | 0.934174 | 276 |
| hsa05168 | Herpes simplex infection | 153 | -0.15299 | -0.61135 | 0.950593 | 0.938524 | 277 |
| hsa04940 | Type I diabetes mellitus | 39 | -0.19578 | -0.54466 | 0.892368 | 0.953215 | 278 |
| hsa05150 | Staphylococcus aureus infection | 49 | 0.19551 | 0.484912 | 0.864341 | 0.987373 | 279 |
| hsa04612 | Antigen processing and presentation | 64 | 0.146386 | 0.45662 | 0.968815 | 0.990203 | 280 |
| hsa05322 | Systemic lupus erythematosus | 108 | 0.118428 | 0.374474 | 0.991753 | 0.995942 | 281 |
